# Microarray Analysis on Human Neuroblastoma Cells Exposed to Aluminum, β_1–42_-Amyloid or the β_1–42_-Amyloid Aluminum Complex

**DOI:** 10.1371/journal.pone.0015965

**Published:** 2011-01-27

**Authors:** Valentina Gatta, Denise Drago, Karina Fincati, Maria Teresa Valenti, Luca Dalle Carbonare, Stefano L. Sensi, Paolo Zatta

**Affiliations:** 1 Department of Biomedical Sciences, “G. d'Annunzio” University, Chieti-Pescara, Italy; 2 Functional Genetics Unit-Center of Excellence on Aging (Ce.S.I.), Chieti, Italy; 3 “Metalloproteins” Unit, Department of Biology, CNR-Institute for Biomedical Technologies, University of Padua, Padua, Italy; 4 Association of the Universities of Padua, Venice and Verona for the Development of Nanotechnologies (CIVEN), Venice, Italy; 5 Department of Biomedical and Surgical Sciences, University of Verona, Verona, Italy; 6 Molecular Neurology Unit-Ce.S.I., Chieti, Italy; 7 Department of Neuroscience and Imaging, “G. d'Annunzio” University, Chieti, Italy; 8 Department of Neurology, University of California Irvine, Irvine, California, United States of America; Nathan Kline Institute and New York University School of Medicine, United States of America

## Abstract

**Background:**

A typical pathological feature of Alzheimer's disease (AD) is the appearance in the brain of senile plaques made up of β-amyloid (Aβ) and neurofibrillary tangles. AD is also associated with an abnormal accumulation of some metal ions, and we have recently shown that one of these, aluminum (Al), plays a relevant role in affecting Aβ aggregation and neurotoxicity.

**Methodology:**

In this study, employing a microarray analysis of 35,129 genes, we investigated the effects induced by the exposure to the Aβ_1–42_-Al (Aβ-Al) complex on the gene expression profile of the neuronal-like cell line, SH-SY5Y.

**Principal Findings:**

The microarray assay indicated that, compared to Aβ or Al alone, exposure to Aβ-Al complex produced selective changes in gene expression. Some of the genes selectively over or underexpressed are directly related to AD. A further evaluation performed with Ingenuity Pathway analysis revealed that these genes are nodes of networks and pathways that are involved in the modulation of Ca^2+^ homeostasis as well as in the regulation of glutamatergic transmission and synaptic plasticity.

**Conclusions and Significance:**

Aβ-Al appears to be largely involved in the molecular machinery that regulates neuronal as well as synaptic dysfunction and loss. Aβ-Al seems critical in modulating key AD-related pathways such as glutamatergic transmission, Ca^2+^ homeostasis, oxidative stress, inflammation, and neuronal apoptosis.

## Introduction

The abnormal deposition and aggregation of β-amyloid (Aβ) in senile plaques are hallmarks features of the Alzheimer's disease (AD) brain. Senile plaques are made up of aggregates of misfolded Aβ that are associated with high concentrations of several endogenous or exogenous metal ions (Fe, Zn, Cu, and Al), but they also contain cell elements migrating from the immuno-response system [1], [2]. Metal ions have been indicated as important co-risk factors in several neurodegenerative disorders [3], [4] and, in the context of AD, recent studies have shown that they are key in accelerating Aβ oligomerization as well as modifying the neurotoxic properties of the amyloid peptide [5], [6], [7]. In that respect, we have recently shown that Fe, Zn, Cu, and Al can each specifically affect the pathogenic actions of Aβ [8]. We have also reported that, compared to other Aβ-metal complexes (Aβ-Fe, Aβ-Zn, Aβ-Cu), Aβ-Al is unique in promoting a specific form of Aβ oligomerization that has marked neurotoxic effects [8].

In this study, we have continued to explore the molecular determinants involved in the toxicity induced by the Aβ_1–42_-Al (Aβ-Al) complex. To that aim, we investigated changes in the gene expression profile of neuronal cell lines, the SH-SY5Y, exposed to either Aβ**,** the Aβ-Al complex or Al alone, against untreated cultures. SH-SY5Y cells were chosen because as cell line they offer the advantage of being a homogenous population that does not show the subtype heterogeneity present in primary neuronal cultures, a confounding factor that would make the results hard to interpreter.

Cultures were exposed to Aβ, the Aβ-Al complex or Al alone and investigated for gene profile changes by microarray analysis, a technique that provides a powerful way to identify novel genes involved in physiopathological signaling cascades. This analytical approach has been found to be particularly productive in multi-factorial disorders such as AD [9], [10], [11]. At first, we employed an analysis of the total genome (35,129 transcripts) of SH-SY5Y exposed to Aβ, the Aβ-Al complex or Al under strictly comparable experimental conditions. After this large set of data was collected, we investigated the role and function of genes that were found selectively changed by the exposure to Aβ-Al. By using the Ingenuity Pathway Analysis (IPA) we then studied key biological functions, networks, and pathways related to these genes. Finally, we analyzed the genes changed by the Aβ-Al complex that are known to be involved in AD and present in the alzgene database (http://www.alzgene.org). Results from this study provide an advanced database that may help in deciphering the genetics of AD and serves as a potential model to promote a better understanding of the pathogenic interplay between Al accumulation, Aβ aggregation, and its neurotoxicity.

## Results

### Gene expression profile of SH-SY5Y cells treated with the Aβ-Al complex

To gain insights on the global changes in gene expression produced by the exposure to the Aβ-Al complex in human SH-SY5Y neuroblastoma cells, microarray analysis was performed and results selectively compared with gene expression of cultures exposed to Aβ or Al. Cells were incubated with Aβ or the Aβ-Al complex for 24 h. In control experiments, the same cell treatment was also performed in the presence of Al at a concentration 10-fold higher than what used for the peptide exposure. Other studies have already shown that Al is a potent modulator of gene expression at both high and low concentrations [12] [13], [14]. After evaluating a total of ten experiments, transcripts expression was analyzed and genes differentially expressed by the Aβ-Al complex were selected. A gene was considered to be differentially expressed when showing an absolute value of log-ratio higher or equal to 0.5, an index that translates to a fold-change of 1.4 in transcript quantity. On a total of 28676 transcripts, the exposure to the Aβ-Al complex promoted the selective overexpression of 1535 genes while 1815 were downexpressed (Tables S1, S2). Among these genes we found a gene subset that is directly related to AD. When we matched our results with the 584 AD-related genes present in the alzgene database (http://www.alzgene.org), we found that 29 of those were upregulated and 23 downregulated (see Tables 1, 2).

**Table 1:** List of AD-related genes (http://www.alzgene.org) that are selectively overexpressed upon exposure to Aβ-Al compared to exposures to Aβ or Al alone.

Gene symbol	Aβ(Log_2_ ratio)	AβAl(Log_2_ ratio)	Al(Log_2_ ratio)	RefSeq	Description
AR	−0.15	1.94	0.42	NM_000044	Androgen receptor
NOS2A	−0.21	1.87	−0.14	NM_000625	Nitric oxide synthase, inducible (NOS, type II)
VDR	0.19	1.77	0.17	NM_000376	Vitamin D3 receptor (VDR)
NGFR	−0.12	1.18	−1.12	NM_002507	Tumor necrosis factor receptor
CCNT1	0.28	1.09	−0.06	NM_001240	Cyclin T1
IDE	−0.02	1.05	−0.16	NM_004969	Insulin-degrading enzyme
FTSJ3	0.13	1.02	0.41	NM_017647	FtsJ homolog 3
KCNJ6	−0.53	1.02	0.36	NM_002240	G protein-activated inward rectifier potassium channel 2
NOS1	0.43	1.01	−0.74	NM_000620	Nitric-oxide synthase, brain (NOS, type I)
PLCE1	0.27	0.99	0.34	NM_016341	pancreas-enriched phospholipase C
PRKAA1	−0.02	0.93	0.09	-	5′-AMP-activated protein kinase, catalytic alpha-1 chain
SLC6A3	0.22	0.90	0.35	NM_001044	Sodium-dependent dopamine transporter
MYH8	0.38	0.85	−0.21	NM_002472	Myosin heavy chain, skeletal muscle, perinatal
FDPS	−0.22	0.84	0.13	NM_002004	Farnesyl pyrophosphate synthetase
HMOX1	0.15	0.83	0.30	NM_002133	Heme oxygenase 1
CNTF	0.22	0.82	0.03	NM_170768	Ciliary neurotrophic factor
SOS2	0.32	0.79	−0.69	NM_006939	Son of sevenless protein homolog 2
ACSL4	0.40	0.74	−0.02	NM_004458	Long-chain-fatty-acid–CoA ligase 4
ADRB1	0.35	0.73	−0.28	NM_000684	Beta-1 adrenergic receptor
ABCA1	0.05	0.72	0.45	-	ATP-binding cassette, sub-family A, member 1
APOM	0.00	0.59	−0.63	NM_019101	Apolipoprotein M
GLP1R	0.36	0.55	0.21	NM_002062	Glucagon-like peptide 1 receptor precursor
CH25H	−0.14	0.55	−0.47	NM_003956	cholesterol 25-hydroxylase
ECE1	−0.06	0.51	−0.07	NM_001397	Endothelin-converting enzyme 1
ATF7	−0.07	0.51	−0.04	NM_006856	Cyclic-AMP-dependent transcription factor
F13A1	0.30	0.50	0.22	-	Coagulation factor XIII A chain precursor

**Table 2:** List of AD-related genes (http://www.alzgene.org) that are selectively downexpressed upon exposure to Aβ-Al compared to exposures to Aβ or Al alone.

Gene Symbol	Aβ(Log_2_ ratio)	AβAl(Log_2_ ratio)	Al(Log_2_ ratio)	RefSeq	Description
OLIG2	−0.09	−1.93	0.68	NM_005806	Oligodendrocyte transcription factor 2
NAT1	−0.39	−1.68	−0.24	NM_000662	Arylamine N-acetyltransferase 1
APOC3	0.67	−1.00	−0.35	NM_000040	Apolipoprotein C-III precursor
WNT8B	0.54	−0.96	0.68	NM_003393	Wnt-8b protein precursor
MME	−0.20	−0.93	−0.26	NM_007289	Neprilysin
APOD	0.36	−0.88	−0.08	NM_001647	Apolipoprotein D precursor
LRP1	−0.13	−0.84	−0.35	NM_002332	Low-density lipoprotein receptor-related protein 1 precursor
AHSG	−0.49	−0.78	−0.47	NM_001622	Alpha-2-HS-glycoprotein precursor
GAB2	−0.13	−0.78	−0.05	NM_080491	GRB2-associated binding protein 2
MMP1	−0.20	−0.73	0.27	NM_002421	Interstitial collagenase precursor
TCN1	0.25	−0.71	−0.05	NM_001062	Transcobalamin I precursor
MYST4	−0.18	−0.68	0.15	NM_012330	MYST histone acetyltransferase
TNFRSF1B	1.06	−0.67	0.16	NM_001066	Tumor necrosis factor receptor superfamily member 1B precursor
ANXA8	0.28	−0.63	0.22	-	Annexin A8
CD36	0.36	−0.61	0.35	NM_001001548	Platelet glycoprotein IV
CTSS	−0.36	−0.60	1.25	NM_004079	Cathepsin S precursor
ESR1	1.01	−0.60	−0.26	NM_000125	Estrogen receptor
SFRP5	0.43	−0.58	0.09	NM_003015	secreted frizzled-related protein 5
PIK3R1	−0.43	−0.56	−0.50	NM_181524	Phosphatidylinositol 3-kinase regulatory alpha subunit
POU2F1	0.32	−0.54	0.34	NM_002697	POU domain, class 2, transcription factor 1
TNF	0.01	−0.52	0.39	NM_000594	Tumor necrosis factor precursor (TNF-alpha)
THRA	−0.48	−0.52	−0.35	NM_003250	Thyroid hormone receptor alpha
FCER1G	−0.26	−0.52	−0.01	NM_004106	High affinity immunoglobulin epsilon receptor gamma-subunit precursor

### TaqMan real time quantitative PCR: Validation of the microarray data

To validate the microarray results, qRT-PCR analysis was performed on SH-SY5Y cells exposed to the same experimental conditions used for the microarray assay. In the case of the Aβ-Al complex exposure, genes investigated by quantitative PCR were found to be the same changed in the microarray assay (i.e.: APLP1, APLP2, MAPT; Figures S1 and S2).

### Biological functions associated with genes selectively changed by the exposure to the Aβ-Al complex

**Figure 1:**
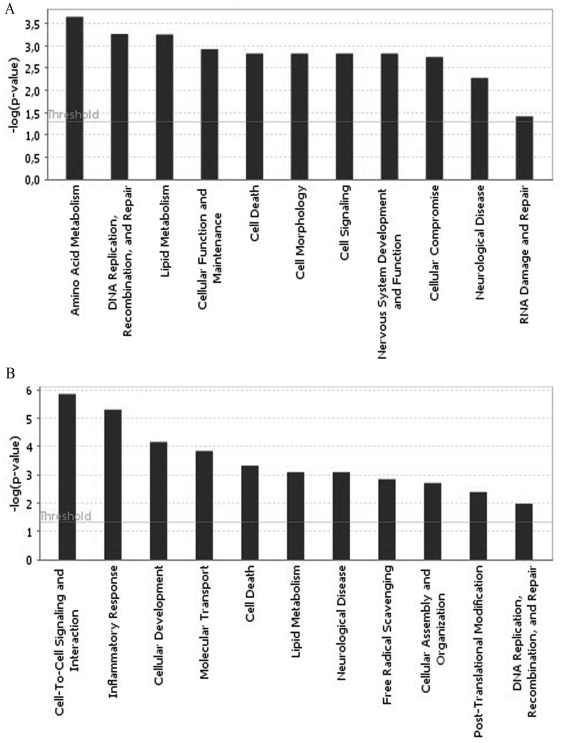
Biological functions associated with genes selectively changed by the exposure to the Aβ-Al complex. Bar charts show key biological functions associated with genes found to be selectively either overexpressed (**A**) or downexpressed (**B**) in SH-SY5Y cells exposed to Aβ-Al.

After the identification of genes selectively changed by the exposure to the Aβ-Al complex, we employed Ingenuity Pathway Analysis (IPA) to further investigate key biological functions linked to these genes. In the case of the upregulated gene dataset (n = 1535), we found that the main central nervous system (CNS) functions involved were: amino acid metabolism, DNA replication recombination and repair, lipid metabolism, cellular function and maintenance, cell death, cell morphology, cell signaling, nervous system development and function, cellular compromise, neurological disease, as well as RNA damage and repair (Figure 1A, Table 3). IPA analysis of the downregulated genes (n = 1815) showed functions in the categories of cell-to-cell signaling and interaction, inflammatory response, cellular development, molecular transport, cell death, lipid metabolism, neurological disease, free radical scavenging, cellular assembly and organization, post-translational modification as well as DNA replication recombination and repair (Figure 1B, Table 4).

**Table 3:** Central nervous system (CNS) genes present in the functional groups associated with overexpression promoted by the exposure to the Aβ-Al complex.

Category	p-value	Molecules
Amino Acid Metabolism	2,23E-04-3,86E-02	NOS1, SLC6A8, TGFBR1, ATG7, TDO2, HIPK3, BHMT, SLC1A3, NAGS, IDO2, SLC7A4, BDKRB2, FTCD, SBK1, SLC16A2, GNRH1, NOS2, SLC7A6, ARG1
DNA Replication, Recombination, Repair	5,41E-04-3,86E-02	NOS1, BDKRB2, DMC1, NOS2, NUDT15
Lipid Metabolism	5,57E-04-3,86E-02	PTGIS, RAB27B, PLA2G3, TNFSF10, ARSA, CES1 (includes EG:1066), PLCD1, BDKRB2, HMOX1, F2RL3, AR, PLCE1, ARRB1, INPP5F, PLA2G2D, ACP6, NGFR, GPLD1, GNRH1, KISS1, VDR, NOS2, LPP
Cellular Function and Maintenance	1,19E-03-3,86E-02	NOS1, SPIB, C3, STX6, SRL, CD3D, JUN, CAMK2A, CBL, AR, CXCL13, NGFR, S1PR1, GNRH1, KIT, PRDM1, VDR, FASLG
Cell Death	1,49E-03-3,86E-02	NOS1, FURIN, TGFBR1, ATF7, NEFL, TNFSF10, HMOX1, YES1, JUN, AR, PDCD1, NGFR, KIT, PRR13, NOS2, FASLG, EBAG9, ITGA2, SLC8A3, F13A1, MLANA, CLCF1, HMGA2, CTF1, NR4A1, HAS2, RARRES3, ARG1
Cell Morphology	1,49E-03-3,86E-02	NOS1, IFT88, KALRN, LMO2, RP1, PROX1, PRKG1, LAMB2, BDKRB2, AR, ESPN, NGFR, EFNB1, EFNA5, CNTNAP1, VDR, CALML3, MARCO, ARG1
Cell Signaling	1,49E-03-3,86E-02	NOS1, DSPP, STX6, F13A1, TNFSF10, KCNIP3, IL12RB2, BDKRB2, HMOX1, CAMK2A, JUN, S1PR1, VDR, NOS2, FASLG, ATP2B4, ARG1
Nervous System Development and Function	1,49E-03-3,86E-02	NOS1, POU3F1, NODAL, SLC1A3, EMX2, PRKG1, EN1, CAMK2A, AR, ABR, APLP1, EFNA5, NGFR, MAL, CDK5R2, NHLH2, ECE1, CNTNAP1, NOS2, IRX3, ZBTB16, FRS2, IFT88, KALRN, ARSA, CLCF1, LAMB2, SEPP1, PYGO2, CTF1, ARHGEF10, GNRH1, S1PR1, PTH2, ARG1, ATOH7
Cellular Compromise	1,79E-03-3,86E-02	NOS1, C3, SLC1A3, TNFSF10, PRKG1, LIMK1, BDKRB2, HMOX1, JUN, NGFR, EFNA5, EFNB1, GNRH1, KIT, CEP250, TRRAP
Neurological Disease	5,25E-03-3,86E-02	SNTG1, KIF13B, FURIN, CACNA1H, NKAIN3, IDE, MYH7B (includes EG:57644), TESC, ILDR1, CAMK2A, KIF13A, APLP1, SLC16A2, HS6ST3 (includes EG:266722), HIPK1, ZBTB16, FRS2, PTGIS, SLC24A4, CYP7B1, ATG7, APOM, KALRN, LPHN3, GPR15, IL12RB2, ZNF215, MED12, AIG1, RASL12, ARHGDIB, NRXN1, GPC5, SRGAP3, PODXL2, PCSK1, SCN1B, ST3GAL1, EHMT2, NEK4, CXCR6 (includes EG:10663), HAS2, CYCS (includes EG:54205), NR3C2, RCVRN, TSNARE1, FLJ45684, ITGA2B (includes EG:3674), UBASH3A, SCD5, MYH8, HLA-DQA1, SLC1A3, HSPB8, TNFSF10, TMEM185A, HTR1D, SEPT11, PRKG1, CORO2B, TYW1, SLC6A3, MAFG, PLCD1, CGNL1, YES1, PRKX, AR, ARRB1, ADRB1, JUN, TRHDE, GRID1, NGFR, EFNA5, MAL, ACSL4, LY75, DEGS2, EEF2K, VDR, MUC2, LPP, TRIM67, DNER, FKTN, CCDC6, CXCL6, TAAR6, CXORF48, SVIL, GLE1, GLT25D2, NR4A1, S1PR1, RAB3GAP2, COL4A4 (includes EG:1286), AK3L1, ACVR2A, ST8SIA3, STRN, T, CLTB, EDA, TRIM31, USH1C, LIMK1, SHC4, GGA1, ESPN, GRIN2C, BRSK1, SGPP2, SLC2A9, NUPR1, CNTNAP1, ACTA1, IFT88, NDUFB3, SEC14L3, C3, SESN3, PSMF1, MAST4, MYH14, ARID1B, COL2A1, CCDC129, ARSA, CHRND, SLC6A11, C7ORF57, SLC22A5, GNRH1, RARRES3, RFX2, CA11, KCNJ6, C1ORF21, CNIH3, SYNPR, AGTR1, LRRC8B, ARG1, NOS1, POU3F1, CCIN, NEFL, GPR156, FLRT2, PCDH19, EMX2, ENOX1, KIAA1409, ZNF225, OPRL1, HMOX1, PLA2G4E, FXYD7, LRGUK, KIT, NAMPT, NOS2, LASP1, CPNE9, C6ORF15, BTLA, RPGRIP1, WDR25, SCN1A, HLA-DMA, COL5A2, DSPP, MDGA1, RP1, SLC8A3, TRPA1, F13A1, CCDC85A, KCNIP1, CLCF1, PLXND1, AFF1, FDPS, SEPP1, ARHGEF10, DIP2C, HERC6, AKAP10, FOXI1, BCAS1, GRIA3
RNA Damage and Repair	3,86E-02-3,86E-02	NOS2

**Table 4:** Central nervous system (CNS) genes present in the functional groups associated with downexpression promoted by the exposure to the Aβ-Al complex.

Category	p-value	Molecules
Cell-To-Cell Signaling and Interaction	1,44E-06-2,05E-02	TAF4B, GAB2, CD3E, TGFBR3, POU2AF1, PIK3R1, LAT2, GHSR, FOXP3, CD1E, CD163, TREM2, STAB2, F2, PTPRC, HRH1, ALDH1A1, HAVCR1, PIK3CG,PPBP, TAS1R3, CD226, OSM, KLRC3, SCN5A, PTX3, KIR2DS3, GLRA2, OPRM1, CD200R1, SNAI1, OPCML, PLEC, THRA, CLEC5A, FYB, LAX1, TLR2, TNFSF8, PNLIPRP2, CDH1, PGLYRP4, UMOD, GJA3, IL10RA, PECAM1, BCL2A1, TNF, LCP2, TCL1A, ALK, ADM, MSR1, PLA2G10, KLRF1, ADIPOQ, RHOH, WNT7A, C5AR1, PDCD1LG2,APOC3, IL2, TAOK2, CD38, PRKCE, S100A8, IL27RA, MMP1, RNASE2, RELB, AHSG, MGAT5, CD36, GNAQ, ERBB3, IL1R1, IL20RB, ID3, CCL7, BCL10, CXCL12, FCER1G, DEFB129, NFATC2, TIRAP, LRP1, IFNA1, PRKCB
Inflammatory Response	5,08E-06-2,12E-02	PIK3R1, TGFBR3, CD1E, AFAP1L2, PTPRC, HRH1, CTSS, PIK3CG, PPBP, ALPP, RAB27A, CD200R1, PLEC, CLEC5A, CDH1, HLA-DRB3, IL10RA, TNF, LCP2, MME, ADM, PLA2G10, KLRF1, ADIPOQ, FOXN1, TRPM2, NFIL3, C5AR1, TAOK2, CD38, PRKCE, IL27RA, TNFRSF1B, CFP, MMP1, RNASE2, ALOX15, MX2, AHSG, GNAQ, ALOX5AP, BCL10, FCER1G, HRH4, TIRAP, LRP1, IFNA1, PRKCB, GAB2, RNASE6, CD3E, POU2AF1, SOCS6, LAT2, GHSR, FOXP3, LYZ, TREM2, CD163, IFNA6, F2, STAB2, MTTP, HAVCR1, CD226, PRR13, OSM, MGLL, SCN5A, PTX3, OPRM1, CLEC1A, LAMA2, THRA, PDE4B, HHIP, FYB, LAX1, MECOM, TLR2, PGLYRP4, UMOD, WFDC12, PECAM1, RAG1, IL22RA2, ESR1, TCL1A, MSR1, AMBP, PDCD1LG2, LYST, IL2, PRKAR1B, S100A8, RELB, CD36, MGAT5, IL1R1, IL20RB, APOL1, CCL7, CXCL12, PI3, SLC1A2, NFATC2, DEFB129
Cellular Development	7,11E-05-2,12E-02	HTR2B, PIK3R1, IL13RA2, PTPRC, HRH1, OLIG2, MOS, MCOLN3, PIK3CG, WWOX, BGLAP, HEYL, RAB27A, POU4F1, PCDH15, PITX2, OTX2, ATF2, TNFSF8, CDH1, SOSTDC1, CD5, RXFP1, IL10RA, RGL2, BCL2A1, ISL2, TNF, LCP2, CA2, ADIPOQ, TWSG1, FOXN1, EP300, CLEC11A, C5AR1, WNT7A, CD38, IL27RA, TNFRSF1B, MMP1, RNASE2, ALOX15, USH1G, POU4F2, CACNB4, TAF8, GNAQ, HIP1, ID3, SPDYA, BCL10, FCER1G, TIRAP, CHRDL1, PTPN22, IFNA1, POU5F1, PRKCB, TCF21, TAF4B, GAB2, CD3E, POU2AF1, CUL4A, SKI, LAT2, FOXP3, RNASE1, EOMES, CD163, TREM2, MEN1, CD8B, IFNA6, F2, USH1C, MBNL3, ALDH1A1, HAVCR1, CD226, MRAS, OSM, FLG, ZMYM2, TBX19, LAMA2, THRA, CITED1, LAX1, MLPH, MECOM, TLR2, GJA3, RAG1, PECAM1, SF3B4, ESR1, TCL1A, FGF5, GATA1, MSR1, PICALM, CHRDL2, RHOH, BLK, IL2, GNAT2, RELB, EMX1, CD36, ERBB3, HES3, IL1R1, NKX2-5, CXCL12, LINGO1, SLC1A2, NFATC2
Molecular Transport	1,47E-04-2,15E-02	GAB2, MRGPRD, CACNG1, CD3E, PIK3R1, LAT2, UCP1, GHSR, LYZ, F2, MTTP, PTPRC, GUCA2B, HRH1, OSTALPHA, PIK3CG, PPBP, TAS1R3, OSM, MGLL, MCHR2, ALPP, CASQ2, CHRM2, GRIK1, MAS1, RAB27A, SLC1A6, OPRM1, CLEC5A, NPY5R, MLNR, TLR2, ACADL, CD5, MCHR1, PECAM1, OSTBETA, ESR1, TNF, LCP2, FGF5, APOD, ADM, CA2, HOMER2, MSR1, SLC1A5, PLA2G10, KLRF1, ADIPOQ, RAPGEF4, EP300, TRPM2, OSCAR, CLEC11A, WNT7A, C5AR1, IL2, APOC3, GNAT2, IBTK, PRKCE, CD38, S100A8, AVPR1B, MMP1, ALOX15, CNGA2, NOX5, SLC25A2, GNAQ, CD36, ALOX5AP, HIP1, IL1R1, TRPC7, EPB41, CCL7, CXCL12, FCER1G, NFATC2, NR5A2, SLC1A2, DGAT1, HRH4, LRP1, PRKCB
Cell Death	4,82E-04-2,09E-02	TCF21, TAF4B, GAB2, CRYAB, CD3E, POU2AF1, PIK3R1, UCP1, TREM2, F2, PTPRC, HAVCR1, PIK3CG, PPBP, CD226, OSM, PTX3, POU4F1, TNFSF8, POU2F1, UMOD, CD5, BCL2A1, ESR1, TNF, ALK, FGF5, GATA1, MSR1, PLA2G10, ADIPOQ, BOK, C8A, BRAF, CLEC11A, NFIL3, PDCD1LG2, IL2, PRKCE, S100A8, IL27RA, TNFRSF1B, SLC4A1, POU4F2, CD36, ERBB3, HIP1, IL1R1, ID3, BCL10, CXCL12, LINGO1, FCER1G, SLC1A2, LRP1, PRKCB
Lipid Metabolism	8,28E-04-1,97E-02	ADM, CYP4F2, PLA2G10, PIK3R1,UCP1, LGALS4,MTTP, HRH1,APOC3, IL2, PIK3CG, PPBP, CYP7A1, PRKCE, S100A8, MGLL, AGPAT9, ALPP, CHRM2, CYP4A11, AVPR1B, HSD3B2, MAS1, GNAQ, CD36, ALOX5AP, IL1R1, NPY5R, TLR2, PNLIPRP2,CXCL12, FCER1G, NFATC2, NR5A2, OSTBETA, DGAT1, ESR1, TNF, LRP1, PRKCB, APOD
Neurological Disease	8,28E-04-2,17E-02	MME, BTN1A1, F2, USH1C, HRH1, PDCD1LG2, ALDH1A1, IL2, CTSS, MOS, KCNC1, PRKCE, IL27RA, TNFRSF1B, CHRM2, USH1G, CNTN1, OPRM1, CACNB4, MGAT5, GNAQ, PCDH15, HIP1, IL1R1, NPY5R, OTX2, TLR2, GABRG2, CD5, CXCL12, SLC1A2, PECAM1, RAG1, FOXI1, C6, HRH4, TNF, ESR1, LRP1
Free Radical Scavenging	1,46E-03-1,47E-02	GAB2, NOX5, SHANK2, CD36, UCP1, THRA, FNTB, F2, TLR2, HK1, IL2, PIK3CG, CXCL12, PPBP, FCER1G, PECAM1, TNF, LCP2, PRKCB
Cellular Assembly and Organization	1,99E-03-1,82E-02	RAB27A, CRYAB, CD3E, SNAI1, PLEC, HIP1, F2, MLPH, CDH1, C5AR1, CXCL12, FCER1G, PECAM1, ESR1, LRP1
Post-Translational Modification	4,14E-03-1,82E-02	ADM, CNTN1, CD3E, CD36, HIP1, MEN1, EP300, AFAP1L2, PTPRC, FAM129A, CD5, IL2,
DNA Replication, Recombination, Repair	1,07E-02-1,82E-02	BRAF, GPHA2, NOX5, GATA1, POU2F1, IL2, PIK3CG, MEN1, ESR1, TNF, F2, ATF2

### Networks associated with genes selectively changed by the exposure to the Aβ-Al complex

IPA predicts functional networks based on known protein-protein and functional interactions. IPA infers and ranks networks by a score, expressed as a numerical value, which is a probabilistic fit between the amount of focus genes that are potentially eligible for a network composition and present on a given gene list, the size of the network, as well as all the molecules present in the Ingenuity Knowledge Base that can be part of such a network. We therefore employed IPA to study how the genes selectively changed in their expression by the Aβ-Al exposure were interacting in specific networks.

#### Networks associated with upregulated genes

**Figure 2:**
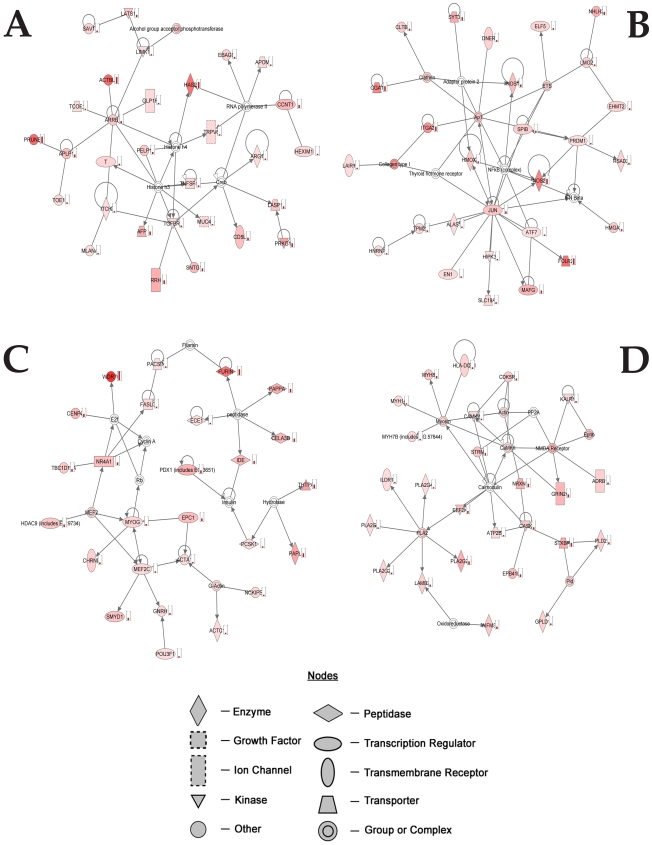
Top networks generated by IPA associated with genes selectively overexpressed by the exposure to the Aβ-Al complex. Genes in red show increased expression in SH-SY5Y cells exposed to Aβ-Al when compared with untreated SH-SY5Y cells. Arrows indicate that a molecule acts on another while lines indicate that a molecule binds to another. Small histograms indicate changes in gene expression.

Analysis of the upregulated genes indicated 25 networks with a score ranging from 46 to 13, the four top networks generated by IPA are provided in Fig. 2 and Tables S3, S4, S5, S6.

The first network (Figure 2A, Table S3) involves nodes of genes that participate in the inflammatory response (TGFB1) as well as in synaptic functioning (ARRB1 and APLP1).

The second network (Figure 2B, Table S4) involves nodes of genes that play an important role in oxidative stress (NOS), endocytosis (clathrin), inflammatory response (AP1), and apoptosis (JUN).

The third network (Figure 2C and Table S5) involves nodes of genes that serve a role in AβPP processing (Furin), synapse development (MEF2), neuroprotection (NR41), insulin metabolism (IDE), and histones deacetylation (HDAC9).

The fourth network (Figure 2D and Table S6) is built on nodes of genes that participate in the cleavage of fatty acids (PLA2), in calcium signalling (calmodulin) as well as in glutamatergic neurotransmission (GRIN1, GRIN2c).

#### Networks associated with downregulated genes

**Figure 3:**
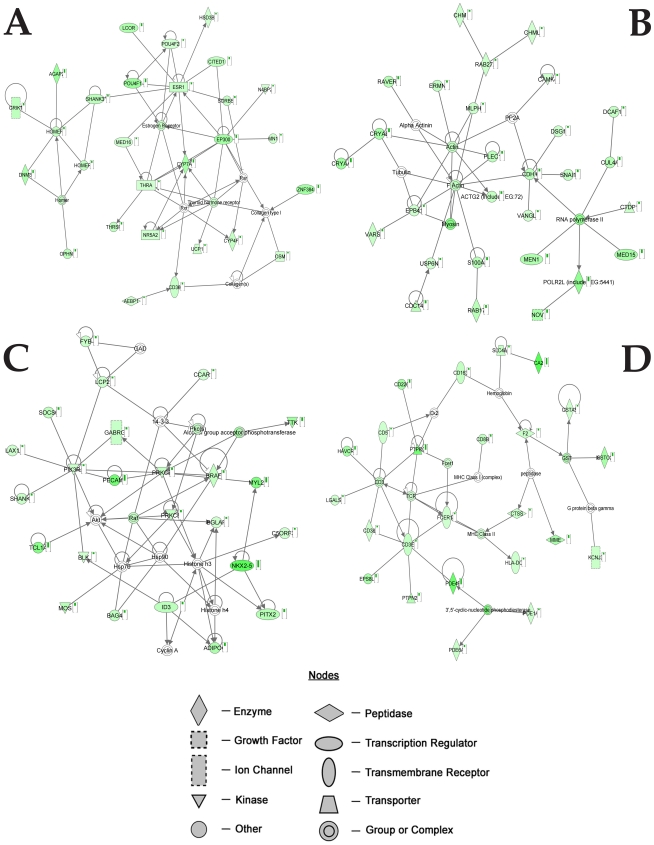
Top networks generated by IPA associated with genes selectively downexpressed by the exposure to the Aβ-Al complex. Genes in green show decreased expression in SH-SY5Y cells exposed to Aβ-Al when compared with untreated SY5Y cells. Arrows indicate that a molecule acts on another while lines indicate that a molecule binds to another. Small histograms indicate changes in gene expression.

Analysis of the downregulated genes showed 25 networks with a score ranging from 39 to 14. The four top networks are provided in Figure 3 and Tables S7, S8, S9, S10.

The first network (Fig. 3A and Table S7) is centered on nodes of genes involved in the morphological and functional integrity of synapses (Homer, Homer I), preservation of cholinergic activity (ESR1), and gene expression (EP300).The second network (Figure 3B and Table S8) shows nodes involved in apoptosis (CDH1) and cell motility (G-actin).

The third highest ranked network (Figure 3C and Table S9) is focused on down regulated genes that are involved in biological functions related to aminoacid metabolism and post translational modification. The main network nodes are represented by Protein kinase C (PKC) and SHANK2.

The fourth down regulated network (Figure 3D; Table S10) is centered on nodes of genes that are playing a role in the inflammatory response (CD3) and oxidative stress (GST).

### Pathways associated with genes selectively changed by the exposure to the Aβ-Al complex

We performed a pathway analysis of the genes selectively modulated by the exposure to the Aβ-Al complex. This analysis reveals two main pathways that are likely involved in AD.

**Figure 4:**
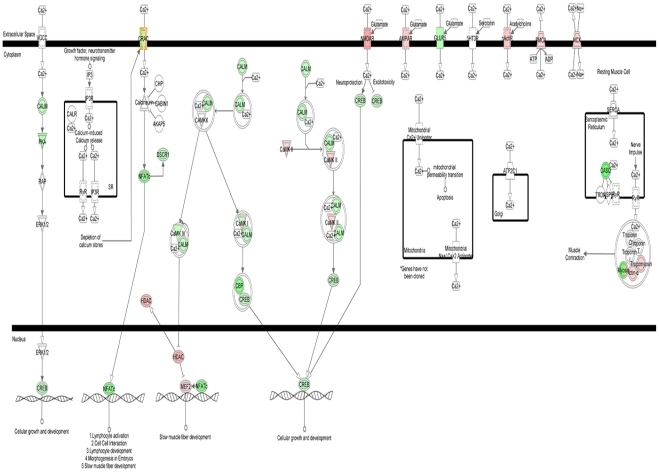
IPA-generated pathways associated with genes selectively over or downexpressed upon exposure to Aβ-Al: modulation of glutamatergic transmission and synaptic plasticity. IPA-generated pathways as resulting from the analysis of gene changes in SH-SY5Y cells exposed to the Aβ-Al when compared with untreated SH-SY5Y cells. Overexpressed genes are depicted in red, downexpressed genes are in green while genes in white are the ones inferred by IPA.

The first pathway pertains to the regulation of intracellular Ca^2+^ ([Ca^2+^]_i_) homeostasis, a mechamism that has functional consequences on neuronal and dendritic/spine loss as well as in the maintaining of Long-term Potentiation (LTP) and memory (Figure 4). This pathway shows the upregulation of genes encoding for key proteins like glutamate receptor subunits (GRIN1, GRIN2c, and GRIA), CAMKII, HDAC, MEF2, Actin alfa, as well as NCX, the gene encoding for the Na^+^-Ca^2+^ exchanger, PMCA that encodes for the Na^+^-Ca^2+^/ATPase pump as well as nAchR that encodes for the nicotinic receptor, a key receptor involved in AD [15]. Interestingly, this pathway also shows a series of downregulated genes that are critical for synaptic plasticity. Among these genes, we find CALM, the gene encoding for calmodulin, a key regulator of transcription, memory and neuronal survival, PKC, as well as GLUR5 (i.e.: GRIK1) that encodes for a kainate receptor subunit, CASQ encoding for calsequestrin, a mitochondrial calcium-binding protein [16], CREB encoding for the Cyclic AMP Response Element Binding, a key transcriptor factor involved in neuronal plasticity and LTP. We also find CBP that encodes for the CREB-binding protein and is a co-activator interacting with CREB, thereby reinforcing the modulation of LTP. Moreover, we find a downexpression of NFTAc encoding for a component of the nuclear factor of activated T cells DNA-binding transcription complex. Intriguingly, in cultured neurons as well as in the adult mouse brain, NFTA activation promotes dendritic alterations and spine loss [17]. In addition we see a downregulation of DSCR1, an oxidative stress-response gene that represses calcineurin (CaN), ultimately leading to increased CaN activity, a phenomenon that has been shown to induce synaptic dysfunction and memory impairment [18].

Finally, we find a downregulation of CRAC that encodes for activation of store-operated Ca^2+^ release-activated Ca^2+^channels. CRACs are critical channels controlling capacitative Ca^2+^ entry and the maintaining of Ca^2+^ homeostasis when the cation is depleted from intracellular stores [19].

**Figure 5:**
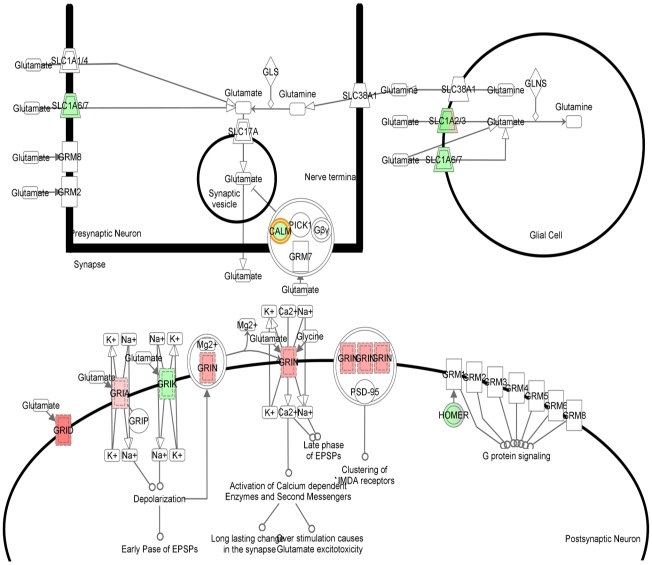
IPA-generated pathways associated with genes selectively over or downexpressed upon exposure to Aβ-Al: modulation of Ca^2**+**^ homeostasis. IPA-generated pathways as resulting from the analysis of gene changes in SH-SY5Y cells exposed to the Aβ-Al when compared with untreated SH-SY5Y cells. Overexpressed genes are depicted in red, downexpressed genes are in green while genes in white are the ones inferred by IPA.

The second pathway inferred by IPA is functionally related to the first one and shows genes that are controlling glutamatergic neurotransmission. In the second pathway we find upregulated genes encoding for NMDA and AMPA receptor subunits like GRIN, GRIA and GRID as well as downregulated genes, like HOMER, calmodulin. Downregulated are also SLC1A 6/7 and SLC1A 2/3, that encode for glutamate transporters as well as GRIK a gene encoding for kainate receptors (Figure 5).

The involvement of these pathways in AD pathogenesis is further analyzed below in the discussion.

## Discussion

The IPA-generated network and pathway analysis reveals that, compared to Aβ or Al alone, the Aβ-Al complex selectively modulates the expression of genes that can have an important role in AD pathogenesis.

For clarity, we have broken down the discussion on specific genes, analysis of networks, and pathways.

### Gene analysis

#### Upregulated genes of the first network

TGFB1 encodes for the Golgi-specific brefeldin A-resistance guanine nucleotide exchange factor 1 protein that forms a heteromeric complex with the TGF-beta receptors type II and participates in the transforming growth factor (TGF)-1 signaling cascade. TGF Beta -1 can have a role in AD as experiments in AD transgenic mice have indicated that the protein promotes a marked reduction in brain accumulation of AβPP through the activation of microglia [20]. Thus, TGFB1 overexpression may promote a protective compensatory inflammatory response that can reduce the brain amyloid load.

ARRB1 belongs to the Beta-arrestin family and modulates the desensitization and endocytosis of seven-transmembrane receptors, 7TMRs, the largest group of plasma membrane receptors [21]. In the context of AD, ARRB1 may be critically important as it can modulate the endocitosys of metabotropic glutamate receptors, mGluRs, and affect glutamatergic neurotransmission [22]. Furthermore, ARRB1 can interact with APLP1in promoting neuronal apoptosis [21].

APLP1 is a member of a protein family that also includes the Aβ precursor protein (AβPP). APLP1 encodes for a membrane-associated glycoprotein that, like AβPP, is cleaved by secretases. While AβPP has received much attention in the context of AD as the protein is involved in neurite outgrowth, cell adhesion, synaptogenesis, synaptic plasticity and neuroprotection [23], far less studies have investigated the role of APLPs. Despite a high degree of similarity with AβPP, APLP1 lacks an amyloidogenic domain; however, APLP1 has been found in the amyloid plaques of AD brains [24], [25], [26]. Furthermore, given its synaptic localization, APLP1, like AβPP, can be involved in synaptogenesis and synaptic plasticity. APLP1 also influences AβPP endocytosis [27] and an accumulation of APLP1 has been reported in neuritic plaques of the hippocampus of AD patients [24].

#### Upregulated genes of the second network

Nitric oxide synthase (NOS) generates nitric oxide (NO) and is present in the CNS in three isoforms: neuronal Type-1 NOS (nNOS), inducible Type-2 NOS (iNOS), and endothelial Type-3 NOS (eNOS). iNOS is induced in astrocytes near the senile plaques [28] and, in transgenic AD animal models, its overexpression is an early event that precedes the appearance of amyloid plaques [29]. An increased NOS-mediated NO production can promote neuronal dysfunction and/or cell death. NO is also a major determinant of NMDAR-dependent neurotoxicity and, as such, can be a key player in the excitotoxic neuronal loss found in AD [30], [31]. Clathrin-is a key protein that regulates endocytosis. In the context of AD, several studies indicate that the endocytotic machinery plays an active role in AβPP [32]. Furthermore, clathrin-mediated endocytosis is also a regulator of the internalization of a subtype of ionotropic glutamate receptor, the AMPA receptor (AMPAR), that critically modulates the efficacy of glutamatergic neurotransmission as well as LTP and Long-term Depression [33]. Modulation of synaptic activity impinges in amyloid metabolism as sustained synaptic activation leads to a rapid increase in Aβ levels in brain interstitial fluid (ISF) while a depressesed synaptic transmission reduces Aβ ISF levels [34].

C-Jun is a component of the AP-1 protein complex and AP-1 is activated downstream of c-Jun [35]. The c-Jun N-terminal Kinase (JNK)-AP1 signaling pathway plays a key role in AD by affecting gene expression, cell proliferation, the inflammatory response as well as neuronal apoptosis [36]. In AD brains, c-Jun phosphorylation promotes the induction of JNK activity, a phenomenon that eventually triggers the pathogenic processing of AβPP by inducing its hyperphosphorylation [37]. Furthermore, the AD-related oxidative stress stimulates the activation of JNK, p53, and p38 which leads to apoptotic neuronal death [27]. Interestingly, AP1 is also involved in inflammation as experiments in cultured human brain endothelial cells exposed to Aβ, indicate that AP1 upregulates the expression of inflammatory genes like MCP-1, GRO, IL-6, and IL-1β [35]. Finally, AP1is also activated in the cerebral amyloid angiopathy associated with AD [35]. Thus, C-JUN and AP1 upregulation can have a role in the neurovascular inflammation triggered by Aβ oligomers.

#### Upregulated genes of the third network

Furin participates in the amyloid metabolism by regulating the cleavage of AβPP through α-secretase activation [38], thereby reducing Aβ production. The process is mediated by promoting the maturation of A Dysintegrin And Metalloproteases 10 (ADAM10) and ADAM 17/tumor necrosis factor-a converting enzyme (TACE), two enzymes that have α secretase activity [39], [40]. Interestingly, furin mRNA levels have been shown to be reduced in the brain of AD patients and transgenic AD mice [41].

The Myocyte enhancer factor 2 (MEF2) is a Ca^2+^-regulated transcription factor that promotes neuronal survival [42] and is also a key modulator of activity-dependent synaptic development [43]. MEF2C is a transcription factor that facilitates hippocampal-dependent learning and memory in rodents. MEF2C is positively modulated by BDNF [44] and regulates the synaptic number as well the efficacy of synaptic connections [45].

NR41 is a member of a pool of 9 neuroprotective genes called Activity-regulated Inhibitor of Death (AID) genes that have been shown to promote neuronal survival by making neuronal mitochondria more resistant to cellular stress [46].

IDE encodes the insulin degrading enzyme which, beside its role in insulin metabolism plays its part in modulating brain Aβ levels as the enzyme, with lower affinity than insulin, also recognizes Aβ as a substrate [47], [48]. Several studies have demonstrated the role of the IDE gene in AD, in fact genome-wide linkage analysis of families of late onset AD patients showed significant linkage peaks mapped near the IDE locus [10q23.33; [49]]. Moreover, a recent study showed a strong association between certain IDE polymorphisms that are able to modify brain IDE transcription levels and the development of late onset AD [50], [51]. Finally, IDE has also been found to be decreased upon aging and AD [52].

Histone deacetylases (HDACs) belong to a family of proteins that by catalyzing the deacetylation of histones plays an important role in the epigenetic brain regulation of transcription, apoptosis as well as learning and memory [53], [54]. Interestingly, many neuroprotective genes such as BDNF, GDNF, HSP70, a-synuclein, Bcl-2, Bcl-XL, and p21 are modulated by HDAC inhibition. Furthermore, several findings indicate that HDAC inhibition in AD animal models restores the histone hypoacetylation, increases synaptic plasticity, decreases Aβ production and tau hyperphosphorylation, improves learning and memory and reverses spatial memory deficits [55], [56]. Finally, experimental data show that overexpression of HDAC9 and HDRP, a spliced form of HDAC9, can serve a role as an antiapoptotic molecule by binding and inhibiting the apoptosis-inducing MAP kinase, JNK [57].

#### Upregulated genes of the fourth network

Phospholipase A2 (PLA2) participates in the metabolism of fatty acids [58], [59]. PLA2 modulates several signaling pathways that link oxidative stress and proinflammatory cytokines to the release of arachidonic acid and the synthesis of eicosanoids. PLA2 is also involved in intracellular membrane trafficking, differentiation, proliferation, and apoptosis [60]. The role of PLA2 in AD is complex. Reduced activity of specific subtypes of intracellular PLA2 (cPLA2 and iPLA2) are found in AD and thought to participate in the cognitive decline and neuronal loss associated with the disease [61]. On the other hand, PLA2 has been found to be strongly overexpressed in AD, suggesting that, overall, this gene may have a negative effect by promoting the inflammatory response of the AD brain [62].

The Calcium/calmodulin-dependent protein kinase II, CaM kinase II, is the most abundant protein kinase in the brain and by controlling the trafficking of AMPAR subunits [63] a key modulator of LTP and neuronal plasticity [64]. In the context of AD it is worth noting that the CaMKII synaptic pool is reduced in cultured cortical neurons obtained from AβPP transgenic mice, a phenomenon that promotes the loss of synaptic AMPARs and the impairment of glutamatergic transmission [65]. As far as NMDA receptor subunits, IPA shows a very intriguing upregulation of two specific subunits, GRIN1 and GRIN2c, and the functional implications of such changes are discussed in the pathway section.

#### Downregulated genes of the first network

The homer family is a class of postsynaptic scaffolding proteins that regulate the structural and functional integrity of synapses [66]. Homer I is particularly important in the modulation of the trafficking of mGluRs [67] as well as in the regulation of dendritic spine formation [68]. Homer is particularly relevant in the modulation of mGluR-dependent LTD [69], [70], can also participate in the stabilization of the post synaptic density (PSD), and influences the endocytosis of NMDARs and AMPARs [66], [71].

ESR1 encodes for the estrogen receptor alpha, ER-α. ER-α is present in AD-affected regions like the hippocampus, the basal forebrain, and amygdala [72] and helps in maintaining cholinergic neurotransmission [73], [74]. A postmenopausal decrease in estrogen levels is a well-known AD co-risk factor as the hormones favor the brain catabolization of Aβ by regulating the expression of neprilysin, an enzyme that promotes Aβ degradation [75]. Moreover, ER-α polymorphisms are associated with both familial and sporadic AD [76] and decrements of the receptor mRNA splice variants have also been detected with higher frequency in AD female subjects [77]. These changes may help to explain why estrogen-replacement therapy fails to rescue cognitive functions in AD patients [78].

The transcriptional co-activator EP300 plays an important role in regulating gene expression in a number of different cell types [79] and is regulated by Presenilin 1 (PS1), an endoplasmic reticulum/Golgi transmembrane protein whose mutations have been associated with early-onset familial Alzheimer's disease ([FAD; [80]). P300 promotes a signalling mechanism that is important for long-term memory formation and neuronal survival. The regulation of p300 activity by wild-type PS1 and not by mutant PS1 indicates a partial loss of function in AD that may lead to memory loss and neurodegeneration [79].

#### Downregulated genes of the second network

CDH1 belongs to the cadherin superfamily and encodes for cadherin1, a protein that is involved in neuronal apoptosis. Depletion of cadherin 1 leads to apoptotic neuronal death by favoring the re-entry or reactivation of the cell cycle [81], an important phenomenon as aberrant cell cycle reactivation has been described in AD neurons. Finally, it must be noted that, in cortical cultured neurons, overexpression of Cdh1 results in neuroprotection against Aβ toxicity [82], [83].

Actins are key proteins involved in the maintenance of the cytoskeleton as well as in the stabilization of synapses [84]. Actin filaments can have a particular pathogenic role in AD as they are associated with apolipoprotein E, AβPP, PS1, and the tau protein [46], [85]. Actin rods have been shown in autoptic samples from AD patients. Moreover, actin is associated with phoshophorylation of the synaptic protein, cofilin, and participates in the synaptic structural changes that underlie LTP in vivo and in vitro [86]. In summary, a down expression of actin may affect synaptic efficacy and ultimately impair the molecular machinery that is responsible for learning and memory.

#### Downregulated genes of the third network

PKC can play an important role in AD as its activation promotes the non-amyloidogenic cleavage of AβPP by directly activating the α-secretase pathway or through an upstream involvement of the MAP kinases ERK1/2 [87]. PKC also inhibits the activity of the beta-site AβPP-cleaving enzyme 1, BACE-1, thereby reducing Aβ biosynthesis [88]. PKC dysfunction can also interfere with synaptic functioning. Phosphorylation of key synaptic proteins like adducin, stathmin and myristoylated alanine-rich C-kinase substrate (MARCKS) by PKC and Ca^2+^/calmodulin-dependent protein kinase II (CaMKII) can severely affect spine integrity. Interestingly, the AD brain appears to suffer from a deficit in PKC activation rather than from a defective PKC expression [89]. Furthermore, a decrease in PKC activation leads to enhanced levels of phosphorylated tau through a reduced PKC-mediated inhibition of GSK-3b [90].

The Shank family of synaptic proteins are particularly important as molecular scaffolds that control the integrity of the postsynaptic density (PSD) and handle the synaptic trafficking of glutamate receptor subunits [91]. A decrease in Shank expression can favor a dysfunctional synaptic rearrangement of NMDA and AMPA receptors and ultimately impair LTP and synaptic efficiency.

#### Downregulated genes of the fourth network

CD3 encodes for the T-cell receptor zeta, a subunit of the T-cell receptor-CD3 complex which is responsible for recognizing antigens in different intracellular signal-transduction pathways. CD3 positive T-cells induce microglial activation and actively participate in the AD development [92]. However, it must be noted that a significant decrease of CD3(+) lymphocytes can also be responsible for the general decline of immune activity that is observed in AD [78].

In the brain, the glutathione transferase (GST) promotes the cellular response that protect against oxidative injury [93]. Decreased GST activity has been found in the brain of AD patients and has been linked to AD-related neuronal death [94], [95]. Furthermore, GST polymorphisms, such as homozygous deletions in GSTM1 and GSTT1 genes, are encoding for defective proteins with less efficient enzymatic activities. These GST polymorphisms have been found with higher frequency in patients affected by late onset AD [96].

### Network analysis

Upregulated genes like ARRB1, NOS, c-JUN, and PLA2 fit in networks that are linked to neuronal apoptosis. In line with this trend, we also found a parallel downregulation of some neuroprotective and antiapoptotic genes like EP300, CDH1, and GST. Furthermore, our networks analysis indicates that Aβ-Al can promote changes of genes like APLP1, Furin, Clathrin, and IDE that favor alterations of AβPP processing and genes like CaMkII, GRIN1, and GRIN2c that modulate glutamatergic neurotransmission. We also detected changes in c-JUN and PLA2 that can influence the inflammatory response as well as an overexpression of TGFB1, MEF2, and NR41 that can promote a protective compensatory response.

Finally, we find the downregulation of key genes like SHANK 2 and actin that are involved in the maintenance of the structural and functional integrity of synapses.

### Pathway analysis

Looking at pathways, we found a IPA-inferred pathway that is related to [Ca^2+^]_i_ homeostasis (Fig. 4A). The issue of [Ca^2+^]_i_ dyshomeostasis is particularly relevant to AD. Recent findings suggest that Ca^2+^ signaling is altered in AD long before the appearance of macroscopic pathological changes [97], [98]. [Ca^2+^]_i_ rises might affect formation of AβPP and Aβ [99], [100] and promote accumulation of intraneuronal Aβ [97]. Perturbation of [Ca^2+^]_i_ levels might also interfere with neurofibrillary tangle formation [101], [102]. An altered [Ca^2+^]_i_ homeostasis in aging neurons can have important functional consequences and impair synaptic plasticity as well as learning or memory [102]. Some of the genes, like CRAC and CASQ that we found changed by Aβ-Al can also promote [Ca^2+^]_i_ deprivation. [Ca^2+^]_i_ deprivation can be deleterious too as neurons depleted of Ca^2+^ can undergo death and activate the apoptotic machinery [103], [104].

**Figure 6:**
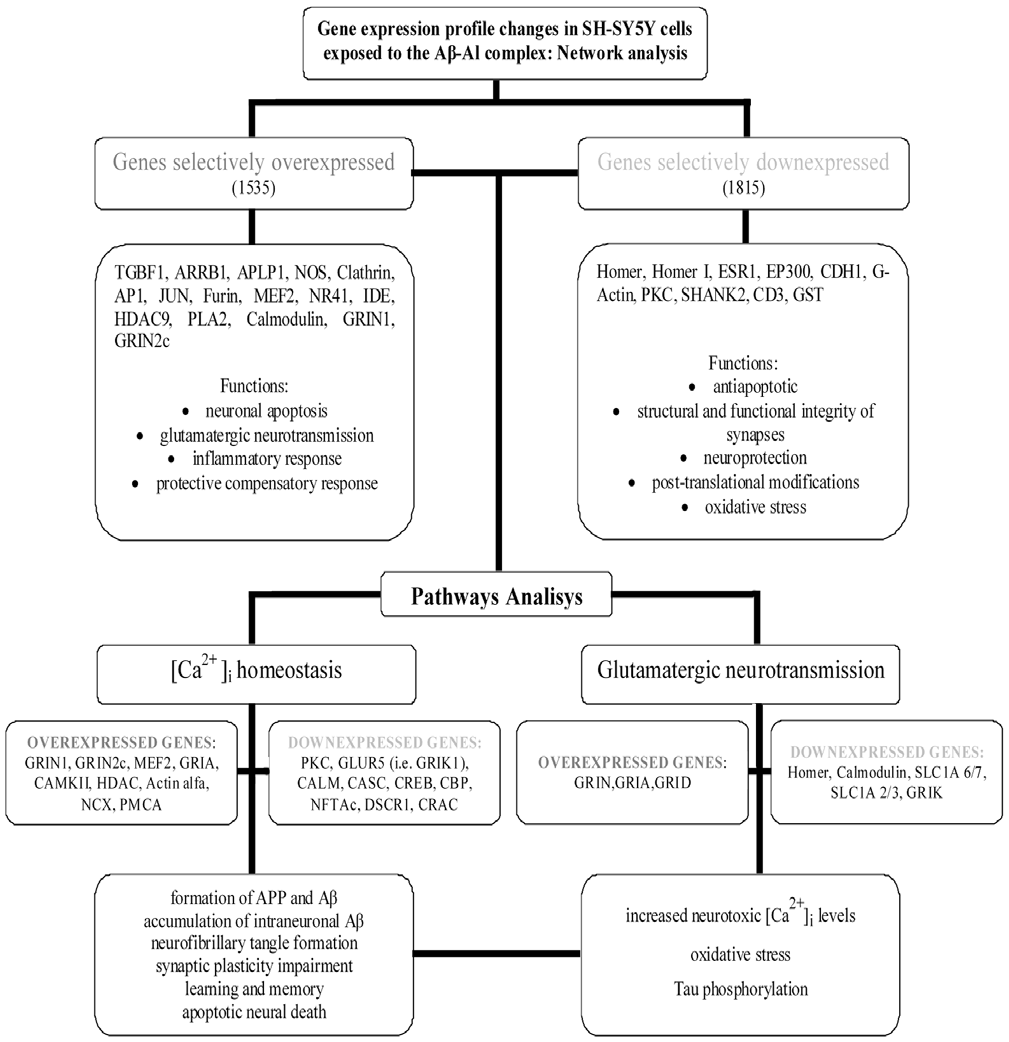
Summary of genes, biological functions, and pathways associated with gene expression changes triggered by the exposure to the Aβ-Al complex in SH-SY5Y cells.

The second pathway inferred by IPA indicates an overall increase of glutamatergic neurotransmission which is interconnected with the issue of [Ca^2+^]_i_ dyshomeostasis. Glutamate activates post-synaptic ionotropic receptors and promotes neurotoxic [Ca^2+^]_i_ rises. Interestingly, we have previously shown that Aβ-Al elicits the formation of oligomers that selectively increase NMDAR-mediated [Ca^2+^]_i_ rises [8]. Excessive [Ca^2+^]_i_ rises trigger robust oxidative stress, [101], [105] and make neurons more vulnerable to excitoxicity as free radicals inhibit glutamate re-uptake [106]. Aβ-Al can potentiate such loop, not only by increasing NMDAR-evoked currents (as well as the expression of NMDARs and AMPARs; [8]), but also by down regulating glutamate transporters that mediate glutamate re-uptake. Thus, glutamate and Aβ-Al can operate synergistically to promote excitotocic [Ca^2+^]_i_ rises and oxidative stress and set the stage for a self-perpetuating harmful loop. It must also be emphasized that oxidative stress can enhance tau phosphorylation and tau-dependent pathology which further exacerbates Aβ-driven pathology [97], [107]. In conclusion, the changes in the gene expression profile triggered by Aβ-Al lend support to the idea that this complex is largely involved in the molecular machinery that regulates neuronal as well as synaptic dysfunction and loss (summarized in Figure 6). Aβ-Al seems to modulate the expression of genes that are critical in controlling glutamatergic transmission, Ca^2+^ homeostasis as we have previously shown [108], oxidative stress, inflammation, and neuronal apoptosis. All these processes are key steps in the development of AD pathology. Our analysis can help to unravel the blueprint of the molecular determinants that are set in motion by toxic exposures to the Aβ-Al complex and offer a new perspective on how Al can play a relevant role in AD pathogenesis.

## Matherials and Methods

### Preparation of Aβ-metal complexes

1.0 mg of synthetic Aβ_1–42_ was dissolved to 1 mM in hexafluorisopropanol (HFIP) for 40 min at room temperature. After this incubation, the Aβ_1–42_ solution was separated into aliquots in microcentrifuge tubes. Hexafluorisopropanol was removed under vacuum in a Speed Vac (Sc110 Savant Instruments) and lyophilized peptide film was stored desiccated at – 20°C. Immediately prior to use, the HFIP-treated aliquots were completely re-suspended in distilled water to a concentration of 50 µM (following a modified protocol from Dahlgren et al., 2002). The Aβ-Al complex was prepared with a 24 h dialysis against a metal solution containing Al(C3H5O3)3 at T = 4°C using Spectra/PorR Float-A-LyserR tubes (Spectrum Labs) with 1000 Molecular Weight Cut Offs (MWCO). The Aβ-Al complex was then dialysed against water (three water changes) for 24 h in order to remove the excess of metal not bound to the peptide. The same treatment was also performed with Aβ alone. SH-SY5Y cells were treated with Aβ or Aβ-Al complex at 0.5 µM peptide concentration for 24 h. Aliquots of Aβ and Aβ–Al complex were taken, after dialysis, at 48 h incubation time to be analyzed by electron microscopy. Metal detection was done by atomic absorption (electrothermal atomic absorption spectrometry or flame atomic absorption spectrometry) and size exclusion chromatography. After 48 h dialysis at 4°C, many short and irregular protofibrillar structures were present in the Aβ sample as the consequence of self aggregation while few fibrils were observed. By contrast, the Aβ–Al complex was characterized by a large population of small oligomeric aggregates that as previously shown [8] possess marked neurotoxic effects. Thus, as previously described [8]. Al freezes the oligomeric state of Aβ.

### Cell cultures

SH-SY5Y human neuroblastoma cells were purchased from ECACC (European Collection of Cell Culture, Salisbury, UK). SH-SY5Y human neuroblastoma cells were maintained in Dulbecco's modified Eagle's medium (MEM): F-12 (1: 1) with L-Glutamine and 15 mM Hepes (Gibco, Carlsbad, CA USA) at 37°C with 5% CO_2_ in a humidified atmosphere (90% humidity). The medium was replaced every 2 days. Penicillin (100 units/ml; Gibco, Carlsbad, CA USA) and streptomycin (100 µg/ml; Gibco, Carlsbad, CA USA), 15% fetal bovine serum (FBS; Sigma Aldrich, St. Louis, MO), and MEM non-essential amino acids (100x; Sigma Aldrich, St. Louis, MO) were added to the medium. 0.25% Trypsin-EDTA solution and phosphate buffered saline (PBS) were obtained from Sigma Aldrich (St. Louis, MO). SHSY5Y cells were plated onto 6-well plates. The day after this plating, the culture medium was replaced with the same medium with 2% FBS containing Aβ_1–42_ or Aβ_1–42_-Al at 0.5 µM peptide concentration. The cells were incubated with different Aβ or Aβ-Al for 24 h. The cell treatment was also performed in the presence of metal (Al) at a concentration 10-fold higher than the peptide concentration.

### RNA isolation, quality control and labeling

Total RNA of neuroblastoma cells untreated or treated with various Aβ and Aβ-Al was isolated using the Qiagen RNA/DNA Mini Kit (Qiagen S.p.A., Milan, Italy) following the manufacturer's instructions. 1 µl total RNA aliquots were used for quality control by capillary electrophoresis using the RNA 6000 Nano LabChip and the Agilent Bioanalyzer 2100 (Agilent Technologies, Palo Alto, CA). All RNA samples used in this study showed no sign of degradation. 1 µg of total RNA was amplified following the Superscript™ Indirect RNA Amplification System Kit (Invitrogen™): total RNA was reverse transcribed into single-stranded complementary DNA (cDNA) using oligo(dT) primer containing a T7 promoter and Superscript III reverse transcriptase. RNase H was then added together with *E. coli* DNA polymerase I and *E. coli* DNA ligase, followed by a short incubation in order to achieve synthesis of the second-strand cDNA. The purified double-strand cDNA served as the template for the *in vitro* transcription reaction, which was carried out overnight in the presence of T7-RNA polymerase. This step generated antisense RNA (aRNA) molecules complementary to the original mRNA targets, incorporating amino-allyl UTP (aa-UTP) into the aRNA. 10 µg of purified aRNA was coupled with Alexa Fluor 555 or Alexa Fluor 647 dyes (Invitrogen, Grand Island, NY) according to the manufacturer instructions.

### Microarray

Labeled aRNA was dissolved in 90 µl of hybridization buffer, denaturated at 90°C for 3 min and applied directly to human OpArrays DNA microarray slides containing 35130 spotted oligonucleotide sequences representing 29166 different human genes (Operon Biotechnologies, Inc.). Each slide was hybridizated with treated (either Aβ, Aβ-Al, or Al) and untreated (sham) samples. Microarray hybridization was carried out in an ArrayBooster Hybridization Station (Advalytix, Brunnthal, Germany). The reaction was carried out overnight at 42°C. Posthybridization washing was performed according to the microarray slides manufacturer's instructions. Two replicates of each experiment were performed using different microarray slides, in which sample and control RNA, labeled either with Alexa Fluor 555 or Alexa Fluor 647 fluorochromes, were crossed in both combinations (dye-swapping procedure). Each experiment used material from the same cell culture and therefore represents a technical replicate. The microarrays slides were scanned using a GenePix 4000B laser scanner (Molecular Devices Corp., Sunnyvale, CA, USA) and images were processed using Genepix 6.0 software.

Microarray data are deposited in the GEO public database (accession number:GSE23000). All data are MIAME compliant.

### Statistical analysis

Statistical analysis of the microarray data was performed using Acuity 4.0 software (Molecular Devices Corp., Sunnyvale, CA, USA). Initial results were normalized using Lowess algorithm and filtered to exclude extreme outliers and flags. We also excluded any spot that had no detectable response on all slides. The log-ratios of expression were calculated as the base 2 logarithm of the ratios of background-corrected intensity medians of red dye over green dye intensities. In order to obtain a single expression value for each gene, the internal replicates (1 to 14) were plotted versus the technical replicates (6 for Aβ treatment and 2 for Al and Aβ-Al complex treatment). The X-Y scatter plot confirmed that the data were generally reproducible but we observed the presence of extreme outlier values. Then we excluded the outliers and the values of spots replicates within arrays were averaged using a trimmed mean. We excluded any spot that had no detectable response in all three treatments. Consequently, the final dataset was composed of 28676 genes. In order to identify important genes with great statistical confidence, statistical testing and fold change criteria were employed simultaneously. A gene was considered to be differentially expressed if it had an absolute value of a log-ratio higher or equal to 0.5, representing a fold-change of 1.4 in transcript quantity. Student's t-test was applied and statistical significance set at *p*<0.05.

### TaqMan real-time quantitative PCR (qRT-PCR)

A qRT-PCR analysis was performed on SH-SY5Y neuroblastoma treated with Aβ, Aβ-Al complex or Al alone to verify the gene expression profile of APLP1, APLP2, MAPT and AβPP, the genes we identified by microarray analysis. Real time quantitative PCR was carried out in a total volume of 25 µl containing 1X TaqMan Universal PCR Master mix, no AmpErase UNG and 2.5 µl of cDNA using the TaqMan assay (TAB) on ABI 7300 Sequence Detection System (ABI, Foster City, CA). Gene-specific primers and probe sets for each gene (APLP1; Hs00193069-M1) (APLP2; Hs00155778-M1) (AβPP; Hs00169098-M1) (MAPT; Hs00213491-M1) (GAPDH; Hs99999905-m1) were obtained from Assay-on-Demand Gene Expression Products (Applied Biosystems). Duplicate samples were run for each gene along with a no-template control. The housekeeping gene GAPDH was used as an internal control to normalize the expression of target genes. The real time amplifications included 10 minutes at 95°C (AmpliTaq Gold activation), followed by 40 temperature cycles for 15 seconds at 95°C and for 1 minute at 60°C. Relative expression levels were calculated for each sample after normalization against the housekeeping gene GAPDH, using the ΔΔCt method for comparing relative fold expression differences [109].

## Supporting Information

Figure S1 Graph bars show mRNA levels of APLP1 (a) and APLP2 (b) as measured by real-time PCR in SH-SY5Y cell exposed to the Aβ-Al complex, Aβ, or Al (^*^indicates p<0.0001 vs control; ^#^ indicates p<0.0001 vs Aβ and Al, n = 3). Values are expressed as means ±S.E.M.
(TIF)

Figure S2 Graph bars show mRNA levels of AβPP (a) and MAPT (b) as measured by real-time PCR in SH-SY5Y cell exposed to the Aβ-Al complex, Aβ, or Al (^*^indicates p<0.0001 vs control, ^#^ indicates p<0.0001 vs Aβ and Al, n = 3). Values are expressed as means ±S.E.M.
(TIF)

Table S1 **List of genes (1535) selectively overexpressed upon exposure to Aβ-Al compared to exposures to Aβ or Al alone.**
(DOC)

Table S2 **List of genes (1815) selectively downexpressed upon exposure to Aβ-Al compared to exposures to Aβ or Al alone.**
(DOC)

Table S3 **List of the overexpressed genes found in the first network (see** **Fig. 2A** **).**
(DOC)

Table S4 **List of the overexpressed genes found in the second network (see** **Fig. 2B** **).**
(DOC)

Table S5 **List of the overexpressed genes found in the third network (see** **Fig. 2C** **).**
(DOC)

Table S6 **List of the overexpressed genes found in the fourth network (see** **Fig. 2D** **).**
(DOC)

Table S7 **List of the downexpressed genes found in the first network (see** **Fig. 3A** **).**
(DOC)

Table S8 **List of the downexpressed genes found in the second network (see** **Fig. 3B** **).**
(DOC)

Table S9 **List of the downexpressed genes found in the third network (see** **Fig. 3C** **).**
(DOC)

Table S10 **List of the downexpressed genes found in the fourth network (see** **Fig. 3D** **).**
(DOC)

## References

[1] Lovell MA, Robertson JD, Teesdale WJ, Campbell JL, Markesbery WR (1998). Copper, iron and zinc in Alzheimer's disease senile plaques.. J Neurol Sci.

[2] Good PF, Perl DP, Bierer LM, Schmeidler J (1992). Selective accumulation of aluminum and iron in the neurofibrillary tangles of Alzheimer's disease: a laser microprobe (LAMMA) study.. Ann Neurol.

[3] Liu G, Huang W, Moir RD, Vanderburg CR, Lai B (2006). Metal exposure and Alzheimer's pathogenesis.. J Struct Biol.

[4] Sensi SL, Paoletti P, Bush AI, Sekler I (2009). Zinc in the physiology and pathology of the CNS.. Nat Rev Neurosci.

[5] House E, Collingwood J, Khan A, Korchazkina O, Berthon G (2004). Aluminium, iron, zinc and copper influence the in vitro formation of amyloid fibrils of Abeta42 in a manner which may have consequences for metal chelation therapy in Alzheimer's disease.. J Alzheimers Dis.

[6] Ricchelli F, Drago D, Filippi B, Tognon G, Zatta P (2005). Aluminum-triggered structural modifications and aggregation of beta-amyloids.. Cell Mol Life Sci.

[7] Bush AI (2003). The metallobiology of Alzheimer's disease.. Trends Neurosci.

[8] Drago D, Bettella M, Bolognin S, Cendron L, Scancar J (2008). Potential pathogenic role of beta-amyloid(1-42)-aluminum complex in Alzheimer's disease.. Int J Biochem Cell Biol.

[9] Blalock EM, Geddes JW, Chen KC, Porter NM, Markesbery WR (2004). Incipient Alzheimer's disease: microarray correlation analyses reveal major transcriptional and tumor suppressor responses.. Proc Natl Acad Sci U S A.

[10] Ricciarelli R, d'Abramo C, Massone S, Marinari U, Pronzato M (2004). Microarray analysis in Alzheimer's disease and normal aging.. IUBMB Life.

[11] Hoerndli FJ, Toigo M, Schild A, Gotz J, Day PJ (2004). Reference genes identified in SH-SY5Y cells using custom-made gene arrays with validation by quantitative polymerase chain reaction.. Anal Biochem.

[12] Alexandrov PN, Zhao Y, Pogue AI, Tarr MA, Kruck TP (2005). Synergistic effects of iron and aluminum on stress-related gene expression in primary human neural cells.. J Alzheimers Dis.

[13] Lukiw WJ, LeBlanc HJ, Carver LA, McLachlan DR, Bazan NG (1998). Run-on gene transcription in human neocortical nuclei. Inhibition by nanomolar aluminum and implications for neurodegenerative disease.. J Mol Neurosci.

[14] Lukiw WJ, Percy ME, Kruck TP (2005). Nanomolar aluminum induces pro-inflammatory and pro-apoptotic gene expression in human brain cells in primary culture.. J Inorg Biochem.

[15] Buckingham SD, Jones AK, Brown LA, Sattelle DB (2009). Nicotinic acetylcholine receptor signalling: roles in Alzheimer's disease and amyloid neuroprotection.. Pharmacol Rev.

[16] Bataille N, Schmitt N, Aumercier-Maes P, Ollivier B, Lucas-Heron B (1994). Molecular cloning of human calmitine, a mitochondrial calcium binding protein, reveals identity with calsequestrine.. Biochem Biophys Res Commun.

[17] Wu HY, Hudry E, Hashimoto T, Kuchibhotla K, Rozkalne A (2010). Amyloid beta induces the morphological neurodegenerative triad of spine loss, dendritic simplification, and neuritic dystrophies through calcineurin activation.. J Neurosci.

[18] Malleret G, Haditsch U, Genoux D, Jones MW, Bliss TV (2001). Inducible and reversible enhancement of learning, memory, and long-term potentiation by genetic inhibition of calcineurin.. Cell.

[19] Putney JW (1986). A model for receptor-regulated calcium entry.. Cell Calcium.

[20] Wyss-Coray T, Yan F, Lin AH, Lambris JD, Alexander JJ (2002). Prominent neurodegeneration and increased plaque formation in complement-inhibited Alzheimer's mice.. Proc Natl Acad Sci U S A.

[21] Xiao K, McClatchy DB, Shukla AK, Zhao Y, Chen M (2007). Functional specialization of beta-arrestin interactions revealed by proteomic analysis.. Proc Natl Acad Sci U S A.

[22] Gerber U, Gee CE, Benquet P (2007). Metabotropic glutamate receptors: intracellular signaling pathways.. Curr Opin Pharmacol.

[23] Thinakaran G, Koo EH (2008). Amyloid precursor protein trafficking, processing, and function.. J Biol Chem.

[24] Bayer TA, Paliga K, Weggen S, Wiestler OD, Beyreuther K (1997). Amyloid precursor-like protein 1 accumulates in neuritic plaques in Alzheimer's disease.. Acta Neuropathol.

[25] Neumann S, Schobel S, Jager S, Trautwein A, Haass C (2006). Amyloid precursor-like protein 1 influences endocytosis and proteolytic processing of the amyloid precursor protein.. J Biol Chem.

[26] Adlerz L, Holback S, Multhaup G, Iverfeldt K (2007). IGF-1-induced processing of the amyloid precursor protein family is mediated by different signaling pathways.. J Biol Chem.

[27] Querfurth HW, LaFerla FM (2010). Alzheimer's disease.. N Engl J Med.

[28] Aliev G, Palacios HH, Lipsitt AE, Fischbach K, Lamb BT (2009). Nitric oxide as an initiator of brain lesions during the development of Alzheimer disease.. Neurotox Res.

[29] Cuello AC, Ferretti MT, Leon WC, Iulita MF, Melis T (2010). Early-stage inflammation and experimental therapy in transgenic models of the Alzheimer-like amyloid pathology.. Neurodegener Dis.

[30] Lipton SA (2007). Pathologically-activated therapeutics for neuroprotection: mechanism of NMDA receptor block by memantine and S-nitrosylation.. Curr Drug Targets.

[31] Sattler R, Xiong Z, Lu WY, Hafner M, MacDonald JF (1999). Specific coupling of NMDA receptor activation to nitric oxide neurotoxicity by PSD-95 protein.. Science.

[32] Wu F, Yao PJ (2009). Clathrin-mediated endocytosis and Alzheimer's disease: an update.. Ageing Res Rev.

[33] Kopec CD, Li B, Wei W, Boehm J, Malinow R (2006). Glutamate receptor exocytosis and spine enlargement during chemically induced long-term potentiation.. J Neurosci.

[34] Cirrito JR, Yamada KA, Finn MB, Sloviter RS, Bales KR (2005). Synaptic activity regulates interstitial fluid amyloid-beta levels in vivo.. Neuron.

[35] Vukic V, Callaghan D, Walker D, Lue LF, Liu QY (2009). Expression of inflammatory genes induced by beta-amyloid peptides in human brain endothelial cells and in Alzheimer's brain is mediated by the JNK-AP1 signaling pathway.. Neurobiol Dis.

[36] Borsello T, Forloni G (2007). JNK signalling: a possible target to prevent neurodegeneration.. Curr Pharm Des.

[37] Chang KA, Kim HS, Ha TY, Ha JW, Shin KY (2006). Phosphorylation of amyloid precursor protein (APP) at Thr668 regulates the nuclear translocation of the APP intracellular domain and induces neurodegeneration.. Mol Cell Biol.

[38] Thomas G (2002). Furin at the cutting edge: from protein traffic to embryogenesis and disease.. Nat Rev Mol Cell Biol.

[39] Buxbaum JD, Liu KN, Luo Y, Slack JL, Stocking KL (1998). Evidence that tumor necrosis factor alpha converting enzyme is involved in regulated alpha-secretase cleavage of the Alzheimer amyloid protein precursor.. J Biol Chem.

[40] Fahrenholz F, Gilbert S, Kojro E, Lammich S, Postina R (2000). Alpha-secretase activity of the disintegrin metalloprotease ADAM 10. Influences of domain structure.. Ann N Y Acad Sci.

[41] Hwang EM, Kim SK, Sohn JH, Lee JY, Kim Y (2006). Furin is an endogenous regulator of alpha-secretase associated APP processing.. Biochem Biophys Res Commun.

[42] Mao Z, Bonni A, Xia F, Nadal-Vicens M, Greenberg ME (1999). Neuronal activity-dependent cell survival mediated by transcription factor MEF2.. Science.

[43] Flavell SW, Cowan CW, Kim TK, Greer PL, Lin Y (2006). Activity-dependent regulation of MEF2 transcription factors suppresses excitatory synapse number.. Science.

[44] Wang Y, Liu L, Xia Z (2007). Brain-derived neurotrophic factor stimulates the transcriptional and neuroprotective activity of myocyte-enhancer factor 2C through an ERK1/2-RSK2 signaling cascade.. J Neurochem.

[45] Barbosa AC, Kim MS, Ertunc M, Adachi M, Nelson ED (2008). MEF2C, a transcription factor that facilitates learning and memory by negative regulation of synapse numbers and function.. Proc Natl Acad Sci U S A.

[46] Zhang SJ, Zou M, Lu L, Lau D, Ditzel DA (2009). Nuclear calcium signaling controls expression of a large gene pool: identification of a gene program for acquired neuroprotection induced by synaptic activity.. PLoS Genet.

[47] Kurochkin IV, Goto S (1994). Alzheimer's beta-amyloid peptide specifically interacts with and is degraded by insulin degrading enzyme.. FEBS Lett.

[48] Farris W, Mansourian S, Chang Y, Lindsley L, Eckman EA (2003). Insulin-degrading enzyme regulates the levels of insulin, amyloid beta-protein, and the beta-amyloid precursor protein intracellular domain in vivo.. Proc Natl Acad Sci U S A.

[49] Bertram L, Blacker D, Mullin K, Keeney D, Jones J (2000). Evidence for genetic linkage of Alzheimer's disease to chromosome 10q.. Science.

[50] Carrasquillo MM, Belbin O, Hunter TA, Ma L, Bisceglio GD (2010). Replication of CLU, CR1, and PICALM Associations With Alzheimer Disease.. Arch Neurol.

[51] Carrasquillo MM, Belbin O, Zou F, Allen M, Ertekin-Taner N (2010). Concordant association of insulin degrading enzyme gene (IDE) variants with IDE mRNA, Abeta, and Alzheimer's disease.. PLoS One.

[52] Miners JS, Baig S, Tayler H, Kehoe PG, Love S (2009). Neprilysin and insulin-degrading enzyme levels are increased in Alzheimer disease in relation to disease severity.. J Neuropathol Exp Neurol.

[53] Morrison BE, Majdzadeh N, D'Mello SR (2007). Histone deacetylases: focus on the nervous system.. Cell Mol Life Sci.

[54] Alberini CM (2009). Transcription factors in long-term memory and synaptic plasticity.. Physiol Rev.

[55] Chuang DM, Leng Y, Marinova Z, Kim HJ, Chiu CT (2009). Multiple roles of HDAC inhibition in neurodegenerative conditions.. Trends Neurosci.

[56] Peleg S, Sananbenesi F, Zovoilis A, Burkhardt S, Bahari-Javan S (2010). Altered histone acetylation is associated with age-dependent memory impairment in mice.. Science.

[57] Morrison BE, Majdzadeh N, Zhang X, Lyles A, Bassel-Duby R (2006). Neuroprotection by histone deacetylase-related protein.. Mol Cell Biol.

[58] Dennis EA (1994). Diversity of group types, regulation, and function of phospholipase A2.. J Biol Chem.

[59] Balsinde J, Shinohara H, Lefkowitz LJ, Johnson CA, Balboa MA (1999). Group V phospholipase A(2)-dependent induction of cyclooxygenase-2 in macrophages.. J Biol Chem.

[60] Sun GY, Xu J, Jensen MD, Simonyi A (2004). Phospholipase A2 in the central nervous system: implications for neurodegenerative diseases.. J Lipid Res.

[61] Schaeffer EL, Forlenza OV, Gattaz WF (2009). Phospholipase A2 activation as a therapeutic approach for cognitive enhancement in early-stage Alzheimer disease.. Psychopharmacology (Berl).

[62] Bazan NG, Colangelo V, Lukiw WJ (2002). Prostaglandins and other lipid mediators in Alzheimer's disease.. Prostaglandins Other Lipid Mediat.

[63] Hayashi Y, Shi SH, Esteban JA, Piccini A, Poncer JC (2000). Driving AMPA receptors into synapses by LTP and CaMKII: requirement for GluR1 and PDZ domain interaction.. Science.

[64] Derkach VA, Oh MC, Guire ES, Soderling TR (2007). Regulatory mechanisms of AMPA receptors in synaptic plasticity.. Nat Rev Neurosci.

[65] Gu Z, Liu W, Yan Z (2009). {beta}-Amyloid impairs AMPA receptor trafficking and function by reducing Ca2+/calmodulin-dependent protein kinase II synaptic distribution.. J Biol Chem.

[66] Roselli F, Hutzler P, Wegerich Y, Livrea P, Almeida OF (2009). Disassembly of shank and homer synaptic clusters is driven by soluble beta-amyloid(1-40) through divergent NMDAR-dependent signalling pathways.. PLoS One.

[67] Mao L, Yang L, Tang Q, Samdani S, Zhang G (2005). The scaffold protein Homer1b/c links metabotropic glutamate receptor 5 to extracellular signal-regulated protein kinase cascades in neurons.. J Neurosci.

[68] Sala C, Piech V, Wilson NR, Passafaro M, Liu G (2001). Regulation of dendritic spine morphology and synaptic function by Shank and Homer.. Neuron.

[69] Ronesi JA, Huber KM (2008). Homer interactions are necessary for metabotropic glutamate receptor-induced long-term depression and translational activation.. J Neurosci.

[70] Aronica E, Dickson DW, Kress Y, Morrison JH, Zukin RS (1998). Non-plaque dystrophic dendrites in Alzheimer hippocampus: a new pathological structure revealed by glutamate receptor immunocytochemistry.. Neuroscience.

[71] Roselli F, Tirard M, Lu J, Hutzler P, Lamberti P (2005). Soluble beta-amyloid1-40 induces NMDA-dependent degradation of postsynaptic density-95 at glutamatergic synapses.. J Neurosci.

[72] McEwen BS (2001). Invited review: Estrogens effects on the brain: multiple sites and molecular mechanisms.. J Appl Physiol.

[73] Savaskan E, Olivieri G, Meier F, Ravid R, Muller-Spahn F (2001). Hippocampal estrogen beta-receptor immunoreactivity is increased in Alzheimer's disease.. Brain Res.

[74] Schupf N, Lee JH, Wei M, Pang D, Chace C (2008). Estrogen receptor-alpha variants increase risk of Alzheimer's disease in women with Down syndrome.. Dement Geriatr Cogn Disord.

[75] Liang K, Yang L, Yin C, Xiao Z, Zhang J (2010). Estrogen stimulates degradation of beta-amyloid peptide by up-regulating neprilysin.. J Biol Chem.

[76] Porrello E, Monti MC, Sinforiani E, Cairati M, Guaita A (2006). Estrogen receptor alpha and APOEepsilon4 polymorphisms interact to increase risk for sporadic AD in Italian females.. Eur J Neurol.

[77] Ishunina TA, Swaab DF (2010). Decreased alternative splicing of estrogen receptor-alpha mRNA in the Alzheimer's disease brain.. Neurobiol Aging.

[78] Richartz-Salzburger E, Batra A, Stransky E, Laske C, Kohler N (2007). Altered lymphocyte distribution in Alzheimer's disease.. J Psychiatr Res.

[79] Francis YI, Diss JK, Kariti M, Stephanou A, Latchman DS (2007). p300 activation by Presenilin 1 but not by its M146L mutant.. Neurosci Lett.

[80] Weihl CC, Ghadge GD, Miller RJ, Roos RP (1999). Processing of wild-type and mutant familial Alzheimer's disease-associated presenilin-1 in cultured neurons.. J Neurochem.

[81] Becker EB, Bonni A (2004). Cell cycle regulation of neuronal apoptosis in development and disease.. Prog Neurobiol.

[82] Almeida A, Bolanos JP, Moreno S (2005). Cdh1/Hct1-APC is essential for the survival of postmitotic neurons.. J Neurosci.

[83] Aulia S, Tang BL (2006). Cdh1-APC/C, cyclin B-Cdc2, and Alzheimer's disease pathology.. Biochem Biophys Res Commun.

[84] Citri A, Soler-Llavina G, Bhattacharyya S, Malenka RC (2009). N-methyl-D-aspartate receptor- and metabotropic glutamate receptor-dependent long-term depression are differentially regulated by the ubiquitin-proteasome system.. Eur J Neurosci.

[85] Bamburg JR, Bloom GS (2009). Cytoskeletal pathologies of Alzheimer disease.. Cell Motil Cytoskeleton.

[86] Fedulov V, Rex CS, Simmons DA, Palmer L, Gall CM (2007). Evidence that long-term potentiation occurs within individual hippocampal synapses during learning.. J Neurosci.

[87] Alkon DL, Sun MK, Nelson TJ (2007). PKC signaling deficits: a mechanistic hypothesis for the origins of Alzheimer's disease.. Trends Pharmacol Sci.

[88] Fu H, Dou J, Li W, Cui W, Mak S (2009). Promising multifunctional anti-Alzheimer's dimer bis(7)-Cognitin acting as an activator of protein kinase C regulates activities of alpha-secretase and BACE-1 concurrently.. Eur J Pharmacol.

[89] Pascale A, Amadio M, Govoni S, Battaini F (2007). The aging brain, a key target for the future: the protein kinase C involvement.. Pharmacol Res.

[90] Wang ZF, Li HL, Li XC, Zhang Q, Tian Q (2006). Effects of endogenous beta-amyloid overproduction on tau phosphorylation in cell culture.. J Neurochem.

[91] Sheng M, Kim E (2000). The Shank family of scaffold proteins.. J Cell Sci.

[92] Stalder M, Phinney A, Probst A, Sommer B, Staufenbiel M (1999). Association of microglia with amyloid plaques in brains of APP23 transgenic mice.. Am J Pathol.

[93] Hayes JD, Strange RC (2000). Glutathione S-transferase polymorphisms and their biological consequences.. Pharmacology.

[94] Lovell MA, Xie C, Markesbery WR (1998). Decreased glutathione transferase activity in brain and ventricular fluid in Alzheimer's disease.. Neurology.

[95] Bernardini S, Bellincampi L, Ballerini S, Federici G, Iori R (2005). Glutathione S-transferase P1 *C allelic variant increases susceptibility for late-onset Alzheimer disease: association study and relationship with apolipoprotein E epsilon4 allele.. Clin Chem.

[96] Spalletta G, Bernardini S, Bellincampi L, Federici G, Trequattrini A (2007). Glutathione S-transferase P1 and T1 gene polymorphisms predict longitudinal course and age at onset of Alzheimer disease.. Am J Geriatr Psychiatry.

[97] LaFerla FM, Green KN, Oddo S (2007). Intracellular amyloid-beta in Alzheimer's disease.. Nat Rev Neurosci.

[98] Guo Q, Fu W, Holtsberg FW, Steiner SM, Mattson MP (1999). Superoxide mediates the cell-death-enhancing action of presenilin-1 mutations.. J Neurosci Res.

[99] Querfurth HW, Selkoe DJ (1994). Calcium ionophore increases amyloid beta peptide production by cultured cells.. Biochemistry.

[100] Querfurth HW, Jiang J, Geiger JD, Selkoe DJ (1997). Caffeine stimulates amyloid beta-peptide release from beta-amyloid precursor protein-transfected HEK293 cells.. J Neurochem.

[101] Green KN, LaFerla FM (2008). Linking calcium to Abeta and Alzheimer's disease.. Neuron.

[102] Foster TC (2007). Calcium homeostasis and modulation of synaptic plasticity in the aged brain.. Aging Cell.

[103] Johnson EM, Koike T, Franklin J (1992). A “calcium set-point hypothesis” of neuronal dependence on neurotrophic factor.. Exp Neurol.

[104] Canzoniero LM, Babcock DJ, Gottron FJ, Grabb MC, Manzerra P (2004). Raising intracellular calcium attenuates neuronal apoptosis triggered by staurosporine or oxygen-glucose deprivation in the presence of glutamate receptor blockade.. Neurobiol Dis.

[105] Mattson MP, Chan SL (2003). Neuronal and glial calcium signaling in Alzheimer's disease.. Cell Calcium.

[106] Trotti D, Danbolt NC, Volterra A (1998). Glutamate transporters are oxidant-vulnerable: a molecular link between oxidative and excitotoxic neurodegeneration?. Trends Pharmacol Sci.

[107] Melov S, Adlard PA, Morten K, Johnson F, Golden TR (2007). Mitochondrial oxidative stress causes hyperphosphorylation of tau.. PLoS One.

[108] Drago D, Cavaliere A, Mascetra N, Ciavardelli D, di Ilio C (2008). Aluminum modulates effects of beta amyloid(1-42) on neuronal calcium homeostasis and mitochondria functioning and is altered in a triple transgenic mouse model of Alzheimer's disease.. Rejuvenation Res.

[109] Livak KJ, Schmittgen TD (2001). Analysis of relative gene expression data using real-time quantitative PCR and the 2(-Delta Delta C(T)) Method.. Methods.

