# Supplementary figures and images for: Microarray Analysis on Human Neuroblastoma Cells Exposed to Aluminum, β1–42-Amyloid or the β1–42-Amyloid Aluminum Complex
Source: PLoS One. 2011 Jan 27;6(1):e15965. doi: 10.1371/journal.pone.0015965 (PMC3029275; doi:10.1371/journal.pone.0015965)

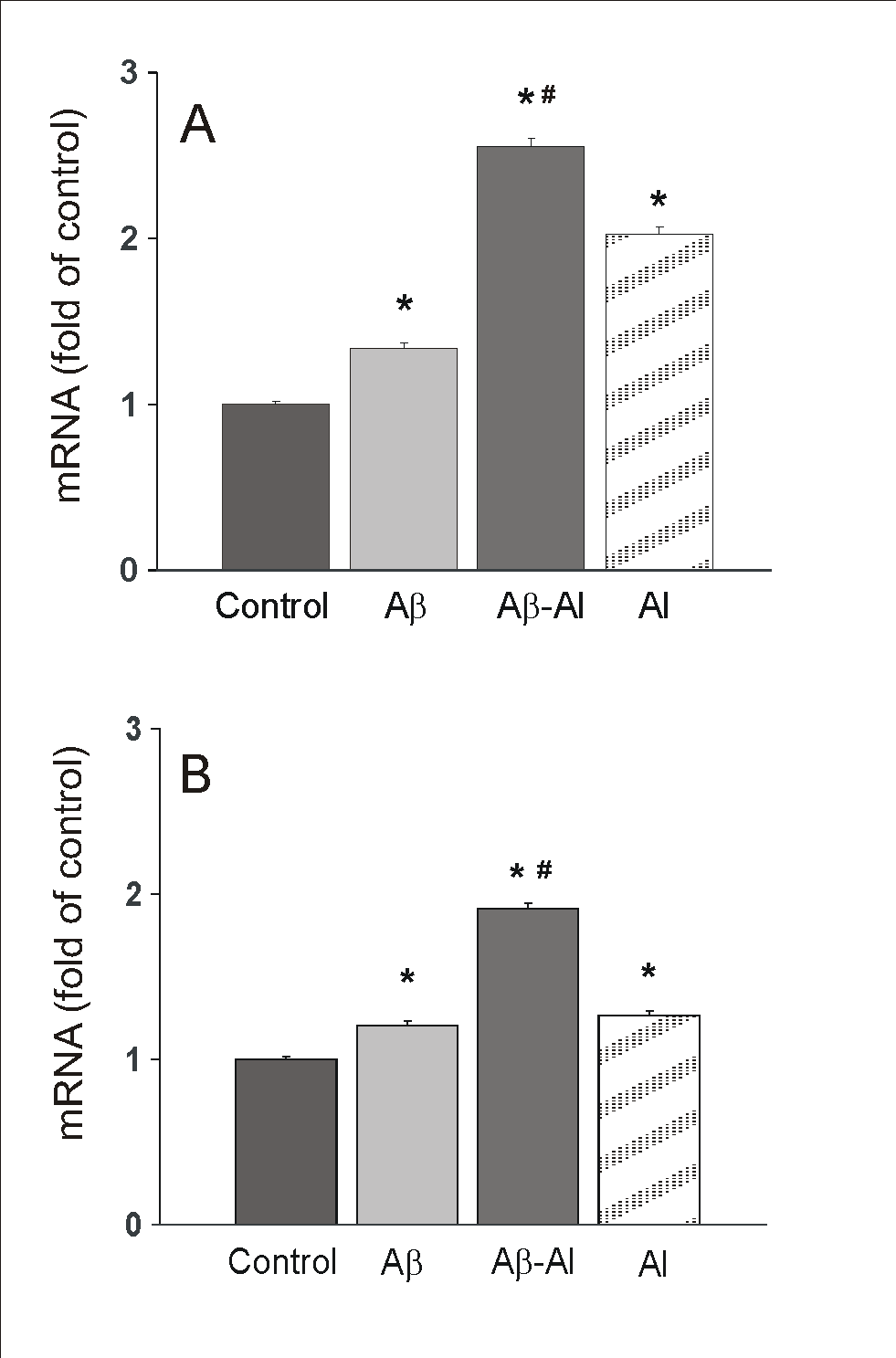

Supplement: Figure S1 — Graph bars show mRNA levels of APLP1 (a) and APLP2 (b) as measured by real-time PCR in SH-SY5Y cell exposed to the Aβ-Al complex, Aβ, or Al (*indicates p<0.0001 vs control; # indicates p<0.0001 vs Aβ and Al, n = 3). Values are expressed as means ±S.E.M. (TIF) [file pone.0015965.s001.tif]

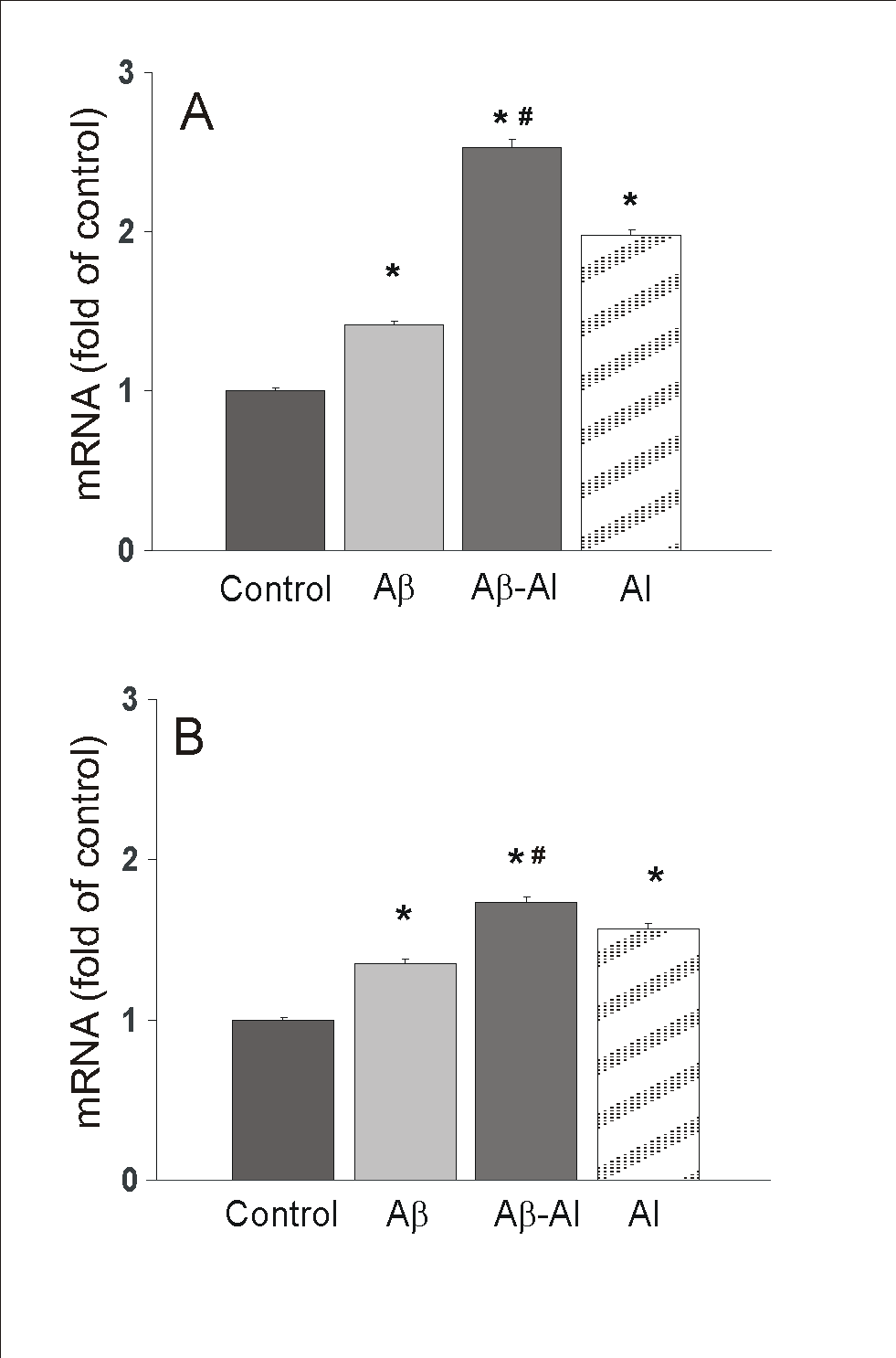

Supplement: Figure S2 — Graph bars show mRNA levels of AβPP (a) and MAPT (b) as measured by real-time PCR in SH-SY5Y cell exposed to the Aβ-Al complex, Aβ, or Al (*indicates p<0.0001 vs control, # indicates p<0.0001 vs Aβ and Al, n = 3). Values are expressed as means ±S.E.M. (TIF) [file pone.0015965.s002.tif]
