# Supplementary material for: Microarray Analysis on Human Neuroblastoma Cells Exposed to Aluminum, β1–42-Amyloid or the β1–42-Amyloid Aluminum Complex
Source: PLoS One. 2011 Jan 27;6(1):e15965. doi: 10.1371/journal.pone.0015965 (PMC3029275; doi:10.1371/journal.pone.0015965)
Supplement: Table S1 — List of genes (1535) selectively overexpressed upon exposure to Aβ-Al compared to exposures to Aβ or Al alone. (DOC) [file pone.0015965.s003.doc]

| Gene  Symbol | AB  (Log2 Ratio ) | ABAL  (Log2 Ratio) | AL  (Log2 Ratio) | GB accession | Description RefSeq |
| --- | --- | --- | --- | --- | --- |
| - | 0.32 | 4.77 | -0.42 | AK057935 | - |
| - | -0.34 | 4.74 | 0.09 | - | similar to Hypothetical protein DJ845O24.1 |
| IL1F9 | -0.50 | 4.00 | -0.85 | AY359111,AY359111 | interleukin 1 family, member 9 |
| - | 0.20 | 3.81 | 0.00 | AK091571 | - |
| - | -0.24 | 3.80 | -0.13 | - | hypothetical LOC392621 |
| - | 0.31 | 3.73 | -0.12 | - | similar to solute carrier family 22 member 3; organic cation transporter 3; extraneuronal monoamine transporter; EMT organic cation transporter 3 |
| Q9BU53_HUMAN | 0.05 | 3.58 | -0.10 | BQ230436,BC002886 | hypothetical gene supported by AK131029; BC002886 |
| - | -0.35 | 3.56 | 0.29 | AK055821 | - |
| - | -0.68 | 3.56 | 0.48 | AK056155 | - |
| BTLA | 0.13 | 3.52 | 0.35 | - | B and T lymphocyte associated |
| - | -0.14 | 3.47 | -0.30 | - | - |
| - | 0.49 | 3.41 | 0.29 | AK056211 | - |
| NEFL | -0.55 | 3.39 | -0.16 | AK075003 | neurofilament, light polypeptide 68kDa |
| - | -0.74 | 3.37 | 0.25 | - | - |
| - | 0.00 | 3.37 | 0.41 | AK058134 | - |
| - | -0.51 | 3.31 | 0.31 | - | similar to 33 kDa protein |
| NP_079184.1 | -0.09 | 3.12 | 0.27 | BC051855 | hypothetical protein FLJ12973 |
| C20orf29 | -0.06 | 3.12 | 0.43 | AK002030 | chromosome 20 open reading frame 29 |
| AKAP10 | 0.05 | 3.11 | -1.18 | AF037439,BC017055 | A kinase (PRKA) anchor protein 10 |
| TMPRSS7 | 0.17 | 3.10 | 0.18 | AK131211 | transmembrane serine protease 7 |
| IL20 | 0.48 | 3.03 | -0.39 | AF224266 | interleukin 20 |
| - | -0.42 | 2.99 | -0.12 | AK094936 | hypothetical gene supported by AK094936 |
| - | -0.19 | 2.88 | 0.27 | BQ072415 | hypothetical LOC389415 |
| - | -0.46 | 2.84 | 0.48 | AK098244 | - |
| - | 0.26 | 2.83 | -0.59 | - | - |
| - | 0.03 | 2.79 | -0.06 | - | - |
| - | -0.19 | 2.78 | 0.36 | AK057987 | - |
| - | -0.20 | 2.77 | 0.32 | AK091302 | - |
| - | 0.00 | 2.75 | -0.15 | AK127863 | FLJ45966 protein |
| - | -0.23 | 2.68 | -0.24 | - | hypothetical LOC388630 |
| - | -0.01 | 2.67 | -0.11 | AK055550 | - |
| NP_001005482.1 | -0.15 | 2.67 | -0.13 | - | olfactory receptor, family 5, subfamily H, member 2 |
| CCIN | -0.55 | 2.61 | -0.76 | Z46967,AF333334 | calicin |
| Q8N8L1_HUMAN | 0.37 | 2.61 | 0.44 | AK098403 | - |
| NP_653249.1 | 0.29 | 2.58 | 0.16 | AK057348 | hypothetical protein FLJ32786 |
| - | 0.43 | 2.58 | 0.10 | - | similar to Polyadenylate-binding protein 4 (Poly(A)-binding protein 4) (PABP 4) (Inducible poly(A)-binding protein) (iPABP) (Activated-platelet protein-1) (APP-1) |
| SFTA2_HUMAN | 0.13 | 2.55 | -0.60 | - | surfactant, pulmonary-associated protein A2 |
| - | -0.31 | 2.55 | -0.03 | AK090612 | - |
| - | -0.33 | 2.54 | 0.46 | - | hypothetical LOC339583 |
| C16orf3 | 0.48 | 2.51 | -0.31 | AF050081 | chromosome 16 open reading frame 3 |
| PRRX2 | -0.07 | 2.51 | -0.27 | BC014645 | paired related homeobox 2 |
| - | -0.17 | 2.39 | -1.45 | AK095831 | - |
| - | 0.10 | 2.37 | 0.22 | - | olfactory receptor, family 2, subfamily L, member 2 |
| - | -0.15 | 2.36 | 0.44 | AK055561 | endoplasmic reticulum to nucleus signalling 1 |
| AOC2 | 0.40 | 2.35 | 0.12 | AF081363 | amine oxidase, copper containing 2 (retina-specific) |
| CACH1_HUMAN | -0.44 | 2.35 | 0.03 | AK122960 | cytosolic acetyl-CoA hydrolase |
| XP_293924.1 | -1.23 | 2.32 | -0.32 | BX648504 | similar to RIKEN cDNA 4732495G21 gene |
| - | 0.00 | 2.32 | -0.04 | AK057720 | - |
| - | 0.30 | 2.29 | 0.44 | AK024248 | hypothetical LOC389221 |
| KIAA0367 | 0.08 | 2.28 | -0.21 | AL834213,AB002365 | KIAA0367 |
| - | 0.48 | 2.28 | -0.12 | - | - |
| - | -0.22 | 2.25 | 0.04 | - | similar to ribosomal protein S19; 40S ribosomal protein S19 |
| - | 0.11 | 2.25 | -0.02 | AF000997 | X Kell blood group precursor-related, Y-linked 2 |
| OR6Y1 | -0.59 | 2.25 | 0.01 | - | olfactory receptor, family 6, subfamily Y, member 1 |
| TEP1 | -1.18 | 2.25 | 0.18 | U86136 | telomerase-associated protein 1 |
| NP_001007537.1 | 0.24 | 2.24 | -0.53 | AK131335 | aldo-keto reductase family 1, member C-like 1 |
| - | 0.04 | 2.23 | -0.41 | AK054601 | - |
| NP_001008494.1 | 0.41 | 2.22 | -1.04 | CR749372,AK025181 | hypothetical protein LOC91464 |
| FOLR2 | -0.10 | 2.22 | -0.22 | AF000380 | folate receptor 2 (fetal) |
| - | 0.07 | 2.21 | 0.36 | - | - |
| BDKRB2 | -0.09 | 2.18 | 0.03 | CR749338 | bradykinin receptor B2 |
| - | -0.32 | 2.17 | 0.49 | AK091108 | - |
| - | 0.06 | 2.17 | 0.38 | AK094559 | - |
| HAS2 | -0.18 | 2.17 | 0.02 | U54804 | hyaluronan synthase 2 |
| - | 0.46 | 2.16 | -0.54 | AB067493 | - |
| KIAA1409 | 0.46 | 2.15 | -0.03 | AB037830 | KIAA1409 |
| - | 0.42 | 2.14 | 0.34 | - | hypothetical LOC388900 |
| - | 0.03 | 2.13 | 0.21 | AK027227 | - |
| CHST4 | -0.16 | 2.11 | 0.18 | BC035282 | carbohydrate (N-acetylglucosamine 6-O) sulfotransferase 4 |
| - | 0.33 | 2.11 | 0.10 | AL110155 | - |
| PRM3 | 0.43 | 2.11 | 0.29 | - | protamine 3 |
| FURIN | -0.34 | 2.09 | -0.41 | X17094 | furin (paired basic amino acid cleaving enzyme) |
| RFX2 | -0.05 | 2.09 | -0.11 | X76091 | regulatory factor X, 2 (influences HLA class II expression) |
| - | -0.31 | 2.09 | -0.24 | - | - |
| - | 0.00 | 2.08 | 0.15 | - | similar to immune associated nucleotide 4 like 1; immunity associated protein 3 |
| - | -0.01 | 2.07 | 0.19 | AK056829 | - |
| SPOCD1 | 0.17 | 2.07 | 0.28 | BC007910 | - |
| DUSP7 | -1.14 | 2.07 | -0.61 | X93921 | dual specificity phosphatase 7 |
| SLC16A2 | 0.27 | 2.07 | -0.01 | U05315 | solute carrier family 16 (monocarboxylic acid transporters), member 2 |
| - | 0.27 | 2.06 | 0.34 | - | similar to alpha tubulin |
| - | -0.50 | 2.06 | -0.01 | AK097819 | - |
| NP_942123.1 | -0.19 | 2.04 | 0.03 | - | similar to microtubule associated testis specific serine/threonine protein kinase |
| - | 0.00 | 2.03 | -0.06 | AK056683 | - |
| NP_689553.1 | -0.10 | 2.03 | -0.44 | - | hypothetical protein FLJ39075 |
| NP_055044.1 | 0.29 | 2.02 | 0.25 | S75989 | solute carrier family 6 (neurotransmitter transporter, GABA), member 11 |
| - | 0.06 | 2.01 | -1.01 | - | hypothetical LOC400572 |
| - | 0.35 | 1.99 | 0.27 | - | similar to hypothetical protein FLJ36144 |
| XP_085463.3 | -0.10 | 1.99 | -0.86 | AL833749,BC015446 | hypothetical LOC146439 |
| - | -0.01 | 1.98 | -0.93 | - | similar to glycine cleavage system protein H (aminomethyl carrier) |
| NP_853631.1 | -0.33 | 1.98 | 0.42 | - | keratin associated protein 13-4 |
| Q63ZE4_HUMAN | -0.56 | 1.96 | 0.27 | - | similar to organic anion transporter 6 |
| C6orf15 | -0.40 | 1.95 | 0.27 | AY358438,AB031481 | chromosome 6 open reading frame 15 |
| XP_173132.2 | -0.31 | 1.95 | 0.04 | - | hypothetical gene supported by NM_030930; AY007125 |
| - | -0.60 | 1.95 | 0.44 | - | similar to extensin-like protein |
| GMPPA | -0.25 | 1.94 | -0.12 | BC007456,AK000999 | GDP-mannose pyrophosphorylase A |
| AR | -0.15 | 1.94 | 0.42 | M23263 | androgen receptor (dihydrotestosterone receptor; testicular feminization; spinal and bulbar muscular atrophy; Kennedy disease) |
| - | -0.23 | 1.94 | -0.36 | AK095281, | LOC440422 |
| IMPG1 | -0.10 | 1.94 | -0.30 | CR749572 | interphotoreceptor matrix proteoglycan 1 |
| NP_853655.1 | 0.00 | 1.93 | 0.30 | - | keratin associated protein 23-1 |
| P2RXL1 | -0.01 | 1.93 | -0.01 | AB002058 | purinergic receptor P2X-like 1, orphan receptor |
| C7orf3 | 0.23 | 1.93 | 0.36 | AF107455 | chromosome 7 open reading frame 3 |
| - | -0.02 | 1.93 | -1.04 | - | similar to ankyrin repeat domain 20A |
| LPPD_HUMAN | -1.56 | 1.92 | 0.33 | AW966174 | secretoglobin family 1D member 4 |
| - | -0.08 | 1.92 | -0.25 | - | hypothetical LOC285248 |
| EDG1 | 0.28 | 1.92 | -1.10 | M31210,BC018650 | endothelial differentiation, sphingolipid G-protein-coupled receptor, 1 |
| - | -0.16 | 1.90 | 0.49 | - | hypothetical protein LOC255167 |
| - | 0.28 | 1.90 | 0.15 | - | hypothetical LOC389657 |
| - | 0.33 | 1.90 | -0.03 | AK026869 | - |
| PSG4 | -0.68 | 1.90 | -0.81 | BC063127 | pregnancy specific beta-1-glycoprotein 4 |
| CENTA2 | -0.15 | 1.90 | -0.36 | AJ272195 | centaurin, alpha 2 |
| Q8NEG1_HUMAN | 0.46 | 1.87 | 0.25 | - | - |
| - | 0.36 | 1.87 | 0.46 | AK094300 | - |
| NOS2A | -0.21 | 1.87 | -0.14 | X73029 | nitric oxide synthase 2A (inducible, hepatocytes) |
| - | 0.00 | 1.86 | 0.45 | - | - |
| GGA1 | -0.02 | 1.86 | -0.27 | AF190862, | golgi associated, gamma adaptin ear containing, ARF binding protein 1 |
| - | -1.11 | 1.86 | 0.14 | - | hypothetical gene supported by AB126076 |
| NP_116012.1 | 0.06 | 1.86 | 0.04 | - | ovary-specific acidic protein |
| - | -0.43 | 1.85 | 0.07 | - | hypothetical gene supported by BC043530 |
| TNRC5 | 0.27 | 1.85 | -0.30 | - | trinucleotide repeat containing 5 |
| - | -0.21 | 1.84 | -0.18 | AK096164 | - |
| - | -0.30 | 1.84 | -0.59 | AL137277 | - |
| - | -0.10 | 1.82 | -0.70 | AL137596 | - |
| NP_997289.1 | 0.46 | 1.81 | 0.41 | AK125953 | FLJ43965 protein |
| HLA-DMA | -0.47 | 1.79 | 0.46 | X62744 | major histocompatibility complex, class II, DM alpha |
| NP_056996.1 | -0.53 | 1.79 | -0.41 | AK091433,AF131744 | hypothetical protein LOC51059 |
| OR4N5 | -0.18 | 1.78 | 0.33 | - | olfactory receptor, family 4, subfamily N, member 5 |
| NFX1 | -0.30 | 1.77 | -0.13 | BC012151,AL832342 | nuclear transcription factor, X-box binding 1 |
| - | 0.07 | 1.77 | 0.34 | - | hypothetical LOC389666 |
| VDR | 0.19 | 1.77 | 0.17 | J03258 | vitamin D (1,25- dihydroxyvitamin D3) receptor |
| C14orf133 | -0.11 | 1.76 | -0.54 | AK098254 | - |
| - | -0.01 | 1.76 | -0.70 | AK091261 | - |
| - | 0.23 | 1.76 | 0.01 | AK096086 | - |
| - | -0.12 | 1.75 | 0.29 | - | - |
| - | -0.07 | 1.75 | 0.09 | AK002209 | - |
| CUGBP1 | 0.03 | 1.74 | -0.65 | BC031079 | CUG triplet repeat, RNA binding protein 1 |
| GABRR3 | -0.07 | 1.74 | 0.25 | - | gamma-aminobutyric acid (GABA) receptor, rho 3 |
| - | -0.22 | 1.73 | -0.08 | AK002199 | - |
| - | 0.07 | 1.72 | 0.04 | AL137346 | - |
| ITGA2 | 0.26 | 1.70 | 0.36 | X17033 | integrin, alpha 2 (CD49B, alpha 2 subunit of VLA-2 receptor) |
| - | -0.14 | 1.70 | 0.15 | - | hypothetical gene supported by NM_001001480 |
| RHAG | -0.22 | 1.69 | -0.13 | X64594 | Rhesus blood group-associated glycoprotein |
| GRID1 | -0.08 | 1.68 | -0.59 | AB033046 | glutamate receptor, ionotropic, delta 1 |
| - | 0.00 | 1.68 | -0.21 | - | - |
| C15orf27 | 0.11 | 1.68 | 0.05 | AK054866 | chromosome 15 open reading frame 27 |
| RCE1 | 0.00 | 1.68 | -0.24 | AL713740 | RCE1 homolog, prenyl protein protease (S. cerevisiae) |
| COL2A1 | -0.20 | 1.68 | 0.07 | - | collagen, type II, alpha 1 (primary osteoarthritis, spondyloepiphyseal dysplasia, congenital) |
| - | 0.00 | 1.68 | 0.26 | AK057297 | - |
| Q7Z2R7_HUMAN | 0.28 | 1.67 | 0.32 | AF176921 | - |
| - | 0.14 | 1.67 | 0.19 | AK098067 | - |
| - | 0.00 | 1.66 | -2.76 | - | LOC440632 |
| KAAG1 | 0.34 | 1.66 | 0.29 | AF181722 | kidney associated antigen 1 |
| NUMA1 | 0.46 | 1.65 | -0.77 | - | - |
| OR10AD1 | 0.00 | 1.65 | -0.90 | - | olfactory receptor, family 10, subfamily AD, member 1 |
| - | 0.18 | 1.64 | 0.38 | AB076353 | keratin associated protein 10-6 |
| - | 0.28 | 1.64 | 0.12 | - | similar to Hypothetical protein DJ845O24.2 |
| ADAMTS18 | -0.21 | 1.63 | -0.05 | BC063283 | a disintegrin-like and metalloprotease (reprolysin type) with thrombospondin type 1 motif, 18 |
| - | 0.00 | 1.63 | 0.18 | AK055074 | - |
| FTCD | 0.35 | 1.63 | -0.97 | U91541,AF289022 | formiminotransferase cyclodeaminase |
| - | 0.49 | 1.62 | 0.44 | - | similar to extensin-like protein |
| C9orf111 | 0.33 | 1.61 | 0.08 | AK055880,AK122590 | chromosome 9 open reading frame 111 |
| - | -0.09 | 1.61 | -0.63 | - | hypothetical LOC389665 |
| XP_032397.1 | 0.40 | 1.60 | 0.08 | AL080062 | DKFZP564I122 protein |
| Q96RZ4_HUMAN | 0.12 | 1.60 | -0.07 | - | - |
| - | -0.22 | 1.59 | 0.06 | AK055602 | - |
| AK7 | 0.24 | 1.58 | -0.42 | BC035256,AK057426 | adenylate kinase 7 |
| - | 0.11 | 1.58 | 0.34 | AK093878 | - |
| - | -0.87 | 1.58 | -0.48 | - | KIAA1817 protein |
| - | -0.77 | 1.58 | 0.27 | AK096897 | - |
| KIFC3 | -0.24 | 1.57 | 0.26 | BC001211 | kinesin family member C3 |
| SLC6A16 | -0.01 | 1.56 | 0.04 | - | solute carrier family 6 (neurotransmitter transporter), member 16 |
| RBM25 | 0.41 | 1.56 | -0.01 | - | - |
| XP_058879.7 | -0.09 | 1.56 | -1.64 | BC041772 | hypothetical protein LOC124976 |
| - | 0.48 | 1.56 | -0.13 | AK090737 | - |
| SLC7A6 | 0.09 | 1.55 | 0.06 | D87432,CR749291 | solute carrier family 7 (cationic amino acid transporter, y+ system), member 6 |
| FBXL14 | -0.21 | 1.55 | -0.89 | CR589998 | F-box and leucine-rich repeat protein 14 |
| NP_848647.1 | 0.42 | 1.55 | 0.13 | BC042635 | hypothetical protein MGC35206 |
| ZNF215 | 0.25 | 1.55 | -0.42 | AF056618 | zinc finger protein 215 |
| NP_872366.1 | 0.40 | 1.55 | -0.05 | AK098639 | hypothetical protein FLJ25773 |
| RAI3_HUMAN | 0.14 | 1.54 | -0.56 | AK001761 | G protein-coupled receptor, family C, group 5, member A |
| - | -0.17 | 1.54 | 0.27 | - | - |
| Q8NF01_HUMAN | -0.01 | 1.54 | -0.75 | AK090481 | - |
| GPR97 | 0.43 | 1.54 | -0.14 | AK172801 | G protein-coupled receptor 97 |
| EVPL | -0.12 | 1.54 | -0.07 | U53786 | envoplakin |
| SLC24A4 | 0.24 | 1.54 | 0.06 | - | solute carrier family 24 (sodium/potassium/calcium exchanger), member 4 |
| - | -0.47 | 1.53 | -0.14 | AK091305 | - |
| MLLT6 | 0.23 | 1.53 | 0.02 | AL133659 | myeloid/lymphoid or mixed-lineage leukemia (trithorax homolog, Drosophila); translocated to, 6 |
| XP_376003.1 | 0.38 | 1.53 | 0.38 | AK056895 | hypothetical gene supported by AK056895 |
| - | -0.01 | 1.53 | 0.03 | BI521010 | hypothetical gene supported by BC015564 |
| - | 0.03 | 1.52 | -0.20 | - | hypothetical LOC389782 |
| - | -0.62 | 1.52 | -1.59 | - | LOC441827 |
| NXPH4 | 0.12 | 1.52 | -0.54 | AK094231 | neurexophilin 4 |
| - | 0.42 | 1.52 | 0.49 | - | olfactory receptor, family 6, subfamily K, member 3 |
| MED12_HUMAN | 0.50 | 1.51 | -0.19 | AF071309 | mediator of RNA polymerase II transcription, subunit 12 homolog (yeast) |
| NP_060840.1 | 0.00 | 1.50 | 0.16 | AK094923,AK002121 | hypothetical protein FLJ11259 |
| Q8N8A0_HUMAN | -0.41 | 1.50 | -0.39 | AK097099 | - |
| WNT11 | 0.12 | 1.49 | -0.45 | - | wingless-type MMTV integration site family, member 11 |
| LPP | 0.09 | 1.49 | -0.66 | U49957 | LIM domain containing preferred translocation partner in lipoma |
| - | -0.02 | 1.49 | -0.27 | AK093579 | - |
| - | 0.06 | 1.49 | -0.14 | AK055008 | - |
| - | 0.00 | 1.49 | -0.18 | - | similar to Solute carrier family 12, member 3 |
| - | -0.11 | 1.49 | -0.98 | AL122065 | - |
| - | -0.16 | 1.48 | 0.46 | - | - |
| - | -0.87 | 1.48 | 0.04 | BC035743 | thioredoxin domain containing 8 |
| OR2J2 | 0.36 | 1.47 | -0.16 | - | - |
| - | 0.29 | 1.47 | 0.38 | - | - |
| NP_787118.2 | 0.00 | 1.47 | -0.10 | - | hypothetical protein MGC35308 |
| Q9NWJ4_HUMAN | 0.30 | 1.46 | -1.40 | - | - |
| IGSF6 | 0.10 | 1.46 | 0.44 | AJ223183 | immunoglobulin superfamily, member 6 |
| XP_028810.6 | -0.47 | 1.45 | 0.22 | AB051542 | KIAA1755 protein |
| Q8IV68_HUMAN | -0.04 | 1.45 | 0.30 | BI600443,BC068605 | similar to prostaglandin E receptor 4, subtype EP4; PGE receptor, EP4 subtype; prostaglandin E2 receptor |
| - | 0.27 | 1.45 | -0.41 | - | hypothetical LOC387854 |
| - | 0.30 | 1.45 | 0.29 | AK095097 | - |
| - | -0.02 | 1.44 | 0.20 | - | hypothetical LOC388291 |
| KIAA1529 | -0.72 | 1.44 | -0.32 | AB040962,CR627453 | KIAA1529 |
| - | 0.15 | 1.44 | 0.31 | - | - |
| XP_498717.1 | -0.70 | 1.44 | -1.29 | BE906094,AL122042,BG489998 | DKFZp564J157 protein |
| THTPA | -1.46 | 1.43 | 0.27 | - | thiamine triphosphatase |
| CU041_HUMAN | 0.12 | 1.43 | 0.11 | - | - |
| MLSTD1 | 0.35 | 1.43 | -0.05 | AK001927 | male sterility domain containing 1 |
| SYNPR | -0.71 | 1.43 | 0.10 | AL834457 | synaptoporin |
| - | -0.05 | 1.43 | 0.22 | AK094394 | - |
| ZC3HC1 | 0.24 | 1.42 | 0.19 | AK001317 | zinc finger, C3HC type 1 |
| - | 0.11 | 1.42 | -0.12 | - | LOC440011 |
| PRKG1 | 0.50 | 1.41 | -0.45 | Y07512 | protein kinase, cGMP-dependent, type I |
| - | 0.26 | 1.41 | 0.08 | BX107914 | hypothetical LOC387884 |
| RAD9B | -0.27 | 1.40 | 0.26 | BC047645 | RAD9 homolog B (S. cerevisiae) |
| - | 0.00 | 1.40 | 0.16 | BX647476 | sarcalumenin |
| KIAA1305 | -0.21 | 1.40 | -1.37 | AB037726 | KIAA1305 |
| - | -0.29 | 1.39 | -0.19 | AK131327 | FLJ16331 protein |
| - | 0.01 | 1.38 | -0.72 | - | - |
| HR | 0.00 | 1.38 | 0.20 | AF039196,AJ277165 | hairless homolog (mouse) |
| - | -0.20 | 1.38 | -0.39 | - | similar to RIKEN cDNA 5430404L10 |
| Q96NI9_HUMAN | -0.16 | 1.38 | -0.70 | - | - |
| TAC4 | -0.16 | 1.37 | -0.04 | - | tachykinin 4 (hemokinin) |
| - | 0.15 | 1.37 | -0.74 | - | - |
| aadA | 0.18 | 1.37 | -0.13 | AY442171 | - |
| ADCK1 | 0.29 | 1.37 | 0.25 | AK126928 | aarF domain containing kinase 1 |
| NP_001004318.1 | -0.06 | 1.36 | -0.32 | AK131245 | FLJ16165 protein |
| - | 0.20 | 1.36 | -0.30 | - | LOC440461 |
| BRUNOL5 | -1.14 | 1.36 | 0.03 | BC047522 | bruno-like 5, RNA binding protein (Drosophila) |
| OR51G1 | -0.25 | 1.36 | -1.38 | - | olfactory receptor, family 51, subfamily G, member 1 |
| - | -0.30 | 1.36 | -0.85 | AK093398 | - |
| IFNA2 | 0.32 | 1.36 | 0.34 | V00544 | interferon, alpha 2 |
| - | -0.46 | 1.36 | -0.52 | - | protocadherin gamma subfamily A, 7 |
| TNT_HUMAN | 0.33 | 1.36 | 0.05 | AF536240 | TNT protein |
| - | -0.18 | 1.35 | 0.50 | AK091251 | - |
| rxr | -0.14 | 1.35 | -0.48 | AF264696 | - |
| - | -0.26 | 1.35 | -0.11 | - | hypothetical LOC389437 |
| - | -0.30 | 1.35 | -0.34 | AL080094 | - |
| - | 0.48 | 1.34 | -1.26 | - | similar to Iroquois-class homeodomain protein IRX-1 (Iroquois homeobox protein 1) (Homeodomain protein IRXA1) |
| NP_001012276.1 | -0.12 | 1.34 | 0.05 | AK125568,AL049674 | hypothetical protein similar to preferentially expressed antigen of melanoma |
| - | 0.12 | 1.34 | 0.48 | AK056646 | - |
| STXB1_HUMAN | 0.02 | 1.33 | -0.13 | AF004563 | syntaxin binding protein 1 |
| - | 0.32 | 1.33 | -0.22 | - | LOC440094 |
| SLC22A5 | -0.35 | 1.33 | 0.31 | AF057164,AK128610 | solute carrier family 22 (organic cation transporter), member 5 |
| OR7A17 | -0.39 | 1.33 | 0.14 | X64993 | olfactory receptor, family 7, subfamily A, member 17 |
| lgTg | 0.26 | 1.33 | 0.46 | AJ276576 | - |
| - | 0.29 | 1.33 | -0.09 | BC052961 | hypothetical gene supported by AK128398 |
| Q8NH71_HUMAN | 0.10 | 1.32 | -0.71 | - | - |
| - | -0.21 | 1.32 | 0.38 | - | G protein-coupled receptor orphanA7 |
| - | -0.19 | 1.32 | 0.43 | - | similar to RBM17 protein |
| Q6ZR45_HUMAN | -0.38 | 1.32 | -0.52 | AK025456,D87455 | similar to hypothetical protein B230397C21 |
| - | 0.08 | 1.32 | 0.44 | - | hypothetical LOC387859 |
| - | -0.41 | 1.32 | 0.31 | AK054586 | - |
| PP1B_HUMAN | 0.14 | 1.31 | 0.35 | X80910 | protein phosphatase 1, catalytic subunit, beta isoform |
| - | -0.32 | 1.31 | 0.47 | AK057597 | - |
| - | -0.06 | 1.31 | -0.17 | AK097822 | - |
| BSF3_HUMAN | -0.23 | 1.31 | -0.90 | - | cardiotrophin-like cytokine |
| HIPK1 | 0.22 | 1.31 | -0.02 | - | homeodomain interacting protein kinase 1 |
| - | 0.15 | 1.31 | 0.06 | - | - |
| - | 0.39 | 1.31 | 0.03 | - | similar to testis-specific Y-encoded protein |
| - | -0.05 | 1.31 | -0.17 | CR749278,AB037734 | protocadherin 19 |
| - | -0.15 | 1.31 | 0.27 | AK090843 | - |
| NP_671727.1 | -0.51 | 1.31 | 0.49 | BC029175 | hypothetical protein MGC35361 |
| CRYGC | 0.48 | 1.30 | -0.04 | U66582 | crystallin, gamma C |
| - | -0.02 | 1.30 | -0.08 | - | similar to arginine-glutamic acid dipeptide (RE) repeats; atrophin 1-like; arginine glutamic acid dipeptide RE repeats |
| - | -0.29 | 1.30 | 0.23 | - | hypothetical LOC388563 |
| ERC2_HUMAN | 0.04 | 1.30 | -0.34 | AB002376 | CAZ-associated structural protein |
| PR285_HUMAN | -0.17 | 1.30 | 0.23 | AB051556,AF517673 | peroxisomal proliferator-activated receptor A interacting complex 285 |
| - | 0.26 | 1.30 | 0.23 | - | similar to Golgi autoantigen, golgin subfamily a, 2; Golgi matrix protein GM130; SY11 protein; golgin-95 |
| LV1G_HUMAN | -0.02 | 1.30 | 0.13 | S77011,BG760087 | - |
| ADPRH | 0.02 | 1.29 | 0.28 | L13291,BC020574 | ADP-ribosylarginine hydrolase |
| - | -0.05 | 1.29 | -0.42 | AK027139 | - |
| - | -0.12 | 1.28 | -0.50 | AK093257 | - |
| SMCR5 | 0.29 | 1.28 | 0.10 | AF467442 | - |
| - | -0.31 | 1.28 | -0.08 | AK021426 | - |
| - | -0.27 | 1.27 | -0.08 | - | hypothetical LOC388914 |
| SYT3 | 0.40 | 1.27 | 0.38 | AL136594 | synaptotagmin III |
| CTAG2 | 0.42 | 1.27 | 0.17 | AJ223040 | cancer/testis antigen 2 |
| ZNF503 | -1.13 | 1.27 | -0.16 | - | zinc finger protein 503 |
| PAPPA | -0.38 | 1.26 | 0.49 | - | pregnancy-associated plasma protein A, pappalysin 1 |
| C10orf10 | 0.20 | 1.26 | 0.09 | AB022718 | chromosome 10 open reading frame 10 |
| WDR17 | 0.47 | 1.25 | 0.00 | - | WD repeat domain 17 |
| PDCL2 | 0.28 | 1.25 | 0.09 | BC034431 | phosducin-like 2 |
| - | 0.48 | 1.25 | 0.10 | - | - |
| - | -0.48 | 1.25 | 0.46 | - | - |
| XP_046437.5 | 0.24 | 1.25 | -0.52 | BC035741,AL137678 | chromosome 20 open reading frame 50 |
| SIGLEC10 | 0.45 | 1.24 | 0.30 | AF311905 | sialic acid binding Ig-like lectin 10 |
| NP_079215.2 | -0.16 | 1.24 | 0.07 | BC069241 | hypothetical protein FLJ21918 |
| - | 0.10 | 1.24 | -2.97 | - | LOC440596 |
| PLA2G2D | -0.02 | 1.24 | -0.18 | BC025706 | phospholipase A2, group IID |
| WNT9A | 0.37 | 1.24 | -0.10 | - | wingless-type MMTV integration site family, member 9A |
| - | 0.03 | 1.24 | 0.20 | BX648524 | leucine rich repeat containing 22 |
| CXorf48 | 0.16 | 1.24 | -0.08 | AK000534,AY352211 | chromosome X open reading frame 48 |
| - | -0.04 | 1.24 | -0.29 | - | similar to Striatin |
| RRH | 0.35 | 1.24 | -0.07 | AF012270 | retinal pigment epithelium-derived rhodopsin homolog |
| - | -0.11 | 1.24 | -0.39 | - | chromosome 2 open reading frame 26 |
| ZADH2 | -0.17 | 1.24 | -0.52 | BC078661,BC018081 | zinc binding alcohol dehydrogenase, domain containing 2 |
| Q8N6Z3_HUMAN | -0.30 | 1.24 | 0.12 | D87682 | KIAA0241 protein |
| AFP | 0.29 | 1.23 | 0.46 | BC027881 | alpha-fetoprotein |
| - | 0.17 | 1.23 | -1.22 | - | similar to FoxB2 protein |
| Q96M16_HUMAN | -0.16 | 1.23 | 0.17 | CR627452,AK057461 | hypothetical protein DKFZp761P1121 |
| CA5BL | 0.02 | 1.23 | -0.03 | AK095608 | carbonic anhydrase VB-like |
| - | -0.27 | 1.23 | 0.04 | - | - |
| - | -1.54 | 1.22 | 0.46 | - | similar to KIAA0649 gene product |
| - | -0.14 | 1.22 | -0.55 | AL137463 | - |
| - | 0.00 | 1.22 | -0.36 | - | similar to Ig kappa variable region |
| - | -0.15 | 1.22 | 0.32 | - | - |
| XP_370686.1 | -0.09 | 1.22 | -0.80 | - | Rab15 effector protein |
| NEK4 | 0.43 | 1.21 | 0.14 | L20321 | NIMA (never in mitosis gene a)-related kinase 4 |
| Q6ZS70_HUMAN | 0.00 | 1.21 | 0.31 | - | KIAA1109 |
| - | -0.12 | 1.21 | -0.15 | AK094535 | - |
| NP_631918.1 | -1.16 | 1.21 | -0.34 | AF450090 | KCCR13L |
| XP_113967.2 | 0.00 | 1.21 | 0.44 | - | similar to Rab12 protein |
| MIPOL1 | -0.35 | 1.21 | -0.09 | BX647795 | mirror-image polydactyly 1 |
| SLC38A6 | -0.40 | 1.21 | -0.90 | AF070578,BC050349 | solute carrier family 38, member 6 |
| Q8N896_HUMAN | 0.15 | 1.21 | -0.40 | - | - |
| OMD | 0.00 | 1.21 | -0.05 | AB000114 | osteomodulin |
| NRX1B_HUMAN | 0.12 | 1.20 | 0.42 | AB035356,AB011150 | neurexin 1 |
| - | -0.08 | 1.20 | 0.30 | - | - |
| - | 0.00 | 1.20 | 0.38 | BI561187 | hypothetical LOC339760 |
| - | -0.30 | 1.20 | -0.55 | - | LOC440497 |
| - | -0.69 | 1.20 | -0.31 | - | LOC440237 |
| - | 0.31 | 1.20 | 0.44 | - | LOC440658 |
| CXCL13 | 0.17 | 1.19 | -0.35 | AF044197 | chemokine (C-X-C motif) ligand 13 (B-cell chemoattractant) |
| SCN1B | -0.18 | 1.19 | 0.16 | BC067122 | sodium channel, voltage-gated, type I, beta |
| TFR2 | 0.42 | 1.19 | 0.05 | AF067864 | transferrin receptor 2 |
| CLDN22 | -0.06 | 1.19 | 0.22 | - | claudin 22 |
| XP_496207.1 | -0.13 | 1.19 | 0.47 | - | - |
| - | 0.24 | 1.19 | 0.39 | - | similar to solute carrier family 16 (monocarboxylic acid transporters), member 14 |
| PADI6_HUMAN | -0.22 | 1.18 | 0.45 | AY422079 | peptidyl arginine deiminase, type VI |
| NGFR | -0.12 | 1.18 | -1.12 | M14764 | nerve growth factor receptor (TNFR superfamily, member 16) |
| - | -0.72 | 1.18 | 0.19 | BC031092 | hypothetical gene supported by BC031092 |
| IPF1 | 0.24 | 1.18 | -0.39 | X99894 | insulin promoter factor 1, homeodomain transcription factor |
| - | -0.86 | 1.18 | 0.17 | BC028204 | LOC441062 |
| NP_078791.2 | -0.19 | 1.18 | -0.76 | AY295082 | pre-mRNA splicing factor-like |
| - | 0.47 | 1.18 | 0.00 | - | similar to Hypothetical protein CBG22491 |
| - | 0.13 | 1.18 | -0.22 | AK055765 | - |
| Q8TBF0_HUMAN | -0.63 | 1.18 | 0.30 | AK128138 | hypothetical protein MGC26816 |
| NHLH2 | 0.49 | 1.17 | -0.94 | - | nescient helix loop helix 2 |
| - | 0.24 | 1.17 | -0.44 | AK096045 | - |
| Q8N6V0_HUMAN | 0.14 | 1.17 | -0.68 | - | hypothetical protein FLJ34389 |
| JPH1 | 0.19 | 1.17 | 0.29 | BC049372 | junctophilin 1 |
| GPR15 | -0.39 | 1.17 | 0.05 | BC069437 | G protein-coupled receptor 15 |
| NP_659454.1 | 0.13 | 1.17 | -0.50 | AK057333 | IIIG9 protein |
| XP_371731.1 | 0.09 | 1.17 | -0.36 | U21556 | similar to bA110H4.2 (similar to membrane protein) |
| - | -0.48 | 1.17 | -0.76 | AK090612 | - |
| - | 0.00 | 1.16 | 0.17 | U24186 | replication protein A4, 34kDa |
| OR4K2 | 0.14 | 1.16 | -0.56 | - | olfactory receptor, family 4, subfamily K, member 2 |
| - | 0.09 | 1.16 | -0.26 | - | hypothetical LOC283029 |
| Q9H375_HUMAN | -0.26 | 1.16 | -1.11 | - | - |
| - | -0.24 | 1.16 | -0.09 | - | similar to Ig kappa chain V region (Z4) - human |
| - | -0.21 | 1.16 | 0.33 | - | similar to folate receptor 1 precursor |
| KIF13B | -0.23 | 1.16 | 0.09 | AF279865,AB014539 | kinesin family member 13B |
| SLC34A2 | -0.15 | 1.16 | 0.15 | AF146796 | solute carrier family 34 (sodium phosphate), member 2 |
| EMX2 | 0.04 | 1.16 | -0.05 | AF301598 | empty spiracles homolog 2 (Drosophila) |
| MAGED4 | 0.28 | 1.15 | -0.22 | AK098830,AB058762,AB040529 | melanoma antigen family D, 4 |
| - | -0.47 | 1.15 | -0.30 | - | - |
| C1QTNF7 | 0.36 | 1.15 | -0.72 | BX647781 | C1q and tumor necrosis factor related protein 7 |
| Q9P168_HUMAN | -0.37 | 1.15 | 0.45 | - | - |
| Q7KZS0_HUMAN | 0.20 | 1.15 | -0.43 | - | - |
| PHLDA3 | -0.94 | 1.15 | 0.31 | - | - |
| - | 0.12 | 1.15 | -0.33 | AK128646,BG546907 | similar to alpha tubulin |
| - | -0.24 | 1.15 | 0.17 | - | - |
| - | 0.22 | 1.15 | 0.43 | AK057451 | hypothetical LOC389778 |
| Q9BRP3_HUMAN | -0.11 | 1.15 | -0.37 | CR611601 | v-maf musculoaponeurotic fibrosarcoma oncogene homolog G (avian) |
| - | 0.00 | 1.15 | 0.15 | - | similar to ribosomal protein S18 |
| - | -0.08 | 1.15 | -0.57 | - | hypothetical gene supported by AL832540 |
| ZN121_HUMAN | -0.72 | 1.14 | -1.38 | - | zinc finger protein 121 (clone ZHC32) |
| - | 0.11 | 1.14 | -0.38 | - | hypothetical LOC402110 |
| - | -0.07 | 1.13 | -0.83 | - | - |
| Q8N1Y0_HUMAN | -0.34 | 1.13 | -0.82 | AK056434,U43374 | unknown MGC21654 product |
| Q8N1K1_HUMAN | -0.30 | 1.13 | 0.48 | - | - |
| GRIN2C | 0.26 | 1.13 | 0.31 | U77782 | glutamate receptor, ionotropic, N-methyl D-aspartate 2C |
| - | 0.19 | 1.13 | 0.42 | BG485030 | hypothetical LOC387764 |
| Q6ZNQ3_HUMAN | 0.32 | 1.13 | 0.45 | - | - |
| - | -0.21 | 1.13 | -0.58 | - | similar to cyclophilin 18 |
| - | 0.33 | 1.13 | -0.26 | X06374,AU118732 | - |
| - | -0.62 | 1.13 | -0.26 | BF676541 | LOC440478 |
| C21orf90 | 0.32 | 1.13 | -0.27 | - | chromosome 21 open reading frame 90 |
| C20orf61 | -0.43 | 1.13 | -0.23 | - | - |
| - | 0.27 | 1.13 | -0.16 | - | RPL13-2 pseudogene |
| OR10J5 | -0.17 | 1.12 | 0.00 | - | olfactory receptor, family 10, subfamily J, member 5 |
| PROX1 | -0.14 | 1.12 | 0.10 | U44060,BC024201 | prospero-related homeobox 1 |
| - | -0.19 | 1.12 | -0.58 | - | - |
| ITGA2B | 0.02 | 1.12 | 0.05 | - | integrin, alpha 2b (platelet glycoprotein IIb of IIb/IIIa complex, antigen CD41B) |
| - | 0.03 | 1.12 | 0.22 | - | hypothetical LOC400968 |
| H1FOO | -0.18 | 1.12 | 0.23 | BM564157 | H1 histone family, member O, oocyte-specific |
| GCNT3 | 0.31 | 1.11 | -0.28 | AF102542 | glucosaminyl (N-acetyl) transferase 3, mucin type |
| XP_372466.1 | 0.38 | 1.11 | -2.32 | - | similar to poly(A) binding protein, cytoplasmic 4 isoform 2 |
| - | -0.04 | 1.11 | 0.09 | BU960446 | hypothetical LOC389189 |
| XP_371139.1 | 0.38 | 1.11 | -0.63 | - | hypothetical protein FLJ14959 |
| PIGQ | 0.00 | 1.11 | 0.48 | AB003723 | phosphatidylinositol glycan, class Q |
| CACNA1H | -0.43 | 1.11 | 0.10 | AF073931 | calcium channel, voltage-dependent, alpha 1H subunit |
| ADAM7 | -0.36 | 1.10 | -0.29 | AF215824,AF090327 | a disintegrin and metalloproteinase domain 7 |
| - | -0.18 | 1.10 | 0.09 | - | similar to mesenchymal stem cell protein DSC92; neurite outgrowth associated protein |
| - | -0.61 | 1.10 | -0.65 | - | similar to Von Ebners gland protein precursor (VEG protein) (Tear prealbumin) (TP) (Tear lipocalin) (Lipocalin 1) |
| TTTY12 | 0.00 | 1.10 | -1.25 | AF332241 | - |
| INGX | -0.29 | 1.10 | -0.40 | AF149724 | Homo sapiens inhibitor of growth family, X-linked, pseudogene (INGX) on chromosome X |
| NP_054900.1 | -0.09 | 1.10 | 0.11 | BC036082 | HSPC159 protein |
| ZNF297 | 0.49 | 1.09 | -1.54 | BC018541 | zinc finger protein 297 |
| CCNT1 | 0.28 | 1.09 | -0.06 | AF048730 | cyclin T1 |
| Q8N4I1_HUMAN | -0.10 | 1.09 | -0.15 | BC016161,BC034052 | thymus expressed gene 3-like |
| Q8N9H1_HUMAN | 0.42 | 1.09 | 0.37 | - | - |
| - | 0.00 | 1.09 | -0.32 | AK057967 | - |
| C20orf82 | -0.19 | 1.09 | -0.25 | - | chromosome 20 open reading frame 82 |
| NP_077298.1 | 0.15 | 1.09 | 0.37 | AK027859 | hypothetical protein MGC11266 |
| ELA3A | -0.16 | 1.09 | 0.20 | - | elastase 3B, pancreatic |
| - | 0.49 | 1.09 | -0.26 | AK094134 | - |
| TRIM1_HUMAN | 0.32 | 1.08 | -0.18 | Y18880 | midline 2 |
| - | -0.10 | 1.08 | -0.36 | - | hypothetical gene supported by AK023501 |
| - | 0.09 | 1.08 | -0.04 | BC043364 | hypothetical protein LOC338809 |
| - | -0.11 | 1.08 | 0.23 | - | hypothetical gene supported by BC019832; NM_012200 |
| Q9C0K3_HUMAN | -0.19 | 1.08 | 0.29 | - | - |
| - | -0.06 | 1.08 | -0.49 | - | similar to zinc finger protein 91 (HPF7, HTF10) |
| - | -0.42 | 1.07 | 0.24 | - | gamma-aminobutyric acid (GABA) receptor, rho 3 |
| - | 0.28 | 1.07 | 0.39 | AK127919 | FLJ46026 protein |
| - | -0.01 | 1.07 | 0.33 | AK057765 | - |
| DMC1 | 0.04 | 1.07 | -0.27 | D64108 | DMC1 dosage suppressor of mck1 homolog, meiosis-specific homologous recombination (yeast) |
| - | 0.13 | 1.07 | 0.39 | - | hypothetical LOC389438 |
| EPB41L1 | 0.28 | 1.06 | 0.21 | BC013885,AB002336 | erythrocyte membrane protein band 4.1-like 1 |
| - | 0.22 | 1.06 | 0.49 | - | hypothetical LOC388298 |
| APG7L | 0.43 | 1.06 | -0.85 | AL122075 | APG7 autophagy 7-like (S. cerevisiae) |
| - | -0.13 | 1.06 | 0.24 | AL133086 | - |
| Q8N793_HUMAN | -0.48 | 1.05 | -0.48 | AK098761 | - |
| IDE | -0.02 | 1.05 | -0.16 | M21188,BX648462 | insulin-degrading enzyme |
| ZNF509 | -0.09 | 1.05 | 0.33 | AK127560 | zinc finger protein 509 |
| MAPT | 0.15 | 1.05 | 0.30 | - | - |
| - | 0.29 | 1.05 | 0.08 | AK127023 | FLJ45079 protein |
| SALF_HUMAN | -0.40 | 1.05 | 0.02 | - | TFIIA-alpha/beta-like factor |
| NP_775778.1 | 0.17 | 1.05 | 0.10 | AK095152 | hypothetical protein FLJ37118 |
| OSBPL7 | -0.12 | 1.05 | 0.35 | BC065482 | oxysterol binding protein-like 7 |
| - | -0.24 | 1.05 | -0.04 | AB046773 | KIAA1553 |
| GPR112 | 0.19 | 1.04 | -0.99 | AY140954 | G protein-coupled receptor 112 |
| SNTG1 | 0.24 | 1.04 | 0.48 | AL161971 | syntrophin, gamma 1 |
| HOXD11 | 0.09 | 1.04 | -0.59 | - | homeo box D11 |
| NP_149989.1 | 0.02 | 1.04 | -0.02 | BC009941 | hypothetical protein LOC92922 |
| - | 0.12 | 1.04 | 0.04 | AK022479 | - |
| SIA8C_HUMAN | -0.26 | 1.04 | -0.26 | AF004668 | sialyltransferase 8C (alpha2,3Galbeta1,4GlcNAcalpha 2,8-sialyltransferase) |
| TRIM31 | 0.14 | 1.04 | 0.17 | X81006 | tripartite motif-containing 31 |
| RASL12 | -0.55 | 1.03 | -0.16 | BC053734,AF233588 | RAS-like, family 12 |
| - | 0.22 | 1.03 | 0.10 | - | similar to polycythemia rubra vera 1; cell surface receptor |
| - | 0.01 | 1.03 | -0.48 | AK093324 | - |
| - | 0.29 | 1.03 | 0.08 | AK093681 | - |
| ARHGEF12 | -0.32 | 1.03 | 0.04 | - | - |
| - | 0.35 | 1.03 | 0.24 | - | similar to ENSANGP00000010305 |
| HERC6 | -0.29 | 1.03 | 0.32 | AK000644,BC042047 | hect domain and RLD 6 |
| - | -0.12 | 1.03 | -0.85 | - | hypothetical LOC388898 |
| C9orf157 | 0.42 | 1.03 | 0.21 | BC031861 | chromosome 9 open reading frame 157 |
| - | -0.32 | 1.03 | -0.30 | - | - |
| ACAS2 | 0.42 | 1.03 | 0.31 | AK000188,BC012172 | acetyl-Coenzyme A synthetase 2 (ADP forming) |
| SVIL | 0.01 | 1.03 | 0.34 | - | supervillin |
| PTGER3 | 0.20 | 1.02 | -0.01 | - | prostaglandin E receptor 3 (subtype EP3) |
| FTSJ3 | 0.13 | 1.02 | 0.41 | AF327355,AK000069 | FtsJ homolog 3 (E. coli) |
| KCNJ6 | -0.53 | 1.02 | 0.36 | - | potassium inwardly-rectifying channel, subfamily J, member 6 |
| MYEOV | 0.44 | 1.02 | -0.31 | AJ223366 | myeloma overexpressed gene (in a subset of t(11;14) positive multiple myelomas) |
| smTg | -0.12 | 1.02 | 0.36 | AJ276576 | - |
| - | 0.02 | 1.02 | 0.05 | AK055242 | - |
| - | 0.00 | 1.02 | 0.40 | X81053 | collagen, type IV, alpha 4 |
| PYGO2 | -0.19 | 1.02 | 0.16 | BC006132 | pygopus homolog 2 (Drosophila) |
| - | 0.45 | 1.02 | -1.80 | - | similar to actin 3 - fruit fly (Drosophila melanogaster) (fragments) |
| - | 0.37 | 1.02 | 0.39 | AK056234 | - |
| - | -0.47 | 1.02 | 0.48 | AK055966 | - |
| - | -0.21 | 1.02 | 0.23 | - | similar to elongation factor 1 delta |
| VSV-g | -0.14 | 1.01 | -0.31 | AJ318514 | - |
| NP_689722.1 | -0.03 | 1.01 | 0.49 | - | hypothetical protein FLJ31568 |
| - | -0.04 | 1.01 | 0.32 | - | hypothetical LOC339453 |
| - | -0.46 | 1.01 | 0.03 | - | similar to Tryptophanyl-tRNA synthetase (Tryptophan--tRNA ligase) (TrpRS) (IFP53) (hWRS) |
| - | 0.42 | 1.01 | -0.97 | AK094892 | - |
| - | 0.38 | 1.01 | -0.08 | AK098335 | - |
| NOS1 | 0.43 | 1.01 | -0.74 | U17327 | nitric oxide synthase 1 (neuronal) |
| APLP1 | -0.75 | 1.01 | -0.62 | U48437 | amyloid beta (A4) precursor-like protein 1 |
| EEF2K | -0.03 | 1.01 | -0.35 | U93850,BC032665 | eukaryotic elongation factor-2 kinase |
| - | -0.35 | 1.01 | -1.44 | BC008503 | hypothetical protein LOC255326 |
| - | 0.25 | 1.01 | -0.18 | AL833566 | - |
| KIAA1468 | -0.17 | 1.00 | 0.41 | BX648481,AB040901 | - |
| - | 0.35 | 1.00 | 0.28 | AK093834 | - |
| CBL | 0.27 | 1.00 | 0.16 | X57110 | Cas-Br-M (murine) ecotropic retroviral transforming sequence |
| NP_057015.1 | 0.00 | 1.00 | -0.18 | AK122938,AB024705 | fls485 |
| - | -0.87 | 1.00 | 0.34 | AK096075 | - |
| - | -0.08 | 1.00 | -0.61 | AK091440 | - |
| - | -0.20 | 1.00 | 0.41 | BC036527 | - |
| - | -0.28 | 1.00 | -0.31 | - | similar to Interferon-induced protein with tetratricopeptide repeats 1 (IFIT-1) (Interferon-induced 56 kDa protein) (IFI-56K) |
| - | -0.43 | 1.00 | -0.88 | - | collagen, type V, alpha 2 |
| MRGRE_HUMAN | -0.14 | 1.00 | -0.51 | AY255572 | MAS-related GPR, member E |
| Q96PZ4_HUMAN | 0.05 | 0.99 | 0.20 | AB067480,BC052950 | G protein-regulated inducer of neurite outgrowth 1 |
| STH_HUMAN | -0.06 | 0.99 | 0.44 | - | saitohin |
| - | -0.02 | 0.99 | -0.38 | AK021924 | - |
| PLCE1 | 0.27 | 0.99 | 0.34 | AB040949,AF190642 | phospholipase C, epsilon 1 |
| XP_375302.2 | 0.29 | 0.99 | 0.35 | AK126539 | hypothetical gene supported by AK126539 |
| FBXO7 | 0.25 | 0.99 | 0.14 | BX648151,AL050254 | F-box protein 7 |
| K1162_HUMAN | -0.08 | 0.99 | -0.27 | BC044777,AB032988 | - |
| KIAA0934 | 0.38 | 0.99 | -1.65 | AB023151 | KIAA0934 |
| CD5L | -0.25 | 0.99 | 0.28 | U82812 | CD5 antigen-like (scavenger receptor cysteine rich family) |
| - | -0.43 | 0.99 | 0.37 | AK057336 | - |
| EPC1 | 0.12 | 0.98 | 0.46 | - | enhancer of polycomb homolog 1 (Drosophila) |
| - | 0.23 | 0.98 | 0.38 | - | chromosome 10 open reading frame 130 |
| - | 0.00 | 0.98 | -0.06 | BC053499 | similar to chromosome 10 open reading frame 88; Em:AC073585.5 |
| HES7 | -0.17 | 0.98 | 0.38 | - | hairy and enhancer of split 7 (Drosophila) |
| - | 0.20 | 0.98 | 0.22 | - | similar to HS1 binding protein |
| - | 0.12 | 0.98 | -0.08 | AK054946 | - |
| Q8N7A7_HUMAN | -0.02 | 0.98 | -0.32 | AL832562 | - |
| Q86X12_HUMAN | -0.44 | 0.97 | -0.01 | - | - |
| SLC2A9 | 0.32 | 0.97 | 0.21 | - | solute carrier family 2 (facilitated glucose transporter), member 9 |
| - | -0.27 | 0.97 | -0.86 | AL833418 | pancreatic lipase-related protein 3 |
| - | 0.02 | 0.97 | -0.05 | AK128015 | hypothetical gene supported by AK128015 |
| NR4A1 | -0.10 | 0.97 | 0.36 | D49728 | nuclear receptor subfamily 4, group A, member 1 |
| - | 0.37 | 0.97 | 0.00 | AK091204 | - |
| OR56A4 | 0.20 | 0.97 | 0.24 | - | olfactory receptor, family 56, subfamily A, member 4 |
| - | -0.02 | 0.97 | -0.39 | AK000966 | - |
| VILL | -0.02 | 0.96 | 0.31 | D88154,BC004300 | villin-like |
| KCNG4 | -0.50 | 0.96 | 0.39 | - | potassium voltage-gated channel, subfamily G, member 4 |
| - | 0.39 | 0.96 | 0.31 | - | similar to peptidylprolyl isomerase A isoform 1; cyclophilin A; peptidyl-prolyl cis-trans isomerase A; T cell cyclophilin; rotamase; cyclosporin A-binding protein |
| CCDC6 | 0.44 | 0.96 | -0.31 | AK055515,BC036757 | coiled-coil domain containing 6 |
| NP_659440.1 | 0.14 | 0.96 | 0.35 | AK055726 | hypothetical protein FLJ31164 |
| - | -0.07 | 0.96 | 0.16 | AK094533 | - |
| - | 0.07 | 0.96 | 0.03 | - | similar to chaperonin containing TCP1, subunit 6A (zeta 1); chaperonin containing T-complex subunit 6 |
| ADAMTS5 | 0.33 | 0.95 | -0.18 | AF142099 | a disintegrin-like and metalloprotease (reprolysin type) with thrombospondin type 1 motif, 5 (aggrecanase-2) |
| NP_775936.1 | 0.00 | 0.95 | -0.48 | BX648338 | hypothetical protein MGC34713 |
| - | -0.49 | 0.95 | -0.33 | - | similar to ataxin 2-binding protein 1 isoform 4; hexaribonucleotide binding protein 1 |
| RASGRP2 | -0.02 | 0.95 | -0.15 | Y12336,AK092882 | RAS guanyl releasing protein 2 (calcium and DAG-regulated) |
| NP_997205.1 | -0.01 | 0.95 | -0.21 | BX648578 | nuclear localized factor 1 |
| UBQLN3 | 0.30 | 0.95 | 0.10 | BC036743 | ubiquilin 3 |
| XP_209936.3 | 0.34 | 0.95 | -0.16 | BC055413 | similar to RIKEN cDNA 1700011J18 |
| NDUFB3 | 0.00 | 0.95 | -0.75 | AF047183 | NADH dehydrogenase (ubiquinone) 1 beta subcomplex, 3, 12kDa |
| - | 0.17 | 0.94 | -0.40 | - | similar to dJ1184F4.4 (novel protein similar to nucleolar protein 4 (NOL4) (NOLP)) |
| C19orf28 | 0.05 | 0.94 | 0.16 | BC068439 | chromosome 19 open reading frame 28 |
| - | 0.47 | 0.94 | 0.21 | - | hypothetical LOC389331 |
| - | -1.47 | 0.94 | 0.30 | AK055856 | - |
| - | -0.93 | 0.94 | 0.39 | BC041889 | TPTE pseudogene |
| HSPB8 | 0.00 | 0.94 | -0.70 | AF191017 | heat shock 22kDa protein 8 |
| - | 0.06 | 0.94 | -0.13 | AK022298 | - |
| - | -1.09 | 0.94 | -0.26 | AK094169 | - |
| XP_374880.2 | 0.40 | 0.94 | 0.23 | BC065704 | hypothetical gene supported by BC065704 |
| - | 0.12 | 0.94 | -0.23 | AK091524 | - |
| - | -0.43 | 0.93 | -0.47 | - | LOC441851 |
| ARHGEF17 | 0.23 | 0.93 | -0.24 | AB002335,AF378754 | Rho guanine nucleotide exchange factor (GEF) 17 |
| - | -0.26 | 0.93 | -0.03 | AK097624 | - |
| ZNF575 | -0.32 | 0.93 | 0.32 | AK057129 | zinc finger protein 575 |
| - | 0.18 | 0.93 | -0.26 | - | similar to Dystroglycan precursor (Dystrophin-associated glycoprotein 1) |
| RFPL1 | 0.12 | 0.93 | -0.51 | AJ010228 | ret finger protein-like 1 |
| PRKAA1 | -0.02 | 0.93 | 0.09 | BC012622 | - |
| NP_110381.1 | 0.02 | 0.93 | -0.30 | - | - |
| - | 0.14 | 0.93 | -0.55 | M74161,BC058932 | inositol polyphosphate-5-phosphatase, 75kDa |
| - | -0.04 | 0.93 | -0.79 | BC053591,AB037779 | DKFZP434B0335 protein |
| - | 0.04 | 0.92 | 0.00 | - | similar to hypothetical protein FLJ23834 |
| - | 0.50 | 0.92 | -0.32 | - | similar to chromosome 9 open reading frame 85 isoform a |
| NP_848609.1 | -0.10 | 0.92 | 0.10 | BX647632 | hypothetical protein LOC283487 |
| QPCT | 0.16 | 0.92 | 0.46 | X71125,BC047756 | glutaminyl-peptide cyclotransferase (glutaminyl cyclase) |
| SLC39A5 | 0.23 | 0.92 | 0.18 | AK172768 | solute carrier family 39 (metal ion transporter), member 5 |
| XP_291770.4 | 0.00 | 0.92 | -0.26 | - | similar to ankyrin repeat domain 30A; breast cancer antigen NY-BR-1 |
| MOV10 | -0.01 | 0.92 | -0.26 | AK074174 | - |
| NP_116099.1 | -0.25 | 0.92 | -0.04 | BC001183 | hypothetical protein MGC13053 |
| Q96B84_HUMAN | 0.44 | 0.92 | -1.19 | - | - |
| TMF1 | -0.92 | 0.92 | -0.06 | L01042 | TATA element modulatory factor 1 |
| - | 0.26 | 0.92 | 0.48 | AK074178 | similar to mFLJ00251 protein |
| XP_211908.3 | -0.75 | 0.91 | -0.56 | - | hypothetical gene supported by NM_030930; AY007125 |
| SNX8 | -0.25 | 0.91 | 0.46 | AF121858 | sorting nexin 8 |
| - | 0.01 | 0.91 | -0.89 | - | - |
| - | -0.45 | 0.91 | -1.34 | AK128136 | FLJ46257 protein |
| - | 0.32 | 0.91 | 0.15 | AL137368 | - |
| - | -0.13 | 0.91 | -0.13 | - | T-cell leukemia/lymphoma 6 |
| - | -0.81 | 0.91 | -0.24 | AL832649 | - |
| CYP2J2 | 0.19 | 0.91 | -0.32 | U37143 | cytochrome P450, family 2, subfamily J, polypeptide 2 |
| SMYD1 | 0.22 | 0.91 | 0.47 | AK022290,AL832035 | SET and MYND domain containing 1 |
| - | 0.00 | 0.91 | -0.19 | - | hypothetical LOC387887 |
| - | 0.15 | 0.91 | -0.20 | - | similar to Mucin 4 (Tracheobronchial mucin) |
| IGSF9 | -0.22 | 0.91 | -0.37 | AB037776 | immunoglobulin superfamily, member 9 |
| JUN | -0.23 | 0.91 | -0.18 | BC068522 | v-jun sarcoma virus 17 oncogene homolog (avian) |
| PLCD4 | -0.12 | 0.90 | 0.28 | - | phospholipase C, delta 4 |
| - | -0.07 | 0.90 | 0.26 | BM664667,BQ774510 | similar to Heat shock transcription factor, Y-linked (Heat shock transcription factor 2-like protein) (HSF2-like) |
| - | -0.05 | 0.90 | -1.04 | - | hypothetical LOC389440 |
| - | -0.35 | 0.90 | 0.26 | AF000980 | basic charge, Y-linked, 2C |
| - | -0.15 | 0.90 | -0.03 | - | similar to filamin-binding LIM protein-1; likely ortholog of mouse CSX-associated LIM |
| SLC6A3 | 0.22 | 0.90 | 0.35 | M95167 | solute carrier family 6 (neurotransmitter transporter, dopamine), member 3 |
| - | -0.01 | 0.90 | -0.50 | BC049373 | hypothetical protein LOC151300 |
| LASP1 | 0.09 | 0.90 | -0.41 | X82456 | LIM and SH3 protein 1 |
| NP_060328.1 | 0.13 | 0.90 | 0.15 | AK000523 | timeless-interacting protein |
| C20orf90 | 0.00 | 0.90 | 0.14 | - | - |
| - | 0.00 | 0.90 | -0.20 | - | LOC439946 |
| - | 0.45 | 0.90 | 0.28 | AK123403,BC068588 | hypothetical gene supported by AK123403 |
| - | -0.06 | 0.90 | -0.01 | BC028905 | - |
| - | 0.32 | 0.90 | -0.49 | AK025052 | - |
| - | 0.09 | 0.90 | -0.49 | AK092057 | - |
| - | 0.04 | 0.90 | -0.60 | - | similar to RIKEN cDNA 2810417M05 |
| CXCR6 | -0.10 | 0.90 | -0.27 | - | chemokine (C-X-C motif) receptor 6 |
| CLCA3 | 0.31 | 0.90 | -0.45 | AF043976 | chloride channel, calcium activated, family member 3 |
| XP_044062.6 | -0.92 | 0.90 | -0.96 | - | hypothetical protein DKFZp761O2018 |
| OR2T1 | -0.24 | 0.90 | -1.25 | - | olfactory receptor, family 2, subfamily T, member 1 |
| Q7Z585_HUMAN | -0.04 | 0.90 | 0.00 | - | - |
| - | 0.09 | 0.90 | 0.45 | - | hypothetical gene supported by BC039671 |
| - | -0.05 | 0.90 | 0.37 | - | - |
| - | 0.00 | 0.89 | -0.49 | - | similar to hypothetical protein |
| - | 0.00 | 0.89 | 0.43 | AK058075 | - |
| XP_497611.1 | -0.05 | 0.89 | 0.31 | - | similar to cytochrome P-450 |
| - | 0.11 | 0.89 | 0.33 | - | similar to hypothetical protein, MNCb-4779 |
| - | -1.04 | 0.89 | -0.04 | AK057476 | - |
| NP_078896.2 | 0.39 | 0.89 | -0.17 | BC047501 | hypothetical protein FLJ12586 |
| HSH2D | 0.13 | 0.89 | 0.10 | AK131222 | hematopoietic SH2 domain containing |
| LATS1 | -0.16 | 0.89 | -0.27 | AF104413 | LATS, large tumor suppressor, homolog 1 (Drosophila) |
| EFNB1 | 0.00 | 0.89 | 0.48 | BC016649 | ephrin-B1 |
| Q9H729_HUMAN | -0.38 | 0.89 | 0.01 | - | - |
| STRN | 0.37 | 0.89 | -0.11 | AK026934,AJ223814 | striatin, calmodulin binding protein |
| - | 0.36 | 0.89 | 0.47 | D81199,AL137524 | DNM1DN11.13 duplicon |
| Q8TEE4_HUMAN | 0.15 | 0.88 | 0.43 | AF521671 | AT rich interactive domain 1B (SWI1-like) |
| C4orf9 | 0.30 | 0.88 | 0.19 | AF040965 | chromosome 4 open reading frame 9 |
| Q8N413_HUMAN | 0.25 | 0.88 | 0.11 | - | hypothetical protein LOC283130 |
| PRAP1 | 0.33 | 0.88 | 0.18 | AF421885 | proline-rich acidic protein 1 |
| PRRX1 | 0.20 | 0.88 | -0.03 | - | paired related homeobox 1 |
| - | 0.12 | 0.88 | 0.04 | - | protocadherin alpha 7 |
| XP_376727.1 | 0.11 | 0.88 | -0.18 | - | hypothetical protein LOC285888 |
| ITPK1 | 0.29 | 0.88 | 0.27 | BC003622 | - |
| Q8NGB5_HUMAN | 0.00 | 0.88 | 0.43 | - | - |
| NP_001008537.1 | -0.57 | 0.88 | -0.87 | AY563507 | KIAA2022 protein |
| - | -0.09 | 0.88 | 0.04 | - | - |
| - | -0.26 | 0.88 | 0.05 | AK091720 | - |
| NP_061330.2 | -0.06 | 0.87 | 0.16 | - | insulin receptor tyrosine kinase substrate |
| - | -0.26 | 0.87 | 0.48 | AK095147 | - |
| GPR143 | 0.19 | 0.87 | 0.11 | Z48804 | G protein-coupled receptor 143 |
| ACRC | -0.05 | 0.87 | 0.27 | AK127607 | acidic repeat containing |
| MRRF | -0.31 | 0.87 | 0.05 | - | mitochondrial ribosome recycling factor |
| - | -0.53 | 0.87 | 0.02 | - | similar to CG3047-PA |
| - | 0.48 | 0.87 | 0.26 | AL137535 | - |
| - | 0.32 | 0.87 | 0.30 | - | - |
| Q8N8G7_HUMAN | 0.20 | 0.87 | 0.41 | AK096849,BC037865 | - |
| TDO2 | 0.19 | 0.87 | -0.73 | U32989,BX647341 | tryptophan 2,3-dioxygenase |
| Q8N5E0_HUMAN | 0.23 | 0.87 | 0.21 | AK055035 | hypothetical protein FLJ30473 |
| NP_997345.1 | 0.47 | 0.87 | 0.01 | AK127589 | FLJ45684 protein |
| - | 0.43 | 0.87 | -0.28 | CA453248 | hypothetical LOC388893 |
| CASK | 0.30 | 0.87 | -0.29 | AF035582 | calcium/calmodulin-dependent serine protein kinase (MAGUK family) |
| GR6_HUMAN | 0.33 | 0.86 | -0.07 | AF008192 | putative GR6 protein |
| - | -0.71 | 0.86 | 0.39 | - | similar to prostaglandin E receptor 4, subtype EP4; PGE receptor, EP4 subtype; prostaglandin E2 receptor |
| IFNA17 | 0.40 | 0.86 | 0.23 | M11026 | interferon, alpha 17 |
| NP_005199.2 | 0.33 | 0.86 | 0.40 | - | crystallin, beta A1 |
| XP_498129.1 | 0.48 | 0.86 | -0.85 | - | similar to glutathione transferase M2 |
| MSLN | 0.47 | 0.86 | 0.01 | - | mesothelin |
| Q8NEF6_HUMAN | 0.45 | 0.86 | 0.34 | BC031230 | hypothetical protein LOC255177 |
| - | -0.20 | 0.86 | 0.34 | - | similar to Glyceraldehyde 3-phosphate dehydrogenase, liver (GAPDH) |
| - | 0.27 | 0.86 | -0.76 | BC040593 | LOC441275 |
| - | 0.00 | 0.86 | -0.85 | AK023572 | - |
| HDAC9 | 0.12 | 0.86 | -0.28 | AJ459808 | histone deacetylase 9 |
| MYH8 | 0.38 | 0.85 | -0.21 | M36769 | myosin, heavy polypeptide 8, skeletal muscle, perinatal |
| Q9H385_HUMAN | -0.14 | 0.85 | -0.19 | - | - |
| RNPC1 | -0.31 | 0.85 | -0.13 | - | RNA-binding region (RNP1, RRM) containing 1 |
| TBC1D10 | 0.32 | 0.85 | -0.98 | AK131086 | TBC1 domain family, member 10 |
| FKHL18 | 0.29 | 0.85 | -0.39 | BC013408 | forkhead-like 18 (Drosophila) |
| Q96MD1_HUMAN | 0.21 | 0.85 | 0.50 | - | - |
| - | -0.06 | 0.85 | 0.38 | - | similar to SNARE protein Ykt6; YKT6, S. cerevisiae, homolog of |
| - | 0.17 | 0.85 | -0.35 | AK057317 | hypothetical protein LOC147004 |
| Q9NVK6_HUMAN | 0.32 | 0.85 | 0.21 | AF110377 | transformation/transcription domain-associated protein |
| Q8NGU7_HUMAN | 0.00 | 0.85 | -0.44 | - | - |
| KCNA6 | -0.64 | 0.85 | -0.03 | X17622 | potassium voltage-gated channel, shaker-related subfamily, member 6 |
| WDR9_HUMAN | -0.57 | 0.85 | 0.43 | AK002177,AJ238214 | WD repeat domain 9 |
| - | -0.09 | 0.85 | 0.23 | BQ056804 | hypothetical LOC388823 |
| ARRB1 | 0.48 | 0.85 | 0.27 | AF084040,BC003636 | arrestin, beta 1 |
| T2R55_HUMAN | 0.16 | 0.85 | -0.24 | - | taste receptor T2R55 |
| - | 0.13 | 0.85 | 0.18 | - | hypothetical LOC151154 |
| ZNF219 | -1.30 | 0.85 | 0.11 | AB015427 | zinc finger protein 219 |
| NP_872322.1 | 0.00 | 0.85 | -0.81 | AK056573 | hypothetical protein FLJ32011 |
| LHFPL2 | 0.11 | 0.85 | 0.01 | D86961,AY309920 | lipoma HMGIC fusion partner-like 2 |
| NM_032947.3 | -0.14 | 0.84 | 0.49 | AK125286,AK027847 | putative small membrane protein NID67 |
| - | -0.10 | 0.84 | 0.10 | BC068610,AK024602 | hypothetical gene supported by AK024602 |
| NP_689574.1 | -0.23 | 0.84 | 0.33 | BC009725,AK096263 | hypothetical protein FLJ38944 |
| - | -0.41 | 0.84 | -0.32 | AK021679 | - |
| ACP6 | 0.20 | 0.84 | -1.04 | AB031478,BC034686 | acid phosphatase 6, lysophosphatidic |
| C6orf114 | -0.23 | 0.84 | -0.42 | AF264036 | chromosome 6 open reading frame 114 |
| NP_079049.1 | -0.16 | 0.84 | 0.17 | BC027911 | hypothetical protein FLJ13798 |
| - | -0.15 | 0.84 | -0.38 | - | hypothetical LOC339875 |
| - | 0.37 | 0.84 | 0.36 | - | hypothetical LOC388586 |
| - | 0.00 | 0.84 | -0.29 | - | similar to hypothetical protein LOC284701 |
| FDPS | -0.22 | 0.84 | 0.13 | D14697 | farnesyl diphosphate synthase (farnesyl pyrophosphate synthetase, dimethylallyltranstransferase, geranyltranstransferase) |
| - | 0.25 | 0.84 | -0.09 | - | transmembrane protein 16G |
| - | 0.00 | 0.84 | 0.18 | - | - |
| SLC5A4 | -0.48 | 0.84 | 0.26 | AJ133127 | solute carrier family 5 (low affinity glucose cotransporter), member 4 |
| - | 0.29 | 0.84 | 0.13 | CR595224 | LOC441214 |
| SLC4A10 | 0.00 | 0.84 | 0.27 | BC051190 | solute carrier family 4, sodium bicarbonate transporter-like, member 10 |
| - | -0.03 | 0.84 | 0.41 | - | similar to NAD-dependent deacetylase sirtuin 5 (SIR2-like protein 5) |
| Q9UL76_HUMAN | 0.13 | 0.83 | 0.07 | - | - |
| Q9HBS7_HUMAN | 0.00 | 0.83 | -0.32 | - | - |
| ZFP36 | 0.40 | 0.83 | -0.14 | CR597101,M92843 | zinc finger protein 36, C3H type, homolog (mouse) |
| CNIH3 | -0.16 | 0.83 | -0.70 | AF070524,CR595048 | cornichon homolog 3 (Drosophila) |
| HMOX1 | 0.15 | 0.83 | 0.30 | - | heme oxygenase (decycling) 1 |
| Q9H8Q9_HUMAN | -0.60 | 0.83 | -0.21 | - | - |
| - | -0.06 | 0.83 | -0.54 | - | hypothetical LOC340477 |
| - | -0.01 | 0.83 | -0.06 | AK057119 | - |
| - | 0.34 | 0.83 | 0.15 | - | similar to hypothetical protein FLJ10408 |
| - | 0.00 | 0.83 | -0.12 | AK093735 | - |
| NUDT15 | 0.41 | 0.83 | 0.19 | AK001818 | nudix (nucleoside diphosphate linked moiety X)-type motif 15 |
| NUPR1_HUMAN | -0.11 | 0.83 | -0.37 | AF069073 | p8 protein (candidate of metastasis 1) |
| - | 0.09 | 0.83 | -0.19 | - | - |
| NP_689911.1 | 0.30 | 0.83 | 0.13 | AK095696 | hypothetical protein FLJ38377 |
| STK32A | 0.39 | 0.82 | 0.46 | AK094580 | - |
| - | 0.15 | 0.82 | 0.08 | AK001013 | - |
| UBASH3A | -0.26 | 0.82 | -0.07 | BC028138 | ubiquitin associated and SH3 domain containing, A |
| RP1 | -0.44 | 0.82 | 0.40 | AF141021 | retinitis pigmentosa 1 (autosomal dominant) |
| - | -0.01 | 0.82 | 0.25 | - | - |
| NP_008831.2 | 0.00 | 0.82 | 0.48 | BC069427 | interferon, alpha 13 |
| TRGV9 | -0.36 | 0.82 | -0.10 | AK056843 | - |
| RNS11_HUMAN | -0.16 | 0.82 | -0.30 | BX161458 | ribonuclease, RNase A family, 11 (non-active) |
| SMARCA2 | -0.11 | 0.82 | -0.10 | - | - |
| - | 0.08 | 0.82 | 0.27 | - | - |
| SCN1A | -0.47 | 0.82 | -1.21 | AY043484 | sodium channel, voltage-gated, type I, alpha |
| FAM12B | 0.08 | 0.82 | -0.26 | AY358865 | family with sequence similarity 12, member B (epididymal) |
| XP_379939.1 | -0.86 | 0.82 | -1.54 | AK097648 | similar to RIKEN cDNA 1700016G05 |
| - | -0.18 | 0.82 | -0.26 | AK093080 | - |
| NP_997530.1 | -0.32 | 0.82 | -0.93 | BC036746 | similar to fibronectin type 3 and SPRY domain-containing protein |
| CNTF | 0.22 | 0.82 | 0.03 | - | zinc finger protein 91 homolog (mouse) |
| - | -0.11 | 0.82 | 0.33 | - | similar to Rpl7a protein |
| MTBP | -0.61 | 0.81 | 0.44 | BC013136 | Mdm2, transformed 3T3 cell double minute 2, p53 binding protein (mouse) binding protein, 104kDa |
| FXD4B_HUMAN | 0.21 | 0.81 | -0.18 | - | forkhead box protein D4b |
| O15451_HUMAN | 0.11 | 0.81 | 0.45 | BC069058 | proline-, glutamic acid-, leucine-rich protein 1 |
| - | -0.49 | 0.81 | -0.94 | AK093309 | - |
| - | 0.44 | 0.81 | 0.01 | X92518,U28749 | high mobility group AT-hook 2 |
| - | -0.10 | 0.81 | -0.51 | AK094609 | - |
| NP_078841.2 | -0.33 | 0.81 | -0.32 | AK131381 | - |
| Q14179_HUMAN | -0.62 | 0.81 | 0.47 | - | - |
| CSEN | -0.23 | 0.81 | -0.12 | BC012850 | calsenilin, presenilin binding protein, EF hand transcription factor |
| LY75 | 0.42 | 0.81 | 0.19 | AF011333 | lymphocyte antigen 75 |
| - | 0.00 | 0.81 | -0.17 | AK095374 | - |
| C3 | 0.32 | 0.81 | -0.74 | BC063852,K02765 | complement component 3 |
| P2RY8 | 0.02 | 0.81 | -0.53 | BC043610 | purinergic receptor P2Y, G-protein coupled, 8 |
| - | 0.05 | 0.81 | 0.48 | CR593492 | similar to hypothetical protein A630014H24 |
| - | 0.05 | 0.81 | -0.54 | CN413190 | LOC442626 |
| - | 0.38 | 0.80 | 0.33 | AK095367 | - |
| ZNF326 | -0.15 | 0.80 | -0.03 | BC038454 | zinc finger protein 326 |
| C21orf32 | 0.00 | 0.80 | 0.34 | AI224560 | - |
| - | -0.08 | 0.80 | -0.41 | AK098797 | - |
| OR5T3 | 0.50 | 0.80 | 0.22 | - | olfactory receptor, family 5, subfamily T, member 3 |
| PIP5KL1 | -0.21 | 0.80 | -0.11 | - | phosphatidylinositol-4-phosphate 5-kinase-like 1 |
| - | -0.68 | 0.80 | -0.26 | AK093345 | - |
| ZNF544 | -0.66 | 0.80 | 0.27 | AF020591 | zinc finger protein 544 |
| - | 0.30 | 0.80 | 0.35 | - | similar to CG2720-PA |
| - | 0.05 | 0.80 | -0.08 | - | - |
| C1orf46 | -0.05 | 0.79 | 0.45 | AF005082 | chromosome 1 open reading frame 46 |
| SOS2 | 0.32 | 0.79 | -0.69 | L13858 | son of sevenless homolog 2 (Drosophila) |
| Q8NHB6_HUMAN | 0.33 | 0.79 | 0.30 | - | - |
| - | -0.13 | 0.79 | -0.30 | - | hypothetical LOC400781 |
| - | 0.16 | 0.79 | -0.19 | AK096706 | - |
| Q9H7H6_HUMAN | 0.15 | 0.79 | 0.14 | AK024504 | Ser/Thr-like kinase |
| NP_079044.1 | 0.48 | 0.79 | 0.24 | - | hypothetical protein FLJ12057 |
| Q9UDD7_HUMAN | 0.04 | 0.79 | -0.59 | - | - |
| Q8N9J7_HUMAN | -0.07 | 0.79 | 0.06 | BC047423,BC078664 | - |
| - | -0.09 | 0.79 | 0.20 | - | similar to ribosomal protein L6 |
| - | -0.06 | 0.79 | -0.17 | - | LOC440919 |
| GPR150 | 0.00 | 0.79 | -0.96 | BC030197 | G protein-coupled receptor 150 |
| ARPM2_HUMAN | -0.18 | 0.79 | 0.26 | - | actin-related protein M2 |
| STX6 | -0.78 | 0.79 | 0.39 | AJ002078,BC039118 | syntaxin 6 |
| - | -0.08 | 0.79 | -0.25 | AK131230 | FLJ16139 protein |
| - | -0.17 | 0.79 | -0.17 | BM547222 | - |
| - | -0.13 | 0.79 | -0.06 | AK058067 | hypothetical protein LOC286114 |
| - | 0.45 | 0.79 | 0.46 | - | similar to Cathepsin L precursor (Major excreted protein) (MEP) |
| NP_997249.1 | -0.12 | 0.79 | -1.22 | AK126048 | FLJ44060 protein |
| - | 0.15 | 0.79 | -0.17 | AK025090 | - |
| LY86 | 0.00 | 0.79 | -0.24 | AF057178,BF794909 | lymphocyte antigen 86 |
| - | -0.21 | 0.79 | -0.46 | - | hypothetical protein MGC35440 |
| Q96I56_HUMAN | 0.28 | 0.78 | 0.00 | BC072390,AF316855,BC054509 | hypothetical protein FLJ90297 |
| CYC_HUMAN | 0.18 | 0.78 | -0.67 | AK056360,AL713681 | cytochrome c, somatic |
| ZNF225 | 0.42 | 0.78 | 0.11 | CR615158 | zinc finger protein 225 |
| NP_079117.2 | -0.06 | 0.78 | 0.20 | - | - |
| - | 0.39 | 0.78 | 0.27 | AK022625,AK094973 | hypothetical protein LOC92270 |
| Q7M4M3_HUMAN | 0.00 | 0.78 | -0.18 | - | - |
| - | -0.47 | 0.78 | 0.07 | AK057274 | - |
| - | 0.23 | 0.78 | 0.09 | - | similar to Ubiquitin carboxyl-terminal hydrolase 7 (Ubiquitin thiolesterase 7) (Ubiquitin-specific processing protease 7) (Deubiquitinating enzyme 7) (Herpesvirus associated ubiquitin-specific protease) |
| FBXW10 | 0.34 | 0.78 | 0.08 | CF593769 | - |
| CLTB | 0.10 | 0.78 | 0.38 | - | clathrin, light polypeptide (Lcb) |
| Q5TEE7_HUMAN | 0.26 | 0.78 | 0.19 | BX641056,AK001138 | hypothetical protein FLJ10276 |
| - | -1.14 | 0.78 | -1.17 | Y07512 | protein kinase, cGMP-dependent, type I |
| PRDM13 | 0.27 | 0.78 | -0.09 | AY004253 | PR domain containing 13 |
| - | -0.53 | 0.78 | -1.30 | - | similar to 40S ribosomal protein SA (p40) (34/67 kDa laminin receptor) (Colon carcinoma laminin-binding protein) (NEM/1CHD4) (Multidrug resistance-associated protein MGr1-Ag) |
| NP_775959.1 | 0.00 | 0.77 | 0.44 | - | hypothetical protein FLJ39630 |
| - | 0.19 | 0.77 | -0.07 | - | similar to hypothetical protein SB153 isoform 2 |
| - | -0.14 | 0.77 | -0.11 | AL117552 | - |
| NP_115613.1 | 0.06 | 0.77 | -0.54 | - | hypothetical protein FLJ23356 |
| TSGA10IP | -0.28 | 0.77 | 0.23 | AK057442 | testis specific, 10 interacting protein |
| CDK5R2 | 0.00 | 0.77 | 0.08 | U34051,BC041771 | cyclin-dependent kinase 5, regulatory subunit 2 (p39) |
| - | 0.31 | 0.77 | 0.41 | - | similar to RIKEN cDNA C230094B15 |
| RCOR3 | 0.31 | 0.77 | 0.14 | AB037764 | REST corepressor 3 |
| ENDOGL1 | 0.20 | 0.77 | 0.14 | AK023235 | - |
| Q8IUV1_HUMAN | 0.35 | 0.77 | -0.07 | - | signal-induced proliferation-associated 1 like 3 |
| - | 0.34 | 0.77 | 0.23 | - | similar to 40S ribosomal protein S16 |
| - | -0.13 | 0.77 | 0.22 | AK090482 | - |
| NP_954857.1 | -0.45 | 0.77 | 0.43 | BC051842 | multidrug resistance-related protein |
| MNAB | 0.31 | 0.77 | 0.32 | AK000308 | membrane associated DNA binding protein |
| - | -0.12 | 0.77 | -0.22 | - | - |
| - | -0.12 | 0.77 | 0.34 | - | LOC440966 |
| TFEC | 0.18 | 0.76 | 0.16 | D43945,BX538223 | transcription factor EC |
| PDCD1 | 0.10 | 0.76 | 0.00 | U64863 | programmed cell death 1 |
| OR4C13 | -0.15 | 0.76 | 0.21 | - | olfactory receptor, family 4, subfamily C, member 13 |
| - | 0.05 | 0.76 | 0.26 | Y00318,AK122686 | I factor (complement) |
| TCP10 | 0.00 | 0.76 | 0.29 | - | similar to T-complex protein 10A homolog |
| SLC2A14 | 0.07 | 0.76 | 0.42 | AK126026 | - |
| NP_653298.1 | 0.18 | 0.76 | -1.06 | AK056960 | hypothetical protein BC017397 |
| NR3C2 | -0.60 | 0.76 | -0.06 | M16801 | nuclear receptor subfamily 3, group C, member 2 |
| - | -0.26 | 0.76 | 0.42 | - | protocadherin 1 (cadherin-like 1) |
| - | 0.01 | 0.76 | 0.19 | AK096179 | - |
| ura3 | 0.01 | 0.76 | 0.36 | AY428060 | - |
| TCTA | 0.00 | 0.76 | 0.44 | AK000824,L41143 | T-cell leukemia translocation altered gene |
| INPP5F | -0.06 | 0.76 | -0.38 | AB023183 | inositol polyphosphate-5-phosphatase F |
| - | 0.06 | 0.76 | 0.42 | - | ficolin (collagen/fibrinogen domain containing lectin) 2 (hucolin) |
| - | 0.36 | 0.76 | 0.46 | AK091791 | - |
| Q75L30_HUMAN | 0.21 | 0.76 | 0.17 | AL136837 | - |
| - | 0.07 | 0.76 | -0.06 | - | - |
| - | 0.20 | 0.76 | -0.07 | AK094398 | - |
| SLC1A3 | -0.27 | 0.76 | 0.19 | D26443,BC037310 | solute carrier family 1 (glial high affinity glutamate transporter), member 3 |
| AIG1 | -0.37 | 0.76 | 0.31 | - | androgen-induced 1 |
| - | -0.02 | 0.76 | 0.17 | - | similar to keratin 8; cytokeratin 8; keratin, type II cytoskeletal 8 |
| - | -0.19 | 0.76 | -0.64 | AK054938 | - |
| LIRA2_HUMAN | 0.11 | 0.75 | 0.07 | CB046242,BC027916,AF025531 | leukocyte immunoglobulin-like receptor, subfamily A (with TM domain), member 2 |
| BHMT | -0.02 | 0.75 | 0.12 | U50929,BC012616 | betaine-homocysteine methyltransferase |
| C20orf67 | 0.21 | 0.75 | -0.57 | AK056553 | chromosome 20 open reading frame 67 |
| OR52B6 | -0.07 | 0.75 | -0.89 | - | - |
| CES1 | -0.40 | 0.75 | 0.47 | BC012418,L07765 | carboxylesterase 1 (monocyte/macrophage serine esterase 1) |
| - | 0.20 | 0.75 | -0.19 | - | similar to AER176Wp |
| NM_030623.1 | 0.49 | 0.75 | 0.27 | AB051465 | - |
| - | 0.19 | 0.75 | -0.82 | X81005,AK056727 | - |
| - | 0.14 | 0.75 | 0.41 | AK098301 | - |
| ABCG8 | -0.01 | 0.75 | -0.19 | AF324494 | ATP-binding cassette, sub-family G (WHITE), member 8 (sterolin 2) |
| - | -0.41 | 0.75 | 0.32 | - | - |
| - | 0.43 | 0.75 | -0.43 | AK126863 | hypothetical gene supported by AK126863 |
| Q8N4B0_HUMAN | 0.28 | 0.75 | -0.35 | BC034822 | - |
| - | -0.42 | 0.75 | -0.25 | AK097878 | - |
| CNTNAP1 | -0.54 | 0.75 | 0.29 | U87223 | contactin associated protein 1 |
| ATP10B | 0.13 | 0.74 | 0.38 | - | - |
| - | 0.00 | 0.74 | 0.29 | BX116511 | chromosome 21 open reading frame 54 |
| - | 0.14 | 0.74 | -0.07 | AL117595 | - |
| ACSL4 | 0.40 | 0.74 | -0.02 | AF030555 | acyl-CoA synthetase long-chain family member 4 |
| PRR7 | 0.33 | 0.74 | 0.43 | - | proline rich 7 (synaptic) |
| MARCH4 | -0.15 | 0.74 | 0.27 | AB037820 | membrane-associated ring finger (C3HC4) 4 |
| CDH22 | 0.39 | 0.74 | 0.43 | AF035300 | cadherin-like 22 |
| - | -0.06 | 0.74 | -0.33 | - | - |
| - | -0.14 | 0.74 | 0.04 | AB011138,AB051358 | ATPase, Class V, type 10A |
| - | 0.45 | 0.74 | -0.13 | - | - |
| LRRN5 | -0.17 | 0.74 | -1.48 | AF030435 | leucine rich repeat neuronal 5 |
| LPHN3_HUMAN | -0.01 | 0.74 | 0.32 | - | latrophilin 3 |
| NP_060369.1 | 0.32 | 0.74 | -0.20 | AK000614 | hypothetical protein FLJ20607 |
| Q8TB00_HUMAN | -0.90 | 0.74 | -0.04 | - | - |
| - | -0.38 | 0.74 | 0.33 | AK023682 | - |
| SIPA1L3 | -0.10 | 0.74 | -0.60 | AB011117 | signal-induced proliferation-associated 1 like 3 |
| NAGS | 0.21 | 0.74 | 0.15 | AK074407 | N-acetylglutamate synthase |
| SNX13 | 0.09 | 0.74 | 0.34 | - | - |
| MDGA1 | 0.20 | 0.74 | -0.43 | AK055376,AF478693 | MAM domain containing glycosylphosphatidylinositol anchor 1 |
| - | -0.19 | 0.74 | -0.11 | - | - |
| - | 0.15 | 0.74 | -0.19 | - | hypothetical LOC388416 |
| - | 0.19 | 0.74 | 0.11 | - | similar to Hkr1p; YDR420W; CAI: 0.10 |
| C6orf166 | 0.23 | 0.73 | -0.41 | BC000764,CR599380 | chromosome 6 open reading frame 166 |
| KIAA0826 | -0.46 | 0.73 | 0.34 | AL833170 | KIAA0826 |
| SCD4 | -0.31 | 0.73 | 0.48 | - | stearoyl-CoA desaturase 4 |
| ADRB1 | 0.35 | 0.73 | -0.28 | J03019 | adrenergic, beta-1-, receptor |
| PLD2 | 0.37 | 0.73 | 0.43 | - | phospholipase D2 |
| ADIPOR1 | 0.01 | 0.73 | 0.06 | AK001484,AK124455 | adiponectin receptor 1 |
| PROK1 | -0.73 | 0.73 | -1.50 | AY029225 | prokineticin 1 |
| CXCL6 | 0.25 | 0.73 | -0.45 | U81234 | chemokine (C-X-C motif) ligand 6 (granulocyte chemotactic protein 2) |
| SPIB | -0.95 | 0.73 | 0.00 | X66079 | Spi-B transcription factor (Spi-1/PU.1 related) |
| OR2M4_HUMAN | -0.34 | 0.73 | 0.12 | X89666 | olfactory receptor, family 2, subfamily M, member 4 |
| NP_848544.1 | 0.31 | 0.73 | 0.38 | AY037555 | tuberoinfundibular 39 residue protein precursor |
| Q8N1Y8_HUMAN | 0.28 | 0.73 | 0.29 | AK094546 | - |
| NP_938016.1 | -0.48 | 0.73 | -0.10 | AK095399,BC008810 | - |
| Q8N0T2_HUMAN | -0.90 | 0.73 | 0.26 | - | - |
| - | 0.07 | 0.73 | -1.29 | BC018787 | - |
| Q9P2H4_HUMAN | 0.04 | 0.73 | 0.19 | AB037794 | - |
| GLT25D2 | -0.64 | 0.73 | -0.22 | AB011156,AF288389 | glycosyltransferase 25 domain containing 2 |
| - | -0.01 | 0.72 | -0.11 | AL122113 | - |
| LAMB2 | 0.27 | 0.72 | 0.22 | S77512 | laminin, beta 2 (laminin S) |
| OR2W1 | 0.33 | 0.72 | 0.06 | - | olfactory receptor, family 2, subfamily W, member 1 |
| - | -0.38 | 0.72 | 0.22 | AK055009 | - |
| XM_496351.1 | -0.13 | 0.72 | -0.19 | AK090414 | hypothetical protein MGC12760 |
| BRSK1 | 0.00 | 0.72 | -0.18 | AB058714,AF479827 | BR serine/threonine kinase 1 |
| C14orf115 | -0.04 | 0.72 | -0.05 | BC053325,AK001673 | chromosome 14 open reading frame 115 |
| - | 0.20 | 0.72 | 0.28 | AK027555 | - |
| - | -0.49 | 0.72 | -1.82 | AK094934 | - |
| XP_371714.1 | 0.05 | 0.72 | 0.11 | - | tripartite motif-containing 61 |
| USH1C | 0.15 | 0.72 | 0.43 | AB006955 | Usher syndrome 1C (autosomal recessive, severe) |
| EPSTI1 | -0.20 | 0.72 | -0.49 | AL831953 | epithelial stromal interaction 1 (breast) |
| NP_115948.3 | 0.34 | 0.72 | -0.74 | AF333335 | kinesin protein |
| - | -0.08 | 0.72 | 0.05 | AK057070 | - |
| - | -0.28 | 0.72 | -0.06 | AK094212 | - |
| - | -0.16 | 0.72 | -2.49 | - | - |
| CHRND | -0.61 | 0.72 | -1.07 | CR623061,X55019 | cholinergic receptor, nicotinic, delta polypeptide |
| ABCA1 | 0.05 | 0.72 | 0.45 | - | - |
| NM_152312.2 | -0.27 | 0.72 | -0.89 | BC037291,AK055829 | glycosyltransferase-like 1B |
| - | 0.41 | 0.72 | 0.45 | - | - |
| RCV1 | -0.09 | 0.71 | 0.35 | S43855 | recoverin |
| PCDHB7 | -0.06 | 0.71 | -0.07 | AF217750 | protocadherin beta 7 |
| NP_835466.1 | -0.22 | 0.71 | -0.25 | BC035810 | high density lipoprotein-binding protein |
| - | 0.10 | 0.71 | 0.42 | L21998 | mucin 2, intestinal/tracheal |
| S100A7L1 | -0.21 | 0.71 | -1.06 | AY189118,M86757,BG680212 | S100 calcium binding protein A7-like 1 |
| - | 0.45 | 0.71 | -0.83 | - | hypothetical gene supported by AK094824 |
| - | -0.07 | 0.71 | 0.14 | AK094239 | - |
| OR2T29 | 0.34 | 0.71 | -0.34 | - | olfactory receptor, family 2, subfamily T, member 5 |
| - | 0.00 | 0.71 | 0.27 | - | - |
| - | 0.46 | 0.71 | -1.21 | - | similar to Hypothetical protein CBG01089 |
| C14orf149 | 0.14 | 0.71 | 0.27 | AK058165 | chromosome 14 open reading frame 149 |
| - | 0.48 | 0.71 | 0.13 | - | - |
| ASB1 | 0.08 | 0.71 | -1.41 | AB032972 | ankyrin repeat and SOCS box-containing 1 |
| XP_495896.1 | 0.17 | 0.71 | 0.09 | - | - |
| - | 0.47 | 0.71 | -3.10 | AK025206 | - |
| Q5T0Z8_HUMAN | -0.15 | 0.71 | 0.22 | - | - |
| - | -0.62 | 0.71 | 0.21 | AK093299 | - |
| SPATS1 | 0.49 | 0.70 | 0.43 | AK058171 | spermatogenesis associated, serine-rich 1 |
| - | -1.15 | 0.70 | 0.06 | BC050686 | DC12 protein |
| - | 0.04 | 0.70 | 0.10 | - | keratin associated protein |
| GPLD1 | -0.32 | 0.70 | -0.17 | L11702 | glycosylphosphatidylinositol specific phospholipase D1 |
| NP_001008949.1 | -0.36 | 0.70 | -0.11 | BC034503 | KIAA1754-like |
| RSAD2 | -0.43 | 0.70 | 0.33 | - | radical S-adenosyl methionine domain containing 2 |
| HA22_HUMAN | 0.48 | 0.70 | 0.09 | BC051832 | major histocompatibility complex, class II, DQ alpha 1 |
| KCTD9_HUMAN | 0.23 | 0.70 | 0.32 | AL117436 | potassium channel tetramerisation domain containing 9 |
| OR6C4 | -0.16 | 0.70 | -0.72 | - | olfactory receptor, family 6, subfamily C, member 4 |
| NP_060631.2 | 0.00 | 0.70 | 0.11 | BC020977,AL512694,AK055452,AK001493 | NAD synthetase 1 |
| AICDA | -0.09 | 0.70 | 0.38 | AB040431 | activation-induced cytidine deaminase |
| - | -0.28 | 0.70 | 0.43 | - | - |
| SYN3 | -0.62 | 0.70 | 0.30 | AF046873 | synapsin III |
| DNMT3L | -0.79 | 0.70 | -0.34 | AF194032 | DNA (cytosine-5-)-methyltransferase 3-like |
| Q8N1N1_HUMAN | 0.42 | 0.70 | 0.46 | - | - |
| NP_777585.1 | 0.17 | 0.70 | 0.47 | BC064430 | hypothetical protein LOC205251 |
| OR1S2 | -0.20 | 0.70 | 0.29 | - | olfactory receptor, family 1, subfamily S, member 2 |
| IL17R | -0.37 | 0.70 | -1.16 | U58917,BC011624 | interleukin 17 receptor |
| - | 0.37 | 0.70 | -0.59 | AL117638 | - |
| NP_001005851.1 | 0.14 | 0.69 | 0.25 | AK127063 | hypothetical BC331191_1 |
| ZNF364 | -0.02 | 0.69 | 0.10 | AL079314,AF419857 | zinc finger protein 364 |
| NP_001010873.1 | -0.34 | 0.69 | 0.49 | BU655765 | benzodiazapine receptor (peripheral)-like 1 |
| LMO2 | -0.14 | 0.69 | 0.39 | X61118 | LIM domain only 2 (rhombotin-like 1) |
| FBXO17 | -0.13 | 0.69 | 0.49 | AF386743 | F-box protein 17 |
| PLCD1 | -0.02 | 0.69 | -1.08 | BX647927,U09117 | phospholipase C, delta 1 |
| UGT2B11 | -0.02 | 0.69 | 0.47 | AF016492,AF177272,BG195984 | UDP glycosyltransferase 2 family, polypeptide B11 |
| - | -0.24 | 0.69 | -0.82 | - | - |
| - | -0.17 | 0.69 | 0.37 | AK026881 | - |
| - | 0.35 | 0.69 | 0.41 | - | - |
| - | -0.12 | 0.69 | -0.09 | AK021616 | - |
| Q8IYK1_HUMAN | -0.27 | 0.69 | 0.11 | - | hypothetical protein FLJ22405 |
| - | 0.30 | 0.69 | -0.24 | AK027294 | - |
| - | -0.48 | 0.69 | 0.06 | AK056852 | - |
| NP_620711.2 | 0.06 | 0.69 | 0.16 | BC035009,AL137311 | delta-notch-like EGF repeat-containing transmembrane |
| - | -0.01 | 0.69 | 0.35 | AK001120 | - |
| - | 0.36 | 0.69 | 0.07 | AK025151 | - |
| Q96DP8_HUMAN | -0.53 | 0.69 | 0.12 | - | - |
| FUT1 | 0.00 | 0.69 | -0.16 | M35531 | fucosyltransferase 1 (galactoside 2-alpha-L-fucosyltransferase) |
| KIAA1622 | 0.06 | 0.68 | -1.01 | AB046842 | KIAA1622 |
| CORO2B | 0.34 | 0.68 | -0.49 | AK124711,AB023142 | coronin, actin binding protein, 2B |
| TTC10 | 0.26 | 0.68 | -0.01 | AK126668,U20362 | tetratricopeptide repeat domain 10 |
| Q96D43_HUMAN | -0.06 | 0.68 | -0.08 | AK001103,AK123631 | hypothetical protein FLJ10241 |
| C14orf172 | 0.08 | 0.68 | 0.00 | BC016033 | - |
| - | -0.19 | 0.68 | 0.12 | AF030165 | fascin homolog 2, actin-bundling protein, retinal (Strongylocentrotus purpuratus) |
| - | 0.05 | 0.68 | -0.15 | AK095800,BC008055 | hypothetical protein LOC284798 |
| STAU | -0.16 | 0.68 | -0.42 | AF061939 | staufen, RNA binding protein (Drosophila) |
| SEC14L3 | 0.46 | 0.68 | 0.02 | - | SEC14-like 3 (S. cerevisiae) |
| GCHFR | -0.46 | 0.68 | 0.47 | - | GTP cyclohydrolase I feedback regulator |
| CTF1 | -0.38 | 0.68 | 0.11 | U43030,BC036787 | cardiotrophin 1 |
| ARHGDIB | 0.25 | 0.68 | 0.07 | L20688 | Rho GDP dissociation inhibitor (GDI) beta |
| RFT1 | -0.02 | 0.68 | -0.18 | BC043595 | RFT1 homolog (S. cerevisiae) |
| - | -0.28 | 0.68 | -0.22 | AK125998 | hypothetical protein MGC13005 |
| - | 0.33 | 0.68 | 0.19 | - | LOC440064 |
| OR1I1_HUMAN | -0.47 | 0.68 | 0.27 | - | olfactory receptor, family 1, subfamily I, member 1 |
| NP_853650.1 | -0.17 | 0.68 | 0.15 | - | keratin associated protein 21-1 |
| - | -0.35 | 0.68 | 0.24 | CN285855 | LOC440071 |
| - | 0.36 | 0.68 | -0.25 | BC019830 | - |
| GPR156 | -0.38 | 0.68 | -0.33 | AF488739 | G protein-coupled receptor 156 |
| Q9BYA7_HUMAN | -0.14 | 0.68 | -0.06 | - | - |
| ERMAP | 0.47 | 0.68 | -0.39 | AK056138 | - |
| EBAG9 | -0.20 | 0.68 | -0.28 | AB007619,BC022506 | estrogen receptor binding site associated, antigen, 9 |
| AGTR1 | 0.42 | 0.68 | 0.26 | BC068494 | angiotensin II receptor, type 1 |
| CYP7B1 | 0.21 | 0.68 | 0.36 | AF127090 | cytochrome P450, family 7, subfamily B, polypeptide 1 |
| TRPA1 | -0.58 | 0.68 | 0.23 | Y10601 | transient receptor potential cation channel, subfamily A, member 1 |
| CALML3 | 0.39 | 0.68 | 0.17 | BC031889 | calmodulin-like 3 |
| SPATS2 | -0.18 | 0.68 | -0.51 | AL833614 | spermatogenesis associated, serine-rich 2 |
| OSR2 | 0.36 | 0.68 | -0.64 | AK074518 | odd-skipped related 2 (Drosophila) |
| PPFIA2 | -1.50 | 0.67 | -0.09 | AF034799,AK126971 | protein tyrosine phosphatase, receptor type, f polypeptide (PTPRF), interacting protein (liprin), alpha 2 |
| - | 0.44 | 0.67 | -0.10 | AL832189 | - |
| SAV1 | 0.11 | 0.67 | 0.24 | AL833378 | salvador homolog 1 (Drosophila) |
| - | 0.33 | 0.67 | 0.43 | - | hypothetical gene supported by BC040831 |
| - | 0.17 | 0.67 | 0.19 | - | - |
| Q86XT6_HUMAN | -0.35 | 0.67 | 0.48 | - | - |
| - | -0.51 | 0.67 | -1.02 | - | - |
| - | 0.11 | 0.67 | -0.19 | BC038108 | hypothetical LOC403313 |
| NP_001001677.1 | 0.29 | 0.67 | -0.49 | AK128177 | FLJ46300 protein |
| NP_659451.1 | -0.17 | 0.67 | -0.55 | AK057477,AK127394 | hypothetical protein FLJ32915 |
| - | -0.78 | 0.67 | -0.05 | - | olfactory receptor, family 2, subfamily J, member 2 |
| RFWD3 | 0.25 | 0.67 | 0.05 | BC002574 | - |
| CEACAM7 | 0.15 | 0.67 | -0.41 | X98311 | carcinoembryonic antigen-related cell adhesion molecule 7 |
| TRIO | 0.35 | 0.67 | 0.36 | - | - |
| NP_859057.3 | -0.14 | 0.67 | -0.50 | AK123087 | zinc finger, CSL domain containing 3 |
| MRGRG_HUMAN | -0.21 | 0.67 | 0.44 | - | - |
| C8orf14 | 0.30 | 0.67 | 0.06 | AJ291678 | - |
| NP_775737.1 | 0.04 | 0.67 | 0.46 | AK001064 | hypothetical protein DKFZp434P055 |
| USP12 | 0.41 | 0.67 | 0.19 | AF022789 | ubiquitin specific protease 12 |
| - | 0.45 | 0.67 | -0.35 | - | hypothetical LOC153959 |
| PHKG1 | -0.03 | 0.66 | 0.38 | X80590,BC051327 | phosphorylase kinase, gamma 1 (muscle) |
| - | -0.14 | 0.66 | 0.36 | - | similar to KIAA1110 protein |
| - | -0.16 | 0.66 | -0.30 | AK091086 | - |
| - | -0.04 | 0.66 | -0.86 | AK093594 | - |
| Q96LN9_HUMAN | 0.02 | 0.66 | -0.37 | BC057843,AL137484 | - |
| KCNB2 | 0.00 | 0.66 | -0.02 | - | potassium voltage-gated channel, Shab-related subfamily, member 2 |
| - | -0.47 | 0.66 | 0.39 | BX117800 | hypothetical LOC338914 |
| - | 0.42 | 0.66 | -0.35 | - | hypothetical LOC388235 |
| - | 0.28 | 0.66 | 0.23 | AK126782,AK055338 | tripartite motif-containing 67 |
| NP_775923.1 | 0.11 | 0.66 | -0.70 | BC031410 | hypothetical protein MGC34824 |
| TXNDC2 | -0.31 | 0.66 | -0.11 | - | thioredoxin domain containing 2 (spermatozoa) |
| - | 0.43 | 0.66 | 0.10 | - | similar to Tesp1 protein |
| - | 0.48 | 0.66 | -0.26 | - | - |
| ZNF530 | 0.23 | 0.66 | 0.50 | AB040941 | zinc finger protein 530 |
| NP_789786.1 | -0.14 | 0.66 | -0.19 | AL832658 | Kenae |
| - | 0.22 | 0.66 | -0.69 | - | LOC440665 |
| - | 0.11 | 0.66 | -0.32 | - | solute carrier family 6 (neurotransmitter transporter), member 19 |
| PBEF1 | 0.09 | 0.66 | 0.23 | U02020 | pre-B-cell colony enhancing factor 1 |
| PGLYRP1 | 0.06 | 0.66 | -0.10 | AF242517,AF076483 | peptidoglycan recognition protein 1 |
| XP_171094.1 | -0.21 | 0.66 | 0.18 | AK094270 | similar to eukaryotic translation initiation factor eIF4E-1 |
| XP_376334.1 | 0.41 | 0.66 | 0.47 | AK125951 | similar to hypothetical protein |
| FAM47B | 0.43 | 0.66 | 0.27 | BC035026,AK125992 | family with sequence similarity 47, member B |
| - | -0.01 | 0.66 | -0.72 | - | LOC440761 |
| ELF5 | 0.00 | 0.66 | 0.18 | AF115402 | E74-like factor 5 (ets domain transcription factor) |
| UNC13A | 0.00 | 0.66 | -0.35 | AB028955 | - |
| BT2A1_HUMAN | 0.05 | 0.66 | -0.02 | U90543,BC016661 | butyrophilin, subfamily 2, member A1 |
| SEPP1 | 0.37 | 0.66 | -0.88 | BC030009,Z11793 | selenoprotein P, plasma, 1 |
| - | 0.19 | 0.66 | -1.13 | - | hypothetical LOC388338 |
| - | -0.01 | 0.66 | 0.03 | - | hypothetical LOC389028 |
| - | 0.48 | 0.66 | 0.37 | AK002005 | - |
| C14orf160 | 0.21 | 0.65 | -0.32 | AK022680 | chromosome 14 open reading frame 160 |
| L3MBTL2 | 0.36 | 0.65 | 0.21 | - | - |
| RASA1 | -0.23 | 0.65 | 0.14 | M23379 | RAS p21 protein activator (GTPase activating protein) 1 |
| BAT8 | 0.20 | 0.65 | 0.00 | X69838,BC009351 | HLA-B associated transcript 8 |
| GRIA3 | -0.51 | 0.65 | -0.10 | U10301 | glutamate receptor, ionotrophic, AMPA 3 |
| ELN | -0.04 | 0.65 | 0.04 | BX537939 | elastin (supravalvular aortic stenosis, Williams-Beuren syndrome) |
| CYP11B1 | 0.48 | 0.65 | 0.36 | AK094090 | - |
| SNX26 | 0.40 | 0.65 | 0.46 | - | - |
| CACNA1G | 0.41 | 0.65 | -0.70 | AF134986 | - |
| NP_872389.1 | 0.18 | 0.65 | 0.27 | AK054844 | similar to hypothetical protein FLJ38374 |
| - | 0.17 | 0.65 | 0.17 | - | - |
| - | 0.36 | 0.65 | 0.00 | - | similar to cSH-PTP2 |
| MARCO | -0.07 | 0.65 | -0.24 | BC016004,AF035819 | macrophage receptor with collagenous structure |
| Q9H3C3_HUMAN | -0.03 | 0.65 | 0.22 | - | - |
| EFNA5 | 0.23 | 0.65 | -0.96 | U26403 | ephrin-A5 |
| HSPA12B | -0.02 | 0.65 | 0.38 | AK056712 | heat shock 70kD protein 12B |
| ZWINTAS | 0.05 | 0.65 | 0.37 | X98261 | - |
| MYOG | 0.09 | 0.65 | -1.86 | BC053899 | myogenin (myogenic factor 4) |
| C1orf21 | -0.06 | 0.65 | -0.99 | AF312864 | chromosome 1 open reading frame 21 |
| ZBTB16 | 0.10 | 0.65 | 0.06 | Z19002,BX648973 | zinc finger and BTB domain containing 16 |
| MBD2 | -0.15 | 0.65 | 0.37 | - | methyl-CpG binding domain protein 2 |
| VDP_HUMAN | 0.02 | 0.65 | 0.20 | AL832010,D86326 | vesicle docking protein p115 |
| NP_778238.1 | -0.14 | 0.65 | -1.00 | AJ508776 | keratin 6 irs3 |
| PTGIS | 0.23 | 0.65 | 0.48 | - | prostaglandin I2 (prostacyclin) synthase |
| - | -0.01 | 0.65 | -0.11 | AK124409 | FLJ42418 protein |
| XP_351855.1 | 0.08 | 0.64 | 0.25 | AK074396 | similar to cDNA sequence BC034076 |
| OPRL1 | 0.20 | 0.64 | -0.41 | BC038433,U30185 | opiate receptor-like 1 |
| - | -0.01 | 0.64 | -0.13 | BU561092 | hypothetical LOC389707 |
| CENTB5 | 0.47 | 0.64 | 0.49 | AB051503 | centaurin, beta 5 |
| ANGPTL7 | 0.31 | 0.64 | 0.40 | BC001881 | angiopoietin-like 7 |
| TMEM28 | -0.06 | 0.64 | 0.03 | AF087142 | transmembrane protein 28 |
| - | 0.00 | 0.64 | 0.28 | - | SH3-binding domain kinase 1 |
| MLLT2 | -0.11 | 0.64 | -0.73 | L13773 | myeloid/lymphoid or mixed-lineage leukemia (trithorax homolog, Drosophila); translocated to, 2 |
| Q8N8T8_HUMAN | -0.65 | 0.64 | 0.34 | - | - |
| Q86XB5_HUMAN | -0.25 | 0.64 | -0.18 | BC031107 | hypothetical protein LOC136288 |
| Q8N2Q0_HUMAN | 0.18 | 0.64 | -0.04 | AK126577 | hypothetical protein LOC144347 |
| NODAL | 0.26 | 0.64 | -0.08 | BC033585 | nodal homolog (mouse) |
| GLE1L | -0.05 | 0.64 | -0.44 | AK074801 | GLE1 RNA export mediator-like (yeast) |
| XP_496134.1 | 0.37 | 0.64 | -0.13 | - | similar to BTG3 associated nuclear protein isoform b; BANP homolog; SMAR1 homolog |
| - | 0.21 | 0.64 | -0.08 | AK095793,CA503263,X85545 | protein kinase, X-linked |
| - | 0.48 | 0.64 | 0.03 | AK058061 | - |
| - | 0.29 | 0.64 | 0.13 | AK098632 | - |
| SLC6A8 | 0.47 | 0.64 | -0.26 | L31409 | solute carrier family 6 (neurotransmitter transporter, creatine), member 8 |
| - | 0.35 | 0.64 | 0.22 | BC047939 | - |
| - | 0.38 | 0.64 | 0.19 | - | LOC440504 |
| - | 0.28 | 0.64 | 0.08 | AK095330,AK125921 | similar to hypothetical protein A230046P18; cDNA sequence BC055759 |
| NP_079431.1 | -0.38 | 0.64 | -0.17 | - | hypothetical protein FLJ11848 |
| Q8IW70_HUMAN | 0.30 | 0.64 | 0.43 | - | similar to expressed sequence AW125688 |
| XP_293828.4 | -0.31 | 0.64 | -0.20 | BX648737 | similar to hypothetical protein 9630041N07 |
| - | 0.44 | 0.64 | -0.20 | AK092827 | - |
| - | -0.10 | 0.64 | 0.49 | - | protocadherin alpha 2 |
| TNFSF10 | -0.16 | 0.63 | 0.17 | U37518 | tumor necrosis factor (ligand) superfamily, member 10 |
| - | 0.34 | 0.63 | -0.53 | AK026980 | zinc finger protein 37b (KOX 21) |
| SAGE1 | 0.25 | 0.63 | 0.41 | AJ278111 | sarcoma antigen 1 |
| - | -0.06 | 0.63 | -0.04 | AK126569 | hypothetical gene supported by AK126569 |
| NP_689503.1 | 0.46 | 0.63 | -0.35 | BX647383,AK026705 | - |
| OR8H3 | -0.01 | 0.63 | -0.18 | - | olfactory receptor, family 8, subfamily H, member 3 |
| BCL2L11 | -0.02 | 0.63 | -0.84 | AF032457,BC033694 | BCL2-like 11 (apoptosis facilitator) |
| HAPIP | -0.03 | 0.63 | 0.05 | U94190 | huntingtin-associated protein interacting protein (duo) |
| Q8N372_HUMAN | 0.24 | 0.63 | 0.24 | - | - |
| DMRTC1 | -0.05 | 0.63 | -0.13 | BC029799 | DMRT-like family C1 |
| - | -0.14 | 0.63 | -0.76 | AK092055 | - |
| NP_443094.2 | 0.00 | 0.63 | -0.42 | AF545852 | protein kinase substrate MK2S4 |
| FAM3A | 0.49 | 0.63 | 0.45 | - | family with sequence similarity 3, member A |
| NP_112186.2 | 0.19 | 0.63 | -0.14 | AY358766 | bubblegum related protein |
| - | -0.12 | 0.63 | -0.87 | AK093502 | - |
| - | 0.11 | 0.63 | -0.33 | AK094607 | - |
| WHSC1 | -0.19 | 0.63 | 0.23 | AF071594 | Wolf-Hirschhorn syndrome candidate 1 |
| AARSL | 0.34 | 0.63 | 0.30 | AB033096 | alanyl-tRNA synthetase like |
| - | -0.22 | 0.63 | 0.40 | - | similar to 60S ribosomal protein L21 |
| T | 0.39 | 0.63 | -0.75 | AJ001699 | T, brachyury homolog (mouse) |
| GYG2 | -0.05 | 0.63 | 0.21 | U94362 | glycogenin 2 |
| Q6ZS98_HUMAN | 0.18 | 0.63 | -0.08 | AK127610 | putative UST1-like organic anion transporter |
| RTN3 | 0.24 | 0.63 | 0.30 | AY750848 | similar to Reticulon protein 3 (Neuroendocrine-specific protein-like 2) (NSP-like protein II) (NSPLII) |
| PLXND1 | 0.35 | 0.63 | -0.06 | AB014520,AY116661 | plexin D1 |
| IRX3 | -0.32 | 0.63 | -0.02 | AY335943 | iroquois homeobox protein 3 |
| SGPP2 | 0.13 | 0.63 | 0.24 | AK096323 | sphingosine-1-phosphate phosphotase 2 |
| MAML3 | 0.26 | 0.63 | 0.42 | AL359614,AK123604 | mastermind-like 3 (Drosophila) |
| - | -0.10 | 0.63 | -0.15 | - | similar to 27 kDa Golgi SNARE protein (Golgi SNAP receptor complex member 2) (Membrin) |
| - | -1.02 | 0.63 | -0.30 | AK055231 | - |
| - | 0.23 | 0.63 | 0.22 | AK057130 | - |
| - | 0.17 | 0.63 | -0.17 | AK095170 | - |
| O10A1_HUMAN | 0.25 | 0.63 | 0.18 | - | olfactory receptor, family 10, subfamily A, member 2 |
| SLC19A1 | 0.24 | 0.63 | -0.50 | U17566 | solute carrier family 19 (folate transporter), member 1 |
| VENTX2 | -0.28 | 0.63 | -0.01 | AF068006 | VENT-like homeobox 2 |
| - | 0.19 | 0.63 | -0.09 | - | - |
| Q8TEE0_HUMAN | 0.46 | 0.62 | 0.36 | - | - |
| PRDM9 | -0.05 | 0.62 | 0.34 | AF275816 | PR domain containing 9 |
| FETUB | 0.10 | 0.62 | -0.99 | AJ242928 | fetuin B |
| NP_114119.1 | 0.18 | 0.62 | 0.12 | - | chr3 synaptotagmin |
| DHX37_HUMAN | 0.10 | 0.62 | 0.45 | BC037964,AB040950 | DEAH (Asp-Glu-Ala-His) box polypeptide 37 |
| - | -0.47 | 0.62 | 0.11 | - | similar to 2700029M09Rik protein |
| - | 0.13 | 0.62 | 0.36 | - | similar to KIAA0454 protein |
| - | -0.04 | 0.62 | 0.13 | BM552838 | LOC440604 |
| SLC39A9 | 0.11 | 0.62 | -0.21 | AY358687 | solute carrier family 39 (zinc transporter), member 9 |
| SLC8A3 | 0.00 | 0.62 | 0.21 | AF510502 | solute carrier family 8 (sodium-calcium exchanger), member 3 |
| CLK4 | 0.21 | 0.62 | 0.10 | BC063116 | CDC-like kinase 4 |
| - | 0.11 | 0.62 | 0.32 | AY358209 | similar to YPLR6490 |
| NEK1 | 0.40 | 0.62 | 0.42 | - | NIMA (never in mitosis gene a)-related kinase 1 |
| RAD51L1 | 0.27 | 0.62 | -0.20 | BX248766,U92074 | RAD51-like 1 (S. cerevisiae) |
| - | 0.25 | 0.62 | 0.00 | - | similar to 60S ribosomal protein L35 |
| Q6PY97_HUMAN | -0.24 | 0.62 | -0.17 | - | - |
| - | 0.04 | 0.62 | 0.23 | - | similar to Methionine-R-sulfoxide reductase (Selenoprotein X 1) (HSPC270) |
| - | -0.87 | 0.62 | -0.63 | AK095168 | mitsugumin 29 |
| ABCA8 | -0.50 | 0.62 | -0.46 | AB020629 | ATP-binding cassette, sub-family A (ABC1), member 8 |
| Q8TBI2_HUMAN | 0.46 | 0.62 | 0.06 | - | - |
| SIA4A_HUMAN | 0.06 | 0.62 | 0.22 | - | sialyltransferase 4A (beta-galactoside alpha-2,3-sialyltransferase) |
| - | 0.14 | 0.61 | 0.30 | AK055619 | - |
| - | 0.22 | 0.61 | -0.24 | - | - |
| HOXA5 | -0.16 | 0.61 | -1.64 | - | homeo box A5 |
| NKPD1 | 0.01 | 0.61 | 0.41 | AK090919 | NTPase, KAP family P-loop domain containing 1 |
| XP_372769.2 | 0.24 | 0.61 | 0.48 | AW135717 | similar to Group IIC secretory phospholipase A2 precursor (Phosphatidylcholine 2-acylhydrolase GIIC) (GIIC sPLA2) (PLA2-8) (14 kDa phospholipase A2) |
| O60474_HUMAN | -0.19 | 0.61 | 0.28 | - | - |
| MRPL10 | 0.49 | 0.61 | -0.08 | AK127167 | mitochondrial ribosomal protein L10 |
| OGDHL | 0.18 | 0.61 | 0.04 | BC026320,AK001713 | oxoglutarate dehydrogenase-like |
| Q9Y599_HUMAN | 0.28 | 0.61 | 0.33 | BC008496 | NY-REN-41 antigen |
| XP_372668.2 | 0.05 | 0.61 | -0.21 | - | ADP-ribosylation factor-like 12 |
| C9orf43 | 0.25 | 0.61 | 0.24 | BC026884 | chromosome 9 open reading frame 43 |
| NP_919258.1 | -0.27 | 0.61 | -0.01 | BX640966 | hypothetical protein DKFZp686L1814 |
| - | -0.30 | 0.61 | 0.30 | AK027235 | cartilage associated protein |
| CALCR | -0.01 | 0.61 | 0.07 | - | - |
| - | -0.09 | 0.61 | -0.29 | - | similar to Keratin, type I cytoskeletal 18 (Cytokeratin 18) (K18) (CK 18) |
| - | -0.07 | 0.61 | 0.04 | - | LOC441699 |
| - | 0.00 | 0.61 | 0.15 | - | - |
| TPM2 | -0.26 | 0.61 | -0.15 | - | tropomyosin 2 (beta) |
| GPR115 | 0.16 | 0.61 | -0.14 | - | G protein-coupled receptor 115 |
| - | 0.00 | 0.61 | -0.03 | - | hypothetical LOC389185 |
| - | 0.00 | 0.61 | -0.54 | - | hypothetical LOC388790 |
| RALGPS2 | 0.17 | 0.61 | -0.24 | AK001106 | Ral GEF with PH domain and SH3 binding motif 2 |
| SRGAP3 | 0.13 | 0.61 | 0.00 | AF464189 | SLIT-ROBO Rho GTPase activating protein 3 |
| - | 0.16 | 0.61 | -0.55 | BC034039 | kelch-like 21 (Drosophila) |
| ZNF502 | -0.01 | 0.60 | 0.31 | BC028377 | zinc finger protein 502 |
| NP_848565.1 | 0.42 | 0.60 | -0.31 | BC035674 | WD repeat domain 40B |
| PMM2 | -0.19 | 0.60 | 0.07 | U85773 | phosphomannomutase 2 |
| Q9BTX9_HUMAN | 0.28 | 0.60 | 0.09 | - | - |
| MT1E | 0.00 | 0.60 | 0.27 | BC009699 | - |
| TGFBR1 | 0.26 | 0.60 | -0.01 | BC071181,L11695 | transforming growth factor, beta receptor I (activin A receptor type II-like kinase, 53kDa) |
| - | 0.43 | 0.60 | -0.35 | BQ222464 | similar to hypothetical protein FLJ38281 |
| MAL | -0.24 | 0.60 | 0.20 | - | mal, T-cell differentiation protein |
| GNRH1 | 0.49 | 0.60 | -0.95 | X01059,BC067290 | gonadotropin-releasing hormone 1 (luteinizing-releasing hormone) |
| NP_919276.1 | -0.37 | 0.60 | -0.65 | AK128026 | hypothetical protein LOC223075 |
| DNAJC5G | -0.15 | 0.60 | 0.15 | AK097736 | DnaJ (Hsp40) homolog, subfamily C, member 5 gamma |
| GPR119 | -0.32 | 0.60 | 0.13 | AY288416 | G protein-coupled receptor 119 |
| TFCP2L4 | -0.66 | 0.60 | 0.26 | AL137763,BC036890 | transcription factor CP2-like 4 |
| GPR18 | -0.08 | 0.60 | 0.01 | BC066927 | G protein-coupled receptor 18 |
| - | 0.30 | 0.60 | 0.34 | AK057396 | - |
| - | -0.01 | 0.60 | 0.15 | AK091135 | - |
| GALR3 | 0.04 | 0.60 | -0.09 | AF073799 | galanin receptor 3 |
| SEMA7A | -0.13 | 0.60 | -0.82 | AF069493 | sema domain, immunoglobulin domain (Ig), and GPI membrane anchor, (semaphorin) 7A |
| - | -0.77 | 0.60 | -0.36 | - | similar to nucleolus-cytoplasm shuttle phosphoprotein - rat |
| PKP1 | -0.15 | 0.60 | 0.19 | - | plakophilin 1 (ectodermal dysplasia/skin fragility syndrome) |
| SLC7A4 | 0.04 | 0.60 | -1.47 | AJ000730,BC062565 | solute carrier family 7 (cationic amino acid transporter, y+ system), member 4 |
| Q9P195_HUMAN | 0.16 | 0.60 | 0.39 | - | - |
| GPC5 | -0.68 | 0.60 | 0.38 | BC039730 | glypican 5 |
| - | 0.12 | 0.60 | 0.35 | AK094233 | - |
| ZNF561 | -0.45 | 0.60 | -0.28 | AK122974 | zinc finger protein 561 |
| Q9NSU0_HUMAN | 0.00 | 0.60 | 0.09 | AK093461 | myo-inositol 1-phosphate synthase A1 |
| ZNRF1 | -0.37 | 0.60 | 0.28 | - | zinc and ring finger 1 |
| - | 0.42 | 0.60 | 0.37 | BG202200 | LOC440598 |
| RAB11B | -0.23 | 0.60 | 0.13 | BX647356,X79780 | RAB11B, member RAS oncogene family |
| KISS1 | 0.36 | 0.60 | -0.14 | U43527 | KiSS-1 metastasis-suppressor |
| - | 0.12 | 0.60 | 0.22 | AK025311 | hypothetical protein LOC91948 |
| Q8IUY1_HUMAN | 0.42 | 0.59 | 0.00 | BM685850 | secretory protein LOC284013 |
| - | -0.01 | 0.59 | -0.12 | - | hypothetical LOC387763 |
| XP_293123.4 | -0.48 | 0.59 | -0.07 | - | similar to dJ1100H13.4 (putative RhoGAP domain containing protein) |
| HOXB5 | 0.40 | 0.59 | 0.11 | M92299 | homeo box B5 |
| APOM | 0.00 | 0.59 | -0.63 | BG567934 | apolipoprotein M |
| SYTL5 | -0.05 | 0.59 | -0.41 | BX647688 | synaptotagmin-like 5 |
| ATOH7 | -0.06 | 0.59 | -0.03 | BC032621 | atonal homolog 7 (Drosophila) |
| - | -0.31 | 0.59 | 0.03 | AL137360 | - |
| TNFL6_HUMAN | 0.14 | 0.59 | -0.30 | X89102,D38122 | Fas ligand (TNF superfamily, member 6) |
| NP_919267.1 | -0.38 | 0.59 | -0.15 | BC041379,BC017881 | hypothetical protein BC017881 |
| OR6C2 | 0.17 | 0.59 | 0.00 | - | olfactory receptor, family 6, subfamily C, member 2 |
| Q96DC5_HUMAN | 0.05 | 0.59 | 0.16 | BC069245 | stromal cell protein |
| C20orf31 | 0.10 | 0.59 | -0.44 | AK001645,BC001371 | chromosome 20 open reading frame 31 |
| Q9H486_HUMAN | -0.78 | 0.59 | -0.14 | AJ400633,AJ242549,AJ276359 | mucin 4, tracheobronchial |
| - | -0.45 | 0.59 | -0.41 | CR745100 | similar to RIKEN cDNA 1700019P01 |
| - | 0.17 | 0.59 | -0.65 | - | - |
| TTC7A | 0.24 | 0.59 | 0.48 | AB032966 | - |
| ASB16 | 0.48 | 0.59 | -0.24 | AK054727 | ankyrin repeat and SOCS box-containing 16 |
| - | 0.08 | 0.59 | -0.63 | AK092637 | hypothetical gene supported by AK092637 |
| Q8TBX4_HUMAN | 0.36 | 0.59 | 0.05 | BC040162 | hypothetical protein MGC27085 |
| PODXL2 | 0.44 | 0.59 | 0.14 | AF219137 | podocalyxin-like 2 |
| TRPV6 | -0.14 | 0.59 | -0.77 | AY203947,AJ243501 | transient receptor potential cation channel, subfamily V, member 6 |
| - | 0.45 | 0.59 | 0.47 | - | - |
| - | -0.69 | 0.59 | 0.21 | AK125677 | hypothetical gene supported by AL512723 |
| AGTPBP1 | 0.36 | 0.59 | -0.43 | AB028958 | ATP/GTP binding protein 1 |
| SLC30A4 | 0.22 | 0.59 | 0.26 | BC026089 | solute carrier family 30 (zinc transporter), member 4 |
| - | -0.12 | 0.59 | 0.25 | AK092711 | hypothetical LOC400944 |
| - | -0.22 | 0.59 | -0.06 | AK093056 | - |
| RAB24 | 0.22 | 0.59 | 0.44 | AK021761,CR596072 | RAB24, member RAS oncogene family |
| NP_079133.3 | -0.74 | 0.59 | -0.02 | AJ314648 | hypothetical protein FLJ12735 |
| - | 0.35 | 0.59 | -0.13 | - | - |
| FUT2_HUMAN | -0.04 | 0.58 | 0.17 | BC001899 | fucosyltransferase 2 (secretor status included) |
| - | -0.24 | 0.58 | 0.18 | AK000777 | - |
| - | -0.95 | 0.58 | -0.43 | AK091858 | - |
| FCMD | -0.14 | 0.58 | -0.03 | AB008226 | Fukuyama type congenital muscular dystrophy (fukutin) |
| CLEC9_HUMAN | 0.18 | 0.58 | 0.21 | AB024718 | C-type (calcium dependent, carbohydrate-recognition domain) lectin, superfamily member 9 |
| APCDD1 | 0.39 | 0.58 | -0.34 | - | adenomatosis polyposis coli down-regulated 1 |
| - | 0.29 | 0.58 | 0.22 | AK092112 | - |
| - | -0.14 | 0.58 | -2.48 | AK027134 | - |
| - | 0.38 | 0.58 | -0.63 | AK054562 | - |
| C15orf20 | 0.25 | 0.58 | -0.08 | - | chromosome 15 open reading frame 20 |
| XP_372921.1 | 0.07 | 0.58 | -0.68 | - | similar to UPF0315 protein (AD-001) (HSPC152/HSPC170) |
| Q8IYJ1_HUMAN | 0.40 | 0.58 | -0.34 | BC035735 | copine family member |
| - | -0.12 | 0.58 | 0.02 | BC056606,BC047594,BC068610 | similar to Williams Beuren syndrome chromosome region 19 |
| HTR1D | 0.00 | 0.58 | 0.45 | BC007720 | 5-hydroxytryptamine (serotonin) receptor 1D |
| - | 0.19 | 0.58 | 0.35 | - | - |
| KIF13A | 0.28 | 0.58 | 0.49 | AY014403 | kinesin family member 13A |
| TRHDE_HUMAN | -0.14 | 0.58 | -0.02 | AF126372 | thyrotropin-releasing hormone degrading ectoenzyme |
| MYH7B | -0.48 | 0.58 | 0.33 | AB040945 | myosin, heavy polypeptide 7B, cardiac muscle, beta |
| KCNE1L | 0.00 | 0.58 | 0.21 | BC035330 | KCNE1-like |
| ATP4B | -0.10 | 0.58 | -0.65 | BC029059 | ATPase, H+/K+ exchanging, beta polypeptide |
| FBXW2 | 0.23 | 0.58 | -0.12 | - | F-box and WD-40 domain protein 2 |
| - | 0.10 | 0.58 | -0.31 | AK057593 | - |
| - | 0.13 | 0.58 | -0.31 | AK092751 | - |
| EGFL4 | -0.24 | 0.58 | 0.21 | AB011541,AY280362 | EGF-like-domain, multiple 4 |
| H2AFJ | -0.29 | 0.58 | 0.08 | AL133626 | H2A histone family, member J |
| ACTC_HUMAN | 0.13 | 0.58 | 0.36 | BC009978 | actin, alpha, cardiac muscle |
| - | 0.02 | 0.58 | -0.68 | - | - |
| F2RL3 | 0.38 | 0.58 | 0.21 | AF055917 | coagulation factor II (thrombin) receptor-like 3 |
| Q96AV1_HUMAN | 0.03 | 0.58 | -0.23 | BC068516 | similar to hypothetical protein FLJ20581 |
| - | -1.17 | 0.58 | 0.30 | - | similar to hypothetical protein FLJ21934 |
| C10orf42 | 0.10 | 0.58 | 0.20 | BC010682 | chromosome 10 open reading frame 42 |
| CDH3 | 0.21 | 0.58 | 0.14 | X63629,BC041846 | cadherin 3, type 1, P-cadherin (placental) |
| Q9BXV0_HUMAN | 0.40 | 0.58 | -0.12 | AF280797 | - |
| Q8N1K9_HUMAN | 0.00 | 0.58 | -0.58 | - | - |
| FLRT2 | -0.09 | 0.58 | -0.07 | AK056649 | fibronectin leucine rich transmembrane protein 2 |
| LCP1 | 0.24 | 0.58 | 0.13 | BC010271 | lymphocyte cytosolic protein 1 (L-plastin) |
| MEF2C | 0.41 | 0.58 | -0.37 | L08895,AL833268 | MADS box transcription enhancer factor 2, polypeptide C (myocyte enhancer factor 2C) |
| CENTD3 | 0.50 | 0.57 | -0.21 | AJ310567,AK001579 | centaurin, delta 3 |
| HRASLS | -0.31 | 0.57 | 0.10 | - | HRAS-like suppressor |
| NCKIPSD | 0.32 | 0.57 | 0.28 | AF178432 | NCK interacting protein with SH3 domain |
| - | 0.38 | 0.57 | -0.05 | AK024478 | - |
| NP_077284.1 | 0.39 | 0.57 | 0.35 | AK096551 | hypothetical protein MGC4172 |
| TNFSF7 | -0.10 | 0.57 | 0.39 | L08096,BG421005 | tumor necrosis factor (ligand) superfamily, member 7 |
| - | -0.04 | 0.57 | -0.02 | - | - |
| - | 0.36 | 0.57 | -0.10 | - | coagulation factor XIII, A1 polypeptide |
| Q7Z758_HUMAN | 0.43 | 0.57 | 0.45 | - | - |
| - | 0.42 | 0.57 | -0.15 | - | - |
| - | 0.33 | 0.57 | 0.11 | - | similar to Cadherin-related tumor suppressor precursor (Fat protein) |
| Q8IW66_HUMAN | -0.02 | 0.57 | -0.05 | - | - |
| - | -0.09 | 0.57 | 0.23 | BQ188221 | hypothetical LOC388917 |
| - | -0.20 | 0.57 | 0.01 | AK090690 | - |
| ATP2B4 | 0.23 | 0.57 | -0.10 | M83363 | ATPase, Ca++ transporting, plasma membrane 4 |
| CGNL1 | 0.44 | 0.57 | 0.36 | AY274808,AL110171 | cingulin-like 1 |
| NP_006451.1 | 0.50 | 0.57 | 0.45 | AB021179 | HMBA-inducible |
| RSAFD1 | -0.07 | 0.57 | 0.26 | BC068520,AK001762 | radical S-adenosyl methionine and flavodoxin domains 1 |
| Q96H65_HUMAN | -0.14 | 0.57 | 0.36 | - | hypothetical protein FLJ22301 |
| ENTPD6 | 0.19 | 0.57 | 0.08 | - | ectonucleoside triphosphate diphosphohydrolase 6 (putative function) |
| NP_775899.1 | 0.31 | 0.57 | -0.57 | AK097776 | hypothetical protein FLJ40457 |
| SLC13A4 | -0.14 | 0.57 | -0.06 | BC030689 | solute carrier family 13 (sodium/sulfate symporters), member 4 |
| NP_004245.2 | 0.12 | 0.57 | -0.09 | AK123243 | solute carrier family 22 (organic anion transporter), member 8 |
| - | -1.61 | 0.57 | 0.24 | - | - |
| - | -0.24 | 0.57 | -0.22 | - | - |
| TCBA1 | 0.12 | 0.57 | -0.31 | AK055326,BC035062 | T-cell lymphoma breakpoint associated target 1 |
| - | -0.16 | 0.57 | -0.99 | AK094166 | - |
| - | -0.67 | 0.57 | 0.04 | AK093662 | - |
| - | 0.13 | 0.57 | -1.33 | AK093215 | hypothetical protein LOC283332 |
| BUCS1 | 0.27 | 0.56 | 0.30 | - | butyryl Coenzyme A synthetase 1 |
| C6orf190 | 0.05 | 0.56 | 0.36 | AK128377 | chromosome 6 open reading frame 190 |
| KIT | 0.03 | 0.56 | -0.03 | X06182,BC071593 | v-kit Hardy-Zuckerman 4 feline sarcoma viral oncogene homolog |
| TOE1 | 0.09 | 0.56 | -0.35 | AK093320 | target of EGR1, member 1 (nuclear) |
| Q96DH5_HUMAN | 0.35 | 0.56 | 0.42 | BC063426,AL110227 | - |
| MAP2K5 | 0.37 | 0.56 | 0.01 | - | mitogen-activated protein kinase kinase 5 |
| - | 0.41 | 0.56 | 0.30 | - | similar to beta-tubulin 4Q |
| FAM11A | -0.10 | 0.56 | 0.32 | AF353675 | family with sequence similarity 11, member A |
| COL4A5 | 0.19 | 0.56 | 0.49 | M58526 | collagen, type IV, alpha 5 (Alport syndrome) |
| - | 0.40 | 0.56 | 0.23 | - | hypothetical LOC389172 |
| - | -0.59 | 0.56 | -0.16 | AK023675 | - |
| ZNF524 | 0.06 | 0.56 | 0.09 | BG116646,BC014666 | zinc finger protein 524 |
| PADI2 | -0.46 | 0.56 | 0.41 | AB023211 | - |
| Q6ZVD3_HUMAN | 0.27 | 0.56 | -1.51 | AK124700 | - |
| - | 0.40 | 0.56 | 0.33 | BM931866 | similar to RIKEN cDNA 6030419C18 gene |
| YES1 | -0.58 | 0.56 | 0.11 | M15990,BC048960 | v-yes-1 Yamaguchi sarcoma viral oncogene homolog 1 |
| Q7Z6I4_HUMAN | 0.40 | 0.56 | 0.12 | AK024444 | serine arginine-rich pre-mRNA splicing factor SR-A1 |
| BCAS1 | 0.27 | 0.56 | -0.93 | AF041260 | breast carcinoma amplified sequence 1 |
| Q6UXZ8_HUMAN | 0.30 | 0.56 | 0.25 | AK000582 | hypothetical protein FLJ13710 |
| HSD3B1 | -0.35 | 0.56 | 0.40 | CD014103 | hydroxy-delta-5-steroid dehydrogenase, 3 beta- and steroid delta-isomerase 1 |
| CALB2 | 0.27 | 0.56 | -0.04 | BC015484 | calbindin 2, 29kDa (calretinin) |
| DNAH12 | -0.06 | 0.56 | -0.50 | - | - |
| TEX14 | -0.14 | 0.56 | 0.11 | AL834143 | testis expressed sequence 14 |
| GPR58_HUMAN | -0.10 | 0.56 | -0.04 | AY703480 | G protein-coupled receptor 58 |
| EN1 | 0.20 | 0.56 | -0.16 | - | engrailed homolog 1 |
| - | -0.07 | 0.56 | 0.45 | BC027852 | ribosomal protein L13A-like |
| XP_370692.2 | -0.17 | 0.56 | 0.46 | - | hypothetical protein LOC121006 |
| SIA7C_HUMAN | 0.50 | 0.56 | -0.06 | BX648274 | - |
| OAS2 | -0.66 | 0.56 | -0.15 | M87284 | 2'-5'-oligoadenylate synthetase 2, 69/71kDa |
| - | -0.03 | 0.56 | -0.44 | CD697081 | LOC442143 |
| - | -1.06 | 0.56 | 0.17 | AK091662 | - |
| MYH14 | 0.49 | 0.56 | -0.02 | AY165122 | myosin, heavy polypeptide 14 |
| - | 0.37 | 0.56 | -0.89 | BQ420400 | hypothetical LOC392670 |
| - | -0.01 | 0.56 | -0.98 | BX648423 | hypothetical gene supported by AL713721 |
| - | -0.03 | 0.56 | -0.74 | AK027541 | - |
| IL12RB2 | -0.67 | 0.56 | 0.38 | U64198 | interleukin 12 receptor, beta 2 |
| CDV1 | 0.24 | 0.56 | -0.04 | - | carnitine deficiency-associated, expressed in ventricle 1 |
| GLP1R | 0.36 | 0.55 | 0.21 | U01157 | glucagon-like peptide 1 receptor |
| XP_371286.2 | 0.34 | 0.55 | -0.77 | BC007260 | hypothetical protein MGC45731 |
| - | 0.30 | 0.55 | 0.27 | - | similar to neuralized 1 |
| - | -0.21 | 0.55 | -0.14 | - | similar to 40S ribosomal protein S10 |
| CH25H | -0.14 | 0.55 | -0.47 | AF059214,BC017843 | cholesterol 25-hydroxylase |
| LASS5 | -0.20 | 0.55 | 0.25 | BC033558,AK058033 | LAG1 longevity assurance homolog 5 (S. cerevisiae) |
| - | -0.03 | 0.55 | 0.30 | AK056431 | - |
| Q8NF38_HUMAN | -0.51 | 0.55 | -0.12 | - | - |
| - | -0.47 | 0.55 | 0.14 | AK097191 | - |
| tetA | -0.03 | 0.55 | -0.01 | AB027255 | - |
| LIMK1 | 0.28 | 0.55 | 0.12 | - | LIM domain kinase 1 |
| C10orf64 | 0.39 | 0.55 | 0.31 | - | - |
| - | -0.11 | 0.55 | -0.07 | AK094373 | - |
| ATP8B2 | 0.29 | 0.55 | 0.24 | AB032963 | ATPase, Class I, type 8B, member 2 |
| PGR | 0.29 | 0.55 | -0.39 | - | - |
| NOLA1 | -0.02 | 0.55 | 0.13 | BF241566,AJ276003 | nucleolar protein family A, member 1 (H/ACA small nucleolar RNPs) |
| HILS1_HUMAN | -0.54 | 0.55 | -0.89 | BC033456 | spermatid-specific linker histone H1-like protein |
| C2orf24 | 0.01 | 0.55 | -0.66 | BC012821 | chromosome 2 open reading frame 24 |
| C8orf5 | 0.16 | 0.55 | 0.47 | AJ305312 | - |
| EDA | 0.24 | 0.55 | 0.11 | AF040628 | ectodysplasin A |
| Q96K89_HUMAN | 0.28 | 0.55 | -0.06 | AF156100 | hemicentin |
| XP_371354.1 | -0.22 | 0.55 | -0.29 | BC035896 | hypothetical protein FLJ10157 |
| - | 0.34 | 0.55 | 0.39 | BC045579 | hypothetical protein LOC283914 |
| - | -0.59 | 0.55 | 0.06 | AK025100 | - |
| - | -0.32 | 0.55 | 0.13 | AK095439 | - |
| CAMK2A | -0.26 | 0.55 | -0.03 | BC040457,AB023185 | calcium/calmodulin-dependent protein kinase (CaM kinase) II alpha |
| DUSP2 | 0.16 | 0.55 | -0.38 | L11329 | dual specificity phosphatase 2 |
| TPD52L1 | -0.25 | 0.55 | -0.37 | - | tumor protein D52-like 1 |
| TMEM7 | 0.00 | 0.55 | 0.33 | AJ312776 | transmembrane protein 7 |
| Q8ND95_HUMAN | 0.40 | 0.55 | 0.06 | AB058736 | similar to KIAA1833 protein |
| ASB10 | -0.16 | 0.55 | -0.15 | AF417920 | ankyrin repeat and SOCS box-containing 10 |
| - | -0.13 | 0.55 | -0.51 | AK054983 | - |
| NT5C1B | -0.58 | 0.54 | 0.12 | AK097422 | 5'-nucleotidase, cytosolic IB |
| PLA2G3 | 0.00 | 0.54 | 0.13 | AF220490 | phospholipase A2, group III |
| Q8NAM0_HUMAN | 0.40 | 0.54 | 0.37 | - | - |
| FRS2 | 0.33 | 0.54 | 0.34 | - | fibroblast growth factor receptor substrate 2 |
| TACC3 | 0.31 | 0.54 | 0.23 | AJ243997 | transforming, acidic coiled-coil containing protein 3 |
| ILDR1 | -0.17 | 0.54 | -0.25 | BC044240 | immunoglobulin-like domain containing receptor 1 |
| Q9P166_HUMAN | -0.37 | 0.54 | 0.22 | - | - |
| - | -0.02 | 0.54 | -0.44 | - | hypothetical gene supported by BC040297 |
| - | -0.43 | 0.54 | 0.32 | AK001494 | - |
| - | -0.01 | 0.54 | 0.20 | - | - |
| ESPN | -0.03 | 0.54 | 0.04 | AL136880 | espin |
| Q969L1_HUMAN | -0.59 | 0.54 | -0.27 | - | Homo sapiens effector cell protease receptor 1 (EPR1) on chromosome 17 |
| C9orf115 | -0.16 | 0.54 | -0.29 | AK090922 | chromosome 9 open reading frame 115 |
| - | 0.24 | 0.54 | 0.29 | - | - |
| Q7RTZ2_HUMAN | 0.17 | 0.54 | 0.18 | AY533200,AY509884 | deubiquitinating enzyme 3 |
| AK3 | 0.25 | 0.54 | -0.17 | X60673 | adenylate kinase 3 |
| CEP2 | 0.31 | 0.54 | -0.13 | AF049105 | centrosomal protein 2 |
| OR2AJ1 | 0.08 | 0.54 | -1.25 | - | - |
| Q7Z7J6_HUMAN | -0.45 | 0.54 | 0.33 | J00068 | actin, alpha 1, skeletal muscle |
| NP_060463.1 | -0.08 | 0.54 | 0.31 | AK000956,BC068521 | hypothetical protein FLJ10094 |
| ZPBP | -0.42 | 0.54 | 0.18 | BC005223,D17570 | zona pellucida binding protein |
| OR4K1 | -0.10 | 0.54 | 0.18 | - | olfactory receptor, family 4, subfamily K, member 1 |
| - | -0.36 | 0.54 | 0.28 | - | hypothetical LOC401353 |
| BAZ2B | 0.11 | 0.54 | 0.31 | AB040909 | - |
| NP_660343.1 | 0.35 | 0.54 | -0.74 | BC014492 | LOC200420 |
| NP_862829.1 | -0.02 | 0.54 | 0.45 | BX537741 | GLI-Kruppel family member HKR2 |
| XP_166090.3 | 0.49 | 0.54 | 0.39 | - | placenta-specific 9 |
| FDX1 | -0.14 | 0.54 | -0.70 | BC010284 | ferredoxin 1 |
| FOXI1 | 0.20 | 0.54 | -0.79 | L13203 | forkhead box I1 |
| Q9P1E7_HUMAN | 0.08 | 0.54 | 0.35 | - | - |
| Q9P2C9_HUMAN | 0.12 | 0.54 | -0.09 | AB037839 | - |
| - | -0.08 | 0.54 | 0.28 | AK090782 | - |
| VCY | -0.51 | 0.54 | -0.59 | AF000979 | variable charge, Y-linked |
| NP_068352.1 | 0.17 | 0.54 | -0.11 | AK097869,AF013249 | leukocyte-associated Ig-like receptor 1 |
| NP_001005504.1 | 0.29 | 0.54 | 0.43 | - | olfactory receptor, family 4, subfamily F, member 29 |
| Q96JQ7_HUMAN | -0.14 | 0.54 | 0.14 | - | - |
| PSMF1 | 0.03 | 0.54 | 0.23 | D88378 | proteasome (prosome, macropain) inhibitor subunit 1 (PI31) |
| XP_373338.1 | 0.10 | 0.54 | 0.18 | - | similar to bA92K2.2 (similar to ubiquitin) |
| NP_775944.1 | 0.05 | 0.54 | 0.26 | AK091822 | hypothetical protein FLJ34503 |
| - | -0.75 | 0.54 | 0.01 | BC047598 | hypothetical LOC339926 |
| - | -0.07 | 0.54 | 0.11 | - | similar to microtubule-associated proteins 1A/1B light chain 3 |
| - | 0.19 | 0.54 | 0.11 | AF307451 | cat eye syndrome chromosome region, candidate 6 |
| C9orf88 | 0.02 | 0.53 | -0.14 | AL137555,AF151783 | chromosome 9 open reading frame 88 |
| PACSIN2 | -0.07 | 0.53 | 0.11 | AF128536 | protein kinase C and casein kinase substrate in neurons 2 |
| NP_056165.1 | 0.43 | 0.53 | -0.13 | D86984 | T-cell activation leucine repeat-rich protein |
| RPA2 | 0.35 | 0.53 | 0.17 | J05249 | replication protein A2, 32kDa |
| LRFN3 | 0.08 | 0.53 | -0.50 | AY358127 | leucine rich repeat and fibronectin type III domain containing 3 |
| Q8NA14_HUMAN | -0.21 | 0.53 | 0.30 | - | - |
| - | 0.39 | 0.53 | 0.46 | - | similar to RIKEN cDNA 1700113O17 |
| SLC17A2 | -0.35 | 0.53 | -0.11 | - | - |
| Q8N5X0_HUMAN | -0.05 | 0.53 | 0.12 | AK026226 | hypothetical protein FLJ22573 |
| NP_075562.2 | -0.53 | 0.53 | 0.00 | AK000909,AL713677 | hypothetical protein FLJ12644 |
| - | 0.43 | 0.53 | -1.14 | AK054903 | similar to fat3; fat3 protein |
| XP_055636.1 | 0.00 | 0.53 | -1.13 | AB067499 | KIAA1912 protein |
| - | 0.41 | 0.53 | 0.20 | - | - |
| Q9H8F7_HUMAN | -0.04 | 0.53 | 0.32 | - | - |
| C6orf65 | 0.06 | 0.53 | -0.26 | AK054724 | chromosome 6 open reading frame 65 |
| ABR | 0.43 | 0.53 | -0.43 | U01147,AK124547 | active BCR-related gene |
| NP_001007544.1 | 0.29 | 0.53 | 0.31 | AK055956 | FLJ31394 protein |
| Q96PQ5_HUMAN | 0.05 | 0.53 | -0.16 | - | - |
| - | 0.35 | 0.53 | -0.60 | - | - |
| - | 0.40 | 0.53 | -0.97 | - | hypothetical LOC148766 |
| - | 0.21 | 0.53 | -0.42 | - | hypothetical LOC387746 |
| - | -0.50 | 0.53 | 0.05 | AK021745 | - |
| ACVR2 | -0.20 | 0.53 | -0.16 | M93415 | activin A receptor, type II |
| - | -0.05 | 0.53 | -0.05 | - | - |
| C10orf110 | 0.19 | 0.53 | 0.38 | - | chromosome 10 open reading frame 110 |
| IFNA8 | 0.15 | 0.53 | 0.47 | K01900 | interferon, alpha 8 |
| - | -0.22 | 0.53 | -0.12 | - | hypothetical LOC389390 |
| - | -0.54 | 0.53 | 0.02 | - | similar to hypothetical protein |
| O15420_HUMAN | -0.44 | 0.53 | 0.14 | - | - |
| - | 0.00 | 0.53 | 0.23 | AF339796 | heparan sulfate 6-O-sulfotransferase 3 |
| RAB27B | -0.17 | 0.53 | 0.44 | - | RAB27B, member RAS oncogene family |
| - | 0.26 | 0.53 | 0.12 | AK021976 | - |
| HNRPL | 0.29 | 0.52 | 0.23 | AB044547,X16135 | heterogeneous nuclear ribonucleoprotein L |
| USE1_HUMAN | 0.20 | 0.52 | 0.35 | AK074683 | uncharacterized hematopoietic stem/progenitor cells protein MDS032 |
| TENS1 | 0.26 | 0.52 | -0.01 | AK001539,CR749644 | tensin-like SH2 domain containing 1 |
| NP_689612.1 | 0.27 | 0.52 | -0.19 | AK055396,BC059365 | hypothetical protein FLJ30834 |
| Q8NBL2_HUMAN | 0.25 | 0.52 | -0.10 | AK075442 | - |
| Q8NH82_HUMAN | 0.01 | 0.52 | -0.24 | - | - |
| UBE2J2 | 0.11 | 0.52 | 0.23 | - | ubiquitin-conjugating enzyme E2, J2 (UBC6 homolog, yeast) |
| NP_001008238.1 | -0.35 | 0.52 | -0.39 | - | similar to CG14894-PA |
| - | 0.06 | 0.52 | -0.03 | - | similar to FKSG30 |
| NP_057728.1 | 0.40 | 0.52 | 0.32 | AF242769 | mesenchymal stem cell protein DSC54 |
| SEPT11 | 0.28 | 0.52 | 0.09 | AK001711,CR627457 | septin 11 |
| Q99777_HUMAN | -0.47 | 0.52 | 0.42 | U79275 | - |
| - | 0.14 | 0.52 | 0.42 | - | hypothetical LOC388117 |
| NP_001005285.1 | -0.43 | 0.52 | -0.51 | - | olfactory receptor, family 2, subfamily AT, member 4 |
| - | 0.38 | 0.52 | 0.35 | - | - |
| - | 0.39 | 0.52 | 0.47 | - | similar to RIKEN cDNA E330026B02 |
| RPGRIP1 | 0.26 | 0.52 | -1.01 | AJ417067 | retinitis pigmentosa GTPase regulator interacting protein 1 |
| TRIM6 | 0.21 | 0.52 | -0.03 | BC047564,AF220030 | tripartite motif-containing 6 |
| SESN3 | 0.49 | 0.52 | 0.29 | BC017296 | sestrin 3 |
| GZMH | -0.21 | 0.52 | -0.16 | - | granzyme H (cathepsin G-like 2, protein h-CCPX) |
| R3R2_HUMAN | -0.16 | 0.52 | 0.44 | AY288415 | relaxin 3 receptor 2 |
| NP_996801.1 | 0.08 | 0.52 | 0.05 | BC063598 | degenerative spermatocyte homolog 2, lipid desaturase (Drosophila) |
| - | 0.49 | 0.52 | 0.22 | AF117946 | Rap guanine nucleotide exchange factor (GEF)-like 1 |
| DUSP15 | 0.46 | 0.52 | 0.14 | AK000652 | - |
| - | 0.31 | 0.52 | 0.16 | - | - |
| - | 0.17 | 0.52 | -0.96 | - | - |
| - | 0.16 | 0.52 | 0.18 | AK097441 | - |
| Q8TC74_HUMAN | 0.47 | 0.52 | -0.46 | AK000456 | hypothetical protein FLJ20449 |
| NEK6 | -0.01 | 0.52 | 0.45 | - | - |
| DSPP | -0.17 | 0.52 | -0.31 | - | dentin sialophosphoprotein |
| - | -0.26 | 0.52 | -0.09 | AL049311 | - |
| CA11 | 0.24 | 0.52 | 0.28 | AF067662,AY358967 | carbonic anhydrase XI |
| TAR04_HUMAN | -0.14 | 0.52 | -0.02 | AY183470 | trace amine receptor 4 |
| CLIC6 | -0.05 | 0.52 | -0.48 | AF448439 | chloride intracellular channel 6 |
| - | -0.94 | 0.52 | 0.16 | BC026095 | - |
| VAMP4 | 0.49 | 0.52 | -0.17 | AF052100,AK056124 | vesicle-associated membrane protein 4 |
| C10orf76 | 0.13 | 0.52 | 0.35 | AK023176 | chromosome 10 open reading frame 76 |
| ITCH_HUMAN | 0.27 | 0.52 | -0.46 | AB056663,BC011571 | itchy homolog E3 ubiquitin protein ligase (mouse) |
| NP_009120.1 | 0.30 | 0.52 | -0.17 | AF261091 | ATP-binding cassette, sub-family F (GCN20), member 2 |
| XP_053966.3 | 0.32 | 0.52 | -0.34 | - | hypothetical protein LOC113230 |
| NALP10 | -0.01 | 0.52 | 0.17 | CR622836 | NACHT, leucine rich repeat and PYD containing 10 |
| - | -0.03 | 0.52 | -0.19 | - | - |
| RALGPS1 | 0.18 | 0.52 | 0.23 | AB002349,BX648170 | Ral GEF with PH domain and SH3 binding motif 1 |
| ZNF408 | 0.48 | 0.52 | -0.31 | AF346626 | zinc finger protein 408 |
| - | 0.08 | 0.52 | 0.45 | - | similar to nucleolus-cytoplasm shuttle phosphoprotein - rat |
| - | 0.48 | 0.52 | -0.66 | AK025576 | - |
| Q8TBY7_HUMAN | 0.14 | 0.51 | 0.06 | BC028424 | - |
| WDR33 | 0.30 | 0.51 | 0.01 | AB044749 | WD repeat domain 33 |
| AKAP1 | 0.30 | 0.51 | 0.30 | - | A kinase (PRKA) anchor protein 1 |
| Q8WYU1_HUMAN | -0.28 | 0.51 | -0.23 | - | - |
| - | 0.39 | 0.51 | 0.43 | - | olfactory receptor, family 10, subfamily G, member 4 |
| NP_976224.2 | 0.25 | 0.51 | -0.24 | - | rai-like protein |
| - | -0.13 | 0.51 | -0.05 | - | similar to ribosomal protein S23 |
| PGF | -0.03 | 0.51 | 0.42 | - | - |
| ALAS1 | 0.45 | 0.51 | -0.37 | AY260745 | aminolevulinate, delta-, synthase 1 |
| AMELY | -0.21 | 0.51 | -0.42 | M86933 | amelogenin, Y-linked |
| ECE1 | -0.06 | 0.51 | -0.07 | Z35307,BX648351 | endothelin converting enzyme 1 |
| TUBAL3 | -0.17 | 0.51 | 0.20 | AK025318 | tubulin, alpha-like 3 |
| MOBKL2C | 0.16 | 0.51 | 0.06 | BC026078 | MOB1, Mps One Binder kinase activator-like 2C (yeast) |
| - | 0.43 | 0.51 | 0.14 | AK096319 | - |
| PCSK1 | -0.18 | 0.51 | -0.05 | X64810 | proprotein convertase subtilisin/kexin type 1 |
| - | 0.12 | 0.51 | 0.27 | - | similar to 60S ribosomal protein L32 |
| - | 0.41 | 0.51 | -1.23 | AK093454 | - |
| PRKCSH | 0.08 | 0.51 | -0.06 | J03075,AK130663 | protein kinase C substrate 80K-H |
| Q5T1G5_HUMAN | 0.28 | 0.51 | -0.42 | - | - |
| ARSA | -0.06 | 0.51 | 0.15 | - | arylsulfatase A |
| Q8N3H8_HUMAN | 0.38 | 0.51 | -0.05 | - | - |
| - | 0.24 | 0.51 | 0.32 | - | similar to 1-aminocyclopropane-1-carboxylate synthase |
| - | -0.18 | 0.51 | 0.45 | AK090854 | - |
| Y1223_HUMAN | 0.00 | 0.51 | -0.44 | AB033049 | KIAA1223 protein |
| KIAA0423 | 0.04 | 0.51 | -0.68 | BX648723,AB007883 | KIAA0423 |
| FXYD7 | 0.09 | 0.51 | -0.04 | - | FXYD domain containing ion transport regulator 7 |
| - | -0.35 | 0.51 | -0.34 | BC036413 | hypothetical protein LOC283400 |
| RARRES3 | 0.02 | 0.51 | -0.17 | AF092922 | retinoic acid receptor responder (tazarotene induced) 3 |
| ARG1 | 0.28 | 0.51 | -0.08 | AK128314 | arginase, liver |
| NP_996669.1 | 0.45 | 0.51 | -0.38 | - | similar to Hypothetical protein CBG21647 |
| OR2L13 | 0.21 | 0.51 | 0.22 | BC028158 | olfactory receptor, family 2, subfamily L, member 13 |
| - | 0.15 | 0.51 | 0.13 | - | B-cell receptor-associated protein 29 |
| - | 0.13 | 0.51 | 0.39 | AK126861 | TGFB1-induced anti-apoptotic factor 1 |
| PCTK1 | 0.33 | 0.51 | 0.20 | BC015607 | PCTAIRE protein kinase 1 |
| CD3D | -0.04 | 0.51 | -0.48 | - | CD3D antigen, delta polypeptide (TiT3 complex) |
| - | 0.18 | 0.51 | -0.44 | AL133573 | - |
| ATF7 | -0.07 | 0.51 | -0.04 | X52943 | activating transcription factor 7 |
| THAP8 | -0.02 | 0.51 | -0.02 | AK057453,AK093048 | THAP domain containing 8 |
| TCOF1 | 0.25 | 0.51 | 0.03 | U76366 | Treacher Collins-Franceschetti syndrome 1 |
| POU3F1 | -0.28 | 0.51 | 0.16 | L26494 | POU domain, class 3, transcription factor 1 |
| - | 0.38 | 0.51 | 0.01 | - | LOC440869 |
| - | 0.00 | 0.51 | -0.61 | AK094046 | - |
| ARHGEF10 | 0.00 | 0.50 | -0.16 | AB002292,CR749570 | Rho guanine nucleotide exchange factor (GEF) 10 |
| MICA2_HUMAN | 0.21 | 0.50 | -0.85 | BX538021 | - |
| HIPK3 | -0.07 | 0.50 | -0.09 | AF004849,AF305239 | homeodomain interacting protein kinase 3 |
| C20orf79 | 0.19 | 0.50 | 0.29 | - | chromosome 20 open reading frame 79 |
| POLQ | 0.00 | 0.50 | -1.49 | AY032677 | polymerase (DNA directed), theta |
| NP_060673.1 | 0.05 | 0.50 | -0.48 | AK096590,AK001610 | hypothetical protein FLJ10748 |
| ATXN7L2 | 0.34 | 0.50 | 0.14 | AK090460 | ataxin 7-like 1 |
| F13A1 | 0.30 | 0.50 | 0.22 | - | - |
| - | -0.09 | 0.50 | 0.47 | AK128188 | hypothetical gene supported by AK128188 |
| MLANA | -0.24 | 0.50 | -0.40 | U06452 | melan-A |
| NP_073590.1 | 0.44 | 0.50 | 0.29 | BX647916 | - |
| Q6ZW15_HUMAN | -0.15 | 0.50 | 0.11 | - | - |
| NP_919270.1 | 0.11 | 0.50 | -0.51 | AK128691 | hypothetical protein LOC169355 |
| - | -0.05 | 0.50 | -0.21 | BX100818 | hypothetical LOC388348 |
| Q9HAB0_HUMAN | 0.17 | 0.50 | 0.35 | AF004828,BC036513 | rab3 GTPase-activating protein, non-catalytic subunit (150kD) |
| PRDM1 | 0.17 | 0.50 | 0.04 | AY198414,AF084199 | PR domain containing 1, with ZNF domain |
| KAZALD1 | 0.31 | 0.50 | -0.39 | BC053611,AY014271,AK172864 | hypothetical gene supported by BC012394; BC053611 |
| Q8N4A7_HUMAN | -0.04 | 0.50 | -0.15 | BC041021,BC001607 | - |
| - | -2.07 | 0.50 | -0.36 | AK097032 | hypothetical protein MGC17403 |
| - | 0.18 | 0.50 | -0.01 | AK094659 | - |
