# Supplementary material for: Microarray Analysis on Human Neuroblastoma Cells Exposed to Aluminum, β1–42-Amyloid or the β1–42-Amyloid Aluminum Complex
Source: PLoS One. 2011 Jan 27;6(1):e15965. doi: 10.1371/journal.pone.0015965 (PMC3029275; doi:10.1371/journal.pone.0015965)
Supplement: Table S2 — List of genes (1815) selectively downexpressed upon exposure to Aβ-Al compared to exposures to Aβ or Al alone. (DOC) [file pone.0015965.s004.doc]

| gene_symbol | Aβ  (Log2 Ratio) | AβAL  (Log2 Ratio) | AL  (Log2 Ratio) | RefSeq | description |
| --- | --- | --- | --- | --- | --- |
| - | 0.24 | -3.87 | 0.58 | - | 25 kDa protein [Source:IPI;Acc:IPI00333523] |
| CST9L | 1.00 | -3.81 | 0.47 | NM_080610 | Cystatin 9-like precursor (UNQ1835/PRO3543). [Source:Uniprot/SWISSPROT;Acc:Q9H4G1] |
| - | -0.08 | -3.65 | -0.32 | NM_139173 | CG10806-like (LOC150159), mRNA [Source:RefSeq_dna;Acc:NM_139173] |
| SCML2 | 0.35 | -3.18 | 0.84 | NM_006089 | Sex comb on midleg-like protein 2. [Source:Uniprot/SWISSPROT;Acc:Q9UQR0] |
| - | 0.01 | -2.81 | -0.23 | - | Hypothetical protein |
| - | 0.60 | -2.78 | 0.39 | - | Hypothetical protein |
| B3GNTL1 | -0.16 | -2.75 | 1.05 | NM_001009905,NM_194288 | UDP-GlcNAc:betaGal beta-1,3-N-acetylglucosaminyltransferase-like 1 [Source:RefSeq_peptide;Acc:NP_001009905] |
| - | 0.77 | -2.68 | 0.46 | - | Non-protein coding transcript |
| - | -0.31 | -2.68 | 1.25 | - | Hypothetical protein |
| - | 0.54 | -2.65 | 2.20 | - | Hypothetical protein |
| - | -0.41 | -2.62 | -0.40 | XM_498603 | - |
| - | -0.06 | -2.44 | 0.30 | NM_144725 | - |
| NP_775889.1 | -0.50 | -2.40 | -0.22 | NM_173618 | - |
| OR51E1 | 0.06 | -2.28 | 0.33 | - | Olfactory receptor 51E1. [Source:Uniprot/SWISSPROT;Acc:Q8TCB6] |
| Q8NBV6_HUMAN | 0.41 | -2.25 | 0.47 | - | - |
| MAS1 | 0.12 | -2.21 | 0.33 | NM_002377 | MAS proto-oncogene. [Source:Uniprot/SWISSPROT;Acc:P04201] |
| Q96CB2_HUMAN | 0.02 | -2.16 | -0.41 | - | - |
| OR2B3 | 0.00 | -2.16 | -0.08 | NM_001005226 | Olfactory receptor 2B3 (Olfactory receptor 6-4) (OR6-4) (Hs6M1-1). [Source:Uniprot/SWISSPROT;Acc:O76000] |
| - | 0.47 | -2.12 | -0.41 | - | Hypothetical protein |
| - | -0.25 | -2.09 | -0.45 | - | Hypothetical protein |
| C9orf71 | 0.27 | -2.08 | -0.18 | XM_376874 | PREDICTED: chromosome 9 open reading frame 71 [Source:RefSeq_peptide;Acc:XP_376874] |
| NP_001009913.1 | 0.64 | -2.08 | 0.69 | NM_001009913 | - |
| - | -0.30 | -2.07 | 0.90 | XM_495890 | - |
| OR4K15 | 0.72 | -2.03 | 0.10 | NM_001005486 | Olfactory receptor 4K15. [Source:Uniprot/SWISSPROT;Acc:Q8NH41] |
| SHPRH | 0.38 | -2.02 | -0.30 | NM_173082 | SNF2 histone linker PHD RING helicase [Source:RefSeq_peptide;Acc:NP_775105] |
| - | 0.52 | -2.01 | -0.35 | - | Non-protein coding transcript |
| ALOX15 | 0.00 | -2.00 | 0.23 | NM_001140 | Arachidonate 15-lipoxygenase (EC 1.13.11.33) (Arachidonate omega-6 lipoxygenase) (15-LOX). [Source:Uniprot/SWISSPROT;Acc:P16050] |
| TGFBR3 | -0.15 | -1.99 | 0.54 | NM_003243 | TGF-beta receptor type III precursor (TGFR-3) (Transforming growth factor beta receptor III) (Betaglycan). [Source:Uniprot/SWISSPROT;Acc:Q03167] |
| - | 0.17 | -1.98 | 4.08 | - | 4 kDa protein [Source:IPI;Acc:IPI00411872] |
| TM4SF8 | -0.25 | -1.96 | 0.11 | NM_005724,NM_198902 | Transmembrane 4 superfamily member 8 (Tetraspanin 3) (Tspan-3) (Tetraspanin TM4-A). [Source:Uniprot/SWISSPROT;Acc:O60637] |
| RDH10 | -0.36 | -1.95 | 0.30 | NM_172037 | retinol dehydrogenase 10 [Source:RefSeq_peptide;Acc:NP_742034] |
| Q66ZJ5_HUMAN | -0.05 | -1.95 | -0.04 | NM_178134,XR_000161 | Cytochrome P450. [Source:Uniprot/SPTREMBL;Acc:Q66ZJ5] |
| - | 0.65 | -1.93 | -0.25 | XM_497872 | - |
| OLIG2 | -0.09 | -1.93 | 0.68 | NM_005806 | Oligodendrocyte transcription factor 2 (Oligo2) (Basic helix-loop- helix protein class B 1) (Protein kinase C-binding protein RACK17) (Protein kinase C binding protein 2). [Source:Uniprot/SWISSPROT;Acc:Q13516] |
| Q9P1G9_HUMAN | -0.19 | -1.92 | 0.09 | - | - |
| - | -0.09 | -1.90 | -0.01 | XM_498999 | - |
| - | -0.16 | -1.90 | 0.33 | XM_374086 | - |
| XP_497740.1 | -0.31 | -1.89 | -0.45 | XM_497740 | PREDICTED: similar to Bcl-2-interacting protein beclin [Source:RefSeq_peptide;Acc:XP_497740] |
| CA2 | 1.51 | -1.87 | 0.46 | NM_000067 | Carbonic anhydrase II (EC 4.2.1.1) (Carbonate dehydratase II) (CA-II) (Carbonic anhydrase C). [Source:Uniprot/SWISSPROT;Acc:P00918] |
| - | 1.07 | -1.87 | -0.08 | - | 51 kDa protein [Source:IPI;Acc:IPI00335409] |
| NP_689974.1 | 0.34 | -1.86 | -0.26 | NM_152761 | - |
| XP_371200.1 | 0.26 | -1.85 | 0.88 | XM_371200 | PREDICTED: similar to R30217_1 [Source:RefSeq_peptide;Acc:XP_371200] |
| NP_060812.2 | -0.18 | -1.83 | 0.13 | NM_018342 | - |
| OR52E6 | -0.25 | -1.83 | 0.22 | NM_001005168 | Olfactory receptor 52E6. [Source:Uniprot/SWISSPROT;Acc:Q96RD3] |
| Q96MG3_HUMAN | -0.01 | -1.83 | 1.09 | - | - |
| - | -0.32 | -1.82 | 0.35 | - | Hypothetical protein |
| NP_004378.1 | 0.38 | -1.82 | 0.10 | NM_004387 | NK2 transcription factor related, locus 5 [Source:RefSeq_peptide;Acc:NP_004378] |
| NP_694949.1 | -0.37 | -1.82 | -0.22 | NM_153217 | - |
| - | 0.50 | -1.82 | 1.28 | - | Hypothetical protein |
| - | -0.18 | -1.81 | 0.08 | XM_378828,XM_498732 | - |
| MRGX2_HUMAN | -0.48 | -1.79 | -0.41 | NM_054030 | Mas-related G-protein coupled receptor member X2. [Source:Uniprot/SWISSPROT;Acc:Q96LB1] |
| SERPINA9 | -0.20 | -1.76 | -0.23 | NM_175739 | serine (or cysteine) proteinase inhibitor, clade A (alpha-1 antiproteinase, antitrypsin), member 9 [Source:RefSeq_peptide;Acc:NP_783866] |
| - | 1.84 | -1.75 | 1.59 | - | Hypothetical protein |
| OR2F1 | 1.79 | -1.74 | -0.17 | NM_012369 | Olfactory receptor 2F1 (Olfactory receptor-like protein OLF3). [Source:Uniprot/SWISSPROT;Acc:Q13607] |
| - | -0.24 | -1.74 | 0.75 | XM_378340 | - |
| DUS21_HUMAN | 0.59 | -1.73 | 0.43 | NM_022076 | Dual specificity protein phosphatase 21 (EC 3.1.3.48) (EC 3.1.3.16) (Low molecular weight dual specificity phosphatase 21). [Source:Uniprot/SWISSPROT;Acc:Q9H596] |
| - | -0.12 | -1.73 | -0.30 | - | Hypothetical protein |
| - | -0.21 | -1.72 | -0.38 | XM_497945 | - |
| KIAA1914 | 0.43 | -1.72 | 0.12 | NM_001001936,NM_032550 | - |
| Q8N7F5_HUMAN | -0.08 | -1.71 | -0.25 | NM_173518 | - |
| - | 0.31 | -1.71 | 0.48 | - | Hypothetical protein |
| - | 0.09 | -1.71 | 0.67 | - | Non-protein coding transcript |
| INSL6 | 0.36 | -1.70 | 0.52 | NM_007179 | Insulin-like peptide INSL6 precursor (Insulin-like peptide 6) (Relaxin/insulin-like factor 1). [Source:Uniprot/SWISSPROT;Acc:Q9Y581] |
| XP_370648.2 | -0.08 | -1.69 | 1.42 | XM_370648 | PREDICTED: hypothetical protein XP_370648 [Source:RefSeq_peptide;Acc:XP_370648] |
| - | 0.51 | -1.69 | 0.93 | - | - |
| NAT1 | -0.39 | -1.68 | -0.24 | NM_000662 | Arylamine N-acetyltransferase 1 (EC 2.3.1.5) (Arylamide acetylase 1) (Arylamine N-acetyltransferase, monomorphic) (MNAT) (N- acetyltransferase type 1) (NAT-1). [Source:Uniprot/SWISSPROT;Acc:P18440] |
| - | 0.44 | -1.68 | -0.36 | XM_496740,XM_498995 | - |
| IL2_HUMAN | -0.45 | -1.67 | -0.13 | NM_000586 | Interleukin-2 precursor (IL-2) (T-cell growth factor) (TCGF) (Aldesleukin). [Source:Uniprot/SWISSPROT;Acc:P60568] |
| RIBC2 | 1.19 | -1.67 | 2.09 | NM_015653 | RIB43A domain with coiled-coils 2 [Source:RefSeq_peptide;Acc:NP_056468] |
| - | 0.04 | -1.67 | -0.47 | - | Hypothetical protein |
| ERN1 | -0.20 | -1.66 | 0.00 | NM_001433 | Serine/threonine protein kinase/endoribonuclease IRE1 precursor (Inositol-requiring 1) (hIRE1p) (IRE1a) (Ire1alpha) (Endoplasmic reticulum-to-nucleus signaling 1) [Includes: Serine/threonine protein kinase (EC 2.7.1.37); Endoribonuclease (EC 3.1.26.-)]. [ |
| - | 0.26 | -1.65 | 0.78 | - | Non-protein coding transcript |
| - | 0.31 | -1.64 | 0.12 | NM_002600 | - |
| AKAP14 | 0.00 | -1.63 | -0.20 | NM_178813,NM_001008534 | A-kinase anchoring protein 14 (Protein kinase A anchoring protein 14) (A-kinase anchoring protein 28 kDa). [Source:Uniprot/SWISSPROT;Acc:Q86UN6] |
| - | -0.27 | -1.63 | 0.07 | - | Non-protein coding transcript |
| NP_775938.1 | 0.21 | -1.61 | 0.06 | NM_173667 | - |
| - | -0.19 | -1.61 | 1.35 | XM_498772,XM_498762,XM_498781,XM_498759,XM_498765,XM_498757,XM_498766,XM_498787,XM_498773,XM_498770, | - |
| - | -0.37 | -1.61 | -0.42 | - | Hypothetical protein |
| XP_059399.2 | 0.50 | -1.60 | 0.46 | XM_059399 | PREDICTED: similar to Calcium and integrin-binding protein 1 (Calmyrin) (DNA-PKcs interacting protein) (Kinase interacting protein) (KIP) (CIB) [Source:RefSeq_peptide;Acc:XP_059399] |
| - | 0.13 | -1.60 | 0.04 | - | Hypothetical protein |
| NP_963844.1 | 0.23 | -1.59 | -0.22 | NM_201550 | leucine rich repeat containing 10 [Source:RefSeq_peptide;Acc:NP_963844] |
| - | 0.20 | -1.59 | 1.58 | - | Hypothetical protein |
| OR2T27 | 0.17 | -1.58 | 0.05 | NM_001001824 | olfactory receptor, family 2, subfamily T, member 27 [Source:RefSeq_peptide;Acc:NP_001001824] |
| - | -0.08 | -1.58 | -0.12 | - | Hypothetical protein |
| - | -0.50 | -1.58 | 1.06 | XM_498883 | - |
| GPR87_HUMAN | -0.31 | -1.58 | 0.43 | NM_023915 | Probable G-protein coupled receptor 87 (FKSG88 protein). [Source:Uniprot/SWISSPROT;Acc:Q9BY21] |
| - | 0.38 | -1.58 | 0.08 | - | 15 kDa protein [Source:IPI;Acc:IPI00412799] |
| PECAM1 | -0.29 | -1.56 | 0.00 | NM_000442 | Platelet endothelial cell adhesion molecule precursor (PECAM-1) (CD31 antigen) (EndoCAM) (GPIIA'). [Source:Uniprot/SWISSPROT;Acc:P16284] |
| PASD1 | -0.35 | -1.56 | 0.07 | NM_173493 | PAS domain containing 1 [Source:RefSeq_peptide;Acc:NP_775764] |
| - | 0.70 | -1.54 | 0.97 | XM_378272 | - |
| - | 0.08 | -1.54 | -0.14 | XM_498017 | - |
| - | -0.22 | -1.54 | 0.93 | - | Hypothetical protein |
| - | 0.05 | -1.54 | 0.02 | NM_080923 | - |
| - | 1.11 | -1.53 | 0.20 | XM_373566 | - |
| CRISP2 | 0.46 | -1.53 | -0.37 | NM_003296 | Cysteine-rich secretory protein-2 precursor (CRISP-2) (Testis-specific protein TPX-1). [Source:Uniprot/SWISSPROT;Acc:P16562] |
| MLRM_HUMAN | 0.44 | -1.53 | 1.38 | NM_006471 | Myosin regulatory light chain 2, nonsarcomeric (Myosin RLC). [Source:Uniprot/SWISSPROT;Acc:P19105] |
| LYZL6 | 0.12 | -1.53 | 0.22 | NM_020426 | lysozyme-like 6 [Source:RefSeq_peptide;Acc:NP_065159] |
| - | 0.28 | -1.52 | 1.13 | XM_378617 | - |
| - | -0.41 | -1.52 | 0.47 | - | 60 kDa protein [Source:IPI;Acc:IPI00413437] |
| - | 0.55 | -1.52 | -0.03 | XM_498267 | - |
| - | 0.35 | -1.52 | -0.29 | - | 15 kDa protein [Source:IPI;Acc:IPI00477693] |
| OR8B4 | 0.47 | -1.51 | -0.19 | NM_001005196 | Olfactory receptor 8B4. [Source:Uniprot/SWISSPROT;Acc:Q96RC9] |
| Q86XP7_HUMAN | 0.63 | -1.50 | 0.53 | - | GDDM. [Source:Uniprot/SPTREMBL;Acc:Q86XP7] |
| VPS13D | -0.18 | -1.50 | -0.07 | NM_015378,NM_018156 | vacuolar protein sorting 13D isoform 2 [Source:RefSeq_peptide;Acc:NP_060626] |
| DO | 0.25 | -1.50 | 1.06 | NM_021071 | Ecto-ADP-ribosyltransferase 4 precursor (EC 2.4.2.31) (NAD(P)(+)-- arginine ADP-ribosyltransferase 4) (Mono(ADP-ribosyl)transferase 4) (Dombrock blood group carrier molecule). [Source:Uniprot/SWISSPROT;Acc:Q93070] |
| C6orf78 | -0.36 | -1.50 | 0.12 | NM_153036 | - |
| - | 0.00 | -1.49 | -0.33 | NM_001004476 | - |
| NP_775915.1 | 0.06 | -1.49 | -0.27 | NM_173644 | - |
| - | 0.00 | -1.48 | 0.29 | NM_001008784 | - |
| OR6W1P | -0.36 | -1.47 | 2.33 | NR_002140 | Olfactory receptor 6W1 (Olfactory receptor sdolf) (Fragment). [Source:Uniprot/SWISSPROT;Acc:Q96RR8] |
| - | 0.21 | -1.47 | -0.19 | XM_378009 | - |
| GPR145 | 0.03 | -1.46 | -0.17 | NM_032503 | Melanin-concentrating hormone receptor 2 (MCH receptor 2) (MCHR-2) (MCH-R2) (MCH2R) (MCH-2R) (MCH2) (G-protein coupled receptor 145) (GPRv17). [Source:Uniprot/SWISSPROT;Acc:Q969V1] |
| PTPDC1 | -0.08 | -1.46 | 0.44 | NM_152422,NM_177995 | protein tyrosine phosphatase domain containing 1 protein isoform 1 [Source:RefSeq_peptide;Acc:NP_689635] |
| - | 1.36 | -1.46 | 0.39 | NM_014803 | - |
| - | -0.07 | -1.45 | -0.43 | XM_497879 | - |
| - | 0.07 | -1.44 | 0.27 | XM_373809 | - |
| Q16016_HUMAN | -0.06 | -1.44 | 0.04 | - | Pol protein (Fragment). [Source:Uniprot/SPTREMBL;Acc:Q16016] |
| - | 0.11 | -1.43 | 0.65 | - | 19 kDa protein [Source:IPI;Acc:IPI00478573] |
| - | 1.11 | -1.43 | -0.12 | - | 115 kDa protein [Source:IPI;Acc:IPI00479347] |
| - | 0.18 | -1.43 | 0.27 | - | Hypothetical protein |
| - | 0.55 | -1.43 | 0.62 | XM_294743 | - |
| - | 0.02 | -1.42 | 0.95 | - | 15 kDa protein [Source:IPI;Acc:IPI00156232] |
| - | -0.23 | -1.41 | -0.38 | - | Non-protein coding transcript |
| CPNE5 | 0.03 | -1.40 | -0.23 | NM_020939 | Copine V. [Source:Uniprot/SWISSPROT;Acc:Q9HCH3] |
| MYL2 | 1.16 | -1.40 | 1.58 | NM_000432 | Myosin regulatory light chain 2, ventricular/cardiac muscle isoform (MLC-2) (MLC-2v). [Source:Uniprot/SWISSPROT;Acc:P10916] |
| - | 0.25 | -1.40 | -0.32 | XM_374586,XM_294261 | 87 kDa protein [Source:IPI;Acc:IPI00414099] |
| - | -0.04 | -1.39 | 0.19 | - | 33 kDa protein [Source:IPI;Acc:IPI00396588] |
| KCNJ16 | -0.23 | -1.39 | -0.07 | NM_170741,NM_018658,NM_170742 | Inward rectifier potassium channel 16 (Potassium channel, inwardly rectifying, subfamily J, member 16) (Inward rectifier K(+) channel Kir5.1). [Source:Uniprot/SWISSPROT;Acc:Q9NPI9] |
| Q9H359_HUMAN | -0.01 | -1.39 | 0.34 | - | - |
| NP_777587.1 | -0.10 | -1.38 | -0.25 | NM_174927 | spergen-1 [Source:RefSeq_peptide;Acc:NP_777587] |
| CNGA2 | 0.07 | -1.38 | -0.34 | NM_005140 | Cyclic-nucleotide-gated olfactory channel (Cyclic-nucleotide-gated cation channel 2) (CNG channel 2) (CNG-2) (CNG2) (Fragment). [Source:Uniprot/SWISSPROT;Acc:Q16280] |
| - | 0.96 | -1.37 | 0.19 | - | 4 kDa protein [Source:IPI;Acc:IPI00479029] |
| - | -0.09 | -1.36 | 0.42 | XM_379306 | - |
| Q8TBB7_HUMAN | 1.99 | -1.36 | 1.21 | - | WDR22 protein. [Source:Uniprot/SPTREMBL;Acc:Q8TBB7] |
| OR4P4 | 0.70 | -1.35 | -0.43 | NM_001004124 | Olfactory receptor 4P4. [Source:Uniprot/SWISSPROT;Acc:Q8NGL7] |
| Q8NH47_HUMAN | -0.17 | -1.35 | 0.28 | - | Seven transmembrane helix receptor. [Source:Uniprot/SPTREMBL;Acc:Q8NH47] |
| HCG27 | 0.21 | -1.35 | 1.20 | NM_181717 | HLA complex group 27 [Source:RefSeq_peptide;Acc:NP_859068] |
| MCOLN3 | -0.09 | -1.34 | 0.57 | NM_018298 | Mucolipin-3. [Source:Uniprot/SWISSPROT;Acc:Q8TDD5] |
| DNJB7_HUMAN | 0.00 | -1.34 | 0.01 | NM_145174 | DnaJ homolog subfamily B member 7. [Source:Uniprot/SWISSPROT;Acc:Q7Z6W7] |
| - | -0.47 | -1.34 | 0.63 | XM_496892,XM_499317,XM_496905,XM_499338 | 25 kDa protein [Source:IPI;Acc:IPI00412211] |
| - | 0.64 | -1.34 | 0.11 | XM_499150 | - |
| - | 0.10 | -1.34 | -0.46 | NM_004991 | - |
| NP_997366.1 | 0.11 | -1.33 | 0.46 | NM_207483 | - |
| OR52E4 | -0.23 | -1.33 | -0.16 | NM_001005165 | Olfactory receptor 52E4. [Source:Uniprot/SWISSPROT;Acc:Q8NGH9] |
| Q9P162_HUMAN | 0.63 | -1.33 | 0.61 | - | - |
| - | -0.49 | -1.32 | -0.04 | XM_378692 | - |
| TCL1A | 0.68 | -1.32 | 1.33 | NM_021966 | T-cell leukemia/lymphoma protein 1A (P14 TCL1 protein) (TCL1 oncogene) (TCL-1 protein). [Source:Uniprot/SWISSPROT;Acc:P56279] |
| - | 0.13 | -1.32 | -0.37 | - | Non-protein coding transcript |
| - | -0.49 | -1.32 | -0.42 | NM_025168 | - |
| ZNF570 | 2.00 | -1.31 | 1.28 | NM_144694 | zinc finger protein 570 [Source:RefSeq_peptide;Acc:NP_653295] |
| - | -0.06 | -1.31 | -0.46 | - | AF232917 Human spumaretrovirus envelope protein (env) gene, partial cds; tas (orf-1) gene, complete cds; and long terminal repeat, partial sequence |
| KCNC1 | -0.11 | -1.31 | -0.01 | NM_004976 | Potassium voltage-gated channel subfamily C member 1 (Voltage-gated potassium channel subunit Kv3.1) (Kv4) (NGK2). [Source:Uniprot/SWISSPROT;Acc:P48547] |
| NP_001005210.1 | -0.35 | -1.31 | -0.30 | NM_001005210 | - |
| GIMAP8 | -0.02 | -1.31 | 0.85 | NM_175571 | GTPase, IMAP family member 8 [Source:RefSeq_peptide;Acc:NP_783161] |
| XP_495844.1 | -0.19 | -1.30 | -0.49 | XM_495844 | PREDICTED: similar to tigger transposable element derived 2 [Source:RefSeq_peptide;Acc:XP_495844] |
| Q9BVY2_HUMAN | -0.37 | -1.30 | 0.24 | - | - |
| - | 0.00 | -1.30 | -0.47 | - | Hypothetical protein |
| TMEM20 | 1.74 | -1.30 | 0.08 | NM_153226 | transmembrane protein 20 [Source:RefSeq_peptide;Acc:NP_694958] |
| - | 2.00 | -1.29 | -0.17 | NR_001458 | - |
| - | -0.47 | -1.29 | 0.26 | - | Hypothetical protein |
| - | 0.37 | -1.29 | -0.45 | - | - |
| COPE | 0.38 | -1.29 | 0.03 | NM_199442,NM_007263,NM_199444 | Coatomer epsilon subunit (Epsilon-coat protein) (Epsilon-COP). [Source:Uniprot/SWISSPROT;Acc:O14579] |
| - | 0.61 | -1.29 | -0.22 | - | Non-protein coding transcript |
| - | 0.51 | -1.28 | 1.81 | - | Hypothetical protein |
| ADAM29 | -0.36 | -1.28 | -0.49 | NM_021780,NM_021779,NM_014269 | ADAM 29 precursor (A disintegrin and metalloproteinase domain 29). [Source:Uniprot/SWISSPROT;Acc:Q9UKF5] |
| C20orf137 | 0.26 | -1.28 | 0.03 | XM_372869 | PREDICTED: similar to dJ601O1.1 (novel protein with Kunitz/Bovine pancreatic trypsin inhibitor domain) [Source:RefSeq_peptide;Acc:XP_372869] |
| SFXN5 | 0.00 | -1.28 | 0.83 | NM_144579 | Sideroflexin 5. [Source:Uniprot/SWISSPROT;Acc:Q8TD22] |
| Q8WZ02_HUMAN | -0.09 | -1.28 | -0.39 | - | - |
| - | -0.34 | -1.28 | 0.59 | NM_033496 | - |
| - | 0.92 | -1.28 | 0.01 | - | Non-protein coding transcript |
| C20orf112 | 0.03 | -1.28 | 0.19 | NM_080616 | - |
| - | -0.10 | -1.27 | -0.43 | - | 43 kDa protein [Source:IPI;Acc:IPI00478426] |
| AFM | 0.00 | -1.27 | -0.41 | NM_001133 | Afamin precursor (Alpha-albumin) (Alpha-Alb). [Source:Uniprot/SWISSPROT;Acc:P43652] |
| CASQ2 | 0.06 | -1.27 | -0.29 | NM_001232 | Calsequestrin, cardiac muscle isoform precursor (Calsequestrin 2). [Source:Uniprot/SWISSPROT;Acc:O14958] |
| Q8TBE4_HUMAN | 0.04 | -1.27 | -0.21 | - | - |
| - | 1.37 | -1.27 | -0.36 | XM_373500 | - |
| - | 0.42 | -1.27 | 0.15 | XM_498609 | - |
| NP_112600.1 | -0.12 | -1.26 | 0.18 | NM_031310 | plasmalemma vesicle associated protein [Source:RefSeq_peptide;Acc:NP_112600] |
| - | -0.25 | -1.25 | -0.45 | XM_496302 | - |
| - | 0.85 | -1.25 | 1.86 | XM_373977 | - |
| - | -0.16 | -1.25 | 0.02 | - | Hypothetical protein |
| KIAA1772 | 0.41 | -1.25 | -0.36 | NM_024935 | KIAA1772 [Source:RefSeq_peptide;Acc:NP_079211] |
| Q8TAF5_HUMAN | -0.34 | -1.25 | 1.20 | - | - |
| B3GALT3 | 0.39 | -1.25 | 1.25 | NM_033167,NM_033169,NM_033168,NM_003781 | Beta-1,3-galactosyltransferase 3 (EC 2.4.1.79) (Beta-1,3-GalTase 3) (Beta3Gal-T3) (b3Gal-T3) (Galactosylgalactosylglucosylceramide beta-D- acetyl-galactosaminyltransferase) (UDP-N- acetylgalactosamine:globotriaosylceramide beta-1,3-N- acetylgalactosaminyl |
| - | 0.19 | -1.24 | 0.14 | XM_295155 | - |
| NOX5 | -0.30 | -1.24 | -0.19 | NM_024505 | NADPH oxidase, EF hand calcium-binding domain 5 [Source:RefSeq_peptide;Acc:NP_078781] |
| - | 1.91 | -1.24 | -0.26 | XM_373559 | - |
| LGP1_HUMAN | -0.02 | -1.24 | -0.47 | NM_032484 | Protein D11Lgp1 homolog precursor. [Source:Uniprot/SWISSPROT;Acc:Q8N2G8] |
| - | -0.30 | -1.24 | 0.66 | XM_378211 | - |
| - | -0.15 | -1.24 | 0.03 | NM_024909 | - |
| ZNF90 | -0.25 | -1.24 | -0.29 | - | Zinc finger protein 90 (Zinc finger protein HTF9) (Fragment). [Source:Uniprot/SWISSPROT;Acc:Q03938] |
| - | -0.38 | -1.24 | -0.29 | XM_499237 | - |
| - | -0.24 | -1.23 | -0.04 | XM_173120 | - |
| NP_071770.1 | -0.19 | -1.23 | 0.42 | NM_022375 | oculomedin [Source:RefSeq_peptide;Acc:NP_071770] |
| - | 0.57 | -1.23 | 1.09 | XM_374694,XM_373214 | - |
| SLC25A2 | 0.13 | -1.23 | 0.27 | NM_031947 | Mitochondrial ornithine transporter 2 (Solute carrier family 25, member 2). [Source:Uniprot/SWISSPROT;Acc:Q9BXI2] |
| - | 0.42 | -1.23 | 0.90 | - | Hypothetical protein |
| TAS2R49 | -0.32 | -1.23 | 0.03 | NM_176889 | Taste receptor type 2 member 49 (T2R49) (T2R56). [Source:Uniprot/SWISSPROT;Acc:P59543] |
| - | 0.35 | -1.23 | 0.07 | - | 27 kDa protein [Source:IPI;Acc:IPI00412820] |
| TBX22 | -0.41 | -1.23 | -0.41 | NM_016954 | T-box transcription factor TBX22 (T-box protein 22). [Source:Uniprot/SWISSPROT;Acc:Q9Y458] |
| Q8N9U9_HUMAN | -0.18 | -1.22 | 0.40 | - | - |
| TIGD7 | -0.39 | -1.22 | 0.60 | NM_033208 | tigger transposable element derived 7 [Source:RefSeq_peptide;Acc:NP_149985] |
| - | 1.10 | -1.21 | 0.51 | - | Similar to Metalloprotease 1 |
| MAMDC2 | -0.26 | -1.21 | -0.48 | NM_153267 | MAM domain containing protein 2 precursor. [Source:Uniprot/SWISSPROT;Acc:Q7Z304] |
| - | -0.23 | -1.21 | -0.29 | XM_498385 | - |
| - | 0.50 | -1.21 | -0.27 | - | 79 kDa protein [Source:IPI;Acc:IPI00479482] |
| - | 0.55 | -1.21 | 0.56 | XM_498383 | - |
| - | 0.45 | -1.20 | -0.21 | XM_210515 | - |
| - | 0.38 | -1.20 | 0.69 | NM_015393 | - |
| OR10Z1 | -0.34 | -1.20 | 0.72 | NM_001004478 | Olfactory receptor 10Z1. [Source:Uniprot/SWISSPROT;Acc:Q8NGY1] |
| RNF151 | 0.58 | -1.20 | -0.17 | XM_370927 | PREDICTED: hypothetical protein LOC146310 [Source:RefSeq_peptide;Acc:XP_370927] |
| Q9BSL4_HUMAN | 0.15 | -1.20 | -0.26 | XM_498903 | MGC10955 protein. [Source:Uniprot/SPTREMBL;Acc:Q9BSL4] |
| EML2 | 1.76 | -1.20 | 0.02 | NM_012155 | Echinoderm microtubule-associated protein-like 2 (EMAP-2) (HuEMAP-2). [Source:Uniprot/SWISSPROT;Acc:O95834] |
| - | -0.37 | -1.19 | 0.25 | - | 20 kDa protein [Source:IPI;Acc:IPI00232867] |
| RPB10_HUMAN | 0.98 | -1.19 | 0.89 | NM_021128 | DNA-directed RNA polymerase II 7.6 kDa polypeptide (EC 2.7.7.6) (RPB10) (RPB7.6) (RPABC5). [Source:Uniprot/SWISSPROT;Acc:P62875] |
| CTHRC1 | -0.16 | -1.18 | -0.01 | NM_138455 | collagen triple helix repeat containing 1 [Source:RefSeq_peptide;Acc:NP_612464] |
| Q8NGQ7_HUMAN | -0.06 | -1.18 | -0.23 | - | Seven transmembrane helix receptor. [Source:Uniprot/SPTREMBL;Acc:Q8NGQ7] |
| - | 0.39 | -1.18 | -0.17 | - | 12 kDa protein [Source:IPI;Acc:IPI00479012] |
| NP_077293.1 | 0.06 | -1.18 | 0.21 | NM_024317 | leukocyte immunoglobulin-like receptor, subfamily A, member 5 [Source:RefSeq_peptide;Acc:NP_077293] |
| - | 0.50 | -1.18 | -0.27 | - | Hypothetical protein |
| - | 0.71 | -1.18 | 1.15 | - | Hypothetical protein |
| MTP | 0.06 | -1.17 | -0.33 | NM_000253 | Microsomal triglyceride transfer protein large subunit precursor. [Source:Uniprot/SWISSPROT;Acc:P55157] |
| - | -0.22 | -1.17 | 1.45 | - | 13 kDa protein [Source:IPI;Acc:IPI00478647] |
| - | -0.16 | -1.17 | -0.13 | - | Non-protein coding transcript |
| C20orf187 | 0.11 | -1.17 | -0.46 | - | OTTHUMP00000030283. [Source:Uniprot/SPTREMBL;Acc:Q9UGB4] |
| OR4L1 | 1.22 | -1.17 | 0.31 | NM_001004717 | Olfactory receptor 4L1. [Source:Uniprot/SWISSPROT;Acc:Q8NH43] |
| SPP2 | 1.42 | -1.17 | -0.45 | NM_006944 | Secreted phosphoprotein 24 precursor (Spp-24) (Secreted phosphoprotein 2). [Source:Uniprot/SWISSPROT;Acc:Q13103] |
| HIP1 | -0.21 | -1.17 | 0.11 | NM_005338 | Huntingtin interacting protein 1 (HIP-I). [Source:Uniprot/SWISSPROT;Acc:O00291] |
| - | 0.15 | -1.17 | -0.15 | XM_371778 | - |
| NP_001001683.1 | 0.43 | -1.17 | -0.47 | NM_001001683 | similar to HSPC296 [Source:RefSeq_peptide;Acc:NP_001001683] |
| PEPP1_HUMAN | -0.39 | -1.17 | 0.68 | NM_139282 | Paired-like homeobox protein PEPP-1 (Ovary-, testis- and epididymis- expressed gene protein). [Source:Uniprot/SWISSPROT;Acc:Q8NHV9] |
| - | 0.80 | -1.17 | 0.80 | XM_374066 | - |
| - | 0.16 | -1.16 | 0.11 | - | 85 kDa protein [Source:IPI;Acc:IPI00333154] |
| Q8NES6_HUMAN | 0.00 | -1.16 | 0.15 | NM_020948 | mesoderm induction early response 1 [Source:RefSeq_peptide;Acc:NP_065999] |
| NP_997222.1 | -0.15 | -1.16 | -0.17 | - | PREDICTED: hypothetical protein XP_372224 [Source:RefSeq_peptide;Acc:XP_372224] |
| - | 0.59 | -1.16 | -0.21 | - | Non-protein coding transcript |
| - | 0.14 | -1.16 | 0.73 | XM_378203 | - |
| - | -0.28 | -1.16 | -0.35 | - | AF012331 Human Endogenous Retrovirus K1,2-17 5' retroviral regulatory terminal end |
| - | 1.49 | -1.16 | 1.33 | - | Hypothetical protein |
| PPP1R3D | 0.29 | -1.16 | 1.80 | NM_006242 | Protein phosphatase 1, regulatory subunit 3D (Protein phosphatase 1, regulatory subunit 6) (Protein phosphatase 1 binding subunit R6). [Source:Uniprot/SWISSPROT;Acc:O95685] |
| - | -0.24 | -1.16 | 0.05 | - | Hypothetical protein |
| STNB_HUMAN | 0.31 | -1.15 | 0.08 | NM_033104 | Stonin 2 (Stoned B). [Source:Uniprot/SWISSPROT;Acc:Q8WXE9] |
| C5R1 | -0.13 | -1.15 | 0.29 | NM_001736 | C5a anaphylatoxin chemotactic receptor (C5a-R) (C5aR) (CD88 antigen). [Source:Uniprot/SWISSPROT;Acc:P21730] |
| - | 0.18 | -1.15 | 1.39 | XM_374173 | - |
| NP_997386.1 | 0.70 | -1.15 | 0.58 | NM_207503 | - |
| Q9BYW7_HUMAN | 0.00 | -1.15 | 0.17 | - | OTTHUMP00000029966 (Fragment). [Source:Uniprot/SPTREMBL;Acc:Q9BYW7] |
| TWSG1 | 0.24 | -1.15 | -0.44 | NM_020648 | twisted gastrulation [Source:RefSeq_peptide;Acc:NP_065699] |
| NP_001010889.1 | 0.69 | -1.15 | 1.05 | - | PREDICTED: similar to Hypothetical protein DJ845O24.2 [Source:RefSeq_peptide;Acc:XP_291638] |
| C11orf1 | 0.26 | -1.14 | 0.82 | NM_022761 | - |
| - | 0.79 | -1.14 | 1.23 | - | Hypothetical protein |
| - | -0.31 | -1.14 | 0.10 | XM_497928 | - |
| ENPP4 | 0.21 | -1.14 | -0.37 | NM_014936 | ectonucleotide pyrophosphatase/phosphodiesterase 4 (putative function) [Source:RefSeq_peptide;Acc:NP_055751] |
| ZBED2 | 0.87 | -1.14 | -0.11 | NM_024508 | Zinc finger BED domain containing protein 2. [Source:Uniprot/SWISSPROT;Acc:Q9BTP6] |
| TSKS_HUMAN | 0.29 | -1.14 | 0.88 | - | Testis-specific serine kinase substrate (Testis-specific kinase substrate) (STK22 substrate 1). [Source:Uniprot/SWISSPROT;Acc:Q9UJT2] |
| NP_963859.1 | 0.27 | -1.14 | 0.33 | NM_201565 | - |
| - | -0.01 | -1.13 | -0.33 | NM_001002915 | - |
| NP_060681.2 | 0.48 | -1.13 | -0.37 | NM_018211 | - |
| - | -0.09 | -1.13 | -0.25 | - | Hypothetical protein |
| C8A | 0.08 | -1.13 | 0.36 | NM_000562 | Complement component C8 alpha chain precursor. [Source:Uniprot/SWISSPROT;Acc:P07357] |
| - | 0.26 | -1.13 | 1.07 | - | 36 kDa protein [Source:IPI;Acc:IPI00480031] |
| - | 1.07 | -1.13 | 0.18 | - | Hypothetical protein |
| - | 0.23 | -1.13 | 0.50 | XM_495891,XM_061890,XM_210184 | 52 kDa protein [Source:IPI;Acc:IPI00413099] |
| HRH4 | 0.71 | -1.13 | 0.45 | NM_021624 | Histamine H4 receptor (HH4R) (GPRv53) (G-protein coupled receptor 105) (GPCR105) (SP9144) (AXOR35). [Source:Uniprot/SWISSPROT;Acc:Q9H3N8] |
| - | 0.33 | -1.13 | -0.45 | XM_497563 | - |
| SCN5A | 0.52 | -1.13 | 0.92 | NM_000335,NM_198056 | Sodium channel protein type V alpha subunit (Voltage-gated sodium channel alpha subunit Nav1.5) (Sodium channel protein, cardiac muscle alpha-subunit) (HH1). [Source:Uniprot/SWISSPROT;Acc:Q14524] |
| Q9H5F0_HUMAN | 0.58 | -1.12 | 0.22 | - | - |
| Q8NBH1_HUMAN | -0.42 | -1.12 | -0.19 | - | - |
| TRIM38 | -0.30 | -1.12 | -0.10 | NM_006355 | Tripartite motif protein 38 (RING finger protein 15) (Zinc finger protein RoRet). [Source:Uniprot/SWISSPROT;Acc:O00635] |
| - | 1.19 | -1.12 | 1.38 | - | 23 kDa protein [Source:IPI;Acc:IPI00402189] |
| - | -0.47 | -1.12 | 0.61 | - | Hypothetical protein |
| DEFB129 | 0.07 | -1.12 | 0.19 | NM_080831 | Beta-defensin 129 precursor (Beta-defensin 29) (DEFB-29) (UNQ5794/PRO19599). [Source:Uniprot/SWISSPROT;Acc:Q9H1M3] |
| - | 0.07 | -1.12 | -0.19 | - | Similar to NADH dehydrogenase subunit 2 |
| - | -0.36 | -1.12 | -0.35 | - | 11 kDa protein [Source:IPI;Acc:IPI00478455] |
| NP_079250.1 | -0.20 | -1.11 | 0.60 | NM_024974,XM_496191 | PREDICTED: similar to hypothetical protein FLJ11800 [Source:RefSeq_peptide;Acc:XP_496191] |
| XP_056680.5 | 0.65 | -1.11 | 0.99 | XM_056680 | PREDICTED: hypothetical protein XP_056680 [Source:RefSeq_peptide;Acc:XP_056680] |
| GJB6 | -0.31 | -1.11 | 0.37 | NM_006783 | Gap junction beta-6 protein (Connexin 30) (Cx30). [Source:Uniprot/SWISSPROT;Acc:O95452] |
| - | -0.45 | -1.11 | 0.33 | NM_030967 | - |
| - | 0.02 | -1.11 | 1.76 | XM_379164 | - |
| - | -0.01 | -1.11 | 1.40 | XM_372878 | - |
| CENTG1 | -0.43 | -1.11 | 0.15 | NM_014770 | Centaurin gamma 1. [Source:Uniprot/SWISSPROT;Acc:Q99490] |
| - | -0.40 | -1.11 | -0.32 | XM_498485 | - |
| Q5VU36_HUMAN | 0.67 | -1.10 | -0.07 | XM_372094,NM_015667 | Novel protein. [Source:Uniprot/SPTREMBL;Acc:Q5VU36] |
| - | 0.15 | -1.10 | 0.08 | - | Non-protein coding transcript |
| NP_620158.2 | 0.02 | -1.10 | 0.05 | NM_138803 | - |
| XP_496144.1 | -0.17 | -1.10 | -0.45 | XM_496144 | PREDICTED: similar to zinc finger protein 267; zinc finger (C2H2) [Source:RefSeq_peptide;Acc:XP_496144] |
| POU4F1 | 0.37 | -1.10 | 0.62 | NM_006237 | POU domain, class 4, transcription factor 1 (Brain-specific homeobox/POU domain protein 3A) (Brn-3A) (Oct-T1) (Homeobox/POU domain protein RDC-1). [Source:Uniprot/SWISSPROT;Acc:Q01851] |
| RNASE1 | 1.02 | -1.10 | 0.80 | NM_198235,NM_002933,NM_198234,NM_198232 | Ribonuclease pancreatic precursor (EC 3.1.27.5) (RNase 1) (RNase A) (RNase UpI-1) (RIB-1) (HP-RNase). [Source:Uniprot/SWISSPROT;Acc:P07998] |
| - | 0.07 | -1.10 | 0.34 | - | 14 kDa protein [Source:IPI;Acc:IPI00514536] |
| NP_872310.1 | 0.54 | -1.10 | 0.11 | NM_182504 | Williams-Beuren syndrome critical region 28 [Source:RefSeq_peptide;Acc:NP_872310] |
| MRO | 0.17 | -1.10 | -0.09 | NM_031939 | maestro [Source:RefSeq_peptide;Acc:NP_114145] |
| PTPRU | 0.10 | -1.10 | 0.03 | NM_005704,NM_133177,NM_133178 | Receptor-type tyrosine-protein phosphatase U precursor (EC 3.1.3.48) (R-PTP-U) (Protein-tyrosine phosphatase J) (PTP-J) (Pancreatic carcinoma phosphatase 2) (PCP-2). [Source:Uniprot/SWISSPROT;Acc:Q92729] |
| - | 0.55 | -1.10 | 1.23 | NM_178429 | - |
| KLHL1 | 1.15 | -1.10 | -0.25 | NM_020866 | Kelch-like protein 1. [Source:Uniprot/SWISSPROT;Acc:Q9NR64] |
| ZBTB3 | -0.48 | -1.09 | 0.52 | NM_024784 | Zinc finger and BTB domain containing protein 3. [Source:Uniprot/SWISSPROT;Acc:Q9H5J0] |
| - | 0.01 | -1.09 | -0.26 | XM_498699 | - |
| - | -0.23 | -1.09 | 2.53 | - | Non-protein coding transcript |
| - | 0.30 | -1.09 | 0.11 | XM_497908 | - |
| C21orf81 | -0.25 | -1.09 | -0.07 | XM_496266,NM_153750 | - |
| C2orf11 | 0.02 | -1.09 | 0.09 | NM_144629 | - |
| NP_001007535.1 | -0.34 | -1.08 | -0.47 | NM_001007534 | - |
| RABEP2 | 0.36 | -1.08 | -0.44 | NM_024816 | Rab GTPase binding effector protein 2 (Rabaptin-5beta). [Source:Uniprot/SWISSPROT;Acc:Q9H5N1] |
| - | 0.18 | -1.08 | 0.98 | NM_173571 | - |
| - | 0.56 | -1.08 | 0.26 | XM_497354 | - |
| NP_076983.1 | 0.40 | -1.08 | 0.10 | NM_024078 | - |
| NP_976054.1 | -0.43 | -1.08 | -0.47 | NM_203309 | - |
| - | 0.14 | -1.08 | -0.29 | - | Hypothetical protein |
| - | 2.20 | -1.08 | 0.70 | - | Hypothetical protein |
| - | 0.16 | -1.07 | -0.28 | XM_496637 | - |
| - | -0.04 | -1.07 | 0.29 | - | Hypothetical protein |
| RCOR1 | -0.16 | -1.07 | -0.14 | NM_015156 | REST corepressor 1 [Source:RefSeq_peptide;Acc:NP_055971] |
| GPATC4 | 0.41 | -1.07 | 0.54 | NM_015590,NM_182679,NM_017725 | G patch domain containing 4 protein isoform 1 [Source:RefSeq_peptide;Acc:NP_056405] |
| Q5VY54_HUMAN | 0.39 | -1.07 | 0.54 | - | PREDICTED: similar to family with sequence similarity 35, member A [Source:RefSeq_peptide;Acc:XP_290185] |
| PIK3C2A | -0.20 | -1.07 | -0.24 | NM_002645 | phosphoinositide-3-kinase, class 2, alpha polypeptide [Source:RefSeq_peptide;Acc:NP_002636] |
| - | -0.48 | -1.07 | -0.20 | XM_378925 | - |
| PPBP | 0.55 | -1.07 | 0.29 | NM_002704 | Platelet basic protein precursor (PBP) (Small inducible cytokine B7) (CXCL7) (Leukocyte-derived growth factor) (LDGF) (Macrophage-derived growth factor) (MDGF) [Contains: Connective-tissue activating peptide III (CTAP-III) (Low-affinity platelet factor IV |
| CRYAA | 0.42 | -1.07 | 1.61 | NM_000394 | Alpha crystallin A chain (Heat-shock protein beta-4) (HspB4). [Source:Uniprot/SWISSPROT;Acc:P02489] |
| NP_001005356.1 | 0.45 | -1.06 | 1.46 | NM_001005356,NM_001004053,NM_001005357,NM_207513 | protein expressed in prostate, ovary, testis, and placenta 14 isoform POTE-14C [Source:RefSeq_peptide;Acc:NP_001005357] |
| TBN | -0.13 | -1.06 | 1.31 | NM_138572 | taube nuss [Source:RefSeq_peptide;Acc:NP_612639] |
| - | 0.11 | -1.06 | 0.43 | - | 18 kDa protein [Source:IPI;Acc:IPI00414210] |
| - | -0.08 | -1.06 | 0.12 | - | Hypothetical protein |
| DGAT1 | 0.08 | -1.06 | -0.45 | NM_012079 | Diacylglycerol O-acyltransferase 1 (EC 2.3.1.20) (Diglyceride acyltransferase) (ACAT related gene product 1). [Source:Uniprot/SWISSPROT;Acc:O75907] |
| - | -0.11 | -1.06 | -0.40 | XM_498964 | - |
| - | 0.28 | -1.06 | -0.16 | XM_372028,XM_372030 | - |
| - | 1.05 | -1.06 | -0.16 | - | 10 kDa protein [Source:IPI;Acc:IPI00478322] |
| - | 0.03 | -1.06 | -0.22 | XM_498409 | - |
| ASB11 | 0.46 | -1.05 | -0.18 | NM_001012428 | Ankyrin repeat and SOCS box protein 11 (ASB-11). [Source:Uniprot/SWISSPROT;Acc:Q8WXH4] |
| - | 1.59 | -1.05 | -0.14 | - | Hypothetical protein |
| SRGAP1 | -0.04 | -1.05 | -0.22 | NM_020762 | SLIT-ROBO Rho GTPase activating protein 1 (srGAP1) (Rho-GTPase- activating protein 13). [Source:Uniprot/SWISSPROT;Acc:Q7Z6B7] |
| Q9P1B8_HUMAN | 1.00 | -1.05 | 0.17 | - | - |
| SLC1A2 | 0.29 | -1.05 | -0.36 | NM_004171 | Excitatory amino acid transporter 2 (Sodium-dependent glutamate/aspartate transporter 2). [Source:Uniprot/SWISSPROT;Acc:P43004] |
| NP_853654.1 | 0.66 | -1.05 | -0.18 | NM_181623 | keratin associated protein 15-1 [Source:RefSeq_peptide;Acc:NP_853654] |
| CREBL1 | 0.20 | -1.05 | 1.09 | NM_004381 | Cyclic-AMP-dependent transcription factor ATF-6 beta (Activating transcription factor 6 beta) (ATF6-beta) (cAMP responsive element binding protein-like 1) (cAMP response element binding protein-related protein) (Creb-rp) (G13 protein). [Source:Uniprot/SWI |
| NP_872432.1 | -0.08 | -1.04 | 0.89 | NM_182626 | - |
| NP_689538.1 | 0.29 | -1.04 | -0.17 | NM_152325 | - |
| - | 0.50 | -1.04 | 0.02 | - | Hypothetical protein |
| OR7C2 | 0.29 | -1.04 | 0.17 | NM_012377 | Olfactory receptor 7C2 (Olfactory receptor 19-18) (OR19-18). [Source:Uniprot/SWISSPROT;Acc:O60412] |
| - | 0.48 | -1.04 | 2.14 | XM_168055 | - |
| - | 0.30 | -1.04 | 0.97 | NM_001004054,NM_001005364,NM_001005359 | - |
| OR5AS1 | 0.75 | -1.04 | -0.34 | NM_001001921 | Olfactory receptor 5AS1. [Source:Uniprot/SWISSPROT;Acc:Q8N127] |
| - | -0.41 | -1.04 | -0.22 | XM_498866 | - |
| - | 0.01 | -1.04 | -0.26 | NM_018917,NM_032053 | - |
| NP_001001702.1 | 0.01 | -1.04 | 0.59 | NM_001001702 | - |
| OR1D4 | 1.24 | -1.03 | -0.43 | NM_014566 | Olfactory receptor 1D5 (Olfactory receptor 17-31) (OR17-31). [Source:Uniprot/SWISSPROT;Acc:P58170] |
| Q96HM8_HUMAN | 0.38 | -1.03 | -0.07 | - | - |
| ZNF384 | 0.11 | -1.03 | 0.11 | NM_133476 | Zinc finger protein 384 (Nuclear matrix transcription factor 4) (CAG repeat protein 1). [Source:Uniprot/SWISSPROT;Acc:Q8TF68] |
| - | 0.81 | -1.03 | 0.68 | XM_374069 | - |
| - | 0.02 | -1.03 | -0.10 | XM_498761 | - |
| AQP6 | 0.55 | -1.03 | 0.17 | NM_053286,NM_001652 | Aquaporin 6 (Aquaporin-2 like) (hKID). [Source:Uniprot/SWISSPROT;Acc:Q13520] |
| Q9UI58_HUMAN | 0.13 | -1.03 | -0.01 | - | - |
| TAGAP | 0.64 | -1.03 | -0.32 | NM_054114,NM_152133 | T-cell activation Rho GTPase-activating protein isoform a [Source:RefSeq_peptide;Acc:NP_687034] |
| BOK | 0.11 | -1.02 | -0.03 | NM_032515 | Bcl-2 related ovarian killer protein (Hbok). [Source:Uniprot/SWISSPROT;Acc:Q9UMX3] |
| KRT13 | 0.71 | -1.02 | 0.90 | NM_153490,NM_002274 | Keratin, type I cytoskeletal 13 (Cytokeratin 13) (K13) (CK 13). [Source:Uniprot/SWISSPROT;Acc:P13646] |
| XP_371592.1 | 0.72 | -1.02 | -0.39 | XM_371592 | PREDICTED: similar to RIKEN cDNA D630023F18 [Source:RefSeq_peptide;Acc:XP_371592] |
| KCNJ15 | -0.05 | -1.02 | 0.62 | NM_170737,NM_002243,NM_170736 | ATP-sensitive inward rectifier potassium channel 15 (Potassium channel, inwardly rectifying, subfamily J, member 15) (Inward rectifier K(+) channel Kir4.2) (Kir1.3). [Source:Uniprot/SWISSPROT;Acc:Q99712] |
| Q86XG3_HUMAN | -0.38 | -1.02 | 0.13 | - | DNAJC13 protein (Fragment). [Source:Uniprot/SPTREMBL;Acc:Q86XG3] |
| KCNK13 | -0.18 | -1.02 | 0.82 | NM_022054 | Potassium channel subfamily K member 13 (Tandem pore domain halothane inhibited potassium channel 1) (THIK-1). [Source:Uniprot/SWISSPROT;Acc:Q9HB14] |
| IFNA1 | -0.33 | -1.02 | 0.19 | NM_024013 | Interferon alpha-1/13 precursor (Interferon alpha-D) (LeIF D). [Source:Uniprot/SWISSPROT;Acc:P01562] |
| NP_001012391.1 | 0.53 | -1.02 | -0.29 | XM_375308,XM_496134 | similar to FLJ12363 protein [Source:RefSeq_peptide;Acc:NP_001012391] |
| - | -0.21 | -1.02 | -0.07 | NM_003318 | - |
| - | 1.41 | -1.02 | 0.29 | NM_175884 | - |
| EP300 | -0.26 | -1.02 | -0.25 | NM_001429 | E1A-associated protein p300 (EC 2.3.1.48). [Source:Uniprot/SWISSPROT;Acc:Q09472] |
| MFNG | 0.71 | -1.02 | 0.67 | NM_002405 | Beta-1,3-N-acetylglucosaminyltransferase manic fringe (EC 2.4.1.222) (O-fucosylpeptide 3-beta-N-acetylglucosaminyltransferase). [Source:Uniprot/SWISSPROT;Acc:O00587] |
| RAB9B | -0.17 | -1.02 | -0.46 | NM_016370 | Ras-related protein Rab-9B (Rab-9L) (RAB9-like protein). [Source:Uniprot/SWISSPROT;Acc:Q9NP90] |
| NP_872295.1 | 0.86 | -1.02 | 0.76 | NM_182489 | STRA8 [Source:RefSeq_peptide;Acc:NP_872295] |
| HTR5A | -0.21 | -1.02 | -0.34 | NM_024012 | 5-hydroxytryptamine 5A receptor (5-HT-5A) (Serotonin receptor 5A) (5- HT-5). [Source:Uniprot/SWISSPROT;Acc:P47898] |
| - | 1.39 | -1.02 | -0.27 | - | Hypothetical protein |
| NP_689983.1 | -0.20 | -1.02 | -0.35 | NM_152770 | - |
| - | -0.21 | -1.01 | 0.37 | XM_376616,XM_379844,XM_376615,XM_379843 | - |
| PBXIP1 | -0.27 | -1.01 | -0.39 | NM_020524 | pre-B-cell leukemia transcription factor interacting protein 1 [Source:RefSeq_peptide;Acc:NP_065385] |
| Q14180_HUMAN | -0.42 | -1.01 | -0.47 | - | NF1 gene homologue protein (Fragment). [Source:Uniprot/SPTREMBL;Acc:Q14180] |
| TTC6_HUMAN | 0.81 | -1.01 | -0.25 | NM_001007795 | Tetratricopeptide repeat protein 6 (TPR repeat protein 6). [Source:Uniprot/SWISSPROT;Acc:Q86TZ1] |
| NXF3 | 0.59 | -1.01 | -0.46 | NM_022052 | Nuclear RNA export factor 3 (TAP-like protein 3) (TAPL-3). [Source:Uniprot/SWISSPROT;Acc:Q9H4D5] |
| - | -0.28 | -1.01 | -0.45 | - | Non-protein coding transcript |
| - | 0.66 | -1.01 | -0.09 | - | Non-protein coding transcript |
| - | 0.39 | -1.01 | 0.06 | - | Non-protein coding transcript |
| Q96J52_HUMAN | -0.36 | -1.01 | -0.30 | - | Vang-like 1. [Source:Uniprot/SPTREMBL;Acc:Q96J52] |
| HAVCR1 | 0.52 | -1.01 | -0.20 | NM_012206 | hepatitis A virus cellular receptor 1 [Source:RefSeq_peptide;Acc:NP_036338] |
| - | 0.18 | -1.01 | 0.24 | XM_497445 | - |
| - | 0.14 | -1.01 | 0.00 | NM_001005354,NM_018457,NM_001005355 | - |
| - | -0.22 | -1.01 | -0.04 | XM_172968 | - |
| Q8N9N3_HUMAN | 0.07 | -1.00 | 1.88 | - | - |
| TAS2R41 | -0.07 | -1.00 | 0.09 | NM_176883 | Taste receptor type 2 member 41 (T2R41) (T2R59). [Source:Uniprot/SWISSPROT;Acc:P59536] |
| AGXT2L1 | 0.72 | -1.00 | -0.32 | NM_031279 | alanine-glyoxylate aminotransferase 2-like 1 [Source:RefSeq_peptide;Acc:NP_112569] |
| APOC3 | 0.67 | -1.00 | -0.35 | NM_000040,XM_496537 | Apolipoprotein C-III precursor (Apo-CIII) (ApoC-III). [Source:Uniprot/SWISSPROT;Acc:P02656] |
| OR7C1 | 0.69 | -1.00 | -0.27 | NM_198944 | Olfactory receptor 7C1 (Olfactory receptor TPCR86). [Source:Uniprot/SWISSPROT;Acc:O76099] |
| - | 0.67 | -1.00 | -0.28 | - | 32 kDa protein [Source:IPI;Acc:IPI00145278] |
| - | 0.61 | -1.00 | -0.41 | - | 15 kDa protein [Source:IPI;Acc:IPI00478952] |
| NP_775929.1 | 0.46 | -0.99 | -0.14 | NM_173658 | zinc finger protein 660 [Source:RefSeq_peptide;Acc:NP_775929] |
| Q96NR0_HUMAN | -0.33 | -0.99 | -0.31 | - | - |
| TAS2R50 | -0.46 | -0.99 | 0.12 | NM_176890 | Taste receptor type 2 member 50 (T2R50) (T2R51). [Source:Uniprot/SWISSPROT;Acc:P59544] |
| CD226 | 0.56 | -0.99 | -0.32 | NM_006566 | CD226 antigen precursor (DNAX accessory molecule-1) (DNAM-1). [Source:Uniprot/SWISSPROT;Acc:Q15762] |
| CRYAB | 0.04 | -0.99 | -0.34 | NM_001885 | Alpha crystallin B chain (Alpha(B)-crystallin) (Rosenthal fiber component) (Heat-shock protein beta-5) (HspB5). [Source:Uniprot/SWISSPROT;Acc:P02511] |
| - | 0.43 | -0.99 | -0.02 | - | Non-protein coding transcript |
| - | 1.06 | -0.99 | 1.32 | - | Splice Isoform 3 of GTPase activating Rap/Ran-GAP domain-like 1 [Source:IPI;Acc:IPI00456721] |
| TTTY11 | 0.04 | -0.99 | -0.47 | NR_001548 | Transcript Y 11 protein. [Source:Uniprot/SWISSPROT;Acc:Q9BZ99] |
| - | 0.24 | -0.99 | 0.03 | XM_498893 | - |
| OPRM1 | 0.93 | -0.98 | -0.25 | NM_001008505,NM_001008503,NM_001008504,NM_000914 | Mu-type opioid receptor (MOR-1). [Source:Uniprot/SWISSPROT;Acc:P35372] |
| - | -0.37 | -0.98 | 0.70 | XM_379395 | - |
| GPHA2 | 0.36 | -0.98 | 0.39 | NM_130769 | Glycoprotein hormone alpha 2 precursor (Thyrostimulin alpha subunit). [Source:Uniprot/SWISSPROT;Acc:Q96T91] |
| NP_689661.1 | -0.19 | -0.98 | -0.03 | NM_152448 | - |
| OR4E2 | 1.31 | -0.98 | 2.13 | NM_001001912 | Olfactory receptor 4E2. [Source:Uniprot/SWISSPROT;Acc:Q8NGC2] |
| - | -0.34 | -0.98 | -0.49 | - | - |
| CYP7A1 | 0.31 | -0.98 | -0.25 | NM_000780 | Cytochrome P450 7A1 (Cholesterol 7-alpha-monooxygenase) (CYPVII) (EC 1.14.13.17) (Cholesterol 7-alpha-hydroxylase). [Source:Uniprot/SWISSPROT;Acc:P22680] |
| PCQAP | 1.58 | -0.98 | 0.97 | NM_001003891,NM_015889 | Positive cofactor 2 glutamine/Q-rich-associated protein (PC2 glutamine/Q-rich-associated protein) (TPA-inducible gene-1) (TIG-1) (Activator-recruited cofactor 105 kDa component) (ARC105) (CTG repeat protein 7a). [Source:Uniprot/SWISSPROT;Acc:Q96RN5] |
| - | 0.00 | -0.98 | 0.08 | XM_498484,XM_498482,XM_498480,XM_498479 | - |
| - | 0.29 | -0.98 | -0.38 | - | Hypothetical protein |
| ARMC4 | 0.05 | -0.98 | -0.28 | NM_018076 | armadillo repeat containing 4 [Source:RefSeq_peptide;Acc:NP_060546] |
| - | 0.00 | -0.98 | 0.62 | - | Hypothetical protein |
| TIMM8A | -0.42 | -0.98 | 0.13 | NM_004085 | Mitochondrial import inner membrane translocase subunit TIM8 A (Deafness dystonia protein 1) (X-linked deafness dystonia protein). [Source:Uniprot/SWISSPROT;Acc:O60220] |
| MRGRD_HUMAN | 0.59 | -0.98 | -0.26 | NM_198923 | Mas-related G-protein coupled receptor member D (Beta-alanine receptor) (G-protein coupled receptor TGR7). [Source:Uniprot/SWISSPROT;Acc:Q8TDS7] |
| NP_542396.3 | -0.02 | -0.98 | 0.74 | NM_080665 | - |
| - | 1.56 | -0.98 | 0.09 | XM_498553 | - |
| XM_496607.1 | 0.07 | -0.98 | -0.15 | XM_496607 | - |
| ARID5A | 0.39 | -0.98 | 0.84 | NM_212481,NM_006673 | AT rich interactive domain 5A isoform 1 [Source:RefSeq_peptide;Acc:NP_997646] |
| NP_660280.1 | -0.06 | -0.98 | 0.01 | NM_145237 | similar to RNA polymerase I transcription factor RRN3 [Source:RefSeq_peptide;Acc:NP_660280] |
| - | 0.50 | -0.97 | 0.18 | - | Human betaretrovirus isolate Es177-3 env and sag genes, partial cds |
| hlucCP+ | 0.02 | -0.97 | -0.46 | - | hlucCP+ luciferase reporter protein |
| NPY5R | -0.10 | -0.97 | -0.37 | NM_006174 | Neuropeptide Y receptor type 5 (NPY5-R) (NPY-Y5 receptor) (Y5 receptor) (NPYY5). [Source:Uniprot/SWISSPROT;Acc:Q15761] |
| OR2A42 | 0.17 | -0.97 | -0.42 | NM_001001802 | Olfactory receptor 2A1. [Source:Uniprot/SWISSPROT;Acc:Q8NGT9] |
| GSTO2_HUMAN | 0.50 | -0.97 | -0.43 | NM_183239 | Glutathione transferase omega 2 (EC 2.5.1.18). [Source:Uniprot/SWISSPROT;Acc:Q9H4Y5] |
| DNCL2B | 0.39 | -0.97 | 0.41 | NM_130897 | Dynein light chain 2B, cytoplasmic. [Source:Uniprot/SWISSPROT;Acc:Q8TF09] |
| WDFY3 | -0.47 | -0.97 | 0.14 | NM_014991,NM_178585 | WD repeat and FYVE domain containing 3 isoform 2 [Source:RefSeq_peptide;Acc:NP_848698] |
| Q96EM6_HUMAN | -0.30 | -0.97 | 0.28 | - | - |
| ENK11_HUMAN | -0.21 | -0.97 | 0.26 | - | HERV-K_22q11.21 provirus ancestral Env polyprotein (Envelope polyprotein) (HERV-K101 envelope protein) [Contains: Surface protein (SU); Transmembrane protein (TM)]. [Source:Uniprot/SWISSPROT;Acc:P61566] |
| C6orf192 | 0.35 | -0.97 | 0.12 | NM_052831 | - |
| KBTBD10 | 0.62 | -0.97 | 0.03 | NM_006063 | Kelch repeat and BTB domain containing protein 10 (Kelch-related protein 1) (Kel-like protein 23) (Sarcosin). [Source:Uniprot/SWISSPROT;Acc:O60662] |
| SLC5A11 | -0.04 | -0.96 | 0.99 | NM_052944 | solute carrier family 5 (sodium/glucose cotransporter), member 11 [Source:RefSeq_peptide;Acc:NP_443176] |
| C21orf123 | 0.67 | -0.96 | 0.32 | NM_199175 | - |
| NP_872384.1 | 0.85 | -0.96 | 0.59 | NM_182578 | - |
| PSD | -0.35 | -0.96 | -0.32 | NM_002779 | pleckstrin and Sec7 domain containing [Source:RefSeq_peptide;Acc:NP_002770] |
| - | 0.06 | -0.96 | -0.34 | XM_496620 | - |
| - | 0.09 | -0.96 | -0.17 | - | Hypothetical protein |
| CCT6B | 0.28 | -0.96 | 0.45 | NM_006584 | T-complex protein 1, zeta-2 subunit (TCP-1-zeta-2) (CCT-zeta-2) (TCP- 1-zeta-like) (CCT-zeta-like) (Testis-specific Tcp20) (Testis-specific protein TSA303). [Source:Uniprot/SWISSPROT;Acc:Q92526] |
| WNT8B | 0.54 | -0.96 | 0.68 | NM_003393 | Wnt-8b protein precursor. [Source:Uniprot/SWISSPROT;Acc:Q93098] |
| Q96ME0_HUMAN | -0.26 | -0.96 | -0.18 | NM_152531 | - |
| PDE6H | -0.27 | -0.96 | -0.28 | NM_006205 | Retinal cone rhodopsin-sensitive cGMP 3',5'-cyclic phosphodiesterase gamma-subunit (EC 3.1.4.17) (GMP-PDE gamma). [Source:Uniprot/SWISSPROT;Acc:Q13956] |
| EPS8L1 | -0.24 | -0.96 | 0.58 | NM_133180 | epidermal growth factor receptor pathway substrate 8-like protein 1 isoform c [Source:RefSeq_peptide;Acc:NP_631943] |
| - | 0.59 | -0.96 | -0.03 | - | Hypothetical protein |
| - | -0.29 | -0.96 | 0.82 | XM_376243 | - |
| - | -0.47 | -0.96 | 0.41 | - | 27 kDa protein [Source:IPI;Acc:IPI00412350] |
| - | -0.18 | -0.96 | -0.45 | XM_498844 | - |
| NFATC2 | -0.25 | -0.95 | 1.10 | NM_012340,NM_173091 | Nuclear factor of activated T-cells, cytoplasmic 2 (T cell transcription factor NFAT1) (NFAT pre-existing subunit) (NF-ATp). [Source:Uniprot/SWISSPROT;Acc:Q13469] |
| Q9H399_HUMAN | 1.23 | -0.95 | 0.66 | - | - |
| HUS1B | -0.09 | -0.95 | -0.25 | NM_148959 | HUS1 checkpoint protein B [Source:RefSeq_peptide;Acc:NP_683762] |
| ROXAN_HUMAN | -0.05 | -0.95 | -0.35 | NM_017590 | Rotavirus 'X' associated non-structural protein (RoXaN). [Source:Uniprot/SWISSPROT;Acc:Q9UGR2] |
| - | 1.19 | -0.95 | 0.59 | - | Hypothetical protein |
| ATF7IP2 | -0.28 | -0.95 | -0.48 | NM_024997 | activating transcription factor 7 interacting protein 2 [Source:RefSeq_peptide;Acc:NP_079273] |
| - | 0.95 | -0.95 | -0.37 | NM_001001789 | - |
| - | 0.54 | -0.95 | -0.14 | - | - |
| C6orf146 | -0.40 | -0.95 | -0.15 | NM_173563 | - |
| NP_940914.1 | 0.25 | -0.95 | 0.02 | NM_198512 | diacylglycerol O-acyltransferase 2 like 6 [Source:RefSeq_peptide;Acc:NP_940914] |
| - | 0.93 | -0.94 | 0.32 | NM_001001930,NM_005036,NM_001001928,NM_001001929 | Hypothetical protein |
| FGFRL1 | 0.10 | -0.94 | 0.48 | NM_001004356,NM_021923,NM_001004358 | fibroblast growth factor receptor-like 1 precursor [Source:RefSeq_peptide;Acc:NP_001004358] |
| ELMOD1 | 0.05 | -0.94 | -0.19 | NM_018712 | ELMO domain containing 1 [Source:RefSeq_peptide;Acc:NP_061182] |
| SPATA9 | -0.32 | -0.94 | -0.20 | NM_031952 | spermatogenesis associated 9 [Source:RefSeq_peptide;Acc:NP_775496] |
| FLCN | 0.43 | -0.94 | 0.31 | NM_144997 | folliculin isoform 1 [Source:RefSeq_peptide;Acc:NP_659434] |
| SLC38A4 | -0.29 | -0.94 | -0.41 | - | solute carrier family 38, member 4 [Source:RefSeq_peptide;Acc:NP_060488] |
| PFKL | -0.47 | -0.94 | -0.27 | NM_001002021 | 6-phosphofructokinase, liver type (EC 2.7.1.11) (Phosphofructokinase 1) (Phosphohexokinase) (Phosphofructo-1-kinase isozyme B) (PFK-B). [Source:Uniprot/SWISSPROT;Acc:P17858] |
| - | -0.11 | -0.94 | -0.27 | NM_007333 | - |
| NP_997388.1 | 0.71 | -0.94 | -0.26 | NM_207505 | - |
| - | 0.81 | -0.94 | -0.02 | XM_372676 | - |
| NP_079006.1 | -0.02 | -0.94 | 0.68 | NM_024730 | - |
| - | -0.12 | -0.94 | -0.46 | - | 20 kDa protein [Source:IPI;Acc:IPI00477153] |
| NP_776156.1 | 0.07 | -0.94 | -0.36 | NM_173795 | - |
| - | 0.14 | -0.94 | -0.04 | XM_497623 | 47 kDa protein [Source:IPI;Acc:IPI00552494] |
| NOV | 0.54 | -0.94 | 1.45 | NM_002514 | NOV protein homolog precursor (NovH) (Nephroblastoma overexpressed gene protein homolog). [Source:Uniprot/SWISSPROT;Acc:P48745] |
| Q5QNZ1_HUMAN | -0.23 | -0.94 | 0.60 | NM_174896 | - |
| MME | -0.20 | -0.93 | -0.26 | NM_007289,NM_000902,NM_007287,NM_007288 | Neprilysin (EC 3.4.24.11) (Neutral endopeptidase) (NEP) (Enkephalinase) (Common acute lymphocytic leukemia antigen) (CALLA) (Neutral endopeptidase 24.11) (CD10). [Source:Uniprot/SWISSPROT;Acc:P08473] |
| - | -0.11 | -0.93 | -0.21 | - | Hypothetical protein |
| HRH1 | 0.12 | -0.93 | -0.19 | NM_000861 | Histamine H1 receptor. [Source:Uniprot/SWISSPROT;Acc:P35367] |
| CORIN | 0.11 | -0.93 | -0.42 | NM_006587 | Atrial natriuteric peptide-converting enzyme (EC 3.4.21.-) (pro-ANP- converting enzyme) (Corin) (Heart specific serine proteinase ATC2). [Source:Uniprot/SWISSPROT;Acc:Q9Y5Q5] |
| NP_078871.1 | 0.64 | -0.93 | -0.41 | NM_024595 | - |
| WDR31 | 0.45 | -0.93 | -0.01 | NM_145241 | WD-repeat protein 31. [Source:Uniprot/SWISSPROT;Acc:Q8NA23] |
| ROPN1 | 0.87 | -0.93 | -0.19 | XM_042178 | AKAP-binding sperm protein ropporin [Source:RefSeq_peptide;Acc:NP_001012337] |
| GBP6 | 1.36 | -0.93 | -0.14 | NM_198460 | guanylate binding protein family, member 6 [Source:RefSeq_peptide;Acc:NP_940862] |
| MYPN | -0.16 | -0.93 | -0.48 | - | myopalladin [Source:RefSeq_peptide;Acc:NP_115967] |
| XP_352848.2 | -0.18 | -0.93 | 0.07 | XM_352847 | PREDICTED: hypothetical protein XP_352847 [Source:RefSeq_peptide;Acc:XP_352848] |
| - | 0.12 | -0.93 | 0.83 | - | Non-protein coding transcript |
| - | 0.03 | -0.92 | 0.51 | XM_498515 | - |
| - | 1.33 | -0.92 | 1.01 | XM_059051 | - |
| NAPG | 0.12 | -0.92 | 0.46 | NM_003826 | Gamma-soluble NSF attachment protein (SNAP-gamma) (N-ethylmaleimide- sensitive factor attachment protein, gamma). [Source:Uniprot/SWISSPROT;Acc:Q99747] |
| USP28 | 0.45 | -0.92 | 0.33 | NM_020886 | Ubiquitin carboxyl-terminal hydrolase 28 (EC 3.1.2.15) (Ubiquitin thiolesterase 28) (Ubiquitin-specific processing protease 28) (Deubiquitinating enzyme 28). [Source:Uniprot/SWISSPROT;Acc:Q96RU2] |
| - | -0.22 | -0.92 | -0.38 | XM_378562 | - |
| IL1R1 | 0.08 | -0.92 | 0.70 | NM_000877 | Interleukin-1 receptor, type I precursor (IL-1R-1) (IL-1R-alpha) (P80) (Antigen CD121a). [Source:Uniprot/SWISSPROT;Acc:P14778] |
| - | -0.17 | -0.92 | 0.27 | XM_166971 | - |
| LIRA4_HUMAN | 0.12 | -0.92 | 0.04 | NM_012276 | Leukocyte immunoglobulin-like receptor subfamily A member 4 precursor (Immunoglobulin-like transcript 7) (ILT-7) (CD85g antigen). [Source:Uniprot/SWISSPROT;Acc:P59901] |
| ADIPO_HUMAN | 0.45 | -0.92 | -0.18 | NM_004797 | Adiponectin precursor (Adipocyte, C1q and collagen domain containing protein) (30 kDa adipocyte complement-related protein) (ACRP30) (Adipose most abundant gene transcript 1) (apM-1) (Gelatin-binding protein). [Source:Uniprot/SWISSPROT;Acc:Q15848] |
| ABHD10 | -0.30 | -0.92 | -0.09 | NM_018394 | abhydrolase domain containing 10 [Source:RefSeq_peptide;Acc:NP_060864] |
| NP_997213.1 | 0.11 | -0.92 | 0.39 | NM_207330 | - |
| - | 0.68 | -0.91 | 0.27 | XM_379408 | - |
| - | 1.20 | -0.91 | -0.08 | XM_498627 | - |
| - | 0.16 | -0.91 | 1.84 | - | Gamma-carboxylase-like domain containing protein |
| - | 0.93 | -0.91 | 0.27 | NM_182554 | - |
| - | 1.47 | -0.91 | 1.13 | - | Hypothetical protein |
| - | 0.38 | -0.91 | -0.17 | - | 16 kDa protein [Source:IPI;Acc:IPI00089555] |
| NP_001004760.1 | 0.64 | -0.91 | 0.63 | NM_001004760 | olfactory receptor, family 51, subfamily V, member 1 [Source:RefSeq_peptide;Acc:NP_001004760] |
| IRX1 | 0.28 | -0.91 | -0.48 | NM_024337,XM_380171 | Iroquois-class homeodomain protein IRX-1 (Iroquois homeobox protein 1) (Homeodomain protein IRXA1). [Source:Uniprot/SWISSPROT;Acc:P78414] |
| - | 1.00 | -0.91 | -0.38 | XM_496030 | - |
| - | 0.00 | -0.91 | -0.21 | XM_211108 | - |
| - | 0.65 | -0.91 | -0.48 | XM_496200 | - |
| - | 0.47 | -0.91 | -0.33 | - | Homo sapiens microRNA let-7b stem-loop |
| DNM3 | 0.88 | -0.90 | 0.51 | NM_015569 | Dynamin 3 (EC 3.6.5.5) (Dynamin, testicular) (T-dynamin). [Source:Uniprot/SWISSPROT;Acc:Q9UQ16] |
| - | 0.00 | -0.90 | 0.40 | - | - |
| C10orf79 | -0.18 | -0.90 | -0.35 | NM_025145 | - |
| NP_997365.1 | 0.49 | -0.90 | -0.41 | NM_207482 | - |
| NP_277051.3 | 0.48 | -0.90 | 0.66 | - | protein kinase NYD-SP25 isoform 3 [Source:RefSeq_peptide;Acc:NP_001001875] |
| CHD2 | -0.30 | -0.90 | -0.44 | NM_001271 | Chromodomain-helicase-DNA-binding protein 2 (CHD-2). [Source:Uniprot/SWISSPROT;Acc:O14647] |
| SPRR3 | 0.22 | -0.90 | 0.84 | NM_005416 | Small proline-rich protein 3 (Cornifin beta) (Esophagin) (22 kDa pancornulin). [Source:Uniprot/SWISSPROT;Acc:Q9UBC9] |
| Q9UJN8_HUMAN | -0.17 | -0.90 | -0.31 | - | OTTHUMP00000016179 (Fragment). [Source:Uniprot/SPTREMBL;Acc:Q9UJN8] |
| NP_001009991.1 | 0.40 | -0.90 | 0.15 | - | synaptotagmin-like 3 [Source:RefSeq_peptide;Acc:NP_001009991] |
| - | 0.87 | -0.90 | -0.18 | XM_379594 | - |
| C1QDC1 | 0.78 | -0.90 | -0.05 | NM_001002259,NM_032156,NM_023925 | C1q domain containing 1 isoform 2 [Source:RefSeq_peptide;Acc:NP_076414] |
| NP_777560.1 | -0.33 | -0.90 | -0.17 | NM_174900 | zinc finger protein 42 [Source:RefSeq_peptide;Acc:NP_777560] |
| SLC30A2 | 0.14 | -0.90 | -0.02 | NM_001004434,NM_032513 | Zinc transporter 2 (ZnT-2) (Solute carrier family 30, member 2). [Source:Uniprot/SWISSPROT;Acc:Q9BRI3] |
| - | 0.00 | -0.90 | -0.29 | XM_374162 | - |
| ZNFN1A2 | -0.10 | -0.89 | 0.16 | - | Zinc finger protein Helios. [Source:Uniprot/SWISSPROT;Acc:Q9UKS7] |
| LETM2 | 0.67 | -0.89 | 0.03 | NM_144652 | leucine zipper-EF-hand containing transmembrane protein 2 [Source:RefSeq_peptide;Acc:NP_653253] |
| Q8WYZ6_HUMAN | -0.04 | -0.89 | 0.22 | - | - |
| - | -0.28 | -0.89 | 0.37 | XM_373547 | - |
| ICBR_HUMAN | -0.19 | -0.89 | -0.31 | NM_021571 | Caspase-1 inhibitor Iceberg (UNQ5804/PRO19611). [Source:Uniprot/SWISSPROT;Acc:P57730] |
| CHDH | 0.34 | -0.89 | 0.41 | NM_018397 | Choline dehydrogenase, mitochondrial precursor (EC 1.1.99.1) (CHD) (CDH). [Source:Uniprot/SWISSPROT;Acc:Q8NE62] |
| - | -0.24 | -0.89 | 0.21 | XM_373518 | - |
| - | 0.37 | -0.89 | 0.31 | XM_378434 | - |
| - | -0.41 | -0.89 | -0.24 | XM_496713 | - |
| - | -0.04 | -0.89 | -0.06 | XM_498943 | - |
| - | -0.18 | -0.89 | 0.28 | NM_001010876 | - |
| TAS2R16 | -0.25 | -0.89 | 0.55 | NM_016945 | Taste receptor type 2 member 16 (T2R16). [Source:Uniprot/SWISSPROT;Acc:Q9NYV7] |
| - | 0.31 | -0.89 | 0.09 | XM_379189 | - |
| - | -0.07 | -0.89 | -0.27 | - | 13 kDa protein [Source:IPI;Acc:IPI00478158] |
| STAG1 | 0.99 | -0.89 | -0.14 | NM_005862 | Cohesin subunit SA-1 (Stromal antigen 1) (SCC3 homolog 1). [Source:Uniprot/SWISSPROT;Acc:Q8WVM7] |
| - | -0.04 | -0.89 | -0.04 | - | 135 kDa protein [Source:IPI;Acc:IPI00479239] |
| MANS1_HUMAN | 0.60 | -0.89 | -0.31 | NM_018050 | MANSC domain containing protein 1 precursor (UNQ316/PRO361). [Source:Uniprot/SWISSPROT;Acc:Q9H8J5] |
| - | -0.26 | -0.89 | 0.06 | XM_498985 | - |
| OR8H2 | 0.25 | -0.89 | 1.40 | NM_001005200 | Olfactory receptor 8H2. [Source:Uniprot/SWISSPROT;Acc:Q8N162] |
| - | 1.39 | -0.89 | 0.07 | XM_496261 | - |
| CLUL1 | 0.78 | -0.89 | -0.02 | NM_014410,NM_199167 | clusterin-like 1 (retinal) [Source:RefSeq_peptide;Acc:NP_954636] |
| AKAP4 | 0.35 | -0.89 | 0.68 | NM_139289 | A-kinase anchor protein 4 isoform 2 [Source:RefSeq_peptide;Acc:NP_647450] |
| LARS2 | 0.08 | -0.89 | 0.16 | NM_015340 | Probable leucyl-tRNA synthetase, mitochondrial precursor (EC 6.1.1.4) (Leucine--tRNA ligase) (LeuRS). [Source:Uniprot/SWISSPROT;Acc:Q15031] |
| - | -0.35 | -0.89 | 0.71 | XM_499238 | - |
| POU5F1 | -0.16 | -0.88 | 2.11 | NM_203289,NM_002701,XR_000175 | POU domain, class 5, transcription factor 1 (Octamer-binding transcription factor 3) (Oct-3) (Oct-4). [Source:Uniprot/SWISSPROT;Acc:Q01860] |
| - | 1.93 | -0.88 | 1.01 | XM_498952 | - |
| - | 0.11 | -0.88 | -0.21 | - | Similar to Ovarian cancer related tumor marker CA125 |
| NP_055977.3 | 0.17 | -0.88 | -0.45 | - | lipidosin [Source:RefSeq_peptide;Acc:NP_055977] |
| APOD | 0.36 | -0.88 | -0.08 | NM_001647 | Apolipoprotein D precursor (Apo-D) (ApoD). [Source:Uniprot/SWISSPROT;Acc:P05090] |
| Q9UHU9_HUMAN | -0.08 | -0.88 | 0.04 | - | - |
| - | 0.26 | -0.88 | -0.32 | NM_005709 | - |
| - | 0.07 | -0.88 | 0.52 | XM_497540 | - |
| - | 2.00 | -0.88 | -0.11 | - | Non-protein coding transcript |
| TTC7B_HUMAN | 0.15 | -0.88 | -0.43 | XM_085175 | Tetratricopeptide repeat protein 7B (Tetratricopeptide repeat protein 7 like-1). [Source:Uniprot/SWISSPROT;Acc:Q86TV6] |
| PTHR1 | 0.59 | -0.88 | -0.21 | - | Parathyroid hormone/parathyroid hormone-related peptide receptor precursor (PTH/PTHr receptor) (PTH/PTHrP type I receptor). [Source:Uniprot/SWISSPROT;Acc:Q03431] |
| HSPB9 | -0.16 | -0.88 | -0.03 | NM_033194 | Heat-shock protein beta-9 (HspB9). [Source:Uniprot/SWISSPROT;Acc:Q9BQS6] |
| - | 0.69 | -0.88 | -0.04 | - | SEC7-like domain containing protein |
| USHBP1 | 0.38 | -0.88 | 0.61 | NM_031941 | Usher syndrome 1C binding protein 1 [Source:RefSeq_peptide;Acc:NP_114147] |
| CYLC1 | 0.24 | -0.88 | 2.14 | XM_088636 | Cylicin-1 (Cylicin I) (Multiple-band polypeptide I) (Fragment). [Source:Uniprot/SWISSPROT;Acc:P35663] |
| SF3B4 | 0.85 | -0.88 | 0.36 | NM_005850 | Splicing factor 3B subunit 4 (Spliceosome associated protein 49) (SAP 49) (SF3b50) (Pre-mRNA splicing factor SF3b 49 kDa subunit). [Source:Uniprot/SWISSPROT;Acc:Q15427] |
| - | -0.31 | -0.87 | -0.37 | XM_498957 | - |
| HTR2B | 0.18 | -0.87 | 0.16 | NM_000867 | 5-hydroxytryptamine 2B receptor (5-HT-2B) (Serotonin receptor 2B). [Source:Uniprot/SWISSPROT;Acc:P41595] |
| OR2M1_HUMAN | -0.13 | -0.87 | 0.77 | NR_002141 | Olfactory receptor 2M1 (Olfactory receptor-like protein JCG10) (OST037) (Fragment). [Source:Uniprot/SWISSPROT;Acc:Q96R29] |
| OVCH1 | -0.13 | -0.87 | 0.15 | NM_183378 | ovochymase 1 [Source:RefSeq_peptide;Acc:NP_899234] |
| - | 0.61 | -0.87 | 0.77 | - | Hypothetical protein |
| - | -0.42 | -0.87 | -0.47 | - | - |
| STYX | -0.25 | -0.87 | -0.14 | NM_145251 | Serine/threonine/tyrosine interacting protein (Protein tyrosine phosphatase-like protein). [Source:Uniprot/SWISSPROT;Acc:Q8WUJ0] |
| NP_775754.1 | 0.75 | -0.87 | -0.08 | NM_173483 | cytochrome P450, family 2, subfamily E, polypeptide 2 homolog [Source:RefSeq_peptide;Acc:NP_775754] |
| - | -0.39 | -0.87 | -0.30 | - | Non-protein coding transcript |
| Q8IYS0_HUMAN | -0.26 | -0.87 | 0.20 | - | - |
| PACS1 | 0.14 | -0.87 | 0.44 | NM_018026 | Phosphofurin acidic cluster sorting protein 1 (PACS-1). [Source:Uniprot/SWISSPROT;Acc:Q6VY07] |
| - | -0.03 | -0.87 | 0.18 | NM_032440 | - |
| MEN1 | -0.28 | -0.87 | 1.68 | NM_130803 | Menin. [Source:Uniprot/SWISSPROT;Acc:O00255] |
| ZSWIM4 | 0.16 | -0.87 | -0.49 | XM_031342 | Zinc finger SWIM domain containing protein 4. [Source:Uniprot/SWISSPROT;Acc:Q9H7M6] |
| - | -0.22 | -0.87 | 0.15 | - | Hypothetical protein |
| OR8U1 | 0.67 | -0.87 | 0.17 | NM_001005204 | olfactory receptor, family 8, subfamily U, member 1 [Source:RefSeq_peptide;Acc:NP_001005204] |
| SPDY1 | 1.11 | -0.87 | 0.07 | NM_001008779,NM_182756 | speedy homolog 1 isoform 2 [Source:RefSeq_peptide;Acc:NP_877433] |
| GATA1 | -0.31 | -0.86 | -0.34 | NM_002049 | Erythroid transcription factor (GATA-1) (Eryf1) (GF-1) (NF-E1). [Source:Uniprot/SWISSPROT;Acc:P15976] |
| - | 0.16 | -0.86 | -0.45 | NM_145270 | - |
| - | 1.57 | -0.86 | -0.29 | XM_379355 | - |
| RAET1E | -0.21 | -0.86 | 0.29 | NM_139165 | NKG2D ligand 4 precursor (NKG2D ligand 4) (NKG2DL4) (N2DL-4) (Retinoic acid early transcript 1E) (Lymphocyte effector toxicity activation ligand) (RAE-1-like transcript 4) (RL-4) (UNQ1867/PRO4303). [Source:Uniprot/SWISSPROT;Acc:Q8TD07] |
| SV2B | -0.19 | -0.86 | -0.18 | NM_014848 | synaptic vesicle protein 2B homolog [Source:RefSeq_peptide;Acc:NP_055663] |
| - | 1.24 | -0.86 | -0.18 | XM_497489 | - |
| NP_620125.1 | 1.04 | -0.86 | -0.31 | NM_138770 | - |
| - | -0.04 | -0.86 | 0.19 | XM_497550 | - |
| - | 0.24 | -0.86 | -0.21 | XM_291200,XM_379839 | - |
| NP_061754.1 | -0.05 | -0.86 | -0.14 | NM_018931 | protocadherin beta 11 precursor [Source:RefSeq_peptide;Acc:NP_061754] |
| - | 0.38 | -0.86 | -0.07 | - | Hypothetical protein |
| XP_117224.6 | 1.68 | -0.86 | -0.46 | XM_117224 | PREDICTED: similar to RIKEN cDNA 0610009J22 [Source:RefSeq_peptide;Acc:XP_117224] |
| NP_848518.1 | 0.12 | -0.86 | -0.32 | NM_178431 | late cornified envelope 3A [Source:RefSeq_peptide;Acc:NP_848518] |
| - | 0.00 | -0.86 | 0.79 | - | Hypothetical protein |
| - | 0.42 | -0.86 | 0.70 | NM_020219 | - |
| - | 0.27 | -0.86 | -0.05 | XM_379146 | - |
| TPST2 | 0.18 | -0.85 | 0.02 | NM_003595,NM_001008566 | Protein-tyrosine sulfotransferase 2 (EC 2.8.2.20) (Tyrosylprotein sulfotransferase-2) (TPST-2). [Source:Uniprot/SWISSPROT;Acc:O60704] |
| CNTN5 | 0.81 | -0.85 | 0.35 | NM_014361,NM_175566 | Contactin 5 precursor (Neural recognition molecule NB-2) (hNB-2). [Source:Uniprot/SWISSPROT;Acc:O94779] |
| - | 0.61 | -0.85 | 0.21 | - | 16 kDa protein [Source:IPI;Acc:IPI00479070] |
| XP_499457.1 | -0.04 | -0.85 | 0.53 | XM_498225,XM_499457 | PREDICTED: similar to dJ133P16.1 (ADP-ribosylation factor 1) [Source:RefSeq_peptide;Acc:XP_498225] |
| - | -0.13 | -0.85 | -0.37 | XM_498884 | - |
| Q96R54_HUMAN | 0.10 | -0.85 | -0.49 | - | Olfactory receptor (Fragment). [Source:Uniprot/SPTREMBL;Acc:Q96R54] |
| XP_290351.3 | 0.98 | -0.85 | 0.32 | XM_290351 | PREDICTED: similar to Nedd-4-like E3 ubiquitin-protein ligase WWP1 (WW domain-containing protein 1) [Source:RefSeq_peptide;Acc:XP_290351] |
| NP_689809.2 | 0.59 | -0.85 | 0.42 | NM_152596 | - |
| GLRA2 | 0.29 | -0.85 | 0.05 | NM_002063 | Glycine receptor alpha-2 chain precursor. [Source:Uniprot/SWISSPROT;Acc:P23416] |
| C8orf8 | -0.31 | -0.85 | -0.29 | - | - |
| - | 0.29 | -0.85 | 1.60 | XM_208524 | - |
| SLC22A2 | 1.59 | -0.85 | 0.68 | NM_153191,NM_003058 | solute carrier family 22 member 2 isoform a [Source:RefSeq_peptide;Acc:NP_003049] |
| CD1E | 0.00 | -0.85 | 0.12 | NM_030893 | T-cell surface glycoprotein CD1e precursor (CD1e antigen) (R2G1). [Source:Uniprot/SWISSPROT;Acc:P15812] |
| - | 0.74 | -0.85 | -0.01 | XM_117117 | - |
| - | 0.34 | -0.85 | 0.11 | - | Non-protein coding transcript |
| TRPC7 | 0.78 | -0.85 | -0.16 | NM_020389 | Short transient receptor potential channel 7 (TrpC7) (TRP7 protein). [Source:Uniprot/SWISSPROT;Acc:Q9HCX4] |
| KCNMB2 | -0.22 | -0.85 | 1.24 | NM_005832,NM_181361 | Calcium-activated potassium channel beta subunit 2 (Calcium-activated potassium channel, subfamily M, beta subunit 2) (Maxi K channel beta subunit 2) (BK channel beta subunit 2) (Slo-beta 2) (K(VCA)beta 2) (Charybdotoxin receptor beta subunit 2) (BKbeta2) |
| - | 0.54 | -0.85 | -0.22 | XM_168073 | - |
| B3GNT3 | 1.66 | -0.85 | 1.26 | NM_014256 | Beta-1,3-galactosyltransferase 8 (EC 2.4.1.-) (Beta-1,3-GalTase 8) (Beta3Gal-T8) (b3Gal-T8) (UDP-galactose:beta-N-acetylglucosamine beta- 1,3-galactosyltransferase 8) (UDP-Gal:beta-GlcNAc beta-1,3- galactosyltransferase 8) (Beta-3-Gx-T8) (Core 1 extending |
| VA0D_HUMAN | -0.01 | -0.85 | -0.02 | NM_004691 | Vacuolar ATP synthase subunit d (EC 3.6.3.14) (V-ATPase d subunit) (Vacuolar proton pump d subunit) (V-ATPase AC39 subunit) (V-ATPase 40 kDa accessory protein) (P39) (32 kDa accessory protein). [Source:Uniprot/SWISSPROT;Acc:P61421] |
| - | -0.14 | -0.85 | -0.17 | NM_130844 | - |
| - | 0.35 | -0.85 | -0.04 | XM_378703 | - |
| NP_055775.2 | 0.12 | -0.84 | -0.47 | - | Arylsulfatase G [Source:RefSeq_peptide;Acc:NP_055775] |
| - | 0.55 | -0.84 | 0.42 | - | 18 kDa protein [Source:IPI;Acc:IPI00173720] |
| FMO3 | 0.22 | -0.84 | 0.08 | NM_001002294,NM_006894 | Dimethylaniline monooxygenase [N-oxide-forming] 3 (EC 1.14.13.8) (Hepatic flavin-containing monooxygenase 3) (FMO 3) (Dimethylaniline oxidase 3) (FMO form 2) (FMO II). [Source:Uniprot/SWISSPROT;Acc:P31513] |
| - | 0.30 | -0.84 | 0.23 | XM_377926,XM_379987 | - |
| Q9P143_HUMAN | 0.34 | -0.84 | 0.29 | - | - |
| KERA | 1.40 | -0.84 | 0.16 | NM_007035 | Keratocan precursor (KTN) (Keratan sulfate proteoglycan keratocan). [Source:Uniprot/SWISSPROT;Acc:O60938] |
| FOXP3 | -0.12 | -0.84 | -0.42 | NM_014009 | Forkhead box protein P3 (Zinc finger protein JM2) (Scurfin). [Source:Uniprot/SWISSPROT;Acc:Q9BZS1] |
| NP_775734.1 | 0.00 | -0.84 | -0.07 | NM_173463 | - |
| ZNF100 | -0.22 | -0.84 | 0.11 | NM_173531 | Zinc finger protein 100. [Source:Uniprot/SWISSPROT;Acc:Q8IYN0] |
| - | -0.06 | -0.84 | -0.43 | - | Human endogenous retrovirus HERV-K(II) DNA, complete sequence and flanking region |
| Q86UQ8_HUMAN | 0.91 | -0.84 | -0.33 | - | Transcription factor NF-E4. [Source:Uniprot/SPTREMBL;Acc:Q86UQ8] |
| PDP2_HUMAN | 0.00 | -0.84 | -0.13 | NM_020786 | [Pyruvate dehydrogenase [Lipoamide]]-phosphatase 2, mitochondrial precursor (EC 3.1.3.43) (PDP 2) (Pyruvate dehydrogenase phosphatase, catalytic subunit 2) (PDPC 2). [Source:Uniprot/SWISSPROT;Acc:Q9P2J9] |
| - | 0.37 | -0.84 | 1.24 | - | Hypothetical protein |
| BATF | -0.10 | -0.84 | 0.83 | NM_006399 | ATF-like basic leucine zipper transcriptional factor B-ATF (SF-HT- acivated gene-2) (SFA-2). [Source:Uniprot/SWISSPROT;Acc:Q16520] |
| STRN4 | -0.02 | -0.84 | 0.32 | NM_013403 | Striatin 4 (Zinedin). [Source:Uniprot/SWISSPROT;Acc:Q9NRL3] |
| LRP1 | -0.13 | -0.84 | -0.35 | NM_002332 | Low-density lipoprotein receptor-related protein 1 precursor (LRP) (Alpha-2-macroglobulin receptor) (A2MR) (Apolipoprotein E receptor) (APOER) (CD91). [Source:Uniprot/SWISSPROT;Acc:Q07954] |
| - | -0.42 | -0.84 | -0.15 | - | Conserved hypothetical protein |
| OR2Z1 | 0.61 | -0.84 | -0.21 | NM_001004699 | Olfactory receptor 2Z1. [Source:Uniprot/SWISSPROT;Acc:Q8NG97] |
| C6orf188 | 0.26 | -0.84 | 0.01 | NM_153711 | - |
| TS1R3_HUMAN | 0.35 | -0.84 | 0.42 | XM_371210 | Taste receptor type 1 member 3 precursor (Sweet taste receptor T1R3). [Source:Uniprot/SWISSPROT;Acc:Q7RTX0] |
| - | 0.11 | -0.84 | 0.45 | - | 25 kDa protein [Source:IPI;Acc:IPI00477485] |
| B3GALT5 | -0.32 | -0.84 | 0.76 | NM_033172,NM_006057,NM_033173,NM_033171,NM_033170 | Beta-1,3-galactosyltransferase 5 (EC 2.4.1.-) (Beta-1,3-GalTase 5) (Beta3Gal-T5) (b3Gal-T5) (UDP-galactose:beta-N-acetylglucosamine beta- 1,3-galactosyltransferase 5) (UDP-Gal:beta-GlcNAc beta-1,3- galactosyltransferase 5) (Beta-3-Gx-T5). [Source:Uniprot/ |
| PEPP2_HUMAN | -0.21 | -0.83 | 0.53 | NM_032498 | Paired-like homeobox protein PEPP-2 (Testis homeobox gene 1). [Source:Uniprot/SWISSPROT;Acc:Q9BQY4] |
| - | -0.09 | -0.83 | 0.11 | - | Hypothetical protein |
| FRMPD1 | -0.11 | -0.83 | 0.04 | NM_014907 | FERM and PDZ domain containing 1 [Source:RefSeq_peptide;Acc:NP_055722] |
| - | 0.12 | -0.83 | -0.09 | - | Non-protein coding transcript |
| SCHIP1 | 0.70 | -0.83 | 0.17 | NM_014575 | schwannomin interacting protein 1 [Source:RefSeq_peptide;Acc:NP_055390] |
| RANGAP1 | 0.30 | -0.83 | -0.19 | NM_002883 | Ran GTPase-activating protein 1. [Source:Uniprot/SWISSPROT;Acc:P46060] |
| SCMH1 | -0.24 | -0.83 | -0.06 | - | Polycomb protein SCMH1 (Sex comb on midleg homolog 1). [Source:Uniprot/SWISSPROT;Acc:Q96GD3] |
| XP_171078.5 | -0.40 | -0.83 | -0.06 | XM_171078 | PREDICTED: similar to Epigen protein [Source:RefSeq_peptide;Acc:XP_171078] |
| NXPH1 | -0.17 | -0.83 | -0.38 | NM_152745 | Neurexophilin-1 precursor. [Source:Uniprot/SWISSPROT;Acc:P58417] |
| XP_372838.2 | 0.01 | -0.83 | -0.39 | XM_372755 | PREDICTED: similar to RIKEN cDNA B230396O12 [Source:RefSeq_peptide;Acc:XP_372838] |
| - | -0.11 | -0.83 | -0.31 | - | Hypothetical protein |
| Q9UN39_HUMAN | 0.53 | -0.83 | 0.53 | - | Erythrocyte transmembrane protein. [Source:Uniprot/SPTREMBL;Acc:Q9UN39] |
| - | -0.13 | -0.83 | 0.14 | NM_021647 | - |
| ALOX5AP | 2.68 | -0.83 | -0.23 | NM_001629 | 5-lipoxygenase activating protein (FLAP) (MK-886-binding protein). [Source:Uniprot/SWISSPROT;Acc:P20292] |
| EOMES | 0.98 | -0.83 | 1.86 | NM_005442 | Eomesodermin homolog. [Source:Uniprot/SWISSPROT;Acc:O95936] |
| SBNO1 | 1.17 | -0.83 | 0.22 | NM_018183 | sno, strawberry notch homolog 1 [Source:RefSeq_peptide;Acc:NP_060653] |
| - | -0.18 | -0.83 | 0.16 | XM_498714 | - |
| - | 0.42 | -0.83 | 0.09 | - | Non-protein coding transcript |
| - | 0.42 | -0.83 | 0.35 | XM_498926 | - |
| C6 | -0.06 | -0.82 | -0.14 | NM_000065 | Complement component C6 precursor. [Source:Uniprot/SWISSPROT;Acc:P13671] |
| - | -0.29 | -0.82 | -0.05 | XM_499531 | - |
| NP_116106.2 | -0.28 | -0.82 | -0.37 | NM_032717 | - |
| XP_497833.1 | -0.11 | -0.82 | -0.24 | XM_497833 | PREDICTED: similar to SULT6B1 [Source:RefSeq_peptide;Acc:XP_497833] |
| - | 0.29 | -0.82 | -0.43 | - | 26 kDa protein [Source:IPI;Acc:IPI00414750] |
| - | -0.04 | -0.82 | -0.25 | XM_379180 | - |
| ALDH1L2 | 0.05 | -0.82 | 0.67 | XM_090294 | PREDICTED: similar to RIKEN cDNA D330038I09 [Source:RefSeq_peptide;Acc:XP_090294] |
| GIMAP7 | 2.02 | -0.82 | 0.58 | NM_153236 | GTPase, IMAP family member 7 (Immunity-associated nucleotide 7 protein). [Source:Uniprot/SWISSPROT;Acc:Q8NHV1] |
| - | 0.20 | -0.82 | -0.34 | - | 119 kDa protein [Source:IPI;Acc:IPI00477645] |
| TREM2_HUMAN | 0.62 | -0.82 | -0.10 | NM_018965 | Triggering receptor expressed on myeloid cells 2 precursor (Triggering receptor expressed on monocytes 2) (TREM-2). [Source:Uniprot/SWISSPROT;Acc:Q9NZC2] |
| XP_498465.1 | -0.07 | -0.82 | 0.05 | XM_499595,XM_498465 | PREDICTED: hypothetical protein XP_499595 [Source:RefSeq_peptide;Acc:XP_499595] |
| XP_372774.2 | 0.19 | -0.82 | -0.30 | XM_372774 | PREDICTED: hypothetical protein DJ159A19.3 [Source:RefSeq_peptide;Acc:XP_372774] |
| - | -0.44 | -0.82 | -0.21 | - | Human betaretrovirus isolate Es186-2 pol gene, partial cds |
| - | 2.56 | -0.82 | 0.45 | XM_290799,XM_496159 | - |
| - | 0.40 | -0.82 | 0.57 | XM_373749 | - |
| - | -0.04 | -0.82 | 0.25 | XM_086001 | - |
| ZNF599 | 0.73 | -0.82 | 0.52 | NM_001007247,NM_001007248 | zinc finger protein 599 isoform b [Source:RefSeq_peptide;Acc:NP_001007248] |
| PTPN22 | -0.50 | -0.82 | 0.31 | NM_015967,NM_012411 | Tyrosine-protein phosphatase, non-receptor type 22 (EC 3.1.3.48) (Hematopoietic cell protein-tyrosine phosphatase 70Z-PEP) (Lymphoid phosphatase) (LyP). [Source:Uniprot/SWISSPROT;Acc:Q9Y2R2] |
| - | 0.69 | -0.82 | 0.90 | - | Hypothetical protein |
| PITX2 | 0.35 | -0.81 | 0.10 | NM_153426,NM_000325,NM_153427 | Pituitary homeobox 2 (RIEG bicoid-related homeobox transcription factor) (Solurshin) (ALL1 responsive protein ARP1). [Source:Uniprot/SWISSPROT;Acc:Q99697] |
| XP_375359.1 | -0.23 | -0.81 | 1.11 | XM_375359 | PREDICTED: similar to Nedd4-binding brain specific protein BEAN [Source:RefSeq_peptide;Acc:XP_375359] |
| GRASP | 0.26 | -0.81 | -0.17 | NM_181711 | GRP1 (general receptor for phosphoinositides 1)-associated scaffold protein [Source:RefSeq_peptide;Acc:NP_859062] |
| - | 0.00 | -0.81 | 0.21 | - | Non-protein coding transcript |
| PICALM | 1.54 | -0.81 | 0.18 | NM_007166,NM_001008660 | Phosphatidylinositol-binding clathrin assembly protein (Clathrin assembly lymphoid myeloid leukemia protein). [Source:Uniprot/SWISSPROT;Acc:Q13492] |
| - | -0.41 | -0.81 | 0.44 | XM_379629 | - |
| - | -0.45 | -0.81 | 0.33 | XM_498363 | - |
| - | -0.18 | -0.81 | -0.48 | - | Hypothetical protein |
| CACNB4 | 0.44 | -0.81 | -0.34 | NM_001005747,NM_000726,NM_001005746 | Voltage-dependent L-type calcium channel beta-4 subunit (CAB4) (Calcium channel, voltage-dependent, beta 4 subunit). [Source:Uniprot/SWISSPROT;Acc:O00305] |
| - | -0.18 | -0.81 | 0.50 | - | - |
| - | 0.17 | -0.81 | 0.81 | XM_374190 | - |
| - | -0.41 | -0.81 | 0.85 | - | 17 kDa protein [Source:IPI;Acc:IPI00477015] |
| - | -0.41 | -0.81 | 0.44 | - | Homo sapiens microRNA miR-10b stem-loop |
| Q8N646_HUMAN | -0.18 | -0.81 | -0.36 | - | - |
| - | -0.08 | -0.81 | -0.23 | NM_001005365,NM_001002920 | - |
| - | -0.39 | -0.81 | -0.31 | NM_001010902 | - |
| - | 1.02 | -0.81 | -0.43 | - | Non-protein coding transcript |
| Q96PS2_HUMAN | 0.00 | -0.81 | 0.52 | - | FGF-2 activity-associated protein 3. [Source:Uniprot/SPTREMBL;Acc:Q96PS2] |
| XP_370654.2 | -0.23 | -0.80 | 0.64 | XM_370654 | PREDICTED: KIAA1726 protein [Source:RefSeq_peptide;Acc:XP_370654] |
| OR2TX_HUMAN | -0.01 | -0.80 | -0.14 | NM_001005495,NM_001001821 | Olfactory receptor 2T? (Fragment). [Source:Uniprot/SWISSPROT;Acc:Q8NH03] |
| Q8N1T0_HUMAN | 0.53 | -0.80 | -0.11 | - | - |
| Q7Z766_HUMAN | -0.45 | -0.80 | 0.01 | - | - |
| - | 0.11 | -0.80 | -0.44 | - | - |
| VgEcR | 0.01 | -0.80 | 0.62 | - | Synthetic VP16-glucocorticoid/ecdysone receptor VgEcR |
| XP_371760.2 | 0.01 | -0.80 | -0.21 | XM_371760 | PREDICTED: hypothetical protein LOC116068 [Source:RefSeq_peptide;Acc:XP_371760] |
| C18orf20 | 0.18 | -0.80 | -0.10 | NM_152728 | - |
| Q9Y348_HUMAN | 0.62 | -0.80 | -0.06 | - | - |
| Q8N872_HUMAN | 0.47 | -0.80 | 0.23 | - | - |
| KIAA1596 | 0.06 | -0.80 | -0.39 | - | - |
| NP_940928.1 | -0.11 | -0.80 | 0.61 | NM_198526 | - |
| - | 0.97 | -0.80 | 0.46 | NM_001003399 | - |
| KBTB4_HUMAN | 0.39 | -0.80 | 0.13 | NM_016506 | Kelch repeat and BTB domain containing protein 4 (BTB and kelch domain containing protein 4). [Source:Uniprot/SWISSPROT;Acc:Q9NVX7] |
| ZDHHC22 | -0.18 | -0.80 | 0.99 | NM_174976 | zinc finger, DHHC domain containing 22 [Source:RefSeq_peptide;Acc:NP_777636] |
| NP_872413.1 | -0.43 | -0.80 | 0.03 | NM_182607 | V-set and immunoglobulin domain containing 1 [Source:RefSeq_peptide;Acc:NP_872413] |
| - | 0.42 | -0.80 | 0.56 | XM_497249 | - |
| NP_543138.1 | 0.09 | -0.80 | -0.44 | NM_080862 | SPRY domain-containing SOCS box protein SSB-4 [Source:RefSeq_peptide;Acc:NP_543138] |
| - | 0.31 | -0.80 | -0.18 | XM_497675 | - |
| - | 0.31 | -0.79 | 2.26 | XM_373080 | - |
| MOS | 0.40 | -0.79 | 0.13 | NM_005372 | Proto-oncogene serine/threonine-protein kinase mos (EC 2.7.1.37) (c- mos) (Oocyte maturation factor mos). [Source:Uniprot/SWISSPROT;Acc:P00540] |
| Q8IY60_HUMAN | 0.03 | -0.79 | -0.29 | NM_133452 | RAVER1 [Source:RefSeq_peptide;Acc:NP_597709] |
| CUL4A | 0.27 | -0.79 | -0.04 | NM_003589,NM_001008895 | Cullin homolog 4A (CUL-4A). [Source:Uniprot/SWISSPROT;Acc:Q13619] |
| - | 0.05 | -0.79 | -0.45 | - | Human endogenous retrovirus HERV-K(I) DNA, complete sequence and flanking region |
| - | -0.38 | -0.79 | -0.42 | - | 7 kDa protein [Source:IPI;Acc:IPI00477033] |
| CC14A_HUMAN | 0.13 | -0.79 | -0.02 | NM_003672 | Dual specificity protein phosphatase CDC14A (EC 3.1.3.48) (EC 3.1.3.16) (CDC14 cell division cycle 14 homolog A). [Source:Uniprot/SWISSPROT;Acc:Q9UNH5] |
| NP_065952.1 | -0.21 | -0.79 | -0.41 | NM_020901 | CTD-binding SR-like protein rA9 [Source:RefSeq_peptide;Acc:NP_065952] |
| - | -0.08 | -0.79 | 0.67 | NM_001001691 | - |
| TAF4B | 0.01 | -0.79 | -0.35 | XM_290809 | Transcription initiation factor TFIID 105 kDa subunit (TAFII-105) (TAFII105) (Fragment). [Source:Uniprot/SWISSPROT;Acc:Q92750] |
| Q9UF98_HUMAN | 0.97 | -0.79 | -0.48 | NM_017564 | - |
| DEFB114 | 0.69 | -0.79 | -0.24 | - | Defensin beta 114 (Fragment). [Source:Uniprot/SPTREMBL;Acc:Q8NES9] |
| - | -0.41 | -0.79 | -0.39 | XM_496663 | - |
| - | -0.17 | -0.79 | 1.20 | - | Hypothetical protein |
| WFDC5 | -0.23 | -0.79 | -0.09 | NM_145652 | WAP four-disulfide core domain protein 5 precursor (Putative protease inhibitor WAP1). [Source:Uniprot/SWISSPROT;Acc:Q8TCV5] |
| - | -0.26 | -0.79 | 3.31 | XM_498483 | - |
| AHSG | -0.49 | -0.78 | -0.47 | NM_001622 | Alpha-2-HS-glycoprotein precursor (Fetuin-A) (Alpha-2-Z-globulin) (Ba- alpha-2-glycoprotein) (PRO2743). [Source:Uniprot/SWISSPROT;Acc:P02765] |
| - | 0.67 | -0.78 | 0.28 | - | Hypothetical protein |
| CPA1 | -0.06 | -0.78 | -0.22 | NM_001868 | Carboxypeptidase A1 precursor (EC 3.4.17.1). [Source:Uniprot/SWISSPROT;Acc:P15085] |
| DHX30 | 0.87 | -0.78 | -0.22 | NM_138615,NM_014966 | DEAH (Asp-Glu-Ala-His) box polypeptide 30 isoform 2 [Source:RefSeq_peptide;Acc:NP_055781] |
| NP_001006940.2 | 0.96 | -0.78 | -0.37 | NM_001006939 | leucine rich repeat containing 18 [Source:RefSeq_peptide;Acc:NP_001006940] |
| NP_001004432.1 | 1.41 | -0.78 | -0.20 | NM_001004432 | leucine rich repeat neuronal 6D [Source:RefSeq_peptide;Acc:NP_001004432] |
| - | -0.29 | -0.78 | -0.32 | XM_496659 | - |
| - | -0.27 | -0.78 | -0.49 | - | HMG-I and HMG-Y DNA-binding domain (A+T-hook) containing protein |
| - | -0.40 | -0.78 | -0.32 | - | HRDPXB6 Human type D retrovirus gene, clone:pXB-6 |
| AMPD2 | 0.52 | -0.78 | 0.26 | NM_004037,NM_203404,NM_139156 | AMP deaminase 2 (EC 3.5.4.6) (AMP deaminase isoform L). [Source:Uniprot/SWISSPROT;Acc:Q01433] |
| - | 0.00 | -0.78 | -0.34 | XM_378507 | - |
| OR5W2 | 0.18 | -0.78 | 1.00 | NM_001001960 | olfactory receptor, family 5, subfamily W, member 2 [Source:RefSeq_peptide;Acc:NP_001001960] |
| - | 0.44 | -0.78 | -0.36 | XM_376795 | - |
| FIGN | 0.33 | -0.78 | 0.65 | NM_018086 | fidgetin [Source:RefSeq_peptide;Acc:NP_060556] |
| HUTU_HUMAN | 0.10 | -0.78 | 0.15 | NM_144639 | Probable urocanate hydratase (EC 4.2.1.49) (Urocanase) (Imidazolonepropionate hydrolase). [Source:Uniprot/SWISSPROT;Acc:Q96N76] |
| KRTAP17-1 | 0.13 | -0.78 | 0.06 | NM_031964 | keratin associated protein 17-1 [Source:RefSeq_peptide;Acc:NP_114170] |
| Q8N2X2_HUMAN | 0.34 | -0.78 | -0.34 | NM_001009607 | similar to RIKEN cDNA 4933437K13 [Source:RefSeq_peptide;Acc:NP_001009607] |
| Q96S13_HUMAN | 0.32 | -0.78 | 0.71 | - | - |
| GAB2 | -0.13 | -0.78 | -0.05 | NM_080491,NM_012296 | GRB2-associated binding protein 2 (GRB2-associated binder-2) (pp100). [Source:Uniprot/SWISSPROT;Acc:Q9UQC2] |
| S100A8 | 0.70 | -0.78 | 0.34 | NM_002964 | Calgranulin A (Migration inhibitory factor-related protein 8) (MRP-8) (Cystic fibrosis antigen) (CFAG) (P8) (Leukocyte L1 complex light chain) (S100 calcium-binding protein A8) (Calprotectin L1L subunit) (Urinary stone protein band A). [Source:Uniprot/SWI |
| XRRA1 | -0.02 | -0.78 | 0.80 | XM_374912 | PREDICTED: X-ray radiation resistance associated 1 [Source:RefSeq_peptide;Acc:XP_374912] |
| GAS2L2 | -0.43 | -0.78 | -0.14 | - | growth arrest-specific 2 like 2 [Source:RefSeq_peptide;Acc:NP_644814] |
| RAB17 | -0.12 | -0.78 | 0.42 | NM_022449 | Ras-related protein Rab-17. [Source:Uniprot/SWISSPROT;Acc:Q9H0T7] |
| NP_001005160.1 | 0.21 | -0.78 | 0.22 | NM_001005160 | olfactory receptor, family 52, subfamily A, member 5 [Source:RefSeq_peptide;Acc:NP_001005160] |
| XP_376899.1 | -0.29 | -0.78 | 0.92 | XM_376899 | PREDICTED: similar to gene [Source:RefSeq_peptide;Acc:XP_376899] |
| - | -0.25 | -0.78 | -0.48 | NM_207479 | - |
| - | -0.01 | -0.78 | -0.15 | NM_153379 | - |
| DOC2A | 0.12 | -0.77 | -0.27 | NM_003586 | Double C2-like domain containing protein alpha (Doc2 alpha) (Doc2). [Source:Uniprot/SWISSPROT;Acc:Q14183] |
| GPR26 | 1.43 | -0.77 | 0.07 | NM_153442 | Probable G-protein coupled receptor 26. [Source:Uniprot/SWISSPROT;Acc:Q8NDV2] |
| RNASE6 | -0.05 | -0.77 | -0.19 | NM_005615 | Ribonuclease K6 precursor (EC 3.1.27.-) (RNase K6). [Source:Uniprot/SWISSPROT;Acc:Q93091] |
| NP_996553.1 | 0.28 | -0.77 | -0.15 | NM_206818,NM_206817 | osteoclast-associated receptor isoform 4 [Source:RefSeq_peptide;Acc:NP_573399] |
| GUCA2B | -0.44 | -0.77 | -0.05 | NM_007102 | Uroguanylin precursor (UGN) (Guanylate cyclase activator 2B) (Guanylate cyclase C activating peptide II) (GCAP-II). [Source:Uniprot/SWISSPROT;Acc:Q16661] |
| Q9H4I0_HUMAN | 1.65 | -0.77 | 0.44 | - | OTTHUMP00000029969 (Fragment). [Source:Uniprot/SPTREMBL;Acc:Q9H4I0] |
| NALP14 | 0.71 | -0.77 | 0.16 | NM_176822 | NACHT-, LRR- and PYD-containing protein 14 (Nucleotide-binding oligomerization domain protein 5). [Source:Uniprot/SWISSPROT;Acc:Q86W24] |
| ZNF43 | 0.36 | -0.77 | 0.08 | NM_003423 | Zinc finger protein 43 (Zinc protein HTF6) (Zinc finger protein KOX27). [Source:Uniprot/SWISSPROT;Acc:P17038] |
| ACADL | -0.10 | -0.77 | -0.30 | NM_001608 | Acyl-CoA dehydrogenase, long-chain specific, mitochondrial precursor (EC 1.3.99.13) (LCAD). [Source:Uniprot/SWISSPROT;Acc:P28330] |
| NP_116238.2 | 0.00 | -0.77 | -0.26 | NM_032849 | - |
| Q8N5K5_HUMAN | -0.13 | -0.77 | 0.20 | - | MGC40042 protein. [Source:Uniprot/SPTREMBL;Acc:Q8N5K5] |
| - | -0.31 | -0.77 | 1.59 | - | 24 kDa protein [Source:IPI;Acc:IPI00413638] |
| - | -0.34 | -0.77 | -0.27 | NM_020875 | - |
| PMCHL1 | 0.05 | -0.77 | 2.52 | NM_031887 | Pro-MCH variant (Pro-melanin-concentrating hormone-like 1 protein). [Source:Uniprot/SWISSPROT;Acc:Q16048] |
| C13orf17 | 0.00 | -0.77 | 0.13 | NM_018185 | - |
| - | 0.89 | -0.77 | 0.18 | XM_371164 | - |
| POM121L2 | -0.06 | -0.77 | -0.26 | - | POM121 membrane glycoprotein-like 2 (Rat). [Source:Uniprot/SPTREMBL;Acc:Q96KW2] |
| - | -0.25 | -0.77 | 2.15 | XM_374752 | - |
| ITIH4 | -0.26 | -0.76 | -0.35 | NM_002218 | Inter-alpha-trypsin inhibitor heavy chain H4 precursor (ITI heavy chain H4) (Inter-alpha-inhibitor heavy chain 4) (Inter-alpha-trypsin inhibitor family heavy chain-related protein) (IHRP) (Plasma kallikrein sensitive glycoprotein 120) (PK-120) (GP120) (PR |
| NP_001001414.1 | 1.87 | -0.76 | -0.38 | NM_001001414 | similar to F-box only protein 2 [Source:RefSeq_peptide;Acc:NP_001001414] |
| ACR | 0.17 | -0.76 | 0.84 | XM_208270,NM_001097 | Acrosin precursor (EC 3.4.21.10). [Source:Uniprot/SWISSPROT;Acc:P10323] |
| TGFBRAP1 | -0.30 | -0.76 | -0.05 | NM_004257 | TGF beta receptor associated protein -1 [Source:RefSeq_peptide;Acc:NP_004248] |
| Q8NAG9_HUMAN | 0.88 | -0.76 | 0.43 | - | - |
| - | 0.51 | -0.76 | 0.07 | XM_209076 | - |
| LAMA2 | -0.41 | -0.76 | 0.39 | NM_000426 | Laminin alpha-2 chain precursor (Laminin M chain) (Merosin heavy chain). [Source:Uniprot/SWISSPROT;Acc:P24043] |
| NP_997215.1 | -0.28 | -0.76 | -0.34 | NM_207332 | - |
| XP_059672.3 | 0.58 | -0.76 | 0.15 | XM_059672 | PREDICTED: hypothetical protein XP_059672 [Source:RefSeq_peptide;Acc:XP_059672] |
| - | 1.04 | -0.76 | -0.14 | XM_378941 | - |
| - | 0.30 | -0.76 | -0.45 | - | 33 kDa protein [Source:IPI;Acc:IPI00402438] |
| - | 0.11 | -0.76 | -0.31 | XM_496237 | - |
| - | -0.20 | -0.76 | -0.41 | XM_498846 | - |
| - | 0.41 | -0.76 | 0.54 | - | Hypothetical protein |
| - | -0.05 | -0.76 | 1.06 | - | Similar to Survival motor neuron protein |
| NP_683695.1 | 0.80 | -0.76 | -0.16 | NM_148897 | orphan short-chain dehydrogenase / reductase [Source:RefSeq_peptide;Acc:NP_683695] |
| AP1S3 | 0.21 | -0.76 | 0.49 | - | Adapter-related protein complex 1 sigma 1C subunit (Sigma-adaptin 1C) (Adaptor protein complex AP-1 sigma-1C subunit) (Golgi adaptor HA1/AP1 adaptin sigma-1C subunit) (Clathrin assembly protein complex 1 sigma- 1C small chain) (Sigma 1C subunit of AP-1 cl |
| NP_997723.1 | 0.39 | -0.76 | 0.03 | NM_212558 | similar to RIKEN A930001M12 [Source:RefSeq_peptide;Acc:NP_997723] |
| - | 1.02 | -0.76 | 0.50 | NM_144781 | - |
| - | 0.35 | -0.76 | -0.37 | - | Hypothetical protein |
| PTPN3 | 0.56 | -0.76 | -0.07 | NM_002829 | Tyrosine-protein phosphatase, non-receptor type 3 (EC 3.1.3.48) (Protein-tyrosine phosphatase H1) (PTP-H1). [Source:Uniprot/SWISSPROT;Acc:P26045] |
| KRTAP4-3 | 0.77 | -0.76 | 0.92 | XM_497545 | Keratin-associated protein 4-3 (Keratin-associated protein 4.3) (Ultrahigh sulfur keratin-associated protein 4.3) (Fragment). [Source:Uniprot/SWISSPROT;Acc:Q9BYR4] |
| - | 0.58 | -0.76 | -0.49 | - | Hypothetical protein |
| ADAMTS6 | 0.32 | -0.76 | 0.18 | NM_014273 | ADAMTS-6 precursor (EC 3.4.24.-) (A disintegrin and metalloproteinase with thrombospondin motifs 6) (ADAM-TS 6) (ADAM-TS6). [Source:Uniprot/SWISSPROT;Acc:Q9UKP5] |
| - | -0.08 | -0.76 | -0.49 | XM_379112 | - |
| - | 0.33 | -0.76 | -0.16 | - | Non-protein coding transcript |
| - | -0.04 | -0.75 | -0.13 | XM_373726 | - |
| - | -0.19 | -0.75 | 1.69 | XM_498388,XM_498390 | - |
| OR51L1 | -0.28 | -0.75 | 0.97 | NM_001004755 | Olfactory receptor 51L1. [Source:Uniprot/SWISSPROT;Acc:Q8NGJ5] |
| SLC16A5 | -0.06 | -0.75 | 0.39 | NM_004695 | Monocarboxylate transporter 6 (MCT 6) (MCT 5). [Source:Uniprot/SWISSPROT;Acc:O15375] |
| - | 0.93 | -0.75 | 0.24 | XM_374270 | - |
| SOSTDC1 | 0.54 | -0.75 | 0.75 | NM_015464 | cystine knot-containing secreted protein [Source:RefSeq_peptide;Acc:NP_056279] |
| - | 0.18 | -0.75 | 0.17 | XM_088797 | - |
| NP_001004492.1 | -0.45 | -0.75 | 0.55 | NM_001004492 | olfactory receptor, family 2, subfamily B, member 11 [Source:RefSeq_peptide;Acc:NP_001004492] |
| GPR147 | -0.41 | -0.75 | 0.46 | NM_022146 | Neuropeptide FF receptor 1 (G-protein coupled receptor 147) (RFamide- related peptide receptor OT7T022). [Source:Uniprot/SWISSPROT;Acc:Q9GZQ6] |
| - | 0.45 | -0.75 | 0.75 | XM_498723 | - |
| - | -0.04 | -0.75 | 0.69 | - | Non-protein coding transcript |
| Q96A81_HUMAN | 0.36 | -0.75 | 1.88 | NM_144682 | - |
| NR_001278.1 | 0.37 | -0.75 | 0.29 | NR_001278 | cytochrome P450, family 2, subfamily B, polypeptide 7 pseudogene 1 (CYP2B7P1) on chromosome 19 [Source:RefSeq_dna;Acc:NR_001278] |
| NP_937828.1 | 0.20 | -0.75 | -0.33 | NM_198185 | ovochymase 2 [Source:RefSeq_peptide;Acc:NP_937828] |
| - | 1.04 | -0.75 | 0.33 | NR_001553,XM_293449,XM_497197,XM_497193,XM_497191,XM_497198,XM_497196 | 30 kDa protein [Source:IPI;Acc:IPI00414084] |
| - | -0.06 | -0.75 | 0.01 | XM_372616,XM_497491 | 26 kDa protein [Source:IPI;Acc:IPI00472137] |
| - | 0.71 | -0.75 | -0.33 | - | Hypothetical protein |
| Q96MY2_HUMAN | 0.55 | -0.75 | 0.10 | - | - |
| C20orf70 | 0.53 | -0.75 | -0.49 | NM_080574 | Short palate, lung and nasal epithelium carcinoma associated protein 2 precursor (Parotid secretory protein) (PSP) (UNQ510/PRO1025). [Source:Uniprot/SWISSPROT;Acc:Q96DR5] |
| ERR3_HUMAN | -0.43 | -0.75 | -0.19 | NM_206595,NM_001438,NM_206594 | Estrogen-related receptor gamma (Estrogen receptor related protein 3) (ERR gamma-2). [Source:Uniprot/SWISSPROT;Acc:P62508] |
| - | -0.19 | -0.75 | 0.11 | NM_001010976 | - |
| CPA4 | 1.71 | -0.75 | -0.47 | NM_016352 | Carboxypeptidase A4 precursor (EC 3.4.17.-) (Carboxypeptidase A3). [Source:Uniprot/SWISSPROT;Acc:Q9UI42] |
| STXBP5L | 0.21 | -0.75 | -0.06 | XM_045911 | PREDICTED: syntaxin binding protein 5-like [Source:RefSeq_peptide;Acc:XP_045911] |
| TBX19 | 0.16 | -0.75 | 1.06 | NM_005149 | T-box transcription factor TBX19 (T-box protein 19). [Source:Uniprot/SWISSPROT;Acc:O60806] |
| NP_079217.1 | 0.55 | -0.74 | 0.00 | NM_024941 | - |
| LAPTM5 | -0.19 | -0.74 | -0.43 | NM_006762 | Lysosomal-associated multitransmembrane protein (Retinoic acid- inducible E3 protein) (HA1520). [Source:Uniprot/SWISSPROT;Acc:Q13571] |
| - | 0.04 | -0.74 | 0.05 | XM_211557 | - |
| WNT7A | -0.36 | -0.74 | 0.36 | NM_004625 | Wnt-7a protein precursor. [Source:Uniprot/SWISSPROT;Acc:O00755] |
| NP_001003892.1 | 0.45 | -0.74 | 0.55 | NM_001003892,XM_497237 | dual specificity phosphatase and pro isomerase domain containing 1 [Source:RefSeq_peptide;Acc:NP_001003892] |
| OR52I1 | 0.47 | -0.74 | 0.00 | NM_001005170 | Olfactory receptor 52I2. [Source:Uniprot/SWISSPROT;Acc:Q8NH67] |
| - | 0.18 | -0.74 | -0.46 | NM_152613 | - |
| Q96FU4_HUMAN | -0.20 | -0.74 | -0.18 | XM_499057,XM_499505 | - |
| Q96MB3_HUMAN | 0.36 | -0.74 | -0.45 | - | - |
| GUCA1C | 0.74 | -0.74 | -0.08 | NM_005459 | Guanylyl cyclase activating protein 3 (GCAP 3) (Guanylate cyclase activator 1C). [Source:Uniprot/SWISSPROT;Acc:O95843] |
| PIK3CG | 0.00 | -0.74 | 0.65 | NM_002649 | Phosphatidylinositol-4,5-bisphosphate 3-kinase catalytic subunit, gamma isoform (EC 2.7.1.153) (PI3-kinase p110 subunit gamma) (PtdIns- 3-kinase p110) (PI3K) (PI3Kgamma). [Source:Uniprot/SWISSPROT;Acc:P48736] |
| - | 0.05 | -0.74 | -0.19 | - | 71 kDa protein [Source:IPI;Acc:IPI00412751] |
| - | 0.52 | -0.74 | 0.10 | - | Hypothetical protein |
| ID3 | 0.49 | -0.74 | 0.50 | NM_002167 | DNA-binding protein inhibitor ID-3 (ID-like protein inhibitor HLH 1R21) (Helix-loop-helix protein HEIR-1). [Source:Uniprot/SWISSPROT;Acc:Q02535] |
| O95724_HUMAN | 0.14 | -0.74 | 0.16 | - | Reverse transcriptase (Fragment). [Source:Uniprot/SPTREMBL;Acc:O95724] |
| EMX1 | 0.44 | -0.74 | 0.23 | NM_004097 | Homeobox protein EMX1 (Empty spiracles homolog 1) (Empty spiracles- like protein 1). [Source:Uniprot/SWISSPROT;Acc:Q04741] |
| - | 0.47 | -0.74 | -0.39 | - | Homo sapiens microRNA miR-340 stem-loop |
| GPR55 | 0.10 | -0.74 | -0.13 | NM_005683 | G protein-coupled receptor 55 [Source:RefSeq_peptide;Acc:NP_005674] |
| OR13C8 | -0.19 | -0.74 | 1.34 | NM_001004483 | Olfactory receptor 13C8. [Source:Uniprot/SWISSPROT;Acc:Q8NGS7] |
| NP_659802.1 | 1.55 | -0.74 | 0.07 | NM_145111 | - |
| - | -0.04 | -0.74 | -0.21 | NM_203381 | - |
| Q6ZNC3_HUMAN | 0.12 | -0.74 | -0.03 | - | - |
| PLEC1 | -0.25 | -0.74 | -0.50 | NM_201383,NM_201382,NM_201380,NM_201378,NM_201379,NM_201384,NM_201381,NM_000445 | Plectin 1 (PLTN) (PCN) (Hemidesmosomal protein 1) (HD1). [Source:Uniprot/SWISSPROT;Acc:Q15149] |
| TBC13_HUMAN | 0.00 | -0.74 | -0.40 | NM_018201 | TBC1 domain family member 13. [Source:Uniprot/SWISSPROT;Acc:Q9NVG8] |
| - | -0.02 | -0.74 | -0.19 | - | Hypothetical protein |
| CCDC9 | -0.17 | -0.73 | -0.21 | NM_015603 | Coiled-coil domain containing protein 9. [Source:Uniprot/SWISSPROT;Acc:Q9Y3X0] |
| - | -0.19 | -0.73 | -0.42 | - | Non-protein coding transcript |
| - | -0.28 | -0.73 | -0.15 | - | Conserved hypothetical protein |
| ARMC2 | 0.00 | -0.73 | -0.24 | NM_032131 | armadillo repeat containing 2 [Source:RefSeq_peptide;Acc:NP_115507] |
| CDH1 | 0.37 | -0.73 | 0.20 | NM_004360 | Epithelial-cadherin precursor (E-cadherin) (Uvomorulin) (Cadherin-1) (CAM 120/80). [Source:Uniprot/SWISSPROT;Acc:P12830] |
| NP_689985.1 | 0.00 | -0.73 | 0.16 | NM_152772 | - |
| - | 0.08 | -0.73 | -0.39 | NM_032874 | - |
| - | -0.24 | -0.73 | -0.47 | XM_378786 | - |
| - | -0.01 | -0.73 | 0.48 | - | Hypothetical protein |
| - | -0.02 | -0.73 | -0.32 | - | Non-protein coding transcript |
| carb-6 | -0.13 | -0.73 | 0.06 | - | Vibrio cholerae non O1, non O139 plasmid class A beta-lactamase |
| Q8N6D0_HUMAN | 0.70 | -0.73 | 0.29 | - | NK inhibitory receptor precursor [Source:RefSeq_peptide;Acc:NP_620587] |
| MMP1 | -0.20 | -0.73 | 0.27 | NM_002421 | Interstitial collagenase precursor (EC 3.4.24.7) (Matrix metalloproteinase-1) (MMP-1) (Fibroblast collagenase). [Source:Uniprot/SWISSPROT;Acc:P03956] |
| IL10RA | -0.31 | -0.73 | -0.03 | NM_001558 | Interleukin-10 receptor alpha chain precursor (IL-10R-A) (IL-10R1). [Source:Uniprot/SWISSPROT;Acc:Q13651] |
| - | 0.07 | -0.73 | 1.48 | - | Similar to T-cell surface glycoprotein CD3 epsilon chain precursor (T-cell surface antigen T3/LEU-4 epsilon chain) |
| SEPT8 | -0.21 | -0.73 | -0.17 | - | Septin 8. [Source:Uniprot/SWISSPROT;Acc:Q92599] |
| Q8TBP8_HUMAN | 0.74 | -0.73 | 0.20 | NM_012190,NM_144776 | 10-formyltetrahydrofolate dehydrogenase (EC 1.5.1.6) (10-FTHFDH) (Aldehyde dehydrogenase 1 family member L1). [Source:Uniprot/SWISSPROT;Acc:O75891] |
| GTF2A1 | 1.81 | -0.73 | -0.15 | NM_015859,NM_201595 | Transcription initiation factor IIA alpha and beta chains (TFIIA p35 and p19 subunits) (TFIIA-42) (TFIIAL). [Source:Uniprot/SWISSPROT;Acc:P52655] |
| AURKC | 1.16 | -0.73 | 0.02 | NM_003160 | Serine/threonine-protein kinase 13 (EC 2.7.1.37) (Aurora/Ipl1/Eg2 protein 2) (Aurora/Ipl1-related kinase 3) (Aurora-C). [Source:Uniprot/SWISSPROT;Acc:Q9UQB9] |
| Q96AT3_HUMAN | 0.89 | -0.73 | 1.90 | - | LRP11 protein (Fragment). [Source:Uniprot/SPTREMBL;Acc:Q96AT3] |
| - | 0.53 | -0.73 | 0.58 | XM_497733 | - |
| C6orf51 | 0.72 | -0.73 | -0.20 | NM_138408 | (NPD020) (CDA020). [Source:Uniprot/SWISSPROT;Acc:Q969F1] |
| - | -0.35 | -0.73 | -0.20 | XM_379118 | - |
| Q13808_HUMAN | -0.36 | -0.73 | -0.30 | - | Antisense BCMA peptide. [Source:Uniprot/SPTREMBL;Acc:Q13808] |
| - | -0.25 | -0.73 | 0.15 | - | 18 kDa protein [Source:IPI;Acc:IPI00412260] |
| - | -0.49 | -0.73 | -0.37 | XM_496051 | - |
| HTRA3 | 0.54 | -0.73 | 0.66 | NM_053044 | Probable serine protease HTRA3 precursor (EC 3.4.21.-) (High- temperature requirement factor A3) (Pregnancy-related serine protease). [Source:Uniprot/SWISSPROT;Acc:P83110] |
| Q8NGM0_HUMAN | -0.23 | -0.73 | 0.55 | - | Seven transmembrane helix receptor. [Source:Uniprot/SPTREMBL;Acc:Q8NGM0] |
| XP_208927.4 | -0.39 | -0.73 | 0.39 | XM_208927 | - |
| - | 0.00 | -0.73 | -0.45 | NM_001001676 | - |
| - | 0.16 | -0.73 | 0.14 | XM_497412 | - |
| - | -0.23 | -0.73 | -0.11 | - | Hypothetical protein |
| KRT1B | 0.27 | -0.72 | -0.03 | NM_175078 | keratin 1B [Source:RefSeq_peptide;Acc:NP_778253] |
| NP_620169.1 | 1.24 | -0.72 | -0.27 | NM_138814 | patatin-like phospholipase domain containing 5 [Source:RefSeq_peptide;Acc:NP_620169] |
| - | -0.35 | -0.72 | -0.04 | XM_497601 | - |
| XP_496631.1 | 1.05 | -0.72 | 0.42 | XM_496631 | PREDICTED: hypothetical protein FLJ35107 [Source:RefSeq_peptide;Acc:XP_496631] |
| Q9UHU1_HUMAN | -0.11 | -0.72 | 0.31 | - | - |
| - | 0.45 | -0.72 | 0.02 | XM_037493 | 26 kDa protein [Source:IPI;Acc:IPI00413466] |
| - | 1.31 | -0.72 | -0.22 | XM_498949 | - |
| OPHN1 | -0.23 | -0.72 | 1.14 | NM_002547 | Oligophrenin 1. [Source:Uniprot/SWISSPROT;Acc:O60890] |
| - | -0.12 | -0.72 | 0.93 | XM_498825 | - |
| - | -0.07 | -0.72 | -0.25 | XM_499394,XM_171165,NM_002735 | protein kinase, cAMP-dependent, regulatory, type I, beta (PRKAR1B), mRNA [Source:RefSeq_dna;Acc:NM_002735] |
| - | -0.08 | -0.72 | 0.28 | XM_497271 | - |
| HOXD13 | -0.45 | -0.72 | 0.58 | NM_000523 | Homeobox protein Hox-D13 (Hox-4I). [Source:Uniprot/SWISSPROT;Acc:P35453] |
| IL27RA | -0.12 | -0.72 | 0.97 | NM_004843 | class I cytokine receptor [Source:RefSeq_peptide;Acc:NP_004834] |
| - | -0.11 | -0.72 | -0.22 | NM_207368 | - |
| NINJ2 | 0.43 | -0.72 | 0.35 | NM_016533 | Ninjurin 2 (Nerve injury-induced protein 2). [Source:Uniprot/SWISSPROT;Acc:Q9NZG7] |
| - | -0.08 | -0.72 | -0.08 | XM_089307 | - |
| ANKRD7 | -0.02 | -0.72 | -0.21 | NM_019644 | Ankyrin repeat domain protein 7 (Testis-specific protein TSA806). [Source:Uniprot/SWISSPROT;Acc:Q92527] |
| - | -0.15 | -0.72 | 0.05 | - | 53 kDa protein [Source:IPI;Acc:IPI00260402] |
| - | 0.03 | -0.72 | -0.21 | - | Hypothetical protein |
| ZP4 | 0.52 | -0.72 | -0.36 | NM_021186 | Zona pellucida sperm-binding protein 4 precursor (Zona pellucida sperm-binding protein B). [Source:Uniprot/SWISSPROT;Acc:Q12836] |
| - | 0.13 | -0.72 | -0.45 | - | 24 kDa protein [Source:IPI;Acc:IPI00477000] |
| FGF22 | 0.99 | -0.72 | -0.19 | NM_020637 | Fibroblast growth factor-22 precursor (FGF-22) (UNQ2500/PRO5800). [Source:Uniprot/SWISSPROT;Acc:Q9HCT0] |
| - | 0.20 | -0.72 | -0.08 | - | Hypothetical protein |
| FOXP4 | 0.53 | -0.72 | -0.31 | NM_138457 | Forkhead box protein P4 (Fork head-related protein like A). [Source:Uniprot/SWISSPROT;Acc:Q8IVH2] |
| SPAG11 | -0.14 | -0.72 | 0.31 | NM_058203 | Sperm-associated antigen 11 precursor (EP2 protein) (Sperm antigen HE2). [Source:Uniprot/SWISSPROT;Acc:Q08648] |
| OR51E2 | 0.42 | -0.72 | 0.09 | NM_030774 | Olfactory receptor 51E2 (Prostate specific G-protein coupled receptor) (HPRAJ). [Source:Uniprot/SWISSPROT;Acc:Q9H255] |
| O10J6_HUMAN | 0.89 | -0.72 | -0.37 | - | Olfactory receptor 10J6. [Source:Uniprot/SWISSPROT;Acc:Q8NGY7] |
| TIRAP | -0.14 | -0.72 | -0.11 | NM_148910,NM_052887 | Toll-interleukin 1 receptor domain-containing adapter protein (TIR domain-containing adapter protein) (MyD88 adapter-like protein) (Adaptor protein Wyatt). [Source:Uniprot/SWISSPROT;Acc:P58753] |
| - | 0.66 | -0.72 | 0.49 | - | Hypothetical protein |
| - | 0.64 | -0.72 | 1.47 | - | Hypothetical protein |
| DSCAML1 | 0.32 | -0.72 | -0.35 | NM_020693 | Down syndrome cell adhesion molecule like 1 [Source:RefSeq_peptide;Acc:NP_065744] |
| - | -0.10 | -0.72 | -0.18 | - | Hypothetical protein |
| - | 0.12 | -0.72 | -0.19 | NM_007283,NM_001003794 | - |
| AMPD1 | 0.06 | -0.72 | 0.01 | NM_000036 | AMP deaminase 1 (EC 3.5.4.6) (Myoadenylate deaminase) (AMP deaminase isoform M). [Source:Uniprot/SWISSPROT;Acc:P23109] |
| XP_376186.2 | -0.44 | -0.72 | -0.10 | XM_376186 | PREDICTED: similar to hypothetical protein [Source:RefSeq_peptide;Acc:XP_376186] |
| KRT25C | 0.62 | -0.72 | -0.26 | NM_181537 | keratin 25C [Source:RefSeq_peptide;Acc:NP_853515] |
| - | 0.38 | -0.72 | 0.12 | - | Hypothetical protein |
| Q8WY77_HUMAN | 0.25 | -0.71 | -0.36 | NM_024105 | Dolichyl-P-Man:Man(7)GlcNAc(2)-PP-dolichyl-alpha-1,6- mannosyltransferase (EC 2.4.1.-) (Mannosyltransferase ALG12 homolog) (hALG12) (Membrane protein SB87) (PP14673). [Source:Uniprot/SWISSPROT;Acc:Q9BV10] |
| ZNF588 | -0.44 | -0.71 | 0.94 | - | Zinc finger protein ZFD25. [Source:Uniprot/SWISSPROT;Acc:Q9UII5] |
| TCN1 | 0.25 | -0.71 | -0.05 | NM_001062 | Transcobalamin I precursor (TCI) (TC I). [Source:Uniprot/SWISSPROT;Acc:P20061] |
| NP_940844.1 | 1.24 | -0.71 | 1.75 | NM_198442 | - |
| - | 0.23 | -0.71 | -0.21 | - | 29 kDa protein [Source:IPI;Acc:IPI00479165] |
| - | 0.38 | -0.71 | -0.06 | XM_210400,XM_065445,XM_061880 | 17 kDa protein [Source:IPI;Acc:IPI00472320] |
| - | 0.78 | -0.71 | -0.13 | XM_497280,XM_372423 | - |
| - | -0.50 | -0.71 | -0.46 | - | Hypothetical protein |
| - | 0.06 | -0.71 | 0.58 | - | Similar to Peripheral benzodiazepine receptor interacting protein |
| - | -0.15 | -0.71 | -0.42 | XM_379280 | - |
| CITED1 | 0.44 | -0.71 | -0.04 | NM_004143 | Cbp/p300-interacting transactivator 1 (Melanocyte-specific protein 1). [Source:Uniprot/SWISSPROT;Acc:Q99966] |
| THRSP | 0.14 | -0.71 | 0.38 | NM_003251 | Thyroid hormone-inducible hepatic protein (Spot 14 protein) (SPOT14) (S14 protein). [Source:Uniprot/SWISSPROT;Acc:Q92748] |
| - | -0.02 | -0.71 | -0.05 | NR_001551 | - |
| GRIK1 | -0.32 | -0.71 | -0.21 | NM_000830 | Glutamate receptor, ionotropic kainate 1 precursor (Glutamate receptor 5) (GluR-5) (GluR5) (Excitatory amino acid receptor 3) (EAA3). [Source:Uniprot/SWISSPROT;Acc:P39086] |
| MCM8 | -0.39 | -0.71 | 0.09 | NM_032485,NM_182802 | DNA replication licensing factor MCM8 (Minichromosome maintenance 8). [Source:Uniprot/SWISSPROT;Acc:Q9UJA3] |
| OR6M1 | -0.24 | -0.71 | 0.23 | NM_001005325 | Olfactory receptor 6M1. [Source:Uniprot/SWISSPROT;Acc:Q8NGM8] |
| SLITRK5 | 0.35 | -0.71 | -0.06 | NM_015567 | SLIT and NTRK-like protein 5 precursor. [Source:Uniprot/SWISSPROT;Acc:O94991] |
| O51I2_HUMAN | 0.72 | -0.71 | 1.10 | NM_001004754 | Olfactory receptor 51I2 (HOR5'beta12). [Source:Uniprot/SWISSPROT;Acc:Q9H344] |
| - | 0.24 | -0.71 | 0.45 | XM_496908 | - |
| NP_001007562.1 | -0.27 | -0.71 | -0.33 | NM_001007561 | immunity-related GTPase family, Q1 [Source:RefSeq_peptide;Acc:NP_001007562] |
| KIR3DS1 | -0.17 | -0.71 | -0.20 | - | Killer cell immunoglobulin-like receptor 3DS1 precursor (MHC class I NK cell receptor) (Natural killer associated transcript 10) (NKAT-10). [Source:Uniprot/SWISSPROT;Acc:Q14943] |
| - | -0.24 | -0.71 | 0.07 | NM_001005915 | - |
| - | 0.11 | -0.71 | 0.36 | NM_001004482 | - |
| - | 1.59 | -0.71 | -0.44 | - | Hypothetical protein |
| SLC1A6 | 0.20 | -0.70 | 0.16 | NM_005071 | Excitatory amino acid transporter 4 (Sodium-dependent glutamate/aspartate transporter). [Source:Uniprot/SWISSPROT;Acc:P48664] |
| - | 0.26 | -0.70 | -0.18 | XM_380155,XM_379531 | - |
| - | 0.15 | -0.70 | -0.23 | XM_497921 | - |
| - | 0.17 | -0.70 | 0.15 | XM_370767 | - |
| Q8IYY5_HUMAN | -0.28 | -0.70 | -0.49 | NM_144717 | Interleukin-20 receptor beta chain precursor (IL-20R-beta) (IL-20R2) (UNQ557/PRO1114). [Source:Uniprot/SWISSPROT;Acc:Q6UXL0] |
| HAGHL | 0.25 | -0.70 | -0.11 | NM_032304,NM_207112 | hydroxyacylglutathione hydrolase-like isoform 2 [Source:RefSeq_peptide;Acc:NP_115680] |
| - | 0.03 | -0.70 | -0.05 | XM_114090,XM_114047 | - |
| IL13RA2 | -0.42 | -0.70 | 0.18 | NM_000640 | Interleukin-13 receptor alpha-2 chain precursor (Interleukin-13 binding protein). [Source:Uniprot/SWISSPROT;Acc:Q14627] |
| BAG4 | 0.21 | -0.70 | -0.47 | NM_004874 | BAG-family molecular chaperone regulator-4 (Silencer of death domains). [Source:Uniprot/SWISSPROT;Acc:O95429] |
| NP_848130.1 | -0.13 | -0.70 | 1.02 | NM_178353 | late cornified envelope 1E [Source:RefSeq_peptide;Acc:NP_848130] |
| Q96PX9_HUMAN | -0.43 | -0.70 | -0.21 | - | - |
| XP_498464.1 | 0.37 | -0.70 | -0.10 | XM_498464,XM_499594 | PREDICTED: hypothetical protein XP_499594 [Source:RefSeq_peptide;Acc:XP_499594] |
| - | 0.13 | -0.70 | 0.48 | - | 22 kDa protein [Source:IPI;Acc:IPI00413524] |
| Q6ZT86_HUMAN | -0.20 | -0.70 | 0.05 | - | - |
| PHF7 | 0.10 | -0.70 | 0.25 | NM_016483 | PHD finger protein 7 isoform 1 [Source:RefSeq_peptide;Acc:NP_057567] |
| TFB1M | 0.08 | -0.70 | 0.05 | NM_016020 | transcription factor B1, mitochondrial [Source:RefSeq_peptide;Acc:NP_057104] |
| IRX2 | 0.04 | -0.70 | -0.03 | NM_033267 | Iroquois-class homeodomain protein IRX-2 (Iroquois homeobox protein 2) (Homeodomain protein IRXA2). [Source:Uniprot/SWISSPROT;Acc:Q9BZI1] |
| NP_115749.2 | 0.03 | -0.70 | 0.40 | NM_032373 | polycomb group ring finger 5 [Source:RefSeq_peptide;Acc:NP_115749] |
| - | 0.72 | -0.70 | 0.05 | XM_373986 | - |
| JOS2_HUMAN | 0.10 | -0.70 | 0.41 | NM_138334 | Josephin 2 (SBBI54). [Source:Uniprot/SWISSPROT;Acc:Q8TAC2] |
| Q9NSC1_HUMAN | 0.26 | -0.70 | 0.14 | NM_004838 | Homer protein homolog 3 (Homer-3). [Source:Uniprot/SWISSPROT;Acc:Q9NSC5] |
| TDRD9 | 0.57 | -0.70 | 0.56 | - | tudor domain containing 9 [Source:RefSeq_peptide;Acc:NP_694591] |
| ZCWCC2 | 0.64 | -0.70 | 0.19 | NM_024657 | Zinc finger CW-type coiled-coil domain protein 2. [Source:Uniprot/SWISSPROT;Acc:Q8TE76] |
| - | 0.86 | -0.70 | -0.35 | XM_497068 | - |
| NP_997337.1 | -0.08 | -0.69 | 0.30 | NM_207454 | - |
| NP_149113.2 | 0.21 | -0.69 | 0.62 | NM_033122 | testis development protein NYD-SP26 [Source:RefSeq_peptide;Acc:NP_149113] |
| O43838_HUMAN | 0.11 | -0.69 | 0.18 | NM_001300 | Core promoter element-binding protein (Kruppel-like factor 6) (B-cell derived protein 1) (Proto-oncogene BCD1) (Transcription factor Zf9) (GC-rich sites binding factor GBF). [Source:Uniprot/SWISSPROT;Acc:Q99612] |
| PFC | -0.16 | -0.69 | 1.16 | NM_002621 | Properdin precursor (Factor P). [Source:Uniprot/SWISSPROT;Acc:P27918] |
| MORC | 0.50 | -0.69 | -0.25 | NM_014429 | microrchidia homolog [Source:RefSeq_peptide;Acc:NP_055244] |
| - | -0.12 | -0.69 | 0.00 | - | Similar to Testis-specific testis transcript Y 1 (Fragment) |
| EIF4E3 | -0.09 | -0.69 | -0.46 | XM_497923 | EIF4E3 protein. [Source:Uniprot/SPTREMBL;Acc:Q6NUT1] |
| - | -0.23 | -0.69 | -0.18 | XM_379430 | - |
| CPB1 | 0.30 | -0.69 | 0.38 | NM_001871 | Carboxypeptidase B precursor (EC 3.4.17.2) (Pancreas-specific protein) (PASP). [Source:Uniprot/SWISSPROT;Acc:P15086] |
| BTN1A1 | 0.39 | -0.69 | -0.21 | NM_001732 | Butyrophilin subfamily 1 member A1 precursor (BT). [Source:Uniprot/SWISSPROT;Acc:Q13410] |
| CD8B1 | 0.08 | -0.69 | -0.02 | NM_004931,NM_172099 | T-cell surface glycoprotein CD8 beta chain precursor (Antigen CD8B). [Source:Uniprot/SWISSPROT;Acc:P10966] |
| - | 1.35 | -0.69 | 1.42 | XM_497877 | - |
| KLRF1 | 0.00 | -0.69 | -0.36 | NM_016523 | Killer cell lectin-like receptor subfamily F, member 1 (Lectin-like receptor F1) (Activating coreceptor NKp80). [Source:Uniprot/SWISSPROT;Acc:Q9NZS2] |
| XP_294590.1 | 0.23 | -0.69 | -0.23 | XM_294590 | PREDICTED: similar to bA13B9.3 (novel protein similar to KRT8) [Source:RefSeq_peptide;Acc:XP_294590] |
| - | -0.42 | -0.69 | -0.35 | - | 64 kDa protein [Source:IPI;Acc:IPI00479910] |
| NP_997277.1 | -0.17 | -0.69 | -0.19 | NM_207394 | - |
| - | -0.07 | -0.69 | -0.30 | XM_496859,XM_499273 | - |
| Q6ZTW8_HUMAN | 0.70 | -0.69 | 0.46 | NM_025228 | TRAF3-interacting JNK-activating modulator. [Source:Uniprot/SWISSPROT;Acc:Q9Y228] |
| DAZL | 1.68 | -0.69 | 0.80 | NM_001351 | Deleted in azoospermia-like (DAZ-like autosomal) (Deleted in azoospermia-like 1) (DAZ homolog) (SPGY-like-autosomal). [Source:Uniprot/SWISSPROT;Acc:Q92904] |
| NP_660301.1 | 0.28 | -0.69 | -0.03 | NM_145258 | - |
| SOCS6 | 0.43 | -0.69 | 0.42 | NM_004232 | Suppressor of cytokine signaling 6 (Suppressor of cytokine signaling 4) (SOCS-4) (Cytokine-inducible SH2 protein 4) (CIS-4). [Source:Uniprot/SWISSPROT;Acc:O14544] |
| LSLCL_HUMAN | 0.11 | -0.69 | 0.41 | NM_002975 | Stem cell growth factor precursor (Lymphocyte secreted C-type lectin) (p47) (C-type lectin superfamily member 3). [Source:Uniprot/SWISSPROT;Acc:Q9Y240] |
| BTF3L1 | -0.47 | -0.69 | 0.33 | - | Transcription factor BTF3 homolog 1. [Source:Uniprot/SWISSPROT;Acc:Q13890] |
| - | -0.47 | -0.69 | 0.17 | - | 12 kDa protein [Source:IPI;Acc:IPI00479653] |
| XP_086725.2 | 0.52 | -0.68 | 0.37 | XM_086725 | PREDICTED: similar to bB329D4.2.1 (novel protein similar to a truncated nuclear receptor co-repressor 1 (NCOR1) (retinoid X receptor interacting protein 13), isoform 1) [Source:RefSeq_peptide;Acc:XP_086725] |
| ZIC4 | 0.00 | -0.68 | -0.23 | NM_032153 | Zinc finger protein ZIC 4 (Zinc finger protein of the cerebellum 4) (Fragment). [Source:Uniprot/SWISSPROT;Acc:Q8N9L1] |
| NGEF | -0.49 | -0.68 | -0.35 | NM_019850 | neuronal guanine nucleotide exchange factor [Source:RefSeq_peptide;Acc:NP_062824] |
| COL8A1 | -0.19 | -0.68 | 0.72 | NM_001850,NM_020351 | Smooth muscle cell-expressed and macrophage conditioned medium-induced protein 64 (Smag-64). [Source:Uniprot/SWISSPROT;Acc:Q9NRT5] |
| - | 0.52 | -0.68 | 2.15 | XM_497554 | - |
| - | -0.27 | -0.68 | -0.23 | XM_498475 | - |
| ANKK1 | 0.20 | -0.68 | 0.38 | NM_178510 | ankyrin repeat and kinase domain containing 1 [Source:RefSeq_peptide;Acc:NP_848605] |
| VGLL2 | 0.34 | -0.68 | -0.12 | NM_182645 | Transcription cofactor vestigial-like protein 2 (Vgl-2) (VITO1 protein). [Source:Uniprot/SWISSPROT;Acc:Q8N8G2] |
| - | -0.20 | -0.68 | 1.21 | - | Hypothetical protein |
| GPR146 | 0.24 | -0.68 | -0.43 | NM_138445 | G protein-coupled receptor 146 [Source:RefSeq_peptide;Acc:NP_612454] |
| - | -0.24 | -0.68 | -0.26 | NM_024653 | - |
| - | 0.08 | -0.68 | -0.04 | XR_000208 | - |
| - | -0.47 | -0.68 | 0.36 | XM_380160,XM_379535 | - |
| - | -0.07 | -0.68 | 1.72 | - | Hypothetical protein |
| MKNK1 | -0.02 | -0.68 | -0.04 | - | MAP kinase-interacting serine/threonine kinase 1 (EC 2.7.1.37) (MAP kinase signal-integrating kinase 1) (Mnk1). [Source:Uniprot/SWISSPROT;Acc:Q9BUB5] |
| XP_068632.2 | 1.00 | -0.68 | 0.70 | XM_068632 | PREDICTED: hypothetical protein XP_068632 [Source:RefSeq_peptide;Acc:XP_068632] |
| GLRA3 | -0.24 | -0.68 | -0.44 | NM_006529 | Glycine receptor alpha-3 chain precursor. [Source:Uniprot/SWISSPROT;Acc:O75311] |
| Q9UI61_HUMAN | 0.17 | -0.68 | -0.07 | - | - |
| - | 0.12 | -0.68 | -0.50 | NM_020995 | - |
| Q6NUR6_HUMAN | 0.79 | -0.68 | -0.10 | - | - |
| Q8N9E6_HUMAN | -0.13 | -0.68 | 0.35 | NM_175908 | - |
| - | 0.44 | -0.68 | 0.68 | - | Hypothetical protein |
| MYST4 | -0.18 | -0.68 | 0.15 | NM_012330 | MYST histone acetyltransferase (monocytic leukemia) 4 [Source:RefSeq_peptide;Acc:NP_036462] |
| SERPINA7 | -0.41 | -0.68 | -0.12 | NM_000354 | Thyroxine-binding globulin precursor (T4-binding globulin). [Source:Uniprot/SWISSPROT;Acc:P05543] |
| U2AF1L1 | 0.02 | -0.68 | 0.22 | - | U2 small nuclear ribonucleoprotein auxiliary factor 35 kDa subunit related-protein 1 (U2(RNU2) small nuclear RNA auxillary factor 1-like 1). [Source:Uniprot/SWISSPROT;Acc:Q15695] |
| SELV_HUMAN | -0.01 | -0.68 | -0.09 | NM_182704 | Selenoprotein V. [Source:Uniprot/SWISSPROT;Acc:P59797] |
| ABCA10 | 0.08 | -0.67 | 0.48 | NM_080282 | ATP-binding cassette, sub-family A, member 10 [Source:RefSeq_peptide;Acc:NP_525021] |
| ACTH_HUMAN | 0.52 | -0.67 | 0.05 | NM_001615 | Actin, gamma-enteric smooth muscle (Smooth muscle gamma actin) (Alpha- actin 3). [Source:Uniprot/SWISSPROT;Acc:P63267] |
| C20orf181 | -0.10 | -0.67 | 0.18 | - | OTTHUMP00000031602 (Fragment). [Source:Uniprot/SPTREMBL;Acc:Q9H1S0] |
| NP_060316.1 | 0.72 | -0.67 | -0.41 | NM_017846 | tRNA selenocysteine associated protein [Source:RefSeq_peptide;Acc:NP_060316] |
| - | 0.25 | -0.67 | 0.00 | XM_379243 | - |
| - | 0.25 | -0.67 | -0.34 | XM_496835,XM_496331 | - |
| - | 0.22 | -0.67 | 0.55 | XM_059061 | - |
| - | 1.08 | -0.67 | -0.04 | XM_373685 | - |
| LGR7 | 0.90 | -0.67 | -0.44 | NM_021634 | Relaxin receptor 1 (Leucine-rich repeat-containing G-protein coupled receptor 7). [Source:Uniprot/SWISSPROT;Acc:Q9HBX9] |
| EVX2 | -0.47 | -0.67 | 0.73 | XM_292968 | Homeobox even-skipped homolog protein 2 (EVX-2). [Source:Uniprot/SWISSPROT;Acc:Q03828] |
| EFA6R_HUMAN | -0.16 | -0.67 | 0.33 | NM_206909 | Pleckstrin and Sec7 domain containing protein 3 (Exchange factor for ADP-ribosylation factor guanine nucleotide factor 6) (Hepatocellular carcinoma-associated antigen 67). [Source:Uniprot/SWISSPROT;Acc:Q9NYI0] |
| BRAF | -0.26 | -0.67 | -0.33 | NM_004333 | B-Raf proto-oncogene serine/threonine-protein kinase (EC 2.7.1.37) (p94) (v-Raf murine sarcoma viral oncogene homolog B1). [Source:Uniprot/SWISSPROT;Acc:P15056] |
| HOME2_HUMAN | -0.22 | -0.67 | -0.05 | NM_199330,NM_199331,NM_004839,NM_199332 | Homer protein homolog 2 (Homer-2). [Source:Uniprot/SWISSPROT;Acc:Q9NSB8] |
| MDN1 | 0.33 | -0.67 | 1.29 | NM_014611 | Midasin (MIDAS-containing protein). [Source:Uniprot/SWISSPROT;Acc:Q9NU22] |
| OR5E1P | 0.63 | -0.67 | 0.01 | - | Putative olfactory receptor (Fragment). [Source:Uniprot/SPTREMBL;Acc:Q15616] |
| CEP63_HUMAN | -0.08 | -0.67 | 0.59 | NM_025180 | Centrosomal protein of 63 kDa (Cep63 protein). [Source:Uniprot/SWISSPROT;Acc:Q96MT8] |
| NP_848586.1 | 0.69 | -0.67 | 0.02 | NM_178491 | R3H domain (binds single-stranded nucleic acids) containing-like [Source:RefSeq_peptide;Acc:NP_848586] |
| NP_775813.1 | 0.34 | -0.67 | -0.41 | NM_173542 | - |
| - | -0.02 | -0.67 | -0.46 | NM_012188,XM_292958,NM_144769,NM_207426 | - |
| - | -0.16 | -0.67 | -0.48 | - | Non-protein coding transcript |
| TNFRSF1B | 1.06 | -0.67 | 0.16 | NM_001066 | Tumor necrosis factor receptor superfamily member 1B precursor (Tumor necrosis factor receptor 2) (TNF-R2) (Tumor necrosis factor receptor type II) (p75) (p80 TNF-alpha receptor) (CD120b) (Etanercept) [Contains: Tumor necrosis factor binding protein 2 (TB |
| NP_001006609.1 | 0.07 | -0.67 | -0.37 | NM_001006608 | - |
| - | 0.13 | -0.67 | 1.48 | XM_373028 | - |
| - | 1.21 | -0.67 | -0.20 | NM_001008234 | - |
| NP_776185.1 | -0.24 | -0.67 | -0.08 | NM_173824 | - |
| Q96HG9_HUMAN | -0.25 | -0.67 | -0.34 | - | - |
| XP_166227.3 | -0.14 | -0.67 | 0.20 | XM_166227 | PREDICTED: macrophage expressed gene 1 [Source:RefSeq_peptide;Acc:XP_166227] |
| XP_066859.3 | -0.24 | -0.67 | 0.02 | XM_066859 | PREDICTED: similar to zinc finger protein 92 [Source:RefSeq_peptide;Acc:XP_066859] |
| NP_061134.1 | -0.11 | -0.67 | -0.48 | NM_018664 | Jun dimerization protein p21SNFT [Source:RefSeq_peptide;Acc:NP_061134] |
| HRBL | -0.33 | -0.67 | -0.39 | - | HIV-1 Rev binding protein-like protein (Rev/Rex activation domain binding protein-related) (RAB-R). [Source:Uniprot/SWISSPROT;Acc:O95081] |
| - | 0.10 | -0.67 | 0.63 | - | Non-protein coding transcript |
| XP_499395.1 | 1.06 | -0.66 | 0.82 | XM_499395 | PREDICTED: similar to galectin-related inter-fiber protein [Source:RefSeq_peptide;Acc:XP_499395] |
| KPNA1 | 0.32 | -0.66 | 0.99 | NM_002264 | Importin alpha-1 subunit (Karyopherin alpha-1 subunit) (SRP1-beta) (RAG cohort protein 2) (Nucleoprotein interactor 1) (NPI-1). [Source:Uniprot/SWISSPROT;Acc:P52294] |
| - | 0.52 | -0.66 | 1.00 | - | Hypothetical protein |
| ZNF79 | 1.23 | -0.66 | -0.02 | NM_007135 | Zinc finger protein 79 (ZNFpT7) (Fragment). [Source:Uniprot/SWISSPROT;Acc:Q15937] |
| PRKCE | -0.11 | -0.66 | -0.42 | NM_005400 | Protein kinase C, epsilon type (EC 2.7.1.-) (nPKC-epsilon). [Source:Uniprot/SWISSPROT;Acc:Q02156] |
| PNMA6A | 0.40 | -0.66 | 0.27 | NM_032882 | paraneoplastic antigen like 6A [Source:RefSeq_peptide;Acc:NP_116271] |
| - | 0.27 | -0.66 | -0.33 | - | Hypothetical protein |
| NMES1_HUMAN | -0.16 | -0.66 | -0.02 | NM_032413,NM_197955 | Normal mucosa of esophagus specific gene 1 protein (FOAP-11 protein). [Source:Uniprot/SWISSPROT;Acc:Q9C002] |
| SNAI1 | 0.85 | -0.66 | 0.27 | NM_005985 | Zinc finger protein SNAI1 (Snail protein homolog) (Sna protein). [Source:Uniprot/SWISSPROT;Acc:O95863] |
| - | -0.01 | -0.66 | 0.48 | XM_208847,XM_497431 | Similar to Testicular metalloprotease-like, disintegrin-like, cysteine-rich protein IVb [Source:IPI;Acc:IPI00552096] |
| - | 0.70 | -0.66 | -0.39 | NM_014694 | 35 kDa protein [Source:IPI;Acc:IPI00477776] |
| CACNG1 | -0.04 | -0.66 | -0.01 | NM_000727 | Voltage-dependent calcium channel gamma-1 subunit (Dihydropyridine- sensitive L-type, skeletal muscle calcium channel gamma subunit). [Source:Uniprot/SWISSPROT;Acc:Q06432] |
| - | 0.97 | -0.66 | 0.07 | XM_498284 | - |
| YG96_HUMAN | -0.10 | -0.66 | -0.31 | NM_015703 | - |
| - | 0.61 | -0.66 | 1.28 | XM_374254 | - |
| GPR24 | 0.25 | -0.66 | -0.14 | NM_005297 | Melanin-concentrating hormone receptor 1 (MCH receptor 1) (MCHR-1) (MCH-R1) (MCH1R) (MCH-1R) (MCHR) (G-protein coupled receptor 24) (Somatostatin receptor-like protein) (SLC-1). [Source:Uniprot/SWISSPROT;Acc:Q99705] |
| Q9NY04_HUMAN | 1.01 | -0.66 | 1.72 | - | - |
| Q9NSG4_HUMAN | -0.09 | -0.66 | 0.06 | NM_018087 | - |
| NP_852114.1 | -0.07 | -0.66 | 0.41 | NM_181449 | immune receptor expressed on myeloid cells 2 [Source:RefSeq_peptide;Acc:NP_852114] |
| CRYBA2 | 0.05 | -0.66 | 0.38 | NM_005209,NM_057094,NM_057093 | Beta crystallin A2 (Beta-A2-crystallin). [Source:Uniprot/SWISSPROT;Acc:P53672] |
| WFDC11 | 0.00 | -0.66 | 0.08 | NM_147197 | Protein WFDC11 precursor. [Source:Uniprot/SWISSPROT;Acc:Q8NEX6] |
| CNTN1 | 0.32 | -0.66 | 4.04 | NM_175038,NM_001843 | Contactin 1 precursor (Neural cell surface protein F3) (Glycoprotein gp135). [Source:Uniprot/SWISSPROT;Acc:Q12860] |
| FGF5 | 0.63 | -0.66 | -0.41 | NM_004464,NM_033143 | Fibroblast growth factor-5 precursor (FGF-5) (HBGF-5) (Smag-82). [Source:Uniprot/SWISSPROT;Acc:P12034] |
| CD163 | -0.37 | -0.66 | 0.47 | NM_004244,NM_203416 | CD163 antigen isoform b [Source:RefSeq_peptide;Acc:NP_981961] |
| - | 0.25 | -0.66 | -0.47 | XM_060054 | - |
| - | -0.41 | -0.66 | -0.49 | - | Hypothetical protein |
| KIAA1189 | 0.12 | -0.65 | -0.17 | XM_371576 | - |
| ZNF345 | -0.04 | -0.65 | 0.54 | NM_003419 | Zinc finger protein 345 (Zinc finger protein HZF10). [Source:Uniprot/SWISSPROT;Acc:Q14585] |
| - | -0.31 | -0.65 | 0.22 | - | Non-protein coding transcript |
| - | -0.05 | -0.65 | 0.81 | XM_496886,XM_499307 | - |
| - | -0.37 | -0.65 | -0.05 | XM_498645 | - |
| - | 1.16 | -0.65 | 0.02 | - | Non-protein coding transcript |
| NMBR | -0.16 | -0.65 | 0.38 | NM_002511 | Neuromedin-B receptor (NMB-R) (Neuromedin-B-preferring bombesin receptor). [Source:Uniprot/SWISSPROT;Acc:P28336] |
| PRKCB1 | -0.42 | -0.65 | -0.39 | NM_212535 | Protein kinase C, beta type (EC 2.7.1.37) (PKC-beta) (PKC-B). [Source:Uniprot/SWISSPROT;Acc:P05771] |
| KIF18A | -0.31 | -0.65 | -0.13 | NM_031217 | kinesin family member 18A [Source:RefSeq_peptide;Acc:NP_112494] |
| Q8N8H9_HUMAN | 0.40 | -0.65 | -0.03 | - | - |
| ENPP6 | 0.70 | -0.65 | 0.36 | NM_153343 | ectonucleotide pyrophosphatase/phosphodiesterase 6 [Source:RefSeq_peptide;Acc:NP_699174] |
| PTPRH | 1.27 | -0.65 | -0.36 | NM_002842 | protein tyrosine phosphatase, receptor type, H precursor [Source:RefSeq_peptide;Acc:NP_002833] |
| SARS2 | 0.45 | -0.65 | 0.10 | NM_017827 | Seryl-tRNA synthetase, mitochondrial precursor (EC 6.1.1.11) (Serine-- tRNA ligase) (SerRSmt). [Source:Uniprot/SWISSPROT;Acc:Q9NP81] |
| KIAA0179 | -0.49 | -0.65 | -0.35 | NM_015056 | - |
| NP_660151.1 | 0.24 | -0.65 | -0.31 | - | NAD(P) dependent steroid dehydrogenase-like [Source:RefSeq_peptide;Acc:NP_660151] |
| CYP3A7 | 0.28 | -0.65 | 0.99 | - | Cytochrome P450 3A7 (EC 1.14.14.1) (CYPIIIA7) (P450-HFLA). [Source:Uniprot/SWISSPROT;Acc:P24462] |
| XP_371167.1 | 0.08 | -0.65 | 0.30 | XM_371167 | PREDICTED: similar to Syncollin [Source:RefSeq_peptide;Acc:XP_371167] |
| - | -0.08 | -0.65 | 2.00 | - | 75 kDa protein [Source:IPI;Acc:IPI00479171] |
| SLC5A7 | 0.40 | -0.65 | 0.13 | NM_021815 | High-affinity choline transporter 1 (Solute carrier family 5 member 7) (Hemicholinium-3-sensitive choline transporter) (CHT). [Source:Uniprot/SWISSPROT;Acc:Q9GZV3] |
| MGAT5 | 0.40 | -0.65 | -0.45 | NM_002410 | Alpha-1,6-mannosylglycoprotein 6-beta-N-acetylglucosaminyltransferase V (EC 2.4.1.155) (Alpha-mannoside beta-1,6-N- acetylglucosaminyltransferase) (N-acetylglucosaminyl-transferase V) (GNT-V) (GlcNAc-T V). [Source:Uniprot/SWISSPROT;Acc:Q09328] |
| XP_377426.1 | 1.15 | -0.65 | -0.43 | XM_377426 | PREDICTED: similar to IGHV gene product [Source:RefSeq_peptide;Acc:XP_377426] |
| - | -0.12 | -0.65 | 0.06 | NR_001550 | - |
| NP_653226.2 | 0.16 | -0.65 | 0.67 | NM_144625 | - |
| PAMCI | 0.43 | -0.65 | 0.30 | NM_005447 | peptidylglycine alpha-amidating monooxygenase COOH-terminal interactor [Source:RefSeq_peptide;Acc:NP_005438] |
| ALS2CR8 | 0.55 | -0.65 | -0.14 | NM_024744 | amyotrophic lateral sclerosis 2 (juvenile) chromosome region, candidate 8 [Source:RefSeq_peptide;Acc:NP_079020] |
| - | -0.09 | -0.65 | 0.05 | - | Hypothetical protein |
| CYP4F2 | 0.34 | -0.65 | 0.84 | NM_001082 | Cytochrome P450 4F2 (EC 1.14.13.30) (CYPIVF2) (Leukotriene-B(4) omega- hydroxylase) (Leukotriene-B(4) 20-monooxygenase) (Cytochrome P450-LTB- omega). [Source:Uniprot/SWISSPROT;Acc:P78329] |
| NP_690876.2 | -0.36 | -0.65 | 0.63 | NM_152912 | mitochondrial translational initiation factor 3 [Source:RefSeq_peptide;Acc:NP_690876] |
| ZNF142 | -0.29 | -0.65 | 0.20 | NM_005081 | Zinc finger protein 142 (HA4654). [Source:Uniprot/SWISSPROT;Acc:P52746] |
| NP_997319.1 | 0.23 | -0.65 | -0.05 | NM_207436 | - |
| - | 0.30 | -0.65 | 0.52 | - | Hypothetical protein |
| CACNG3 | -0.31 | -0.65 | 0.86 | NM_006539 | Voltage-dependent calcium channel gamma-3 subunit (Neuronal voltage- gated calcium channel gamma-3 subunit). [Source:Uniprot/SWISSPROT;Acc:O60359] |
| GCKR | -0.22 | -0.65 | -0.08 | NM_001486 | Glucokinase regulatory protein (Glucokinase regulator). [Source:Uniprot/SWISSPROT;Acc:Q14397] |
| PRICKLE1 | 0.08 | -0.65 | -0.33 | - | Prickle-like protein 1 (REST/NRSF-interacting LIM domain protein 1). [Source:Uniprot/SWISSPROT;Acc:Q96MT3] |
| - | -0.03 | -0.65 | 0.78 | NM_004877 | - |
| Q9H762_HUMAN | 0.00 | -0.65 | 0.66 | - | - |
| - | 1.21 | -0.65 | 0.20 | NM_001001794 | - |
| - | 0.40 | -0.65 | 0.39 | - | Hypothetical protein |
| RAPGEF4 | 0.52 | -0.64 | 0.02 | NM_007023 | Rap guanine nucleotide exchange factor 4 (cAMP-regulated guanine nucleotide exchange factor II) (cAMP-GEFII) (Exchange factor directly activated by cAMP 2) (Epac 2). [Source:Uniprot/SWISSPROT;Acc:Q8WZA2] |
| XP_496156.1 | 0.79 | -0.64 | 0.30 | - | PREDICTED: similar to rhophilin-like protein; RhoB effector; rhophilin-2; rhophilin 2 [Source:RefSeq_peptide;Acc:XP_496156] |
| OR5M8 | 0.00 | -0.64 | -0.32 | NM_001005282 | Olfactory receptor 5M8. [Source:Uniprot/SWISSPROT;Acc:Q8NGP6] |
| VINEX_HUMAN | -0.06 | -0.64 | -0.25 | NM_005775 | Vinexin (SH3-containing adapter molecule-1) (SCAM-1). [Source:Uniprot/SWISSPROT;Acc:O60504] |
| Q9H9J1_HUMAN | 0.47 | -0.64 | -0.44 | NM_014388 | - |
| ISL2 | -0.04 | -0.64 | -0.27 | NM_145805 | Insulin gene enhancer protein ISL-2 (Islet-2). [Source:Uniprot/SWISSPROT;Acc:Q96A47] |
| ADH1B | -0.38 | -0.64 | -0.20 | NM_000668 | Alcohol dehydrogenase gamma chain (EC 1.1.1.1). [Source:Uniprot/SWISSPROT;Acc:P00326] |
| - | -0.38 | -0.64 | -0.26 | XM_060880 | 39 kDa protein [Source:IPI;Acc:IPI00411658] |
| TMEM16C | -0.02 | -0.64 | -0.36 | NM_031418 | Transmembrane protein 16C. [Source:Uniprot/SWISSPROT;Acc:Q9BYT9] |
| - | -0.13 | -0.64 | -0.16 | XM_379716 | - |
| RLBP1 | -0.09 | -0.64 | -0.26 | NM_000326 | Cellular retinaldehyde-binding protein (CRALBP). [Source:Uniprot/SWISSPROT;Acc:P12271] |
| MLNR | 0.01 | -0.64 | -0.44 | NM_001507 | Motilin receptor (G-protein coupled receptor 38). [Source:Uniprot/SWISSPROT;Acc:O43193] |
| CDK3 | -0.46 | -0.64 | -0.17 | NM_001258 | Cell division protein kinase 3 (EC 2.7.1.37). [Source:Uniprot/SWISSPROT;Acc:Q00526] |
| - | 0.50 | -0.64 | -0.02 | NM_020880 | - |
| KR108_HUMAN | 0.12 | -0.64 | -0.07 | NM_198695 | Keratin-associated protein 10-8 (Keratin-associated protein 10.8) (High sulfur keratin-associated protein 10.8) (Keratin-associated protein 18-8) (Keratin-associated protein 18.8). [Source:Uniprot/SWISSPROT;Acc:P60410] |
| - | -0.03 | -0.64 | -0.39 | NM_173858 | - |
| - | -0.39 | -0.64 | -0.38 | - | 50 kDa protein [Source:IPI;Acc:IPI00478542] |
| C2orf4 | 0.33 | -0.64 | 0.11 | NM_015955,XM_498862,XM_371413 | (CGI-27) (C21orf19-like protein). [Source:Uniprot/SWISSPROT;Acc:Q9Y316] |
| - | 0.25 | -0.64 | 0.92 | NM_001004741 | - |
| - | -0.32 | -0.64 | -0.04 | - | Hypothetical protein |
| NP_653217.1 | 1.08 | -0.64 | -0.24 | NM_144616 | - |
| Q71F78_HUMAN | 0.84 | -0.64 | -0.20 | - | Lung carcinoma-associated protein. [Source:Uniprot/SPTREMBL;Acc:Q71F78] |
| - | 0.70 | -0.64 | 0.08 | - | Non-protein coding transcript |
| PYY2 | -0.30 | -0.64 | 1.42 | NM_021093 | peptide YY, 2 (seminalplasmin) [Source:RefSeq_peptide;Acc:NP_066579] |
| MRGX4_HUMAN | -0.27 | -0.64 | -0.32 | NM_054032 | Sensory neuron-specific G-protein coupled receptor 5. [Source:Uniprot/SWISSPROT;Acc:Q8TDD7] |
| CD3E | -0.46 | -0.64 | -0.19 | NM_000733 | T-cell surface glycoprotein CD3 epsilon chain precursor (T-cell surface antigen T3/Leu-4 epsilon chain). [Source:Uniprot/SWISSPROT;Acc:P07766] |
| SYT2 | 0.19 | -0.64 | 0.52 | NM_177402 | Synaptotagmin-2 (Synaptotagmin II) (SytII). [Source:Uniprot/SWISSPROT;Acc:Q8N9I0] |
| NPM2_HUMAN | 0.26 | -0.64 | 0.56 | NM_182795 | Nucleoplasmin 2. [Source:Uniprot/SWISSPROT;Acc:Q86SE8] |
| Q9NWJ7_HUMAN | 2.60 | -0.64 | 0.24 | - | - |
| - | -0.42 | -0.64 | 0.96 | - | 16 kDa protein [Source:IPI;Acc:IPI00411319] |
| NP_997290.1 | 0.31 | -0.64 | -0.06 | NM_207407 | - |
| NP_775862.1 | 0.62 | -0.64 | -0.39 | NM_173591 | - |
| RGL1 | 0.93 | -0.64 | -0.25 | NM_015149 | Ral guanine nucleotide dissociation stimulator-like 1 (RalGDS-like 1). [Source:Uniprot/SWISSPROT;Acc:Q9NZL6] |
| NP_872298.1 | 0.83 | -0.64 | -0.48 | NM_182492 | - |
| NP_689782.1 | -0.06 | -0.64 | 0.41 | NM_152569 | - |
| BCL2A1 | 0.36 | -0.63 | 0.39 | NM_004049 | Bcl-2-related protein A1 (BFL-1 protein) (Hemopoietic-specific early response protein) (GRS protein). [Source:Uniprot/SWISSPROT;Acc:Q16548] |
| Q9BX40_HUMAN | -0.31 | -0.63 | 0.45 | - | - |
| SMC1L2 | -0.34 | -0.63 | 0.23 | NM_148674 | Structural maintenance of chromosome 1-like 2 protein (SMC1beta protein). [Source:Uniprot/SWISSPROT;Acc:Q8NDV3] |
| - | -0.05 | -0.63 | 2.04 | - | 26 kDa protein [Source:IPI;Acc:IPI00332247] |
| NP_699190.1 | 0.63 | -0.63 | 0.52 | NM_153359 | - |
| NP_115958.2 | 0.63 | -0.63 | 0.12 | NM_032569 | cytokine-like nuclear factor n-pac [Source:RefSeq_peptide;Acc:NP_115958] |
| ANXA8 | 0.28 | -0.63 | 0.22 | - | Annexin A8 (Annexin VIII) (Vascular anticoagulant-beta) (VAC-beta). [Source:Uniprot/SWISSPROT;Acc:P13928] |
| MMP23A | -0.03 | -0.63 | 0.40 | NM_006983,NM_004659 | matrix metalloproteinase 23B [Source:RefSeq_peptide;Acc:NP_008914] |
| NP_955383.1 | -0.33 | -0.63 | 0.07 | - | - |
| RANBP10 | -0.14 | -0.63 | 0.11 | NM_020850 | RAN binding protein 10 [Source:RefSeq_peptide;Acc:NP_065901] |
| ENK13_HUMAN | 0.81 | -0.63 | -0.28 | - | - |
| RNF17 | 0.12 | -0.63 | -0.18 | NM_019038 | RING finger protein 17. [Source:Uniprot/SWISSPROT;Acc:Q9BXT8] |
| RGL2 | -0.30 | -0.63 | 0.07 | NM_004761 | Ral guanine nucleotide dissociation stimulator-like 2 (RalGDS-like factor) (RAS-associated protein RAB2L). [Source:Uniprot/SWISSPROT;Acc:O15211] |
| - | -0.04 | -0.63 | 0.28 | - | 31 kDa protein [Source:IPI;Acc:IPI00334809] |
| NP_997309.1 | 0.39 | -0.63 | 0.20 | NM_207426 | - |
| - | 0.16 | -0.63 | 0.04 | XM_498890 | - |
| Q9H9R9_HUMAN | -0.08 | -0.63 | -0.28 | NM_024043 | - |
| - | -0.49 | -0.63 | 0.79 | XM_373750 | - |
| APOBEC3A | -0.06 | -0.63 | -0.32 | NM_145699 | Probable DNA dC->dU editing enzyme APOBEC-3A (EC 3.5.4.-) (Phorbolin 1). [Source:Uniprot/SWISSPROT;Acc:P31941] |
| NP_659493.1 | 0.28 | -0.63 | -0.15 | NM_145056 | thymus expressed gene 3-like [Source:RefSeq_peptide;Acc:NP_659493] |
| - | 0.22 | -0.63 | 0.25 | - | Hypothetical protein |
| WDR23_HUMAN | 0.01 | -0.63 | -0.01 | NM_025230,NM_181357 | WD-repeat protein 23 (GL014) (PRO2389). [Source:Uniprot/SWISSPROT;Acc:Q8TEB1] |
| SLC4A1 | 0.57 | -0.63 | -0.10 | NM_000342 | Band 3 anion transport protein (Anion exchange protein 1) (AE 1) (CD233 antigen). [Source:Uniprot/SWISSPROT;Acc:P02730] |
| - | 0.37 | -0.63 | 0.75 | XM_373822 | - |
| - | -0.38 | -0.63 | -0.43 | - | Non-protein coding transcript |
| CYP4A11 | -0.31 | -0.63 | -0.19 | NM_000778 | Cytochrome P450 4A11 precursor (EC 1.14.15.3) (CYPIVA11) (Fatty acid omega-hydroxylase) (P-450 HK omega) (Lauric acid omega-hydroxylase) (CYP4AII) (P450-HL-omega). [Source:Uniprot/SWISSPROT;Acc:Q02928] |
| FXYD4 | 0.04 | -0.63 | 0.04 | NM_173160 | FXYD domain-containing ion transport regulator 4 precursor. [Source:Uniprot/SWISSPROT;Acc:P59646] |
| PCDH15 | -0.45 | -0.63 | -0.46 | NM_033056 | Protocadherin 15 precursor. [Source:Uniprot/SWISSPROT;Acc:Q96QU1] |
| - | -0.06 | -0.63 | 0.26 | - | Hypothetical protein |
| - | -0.08 | -0.63 | -0.34 | - | Non-protein coding transcript |
| NP_872319.1 | 0.84 | -0.63 | 0.30 | NM_182513 | spindle pole body component 24 homolog [Source:RefSeq_peptide;Acc:NP_872319] |
| COL7A1 | 0.32 | -0.62 | 1.21 | NM_000094 | Collagen alpha 1(VII) chain precursor (Long-chain collagen) (LC collagen). [Source:Uniprot/SWISSPROT;Acc:Q02388] |
| NP_872338.1 | -0.24 | -0.62 | -0.30 | NM_182532 | - |
| UNKL | 0.17 | -0.62 | -0.48 | NM_024023 | unkempt-like [Source:RefSeq_peptide;Acc:NP_076928] |
| - | 0.41 | -0.62 | -0.11 | XM_040149 | - |
| - | -0.16 | -0.62 | -0.24 | XM_379438 | - |
| - | 0.33 | -0.62 | -0.03 | - | Non-protein coding transcript |
| GNAQ | 1.11 | -0.62 | -0.44 | NM_002072 | Guanine nucleotide-binding protein G(q), alpha subunit. [Source:Uniprot/SWISSPROT;Acc:P50148] |
| TAS1R2 | 0.00 | -0.62 | 0.60 | NM_152232 | Taste receptor type 1 member 2 precursor (G protein coupled receptor 71) (Sweet taste receptor T1R2). [Source:Uniprot/SWISSPROT;Acc:Q8TE23] |
| - | -0.20 | -0.62 | 0.56 | - | 43 kDa protein [Source:IPI;Acc:IPI00412436] |
| NP_689649.1 | 1.43 | -0.62 | 0.80 | NM_152436 | - |
| Q8IUV2_HUMAN | -0.30 | -0.62 | 0.64 | NM_198471 | - |
| TAOK2 | -0.36 | -0.62 | 0.39 | NM_016151 | TAO kinase 2 [Source:RefSeq_peptide;Acc:NP_004774] |
| XP_376725.1 | 0.28 | -0.62 | -0.37 | XM_379975,XM_376725 | PREDICTED: hypothetical protein XP_379975 [Source:RefSeq_peptide;Acc:XP_379975] |
| - | -0.32 | -0.62 | -0.06 | - | Non-protein coding transcript |
| REC16_HUMAN | 0.00 | -0.62 | -0.18 | - | HERV-K_10p14 provirus Rec protein. [Source:Uniprot/SWISSPROT;Acc:P61578] |
| HTRA4_HUMAN | 0.00 | -0.62 | -0.22 | NM_153692 | Probable serine protease HTRA4 precursor (EC 3.4.21.-). [Source:Uniprot/SWISSPROT;Acc:P83105] |
| NP_690000.1 | 0.75 | -0.62 | -0.41 | NM_198312,NM_152787 | TAK1-binding protein 3 isoform 1 [Source:RefSeq_peptide;Acc:NP_690000] |
| NP_689776.1 | -0.05 | -0.62 | 0.11 | NM_152563,NM_018172 | - |
| Q6RGF5_HUMAN | -0.31 | -0.62 | 0.03 | - | P40 (Fragment). [Source:Uniprot/SPTREMBL;Acc:Q6RGF5] |
| NP_849190.1 | 0.06 | -0.62 | -0.20 | NM_178859 | organic solute transporter beta [Source:RefSeq_peptide;Acc:NP_849190] |
| - | 0.00 | -0.62 | 0.38 | XM_498675 | - |
| - | -0.13 | -0.62 | 0.23 | XM_499000 | - |
| - | 0.28 | -0.62 | 0.28 | - | Non-protein coding transcript |
| RHCG | 0.00 | -0.62 | 0.89 | NM_016321 | Rhesus blood group, C glycoprotein [Source:RefSeq_peptide;Acc:NP_057405] |
| C9orf79 | -0.17 | -0.62 | 0.24 | - | - |
| C9orf68 | 1.77 | -0.62 | -0.19 | NM_017985 | - |
| UBP32_HUMAN | 0.25 | -0.62 | 0.25 | NM_032582 | Ubiquitin carboxyl-terminal hydrolase 32 (EC 3.1.2.15) (Ubiquitin thiolesterase 32) (Ubiquitin-specific processing protease 32) (Deubiquitinating enzyme 32) (NY-REN-60 antigen). [Source:Uniprot/SWISSPROT;Acc:Q8NFA0] |
| ALS4 | 0.29 | -0.62 | -0.11 | NM_015046 | Probable helicase senataxin (EC 3.6.1.-) (SEN1 homolog). [Source:Uniprot/SWISSPROT;Acc:Q7Z333] |
| - | 0.10 | -0.62 | 0.68 | - | Hypothetical protein |
| NP_006686.1 | 0.28 | -0.62 | 0.06 | NM_006695 | RaP2 interacting protein 8 [Source:RefSeq_peptide;Acc:NP_006686] |
| XP_375608.1 | 0.03 | -0.62 | 0.19 | XM_375608 | PREDICTED: similar to hypothetical protein [Source:RefSeq_peptide;Acc:XP_375608] |
| RAG1 | -0.04 | -0.62 | -0.32 | NM_000448 | V(D)J recombination activating protein 1 (RAG-1) (RING finger protein 74). [Source:Uniprot/SWISSPROT;Acc:P15918] |
| - | 0.18 | -0.62 | -0.11 | - | 35 kDa protein [Source:IPI;Acc:IPI00477670] |
| NP_057595.2 | 0.20 | -0.62 | 0.03 | NM_016511 | C-type lectin-like receptor-1 [Source:RefSeq_peptide;Acc:NP_057595] |
| - | 0.90 | -0.62 | -0.06 | - | 24 kDa protein [Source:IPI;Acc:IPI00413638] |
| - | -0.46 | -0.62 | -0.13 | XM_497592 | 41 kDa protein [Source:IPI;Acc:IPI00332378] |
| - | 0.21 | -0.62 | 0.31 | NR_001286 | - |
| - | 0.44 | -0.62 | -0.08 | XM_379543 | - |
| THEDC1 | 0.30 | -0.62 | 0.09 | NM_018324 | S-acyl fatty acid synthase thioesterase, medium chain (EC 3.1.2.14) (Thioesterase II) (Thioesterase domain containing protein 1). [Source:Uniprot/SWISSPROT;Acc:Q9NV23] |
| SOX14 | -0.02 | -0.62 | -0.05 | NM_004189 | Transcription factor SOX-14. [Source:Uniprot/SWISSPROT;Acc:O95416] |
| Q96TB2_HUMAN | 0.00 | -0.62 | 0.81 | - | Envelope protein (Fragment). [Source:Uniprot/SPTREMBL;Acc:Q96TB2] |
| C11orf17 | 0.00 | -0.62 | 6.21 | NM_182901,NM_020642 | Proline-rich protein BCA3 (Breast cancer associated gene 3 protein). [Source:Uniprot/SWISSPROT;Acc:Q9NQ31] |
| - | -0.04 | -0.62 | 0.00 | XM_499110 | - |
| CUL5 | 0.17 | -0.61 | -0.03 | NM_003478 | Cullin homolog 5 (CUL-5) (Vasopressin-activated calcium-mobilizing receptor) (VACM-1). [Source:Uniprot/SWISSPROT;Acc:Q93034] |
| NARG1_HUMAN | 0.21 | -0.61 | -0.24 | NM_057175 | NMDA receptor regulated protein 1 (N-terminal acetyltransferase) (Tubedown-1 protein) (Tbdn100) (Gastric cancer antigen Ga19). [Source:Uniprot/SWISSPROT;Acc:Q9BXJ9] |
| OR1N1 | 0.20 | -0.61 | 2.59 | NM_012363 | Olfactory receptor 1N1 (Olfactory receptor 1-26) (OR1-26) (Olfactory receptor 1N3). [Source:Uniprot/SWISSPROT;Acc:Q8NGS0] |
| ZFP1_HUMAN | 0.52 | -0.61 | 0.51 | NM_153688 | Zinc finger protein 1 homolog (Zfp-1). [Source:Uniprot/SWISSPROT;Acc:Q6P2D0] |
| RGS14 | 0.14 | -0.61 | -0.46 | NM_006480 | Regulator of G-protein signaling 14 (RGS14). [Source:Uniprot/SWISSPROT;Acc:O43566] |
| OR9G4 | 0.34 | -0.61 | -0.48 | NM_001005284 | Olfactory receptor 9G4. [Source:Uniprot/SWISSPROT;Acc:Q8NGQ1] |
| FAM46D | 0.99 | -0.61 | -0.22 | NM_152630 | family with sequence similarity 46, member D [Source:RefSeq_peptide;Acc:NP_689843] |
| NP_002007.1 | 0.62 | -0.61 | -0.27 | XM_048104 | filaggrin [Source:RefSeq_peptide;Acc:NP_002007] |
| CD36 | 0.36 | -0.61 | 0.35 | NM_001001548,NM_000072 | Platelet glycoprotein IV (GPIV) (Glycoprotein IIIb) (GPIIIB) (Leukocyte differentiation antigen CD36) (CD36 antigen) (PAS IV) (PAS- 4 protein) (Platelet collagen receptor) (Fatty acid translocase) (FAT) (Thrombospondin receptor). [Source:Uniprot/SWISSPROT |
| TMEM16A | 0.03 | -0.61 | -0.07 | - | transmembrane protein 16A [Source:RefSeq_peptide;Acc:NP_060513] |
| NYX | 0.48 | -0.61 | 0.24 | NM_022567 | Nyctalopin precursor. [Source:Uniprot/SWISSPROT;Acc:Q9GZU5] |
| - | 0.13 | -0.61 | -0.48 | NM_145809 | TL132 protein (LOC220594), mRNA [Source:RefSeq_dna;Acc:NM_145809] |
| NP_570900.1 | -0.50 | -0.61 | -0.02 | NM_130848 | dendritic cell nuclear protein 1 [Source:RefSeq_peptide;Acc:NP_570900] |
| GBX1 | 0.09 | -0.61 | 0.14 | XM_499494,XM_373219 | Homeobox protein GBX-1 (Gastrulation and brain-specific homeobox protein 1) (Fragment). [Source:Uniprot/SWISSPROT;Acc:Q14549] |
| - | 0.45 | -0.61 | -0.10 | NM_001010907 | - |
| - | -0.32 | -0.61 | -0.25 | XM_098350 | - |
| - | 1.85 | -0.61 | -0.14 | - | Hypothetical protein |
| VRK2_HUMAN | -0.49 | -0.61 | 0.41 | NM_006296 | Serine/threonine-protein kinase VRK2 (EC 2.7.1.37) (Vaccinia-related kinase 2). [Source:Uniprot/SWISSPROT;Acc:Q86Y07] |
| - | 0.71 | -0.61 | -0.42 | XM_029805 | - |
| - | 0.00 | -0.61 | -0.08 | XM_372614 | - |
| - | 2.06 | -0.61 | 0.14 | XM_373964 | - |
| - | 0.11 | -0.61 | -0.36 | - | Non-protein coding transcript |
| Q96LJ3_HUMAN | -0.23 | -0.61 | 0.28 | - | - |
| GABRG2 | -0.34 | -0.61 | -0.35 | NM_000816,NM_198904,NM_198903 | Gamma-aminobutyric-acid receptor gamma-2 subunit precursor (GABA(A) receptor). [Source:Uniprot/SWISSPROT;Acc:P18507] |
| CHCHD5 | 0.69 | -0.61 | 0.40 | NM_032309 | coiled-coil-helix-coiled-coil-helix domain containing 5 [Source:RefSeq_peptide;Acc:NP_115685] |
| RNASE2 | -0.16 | -0.61 | -0.06 | NM_002934 | Nonsecretory ribonuclease precursor (EC 3.1.27.5) (Ribonuclease US) (Eosinophil-derived neurotoxin) (RNase UpI-2) (Ribonuclease 2) (RNase 2). [Source:Uniprot/SWISSPROT;Acc:P10153] |
| NP_776168.1 | 1.31 | -0.61 | 0.30 | NM_173807 | - |
| TMEM41B | -0.11 | -0.61 | 0.33 | XM_495866 | - |
| C20orf7 | -0.42 | -0.61 | 0.23 | - | - |
| NXPH2 | 0.00 | -0.61 | -0.13 | NM_007226 | Neurexophilin-2 precursor. [Source:Uniprot/SWISSPROT;Acc:O95156] |
| OTX2 | 1.20 | -0.61 | -0.36 | NM_021728,NM_172337 | Homeobox protein OTX2. [Source:Uniprot/SWISSPROT;Acc:P32243] |
| Q8WZ91_HUMAN | -0.40 | -0.61 | -0.36 | - | PRbBP-39. [Source:Uniprot/SPTREMBL;Acc:Q8WZ91] |
| XP_372757.2 | 0.15 | -0.61 | -0.42 | XM_372757 | PREDICTED: similar to helix-loop-helix transcription factor [Source:RefSeq_peptide;Acc:XP_372757] |
| ITLN2 | -0.17 | -0.61 | 0.53 | NM_080878 | Intelectin 2 precursor (Endothelial lectin HL-2) (UNQ2789/PRO7179). [Source:Uniprot/SWISSPROT;Acc:Q8WWU7] |
| NP_859073.2 | -0.20 | -0.61 | 0.57 | NM_181722 | - |
| NP_940866.1 | 0.64 | -0.61 | -0.11 | NM_198464 | tryptophan/serine protease [Source:RefSeq_peptide;Acc:NP_940866] |
| IL22RA2 | -0.02 | -0.61 | -0.19 | NM_052962,NM_181309,NM_181310 | interleukin 22-binding protein isoform 1 [Source:RefSeq_peptide;Acc:NP_443194] |
| YG02_HUMAN | 0.69 | -0.61 | -0.04 | - | - |
| Q8N9W7_HUMAN | -0.24 | -0.61 | -0.18 | XM_496041,XM_208835,XM_496044,XM_375272 | - |
| - | -0.24 | -0.61 | -0.11 | XM_373675 | - |
| RNF39 | 0.90 | -0.61 | 0.13 | NM_025236,NM_170769 | HZFw1 protein isoform 2 [Source:RefSeq_peptide;Acc:NP_739575] |
| PP11_HUMAN | 0.35 | -0.61 | -0.06 | NM_006025 | Placental protein 11 precursor (EC 3.4.21.-) (PP11). [Source:Uniprot/SWISSPROT;Acc:P21128] |
| - | 0.10 | -0.61 | -0.11 | XM_495915 | - |
| RBM11 | 0.24 | -0.61 | 1.28 | NM_144770 | Putative RNA-binding protein 11 (RNA binding motif protein 11). [Source:Uniprot/SWISSPROT;Acc:P57052] |
| RCN3 | 0.32 | -0.61 | 0.15 | NM_020650 | Reticulocalbin 3 precursor (EF-hand calcium binding protein RLP49) (UNQ239/PRO272). [Source:Uniprot/SWISSPROT;Acc:Q96D15] |
| OR4F5 | 1.53 | -0.61 | 0.47 | NM_001005484 | Olfactory receptor 4F5. [Source:Uniprot/SWISSPROT;Acc:Q8NH21] |
| KRTAP8-1 | 1.00 | -0.61 | 0.70 | NM_175857 | Keratin-associated protein 8-1 (High glycine-tyrosine keratin- associated protein 8.1). [Source:Uniprot/SWISSPROT;Acc:Q8IUC2] |
| Q8N8B6_HUMAN | 1.20 | -0.61 | 1.34 | - | - |
| Q96M66_HUMAN | 1.12 | -0.61 | 0.34 | - | - |
| Q8N2E2_HUMAN | -0.18 | -0.61 | 0.20 | XM_371878,XM_374405 | - |
| ATP4A | 0.33 | -0.60 | 0.10 | NM_000704 | Potassium-transporting ATPase alpha chain 1 (EC 3.6.3.10) (Proton pump) (Gastric H+/K+ ATPase alpha subunit). [Source:Uniprot/SWISSPROT;Acc:P20648] |
| CRISP1 | 0.01 | -0.60 | -0.26 | NM_001131,NM_170609 | Cysteine-rich secretory protein-1 precursor (CRISP-1) (Acidic epididymal glycoprotein homolog) (AEG-like protein) (ARP). [Source:Uniprot/SWISSPROT;Acc:P54107] |
| ANP32C | -0.33 | -0.60 | -0.39 | NM_012403 | Acidic leucine-rich nuclear phosphoprotein 32 family member C (Tumorigenic protein pp32r1). [Source:Uniprot/SWISSPROT;Acc:O43423] |
| - | 0.17 | -0.60 | -0.37 | XM_498219,XM_499449 | - |
| - | -0.45 | -0.60 | -0.38 | - | Hypothetical protein |
| - | -0.32 | -0.60 | -0.06 | - | Non-protein coding transcript |
| SKI | 0.29 | -0.60 | 0.10 | NM_003036 | Ski oncogene (C-ski). [Source:Uniprot/SWISSPROT;Acc:P12755] |
| NP_076964.1 | -0.06 | -0.60 | 0.10 | NM_024059 | - |
| - | -0.10 | -0.60 | -0.32 | XM_496290 | - |
| - | -0.22 | -0.60 | 0.71 | - | TPR repeat containing protein |
| ADM | -0.38 | -0.60 | -0.49 | NM_001124 | ADM precursor [Contains: Adrenomedullin (AM); Proadrenomedullin N-20 terminal peptide (ProAM-N20) (ProAM N-terminal 20 peptide) (PAMP)]. [Source:Uniprot/SWISSPROT;Acc:P35318] |
| - | 0.00 | -0.60 | 0.10 | - | 35 kDa protein [Source:IPI;Acc:IPI00332981] |
| NP_477516.1 | 0.01 | -0.60 | 0.10 | NM_058168 | gene differentially expressed in prostate [Source:RefSeq_peptide;Acc:NP_477516] |
| FYB | 0.17 | -0.60 | -0.27 | NM_199335,NM_001465 | FYN-binding protein (FYN-T-binding protein) (FYB-120/130) (p120/p130) (SLP-76 associated phosphoprotein) (SLAP-130). [Source:Uniprot/SWISSPROT;Acc:O15117] |
| OPCML | -0.20 | -0.60 | 0.30 | NM_002545 | Opioid binding protein/cell adhesion molecule precursor (OBCAM) (Opioid-binding cell adhesion molecule) (OPCML). [Source:Uniprot/SWISSPROT;Acc:Q14982] |
| - | -0.11 | -0.60 | -0.24 | - | 75 kDa protein [Source:IPI;Acc:IPI00477323] |
| - | -0.17 | -0.60 | 0.21 | - | Hypothetical protein |
| ZS-rfp | -0.31 | -0.60 | -0.38 | - | Zoanthus sp. SAL-2001 red fluorescent protein |
| FKBP11 | -0.17 | -0.60 | -0.32 | NM_016594 | FK506 binding protein 11 precursor (EC 5.2.1.8) (Peptidyl-prolyl cis- trans isomerase) (PPIase) (Rotamase) (19 kDa FK506-binding protein) (FKBP-19) (UNQ336/PRO535). [Source:Uniprot/SWISSPROT;Acc:Q9NYL4] |
| SSH3 | -0.02 | -0.60 | 1.82 | NM_018276,NM_017857 | slingshot homolog 3 [Source:RefSeq_peptide;Acc:NP_060746] |
| IL1F8 | -0.11 | -0.60 | 0.06 | NM_014438,NM_173178 | Interleukin 1 family member 8 (IL-1F8) (Interleukin-1 eta) (IL-1 eta) (FIL1 eta) (Interleukin-1 homolog 2) (IL-1H2). [Source:Uniprot/SWISSPROT;Acc:Q9NZH7] |
| O52L2_HUMAN | -0.32 | -0.60 | -0.19 | - | Olfactory receptor 52L2. [Source:Uniprot/SWISSPROT;Acc:Q8NGH6] |
| SOCS4 | -0.03 | -0.60 | -0.25 | NM_199421,NM_080867 | Suppressor of cytokine signaling 4 (Suppressor of cytokine signaling 7) (SOCS-7). [Source:Uniprot/SWISSPROT;Acc:Q8WXH5] |
| - | 0.59 | -0.60 | -0.09 | NM_006295 | - |
| - | 0.51 | -0.60 | 0.12 | XM_376684,XM_379940 | - |
| - | 0.14 | -0.60 | -0.44 | XM_371026 | - |
| SLC10A3 | 0.66 | -0.60 | -0.05 | NM_019848 | P3 protein. [Source:Uniprot/SWISSPROT;Acc:P09131] |
| U3045_HUMAN | 0.57 | -0.60 | 0.90 | NM_207409 | Protein UNQ3045/PRO9861 precursor. [Source:Uniprot/SWISSPROT;Acc:Q6UWE3] |
| - | -0.41 | -0.60 | 0.25 | XM_376160 | 17 kDa protein [Source:IPI;Acc:IPI00413839] |
| - | 0.00 | -0.60 | 0.45 | - | 9 kDa protein [Source:IPI;Acc:IPI00514288] |
| - | 0.18 | -0.60 | 0.13 | - | Non-protein coding transcript |
| - | 0.96 | -0.60 | -0.39 | - | Hypothetical protein |
| LRRN6A | 0.28 | -0.60 | 0.20 | NM_032808 | leucine-rich repeat neuronal 6A [Source:RefSeq_peptide;Acc:NP_116197] |
| BNIPL_HUMAN | 1.89 | -0.60 | 0.14 | NM_138279 | Bcl-2/adenovirus E1B 19 kDa interacting protein 2 like protein. [Source:Uniprot/SWISSPROT;Acc:Q7Z465] |
| Q9Y4M2_HUMAN | 0.89 | -0.60 | -0.36 | - | - |
| AVPR1B | 1.06 | -0.60 | -0.30 | NM_000707 | Vasopressin V1b receptor (V1bR) (AVPR V1b) (Vasopressin V3 receptor) (AVPR V3) (Antidiuretic hormone receptor 1b). [Source:Uniprot/SWISSPROT;Acc:P47901] |
| XP_376386.1 | -0.26 | -0.60 | 0.49 | XM_376386 | PREDICTED: similar to DNA segment, Chr 13, Brigham & Womens Genetics 1146 expressed [Source:RefSeq_peptide;Acc:XP_376386] |
| - | 0.28 | -0.60 | -0.43 | NM_001010909 | - |
| CTSS | -0.36 | -0.60 | 1.25 | NM_004079 | Cathepsin S precursor (EC 3.4.22.27). [Source:Uniprot/SWISSPROT;Acc:P25774] |
| Q8N9G5_HUMAN | -0.41 | -0.60 | -0.47 | - | - |
| TSAR1_HUMAN | 0.22 | -0.60 | -0.46 | NM_139073 | Testis spermatocyte apoptosis-related gene 1 protein (Testis and spermatogenesis cell related protein 1). [Source:Uniprot/SWISSPROT;Acc:Q8NHX4] |
| - | 0.37 | -0.60 | 0.68 | - | Hypothetical protein |
| ESR1 | 1.01 | -0.60 | -0.26 | NM_000125 | Estrogen receptor (ER) (Estradiol receptor) (ER-alpha). [Source:Uniprot/SWISSPROT;Acc:P03372] |
| RORB | 0.08 | -0.60 | -0.46 | NM_006914 | Nuclear receptor ROR-beta (Nuclear receptor RZR-beta). [Source:Uniprot/SWISSPROT;Acc:Q92753] |
| UBQL2_HUMAN | -0.42 | -0.60 | -0.47 | NM_013444 | Ubiquilin 2 (Protein linking IAP with cytoskeleton-2) (PLIC-2) (hPLIC- 2) (Ubiquitin-like product Chap1/Dsk2) (DSK2 homolog) (Chap1) (HRIHFB2157). [Source:Uniprot/SWISSPROT;Acc:Q9UHD9] |
| C6orf199 | -0.09 | -0.60 | -0.01 | NM_145025 | - |
| NP_997198.1 | -0.23 | -0.60 | 0.02 | NM_207315 | - |
| - | 0.15 | -0.60 | -0.47 | NM_019003 | - |
| NP_859056.1 | -0.21 | -0.60 | -0.25 | NM_181705 | - |
| - | -0.42 | -0.60 | -0.45 | XM_499124 | - |
| XP_498454.1 | -0.24 | -0.59 | -0.26 | XM_498454,XM_499583 | PREDICTED: hypothetical protein XP_499583 [Source:RefSeq_peptide;Acc:XP_499583] |
| - | -0.28 | -0.59 | -0.39 | - | 43 kDa protein [Source:IPI;Acc:IPI00411477] |
| - | -0.29 | -0.59 | -0.09 | - | 38 kDa protein [Source:IPI;Acc:IPI00477286] |
| BCL2L10 | -0.44 | -0.59 | 0.49 | NM_020396 | Apoptosis regulator Bcl-B (Bcl-2-like 10 protein) (Anti-apoptotic protein NrH). [Source:Uniprot/SWISSPROT;Acc:Q9HD36] |
| BCAP29 | 0.04 | -0.59 | -0.02 | NM_018844,NM_001008405 | B-cell receptor-associated protein 29 (BCR-associated protein Bap29). [Source:Uniprot/SWISSPROT;Acc:Q9UHQ4] |
| SLCO1A2 | -0.05 | -0.59 | 0.15 | NM_005075,NM_021094,NM_134431 | Solute carrier organic anion transporter family, member 1A2 (Solute carrier family 21, member 3) (Sodium-independent organic anion transporter) (Organic anion transporting polypeptide 1) (OATP1) (OATP- A). [Source:Uniprot/SWISSPROT;Acc:P46721] |
| GNB1L | 0.45 | -0.59 | 0.24 | NM_024627 | Guanine nucleotide-binding protein beta subunit-like protein 1 (G protein beta-subunit like protein 1) (WD40 repeat containing protein deleted in VCFS) (WDVCF protein) (FKSG1 protein) (DGCRK3). [Source:Uniprot/SWISSPROT;Acc:Q9BYB4] |
| LECT1 | -0.32 | -0.59 | 0.30 | NM_007015 | Chondromodulin-I precursor (ChM-I) (Leukocyte cell-derived chemotaxin 1) [Contains: Chondrosurfactant protein (CH-SP)]. [Source:Uniprot/SWISSPROT;Acc:O75829] |
| XP_371759.2 | -0.02 | -0.59 | 0.05 | XM_371759 | - |
| NP_996563.1 | -0.11 | -0.59 | 0.38 | NM_206827 | RAS-like, family 11, member A [Source:RefSeq_peptide;Acc:NP_996563] |
| Q9P138_HUMAN | -0.24 | -0.59 | 0.60 | - | - |
| - | 0.27 | -0.59 | 0.72 | NM_001004339 | - |
| - | 0.14 | -0.59 | 0.37 | - | Non-protein coding transcript |
| CHRM2 | 0.55 | -0.59 | -0.42 | NM_001006631,NM_001006627,NM_001006629,NM_001006626,NM_001006630,NM_001006628,NM_000739,NM_001006632 | Muscarinic acetylcholine receptor M2. [Source:Uniprot/SWISSPROT;Acc:P08172] |
| SH3TC2 | 0.80 | -0.59 | 0.17 | NM_024577 | SH3 domain and tetratricopeptide repeats containing protein 2 (PP12494). [Source:Uniprot/SWISSPROT;Acc:Q8TF17] |
| RAPGEF5 | 0.04 | -0.59 | 0.16 | NM_012294 | Rap guanine nucleotide exchange factor 5 (Guanine nucleotide exchange factor for Rap1) (Related to Epac) (Repac) (M-Ras-regulated Rap GEF) (MR-GEF). [Source:Uniprot/SWISSPROT;Acc:Q92565] |
| - | -0.15 | -0.59 | 4.05 | XM_376207 | - |
| - | 0.23 | -0.59 | -0.14 | XM_498508 | - |
| NP_981947.1 | 1.33 | -0.59 | 0.41 | NM_203402 | similar to CG10671-like [Source:RefSeq_peptide;Acc:NP_981947] |
| Q8TBB3_HUMAN | 0.00 | -0.59 | 1.53 | - | - |
| NP_872392.1 | 0.38 | -0.59 | -0.05 | NM_182586 | - |
| PDE6A | -0.19 | -0.59 | 0.01 | NM_000440 | Rod cGMP-specific 3',5'-cyclic phosphodiesterase alpha-subunit (EC 3.1.4.17) (GMP-PDE alpha) (PDE V-B1). [Source:Uniprot/SWISSPROT;Acc:P16499] |
| - | 0.06 | -0.59 | -0.03 | XM_294249,XM_374600 | 111 kDa protein [Source:IPI;Acc:IPI00477287] |
| - | 0.30 | -0.59 | -0.47 | NM_194294 | - |
| ANKFY1 | -0.27 | -0.59 | 0.55 | NM_016376 | Ankyrin repeat and FYVE domain protein 1 (Ankyrin repeats hooked to a zinc finger motif). [Source:Uniprot/SWISSPROT;Acc:Q9P2R3] |
| NP_065816.1 | -0.20 | -0.59 | 0.30 | NM_020765 | retinoblastoma-associated factor 600 [Source:RefSeq_peptide;Acc:NP_065816] |
| - | 0.33 | -0.59 | -0.07 | XM_498573 | - |
| TNFSF8 | 0.41 | -0.59 | -0.43 | NM_001244 | Tumor necrosis factor ligand superfamily member 8 (CD30 ligand) (CD30- L) (CD153 antigen). [Source:Uniprot/SWISSPROT;Acc:P32971] |
| LHX9 | 0.31 | -0.59 | 0.71 | NM_020204 | LIM/homeobox protein Lhx9. [Source:Uniprot/SWISSPROT;Acc:Q9NQ69] |
| SPATA13 | 0.56 | -0.59 | -0.07 | - | - |
| TRPM2 | 0.34 | -0.59 | 0.21 | NM_003307,NM_001001188 | Transient receptor potential cation channel subfamily M member 2 (Long transient receptor potential channel 2) (LTrpC2) (Transient receptor potential channel 7) (TrpC7). [Source:Uniprot/SWISSPROT;Acc:O94759] |
| - | 0.96 | -0.59 | -0.50 | - | Hypothetical protein |
| NP_612367.1 | 1.12 | -0.59 | 0.05 | NM_138358 | - |
| SLC1A5 | 0.32 | -0.59 | -0.45 | NM_005628 | Neutral amino acid transporter B(0) (ATB(0)) (Sodium-dependent neutral amino acid transporter type 2) (RD114/simian type D retrovirus receptor) (Baboon M7 virus receptor). [Source:Uniprot/SWISSPROT;Acc:Q15758] |
| KRTAP4-5 | -0.34 | -0.59 | -0.10 | NM_033188,NM_031854 | Keratin-associated protein 4-5 (Keratin-associated protein 4.5) (Ultrahigh sulfur keratin-associated protein 4.5). [Source:Uniprot/SWISSPROT;Acc:Q9BYR2] |
| - | -0.06 | -0.59 | 0.35 | - | - |
| LIMS3 | -0.05 | -0.59 | -0.39 | NM_033514 | LIM and senescent cell antigen-like domains 3 [Source:RefSeq_peptide;Acc:NP_277049] |
| - | 0.05 | -0.59 | -0.03 | XM_497521 | - |
| - | 0.07 | -0.59 | -0.21 | - | Non-protein coding transcript |
| NP_689533.1 | 0.06 | -0.59 | -0.41 | NM_152320 | - |
| XP_495888.1 | 0.09 | -0.59 | -0.31 | XM_495888 | PREDICTED: similar to hypothetical protein [Source:RefSeq_peptide;Acc:XP_495888] |
| ZNF442 | 0.53 | -0.59 | -0.03 | NM_030824 | Zinc finger protein 442. [Source:Uniprot/SWISSPROT;Acc:Q9H7R0] |
| - | 0.29 | -0.59 | 0.54 | NM_194324 | - |
| NP_115981.1 | 0.42 | -0.59 | 0.94 | NM_032592 | 1-aminocyclopropane-1-carboxylate synthase [Source:RefSeq_peptide;Acc:NP_115981] |
| SLC43A3 | 0.43 | -0.59 | -0.39 | NM_014096,NM_017611,NM_199329 | solute carrier family 43, member 3 [Source:RefSeq_peptide;Acc:NP_054815] |
| - | 0.43 | -0.59 | -0.39 | - | 51 kDa protein [Source:IPI;Acc:IPI00477453] |
| NP_853651.1 | 1.39 | -0.59 | -0.37 | NM_181620 | keratin associated protein 22-1 [Source:RefSeq_peptide;Acc:NP_853651] |
| CATR1 | 1.24 | -0.59 | 0.01 | - | CATR tumorigenic conversion 1 protein (CATR1.3). [Source:Uniprot/SWISSPROT;Acc:Q13166] |
| ASPA | -0.35 | -0.58 | 0.37 | NM_000049 | Aspartoacylase (EC 3.5.1.15) (Aminoacylase-2) (ACY-2). [Source:Uniprot/SWISSPROT;Acc:P45381] |
| - | 0.14 | -0.58 | 0.17 | XM_498601 | - |
| ZNF286 | -0.37 | -0.58 | -0.14 | - | Zinc finger protein 286. [Source:Uniprot/SWISSPROT;Acc:Q9HBT8] |
| NM_024107.1 | -0.09 | -0.58 | -0.48 | - | - |
| - | 0.55 | -0.58 | -0.10 | - | Hypothetical protein |
| - | -0.04 | -0.58 | -0.14 | - | Hypothetical protein |
| - | 1.01 | -0.58 | 1.34 | - | Hypothetical protein |
| - | 1.54 | -0.58 | 1.08 | - | Conserved hypothetical protein |
| PCDHB18 | 0.35 | -0.58 | 0.21 | NR_001281 | protocadherin beta 18 pseudogene (PCDHB18) on chromosome 5 [Source:RefSeq_dna;Acc:NR_001281] |
| NP_477521.1 | 0.17 | -0.58 | 0.44 | NM_058173 | small breast epithelial mucin [Source:RefSeq_peptide;Acc:NP_477521] |
| HNRPR | 0.01 | -0.58 | -0.45 | NM_005826 | Heterogeneous nuclear ribonucleoprotein R (hnRNP R). [Source:Uniprot/SWISSPROT;Acc:O43390] |
| MCOLN2 | 0.00 | -0.58 | 0.40 | NM_153259 | Mucolipin-2. [Source:Uniprot/SWISSPROT;Acc:Q8IZK6] |
| - | -0.47 | -0.58 | 0.27 | - | Hypothetical protein |
| AMBP | 0.42 | -0.58 | -0.34 | NM_001633 | AMBP protein precursor [Contains: Alpha-1-microglobulin (Protein HC) (Complex-forming glycoprotein heterogeneous in charge) (Alpha-1 microglycoprotein); Inter-alpha-trypsin inhibitor light chain (ITI-LC) (Bikunin) (HI-30)]. [Source:Uniprot/SWISSPROT;Acc:P |
| OR4N2 | 0.65 | -0.58 | 0.57 | NM_001004723 | Olfactory receptor 4N2. [Source:Uniprot/SWISSPROT;Acc:Q8NGD1] |
| Q7Z470_HUMAN | 0.69 | -0.58 | -0.03 | NM_194285 | - |
| - | 0.27 | -0.58 | -0.17 | XM_497981 | 28 kDa protein [Source:IPI;Acc:IPI00412379] |
| - | -0.10 | -0.58 | -0.05 | NM_000506 | - |
| - | -0.14 | -0.58 | 0.67 | - | Non-protein coding transcript |
| ADD2 | 0.21 | -0.58 | 1.39 | NM_017488,NM_001617,NM_017486,NM_017483,NM_017485 | Beta adducin (Erythrocyte adducin beta subunit). [Source:Uniprot/SWISSPROT;Acc:P35612] |
| WBSCR5 | -0.19 | -0.58 | -0.15 | NM_032463,NM_022040,NM_014146,NM_032464 | WBSCR5 protein isoform 1 [Source:RefSeq_peptide;Acc:NP_071323] |
| NP_115811.1 | 0.07 | -0.58 | -0.10 | NM_032435 | mixed lineage kinase 4 [Source:RefSeq_peptide;Acc:NP_115811] |
| - | -0.03 | -0.58 | -0.30 | NM_001004736 | - |
| MX2 | 0.37 | -0.58 | 0.73 | NM_002463 | Interferon-regulated resistance GTP-binding protein MxB (p78-related protein). [Source:Uniprot/SWISSPROT;Acc:P20592] |
| - | -0.12 | -0.58 | 0.12 | XM_498042 | - |
| - | -0.13 | -0.58 | 0.18 | - | Hypothetical protein |
| CTDP1 | -0.27 | -0.58 | -0.26 | NM_004715,NM_048368 | RNA polymerase II subunit A C-terminal domain phosphatase (EC 3.1.3.16) (TFIIF-associating CTD phosphatase). [Source:Uniprot/SWISSPROT;Acc:Q9Y5B0] |
| UTX | -0.04 | -0.58 | 0.74 | NM_021140 | Ubiquitously transcribed X chromosome tetratricopeptide repeat protein (Ubiquitously transcribed TPR protein on the X chromosome). [Source:Uniprot/SWISSPROT;Acc:O15550] |
| - | 0.74 | -0.58 | 0.04 | - | Hypothetical protein |
| RHOH | 0.17 | -0.58 | 0.53 | NM_004310 | Rho-related GTP-binding protein RhoH (GTP-binding protein TTF). [Source:Uniprot/SWISSPROT;Acc:Q15669] |
| Q9H6A0_HUMAN | 0.20 | -0.58 | -0.34 | - | - |
| - | 1.56 | -0.58 | 0.15 | XM_374088 | - |
| - | -0.08 | -0.58 | -0.47 | XM_378964 | - |
| PLA2G10 | 0.52 | -0.58 | -0.32 | NM_003561,XM_497517,XM_497487,XM_497486 | Group X secretory phospholipase A2 precursor (EC 3.1.1.4) (Phosphatidylcholine 2-acylhydrolase GX) (GX sPLA2) (sPLA2-X). [Source:Uniprot/SWISSPROT;Acc:O15496] |
| UMOD | 1.69 | -0.58 | 0.05 | NM_003361,NM_001008389 | Uromodulin precursor (Tamm-Horsfall urinary glycoprotein) (THP). [Source:Uniprot/SWISSPROT;Acc:P07911] |
| SFRP5 | 0.43 | -0.58 | 0.09 | NM_003015 | secreted frizzled-related protein 5 [Source:RefSeq_peptide;Acc:NP_003006] |
| Q9P171_HUMAN | -0.07 | -0.58 | -0.18 | - | - |
| - | 0.20 | -0.58 | -0.27 | - | 13 kDa protein [Source:IPI;Acc:IPI00455619] |
| NP_115557.1 | 0.62 | -0.58 | 0.30 | NM_032181 | - |
| RELB | 0.66 | -0.58 | 0.20 | NM_006509 | Transcription factor RelB (I-Rel). [Source:Uniprot/SWISSPROT;Acc:Q01201] |
| - | 0.32 | -0.58 | -0.22 | XM_372273 | - |
| - | -0.17 | -0.58 | -0.13 | - | Hypothetical protein |
| - | -0.21 | -0.58 | -0.38 | - | Homo sapiens microRNA miR-108 stem-loop |
| - | 0.03 | -0.58 | -0.41 | - | Similar to Cytochrome c oxidase, subunit CcoO |
| F11 | -0.07 | -0.58 | -0.07 | NM_019559,NM_000128 | Coagulation factor XI precursor (EC 3.4.21.27) (Plasma thromboplastin antecedent) (PTA) (FXI). [Source:Uniprot/SWISSPROT;Acc:P03951] |
| RNF157 | -0.10 | -0.58 | 0.30 | NM_052916 | ring finger protein 157 [Source:RefSeq_peptide;Acc:NP_443148] |
| - | -0.06 | -0.58 | -0.50 | XM_497067 | - |
| SPG4 | -0.46 | -0.57 | -0.02 | NM_014946,NM_199436 | Spastin. [Source:Uniprot/SWISSPROT;Acc:Q9UBP0] |
| C14orf155 | 0.00 | -0.57 | -0.16 | NM_032135 | - |
| C9orf90 | -0.01 | -0.57 | 0.54 | NM_197956 | - |
| - | 0.26 | -0.57 | 0.80 | NM_006122 | - |
| - | -0.28 | -0.57 | 1.62 | NM_033334 | - |
| - | -0.14 | -0.57 | -0.21 | XM_084868 | - |
| KCNJ3 | 0.74 | -0.57 | 0.04 | NM_002239 | G protein-activated inward rectifier potassium channel 1 (GIRK1) (Potassium channel, inwardly rectifying, subfamily J, member 3) (Inward rectifier K(+) channel Kir3.1). [Source:Uniprot/SWISSPROT;Acc:P48549] |
| Q8N822_HUMAN | 0.60 | -0.57 | 0.02 | - | - |
| - | -0.35 | -0.57 | 0.02 | NM_020805 | kelch-like 14 (Drosophila) (KLHL14), mRNA [Source:RefSeq_dna;Acc:NM_020805] |
| - | 0.61 | -0.57 | 0.24 | XM_497865 | - |
| - | 0.09 | -0.57 | -0.20 | - | Human endogenous retrovirus HCML-ARV, complete genome |
| - | 0.57 | -0.57 | 0.51 | XM_498548 | - |
| - | 0.71 | -0.57 | 0.29 | - | Hypothetical protein |
| GNAT2 | 0.14 | -0.57 | -0.36 | NM_005272 | Guanine nucleotide-binding protein G(t), alpha-2 subunit (Transducin alpha-2 chain). [Source:Uniprot/SWISSPROT;Acc:P19087] |
| TCF23 | 0.02 | -0.57 | -0.23 | - | Class II basic helix-loop-helix protein TCF23. [Source:Uniprot/SPTREMBL;Acc:Q7RTU1] |
| - | 0.02 | -0.57 | 0.03 | - | Transcribed locus, moderately similar to XP_499076.1 LOC441269 [Homo sapiens] [Source:UniGene;Acc:Hs.545701] |
| NP_612442.1 | 0.38 | -0.57 | -0.23 | NM_138433 | - |
| USP53 | -0.04 | -0.57 | 0.56 | - | ubiquitin specific protease 53 [Source:RefSeq_peptide;Acc:NP_061923] |
| - | -0.23 | -0.57 | 0.86 | - | 47 kDa protein [Source:IPI;Acc:IPI00376054] |
| ZNF543 | 0.58 | -0.57 | 0.41 | NM_213598,XM_372753 | zinc finger protein 543 [Source:RefSeq_peptide;Acc:NP_998763] |
| - | 0.88 | -0.57 | -0.10 | - | Conserved hypothetical protein |
| MBNL3 | -0.49 | -0.57 | -0.24 | NM_018388 | Muscleblind-like X-linked protein (Muscleblind-like protein 3) (Cys3His CCG1-required protein) (HCHCR protein). [Source:Uniprot/SWISSPROT;Acc:Q9NUK0] |
| HEM1 | -0.01 | -0.57 | 1.68 | NM_005337 | Membrane-associated protein HEM-1 (Hematopoietic protein 1). [Source:Uniprot/SWISSPROT;Acc:P55160] |
| - | 0.25 | -0.57 | 0.06 | NM_032626 | - |
| - | 0.61 | -0.57 | -0.11 | NM_002248 | - |
| - | -0.23 | -0.57 | -0.40 | XM_498165 | - |
| - | 0.15 | -0.57 | -0.21 | XM_498976 | - |
| - | 0.34 | -0.57 | 0.73 | - | Non-protein coding transcript |
| UB2D2_HUMAN | -0.09 | -0.57 | -0.08 | NM_181838,NM_003339 | Ubiquitin-conjugating enzyme E2 D2 (EC 6.3.2.19) (Ubiquitin-protein ligase D2) (Ubiquitin carrier protein D2) (Ubiquitin-conjugating enzyme E2-17 kDa 2) (E2(17)KB 2). [Source:Uniprot/SWISSPROT;Acc:P62837] |
| - | -0.38 | -0.57 | -0.14 | XM_499248,XM_496671,XM_496250,XM_496842 | - |
| sacB | -0.42 | -0.57 | -0.27 | - | Levansucrase |
| HTR1B | 0.69 | -0.57 | 0.62 | NM_000863 | 5-hydroxytryptamine 1B receptor (5-HT-1B) (Serotonin receptor 1B) (5- HT-1D-beta) (Serotonin 1D beta receptor) (S12). [Source:Uniprot/SWISSPROT;Acc:P28222] |
| DSCR1L2 | 1.30 | -0.57 | 0.42 | NM_013441 | Calcipressin 3 (Down syndrome candidate region 1-like protein 2) (Myocyte-enriched calcineurin interacting protein 3) (MCIP3). [Source:Uniprot/SWISSPROT;Acc:Q9UKA8] |
| RNF149 | 0.09 | -0.57 | 0.40 | NM_173647 | ring finger protein 149 [Source:RefSeq_peptide;Acc:NP_775918] |
| - | 0.26 | -0.57 | -0.22 | XM_290629,XM_290629,XM_290629,XM_290629,XM_290629,XM_290629,XM_290629 | 98 kDa protein [Source:IPI;Acc:IPI00477829] |
| - | 0.18 | -0.57 | 0.54 | XM_497349 | - |
| - | 0.06 | -0.57 | -0.03 | XM_499059,XM_499508 | - |
| - | 0.61 | -0.57 | 1.13 | - | Non-protein coding transcript |
| SLA | 0.64 | -0.57 | 0.00 | - | SRC-like-adapter (Src-like-adapter protein 1) (hSLAP). [Source:Uniprot/SWISSPROT;Acc:Q13239] |
| ZNF589 | 0.75 | -0.57 | 0.96 | NM_016089 | zinc finger protein 589 [Source:RefSeq_peptide;Acc:NP_057173] |
| - | -0.02 | -0.57 | 0.92 | XM_378901 | - |
| CHRDL2 | -0.11 | -0.57 | 0.27 | NM_015424 | chordin-like 2 [Source:RefSeq_peptide;Acc:NP_056239] |
| Q86TU0_HUMAN | 0.10 | -0.57 | -0.27 | NM_032630 | cyclin-dependent kinase 2-interacting protein [Source:RefSeq_peptide;Acc:NP_116019] |
| RASSF6 | -0.37 | -0.57 | 0.36 | NM_177532,NM_201431 | Ras association (RalGDS/AF-6) domain family 6 isoform a [Source:RefSeq_peptide;Acc:NP_803876] |
| RASSF3 | 0.95 | -0.57 | -0.40 | NM_178169 | Ras association (RalGDS/AF-6) domain family 3 [Source:RefSeq_peptide;Acc:NP_835463] |
| FILIP1 | 0.19 | -0.57 | 0.81 | NM_015687 | filamin A interacting protein 1 [Source:RefSeq_peptide;Acc:NP_056502] |
| - | 0.00 | -0.57 | 0.55 | NM_004309 | - |
| - | 0.38 | -0.57 | -0.29 | XM_498739 | - |
| - | 0.88 | -0.57 | -0.38 | - | Non-protein coding transcript |
| LYST_HUMAN | 0.01 | -0.57 | -0.37 | NM_000081 | Lysosomal trafficking regulator (Beige homolog). [Source:Uniprot/SWISSPROT;Acc:Q99698] |
| - | 0.56 | -0.57 | -0.21 | - | Non-protein coding transcript |
| - | 0.53 | -0.56 | 0.20 | - | Non-protein coding transcript |
| PIK3R1 | -0.43 | -0.56 | -0.50 | NM_181524,NM_181523,NM_181504 | Phosphatidylinositol 3-kinase regulatory alpha subunit (PI3-kinase p85-alpha subunit) (PtdIns-3-kinase p85-alpha) (PI3K). [Source:Uniprot/SWISSPROT;Acc:P27986] |
| RASIP1 | -0.27 | -0.56 | -0.44 | NM_017805 | Ras-interacting protein 1 [Source:RefSeq_peptide;Acc:NP_060275] |
| FEV | 0.14 | -0.56 | -0.38 | NM_017521 | FEV (ETS oncogene family) [Source:RefSeq_peptide;Acc:NP_059991] |
| OR5J2 | -0.26 | -0.56 | -0.41 | NM_001005492 | Olfactory receptor 5J2. [Source:Uniprot/SWISSPROT;Acc:Q8NH18] |
| CLDN8 | -0.37 | -0.56 | 0.02 | NM_199328 | Claudin-8 (UNQ779/PRO1573). [Source:Uniprot/SWISSPROT;Acc:P56748] |
| FAM55A | 0.87 | -0.56 | -0.46 | NM_152315 | family with sequence similarity 55, member A [Source:RefSeq_peptide;Acc:NP_689528] |
| LCP2 | 0.33 | -0.56 | 0.20 | NM_005565 | Lymphocyte cytosolic protein 2 (SH2 domain-containing leucocyte protein of 76 kDa) (SLP-76 tyrosine phosphoprotein) (SLP76). [Source:Uniprot/SWISSPROT;Acc:Q13094] |
| - | -0.28 | -0.56 | -0.44 | XM_166090 | - |
| - | 0.47 | -0.56 | -0.03 | XM_166747 | - |
| - | 0.88 | -0.56 | 0.33 | XM_378487 | - |
| - | 0.42 | -0.56 | 1.06 | XM_498628 | - |
| - | -0.05 | -0.56 | 0.41 | NM_012219 | - |
| Q9UHT5_HUMAN | 0.15 | -0.56 | -0.49 | - | - |
| DSG1 | -0.42 | -0.56 | 0.11 | NM_001942 | Desmoglein-1 precursor (Desmosomal glycoprotein 1) (DG1) (DGI) (Pemphigus foliaceus antigen). [Source:Uniprot/SWISSPROT;Acc:Q02413] |
| FNTB | 0.11 | -0.56 | 0.04 | NM_002028 | Protein farnesyltransferase beta subunit (EC 2.5.1.58) (CAAX farnesyltransferase beta subunit) (RAS proteins prenyltransferase beta) (FTase-beta). [Source:Uniprot/SWISSPROT;Acc:P49356] |
| - | 0.87 | -0.56 | -0.05 | XM_497358 | - |
| - | -0.24 | -0.56 | -0.42 | XM_498941 | - |
| - | -0.06 | -0.56 | -0.32 | - | Hypothetical protein |
| IL15 | 0.39 | -0.56 | -0.36 | NM_172175 | Interleukin-15 precursor (IL-15). [Source:Uniprot/SWISSPROT;Acc:P40933] |
| OR7A2_HUMAN | 0.22 | -0.56 | -0.28 | - | Olfactory receptor 7A2. [Source:Uniprot/SWISSPROT;Acc:Q8NGA2] |
| LV0A_HUMAN | 0.27 | -0.56 | -0.17 | - | Ig lambda chain V region 4A precursor. [Source:Uniprot/SWISSPROT;Acc:P04211] |
| - | 0.18 | -0.56 | -0.21 | XM_067076 | - |
| - | 0.34 | -0.56 | 0.09 | XM_373876 | - |
| XP_496494.1 | -0.39 | -0.56 | -0.34 | XM_496498,XM_496503,XM_496494 | PREDICTED: similar to hypothetical protein similar to gamma-glutamyltransferase-like activity 1 [Source:RefSeq_peptide;Acc:XP_496503] |
| RAB27A | 0.00 | -0.56 | -0.46 | NM_004580,NM_183235,NM_183234,NM_183236 | Ras-related protein Rab-27A (Rab-27) (GTP-binding protein Ram). [Source:Uniprot/SWISSPROT;Acc:P51159] |
| NP_060512.2 | 0.74 | -0.56 | 0.10 | NM_018042 | - |
| XP_496072.1 | -0.17 | -0.56 | 0.27 | XM_496072 | PREDICTED: RNA-binding protein with multiple splicing 2 [Source:RefSeq_peptide;Acc:XP_496072] |
| FAM9C | 0.40 | -0.56 | 0.18 | NM_174901 | Protein FAM9C. [Source:Uniprot/SWISSPROT;Acc:Q8IZT9] |
| - | -0.13 | -0.56 | -0.25 | XM_379976,XM_165511 | - |
| - | 0.83 | -0.56 | 0.28 | - | Hypothetical protein |
| - | 0.19 | -0.56 | -0.47 | NM_033512 | - |
| CHRDL1 | -0.05 | -0.56 | -0.31 | NM_145234 | Chordin-like protein 1 precursor (Neuralin) (Ventroptin). [Source:Uniprot/SWISSPROT;Acc:Q9BU40] |
| MN1 | -0.48 | -0.56 | 0.12 | NM_002430 | Probable tumor suppressor protein MN1. [Source:Uniprot/SWISSPROT;Acc:Q10571] |
| NP_689501.1 | 0.12 | -0.56 | -0.47 | NM_152288 | - |
| USP35 | -0.20 | -0.56 | 0.90 | XM_290527 | Ubiquitin carboxyl-terminal hydrolase 35 (EC 3.1.2.15) (Ubiquitin thiolesterase 35) (Ubiquitin-specific processing protease 35) (Deubiquitinating enzyme 35). [Source:Uniprot/SWISSPROT;Acc:Q9P2H5] |
| OSBPL5 | 0.61 | -0.56 | 0.04 | NM_145638,NM_020896 | Oxysterol binding protein-related protein 5 (OSBP-related protein 5) (ORP-5). [Source:Uniprot/SWISSPROT;Acc:Q9H0X9] |
| ZNF537 | 0.65 | -0.56 | 0.55 | NM_020856 | zinc finger protein 537 [Source:RefSeq_peptide;Acc:NP_065907] |
| - | 0.62 | -0.56 | 0.70 | XM_374339,XM_374338 | - |
| Q9HAA9_HUMAN | 0.32 | -0.56 | 0.16 | - | CMP-N-acetylneuraminate-beta-galactosamide-alpha-2,3-sialyltransferase (EC 2.4.99.-) (Beta-galactoside alpha-2,3-sialyltransferase) (Alpha 2,3-sialyltransferase IV) (Alpha 2,3-ST) (Gal-NAc6S) (STZ) (SIAT4-C) (ST3Gal III) (SAT-3) (ST-4). [Source:Uniprot/SW |
| CAMK4 | -0.50 | -0.56 | -0.48 | NM_001744 | Calcium/calmodulin-dependent protein kinase type IV (EC 2.7.1.123) (CAM kinase-GR) (CaMK IV). [Source:Uniprot/SWISSPROT;Acc:Q16566] |
| NP_060374.1 | 0.04 | -0.56 | 0.48 | NM_017904 | - |
| - | 1.08 | -0.56 | 0.00 | NM_022140 | - |
| - | 1.48 | -0.56 | -0.17 | XM_498808,XM_498805 | - |
| - | -0.13 | -0.56 | -0.50 | - | Hypothetical protein |
| ROBO2 | -0.26 | -0.56 | -0.10 | XM_031246 | Roundabout homolog 2 precursor. [Source:Uniprot/SWISSPROT;Acc:Q9HCK4] |
| - | 0.07 | -0.56 | -0.31 | XM_378985 | - |
| - | -0.05 | -0.56 | 0.31 | XM_497019 | - |
| - | -0.13 | -0.56 | -0.07 | XM_499450,XM_498220 | - |
| - | 0.09 | -0.56 | 0.08 | - | Homo sapiens microRNA miR-200c stem-loop |
| NP_699185.1 | -0.22 | -0.56 | 0.43 | NM_153354 | - |
| Q9H3B5_HUMAN | -0.08 | -0.56 | -0.24 | - | - |
| NETO1 | 0.16 | -0.55 | 0.09 | NM_138966 | Neuropilin and tolloid like-1 precursor (Brain-specific transmembrane protein containing 2 CUB and 1 LDL-receptor class A domains protein 1). [Source:Uniprot/SWISSPROT;Acc:Q8TDF5] |
| CYorf14 | 0.21 | -0.55 | 0.33 | NR_001544 | - |
| PSORS1C2 | 1.04 | -0.55 | 1.34 | NM_014069 | Psoriasis susceptibility 1 candidate gene 2 protein precursor (SPR1 protein). [Source:Uniprot/SWISSPROT;Acc:Q9UIG4] |
| NP_777586.1 | 1.25 | -0.55 | -0.43 | NM_174926 | - |
| XP_170909.4 | 0.31 | -0.55 | -0.21 | XM_170909 | PREDICTED: similar to RIKEN cDNA 1700009P17 [Source:RefSeq_peptide;Acc:XP_170909] |
| - | 0.02 | -0.55 | 1.24 | XM_497783 | - |
| MINA | 1.73 | -0.55 | -0.09 | NM_032778,NM_153182 | MYC induced nuclear antigen isoform 3 [Source:RefSeq_peptide;Acc:NP_694822] |
| ABCA7 | -0.04 | -0.55 | 0.80 | NM_033308,NM_019112 | ATP-binding cassette, sub-family A, member 7 isoform b [Source:RefSeq_peptide;Acc:NP_150651] |
| Q15401_HUMAN | -0.21 | -0.55 | -0.29 | - | Line-1 repeat mRNA with 2 open reading frames. [Source:Uniprot/SPTREMBL;Acc:Q15401] |
| - | 0.24 | -0.55 | -0.33 | - | 36 kDa protein [Source:IPI;Acc:IPI00104496] |
| NP_982279.1 | 0.61 | -0.55 | -0.34 | NM_203454 | - |
| - | 0.10 | -0.55 | -0.49 | XM_498259 | - |
| - | -0.17 | -0.55 | -0.46 | XM_376419 | - |
| Q8N7N0_HUMAN | 0.33 | -0.55 | -0.17 | - | - |
| MLPH | -0.27 | -0.55 | 0.15 | NM_024101 | Melanophilin (Exophilin 3) (Synaptotagmin-like protein 2a) (Slp homologue lacking C2 domains-a). [Source:Uniprot/SWISSPROT;Acc:Q9BV36] |
| - | 0.86 | -0.55 | -0.04 | XM_496360 | - |
| - | -0.08 | -0.55 | -0.33 | XM_209640 | - |
| Q6NS41_HUMAN | -0.10 | -0.55 | 0.76 | XM_498262,NM_002735 | - |
| - | 0.11 | -0.55 | -0.14 | XM_498916 | - |
| UCP1 | 0.01 | -0.55 | -0.31 | NM_021833 | Mitochondrial brown fat uncoupling protein 1 (UCP 1) (Thermogenin). [Source:Uniprot/SWISSPROT;Acc:P25874] |
| Q96GE5_HUMAN | 0.67 | -0.55 | -0.23 | NM_005815 | HIT-40 protein (Fragment). [Source:Uniprot/SPTREMBL;Acc:Q96GE5] |
| - | 0.33 | -0.55 | -0.14 | XM_498942 | - |
| NP_001011880.1 | -0.49 | -0.55 | -0.37 | - | secretory protein LOC348174 [Source:RefSeq_peptide;Acc:NP_872425] |
| Q96S09_HUMAN | -0.16 | -0.55 | 0.06 | - | - |
| SHB | -0.05 | -0.55 | 0.19 | - | SHB (Src homology 2 domain containing) adaptor protein B [Source:RefSeq_peptide;Acc:NP_003019] |
| NP_872313.1 | 0.32 | -0.55 | -0.33 | NM_182507 | - |
| - | 0.29 | -0.55 | 0.27 | XM_497459 | - |
| NP_110429.1 | 0.32 | -0.55 | -0.38 | NM_030802 | C/EBP-induced protein [Source:RefSeq_peptide;Acc:NP_110429] |
| RECQL4 | -0.22 | -0.55 | 1.24 | NM_004260 | ATP-dependent DNA helicase Q4 (RecQ protein-like 4) (RecQ4). [Source:Uniprot/SWISSPROT;Acc:O94761] |
| CALR3 | 0.02 | -0.55 | -0.29 | NM_145046 | Calreticulin 3 precursor (Calreticulin 2). [Source:Uniprot/SWISSPROT;Acc:Q96L12] |
| TLR2 | 0.23 | -0.55 | 1.22 | NM_003264 | Toll-like receptor 2 precursor (Toll/interleukin 1 receptor-like protein 4). [Source:Uniprot/SWISSPROT;Acc:O60603] |
| - | 1.27 | -0.55 | -0.26 | - | Hypothetical protein |
| - | -0.13 | -0.55 | -0.35 | - | Hypothetical protein |
| - | 0.22 | -0.55 | -0.26 | - | Non-protein coding transcript |
| C1orf24 | 0.20 | -0.55 | 0.75 | NM_052966 | Niban protein. [Source:Uniprot/SWISSPROT;Acc:Q9BZQ8] |
| EAF1 | 0.16 | -0.55 | -0.13 | NM_033083 | ELL associated factor 1 [Source:RefSeq_peptide;Acc:NP_149074] |
| - | 0.14 | -0.55 | -0.06 | NM_145310 | - |
| NP_060880.2 | -0.48 | -0.55 | 0.06 | NM_018410 | - |
| Q9H3C5_HUMAN | 1.30 | -0.55 | 0.45 | - | - |
| - | -0.31 | -0.55 | -0.21 | - | 115 kDa protein [Source:IPI;Acc:IPI00479762] |
| - | -0.43 | -0.55 | -0.31 | - | Hypothetical protein |
| - | -0.32 | -0.55 | 0.06 | - | Hypothetical protein |
| ZNF235 | 0.22 | -0.55 | -0.41 | NM_004234 | Zinc finger protein 235 (Zinc finger protein 93 homolog) (Zfp-93) (Zinc finger protein HZF6) (Fragment). [Source:Uniprot/SWISSPROT;Acc:Q14590] |
| GPR107 | 0.08 | -0.55 | -0.47 | - | G protein-coupled receptor 107 [Source:RefSeq_peptide;Acc:NP_066011] |
| Q8NGP1_HUMAN | 0.28 | -0.55 | 0.03 | - | Seven transmembrane helix receptor. [Source:Uniprot/SPTREMBL;Acc:Q8NGP1] |
| MAGEA11 | 0.12 | -0.55 | 2.64 | NM_005366 | Melanoma-associated antigen 11 (MAGE-11 antigen). [Source:Uniprot/SWISSPROT;Acc:P43364] |
| - | -0.50 | -0.55 | -0.14 | NM_018029 | - |
| - | -0.15 | -0.55 | -0.02 | XM_498909 | - |
| POU2F1 | 0.32 | -0.54 | 0.34 | NM_002697 | POU domain, class 2, transcription factor 1 (Octamer-binding transcription factor 1) (Oct-1) (OTF-1) (NF-A1). [Source:Uniprot/SWISSPROT;Acc:P14859] |
| NP_060398.1 | 0.25 | -0.54 | 0.08 | NM_017928 | - |
| XP_372705.1 | 0.29 | -0.54 | -0.42 | XM_372705 | PREDICTED: similar to adenylate kinase (EC 2.7.4.3), cytosolic - common carp [Source:RefSeq_peptide;Acc:XP_372705] |
| - | -0.05 | -0.54 | -0.40 | - | - |
| - | 0.64 | -0.54 | 1.54 | XM_379741 | - |
| - | -0.28 | -0.54 | 0.18 | XM_499177 | - |
| OR10G3 | -0.35 | -0.54 | 0.03 | NM_001005465 | Olfactory receptor 10G3. [Source:Uniprot/SWISSPROT;Acc:Q8NGC4] |
| NP_848548.1 | 0.08 | -0.54 | -0.17 | NM_178453 | - |
| USH1G | -0.29 | -0.54 | 0.05 | NM_173477 | Usher syndrome 1G protein [Source:RefSeq_peptide;Acc:NP_775748] |
| Q6ZRU0_HUMAN | -0.36 | -0.54 | -0.26 | - | - |
| NP_997228.1 | -0.35 | -0.54 | -0.40 | NM_207345 | C-type lectin domain family 9, member A [Source:RefSeq_peptide;Acc:NP_997228] |
| ITIH2 | 0.52 | -0.54 | 1.71 | NM_002216 | Inter-alpha-trypsin inhibitor heavy chain H2 precursor (ITI heavy chain H2) (Inter-alpha-inhibitor heavy chain 2) (Inter-alpha-trypsin inhibitor complex component II) (Serum-derived hyaluronan-associated protein) (SHAP). [Source:Uniprot/SWISSPROT;Acc:P198 |
| NP_056139.1 | 0.51 | -0.54 | -0.14 | - | - |
| XP_372433.2 | 0.86 | -0.54 | -0.43 | XM_372433 | PREDICTED: similar to brain-specific homeodomain protein [Source:RefSeq_peptide;Acc:XP_372433] |
| - | -0.19 | -0.54 | -0.22 | XM_498481 | - |
| KCNJ12 | 0.97 | -0.54 | 0.02 | NM_021012 | Inward rectifying K(+) channel negative regulator Kir2.2v. [Source:Uniprot/SWISSPROT;Acc:Q15756] |
| NP_612209.1 | 0.04 | -0.54 | -0.01 | NM_138336 | helicase/primase complex protein [Source:RefSeq_peptide;Acc:NP_612209] |
| - | 0.04 | -0.54 | 0.28 | NM_138939,NM_138940 | - |
| - | 0.88 | -0.54 | -0.12 | XM_499143 | - |
| - | -0.04 | -0.54 | -0.12 | NM_031912,NM_181519 | - |
| Q8N1Y7_HUMAN | 0.05 | -0.54 | 0.98 | - | - |
| - | 0.15 | -0.54 | -0.30 | XM_495882 | - |
| C20orf131 | 0.12 | -0.54 | -0.47 | NM_152503,NM_213631 | - |
| GJA3 | 0.09 | -0.54 | -0.49 | NM_021954 | Gap junction alpha-3 protein (Connexin 46) (Cx46). [Source:Uniprot/SWISSPROT;Acc:Q9Y6H8] |
| XP_495835.1 | -0.24 | -0.54 | -0.40 | XM_495836 | PREDICTED: similar to bA182L21.1 (novel protein similar to hypothetical proteins) [Source:RefSeq_peptide;Acc:XP_374817] |
| - | 0.35 | -0.54 | -0.21 | - | 20 kDa protein [Source:IPI;Acc:IPI00411437] |
| GPR17 | 0.77 | -0.54 | 0.64 | NM_005291 | Probable P2Y purinoceptor GPR17 (P2Y-like receptor) (R12). [Source:Uniprot/SWISSPROT;Acc:Q13304] |
| - | -0.23 | -0.54 | -0.32 | NM_001001678 | - |
| - | -0.41 | -0.54 | -0.01 | XM_496545 | - |
| - | 0.32 | -0.54 | 0.28 | XM_499144 | - |
| - | -0.41 | -0.54 | -0.22 | - | Homo sapiens microRNA miR-218-1 stem-loop |
| POU2AF1 | 0.25 | -0.54 | -0.39 | NM_006235 | POU domain class 2, associating factor 1 (B-cell-specific coactivator OBF-1) (OCT binding factor 1) (BOB-1) (OCA-B). [Source:Uniprot/SWISSPROT;Acc:Q16633] |
| ALPP | -0.13 | -0.54 | -0.23 | NM_001632 | Alkaline phosphatase, placental type precursor (EC 3.1.3.1) (PLAP-1) (Regan isozyme). [Source:Uniprot/SWISSPROT;Acc:P05187] |
| PDE1A | 0.39 | -0.54 | 0.01 | NM_005019 | Calcium/calmodulin-dependent 3',5'-cyclic nucleotide phosphodiesterase 1A (EC 3.1.4.17) (Cam-PDE 1A) (61 kDa Cam-PDE) (hCam-1). [Source:Uniprot/SWISSPROT;Acc:P54750] |
| NR_002146.1 | 0.52 | -0.54 | 0.63 | NR_002146 | olfactory receptor, family 7, subfamily E, member 24 (OR7E24) on chromosome 19 [Source:RefSeq_dna;Acc:NR_002146] |
| NP_997397.1 | -0.01 | -0.54 | 0.41 | - | differentially expressed in FDCP 8 isoform 1 [Source:RefSeq_peptide;Acc:NP_997397] |
| DACT1 | 0.49 | -0.54 | 2.08 | NM_016651 | Dapper homolog 1 (hDPR1) (Heptacellular carcinoma novel gene-3 protein). [Source:Uniprot/SWISSPROT;Acc:Q9NYF0] |
| TM6SF2 | -0.27 | -0.54 | 0.53 | - | Transmembrane 6 superfamily member 2 (Fragment). [Source:Uniprot/SWISSPROT;Acc:Q9BZW4] |
| C9orf67 | 0.38 | -0.54 | 1.31 | NM_032728 | - |
| CHM | 1.20 | -0.54 | -0.47 | NM_000390 | Rab proteins geranylgeranyltransferase component A 1 (Rab escort protein 1) (REP-1) (Choroideraemia protein) (TCD protein). [Source:Uniprot/SWISSPROT;Acc:P24386] |
| - | -0.08 | -0.54 | 0.27 | XM_290342,XM_498816 | - |
| HEYL | 0.97 | -0.54 | 0.82 | NM_014571 | hairy/enhancer-of-split related with YRPW motif-like [Source:RefSeq_peptide;Acc:NP_055386] |
| CSNK1A1L | 0.89 | -0.54 | -0.38 | - | Casein kinase I, alpha-like isoform (EC 2.7.1.-) (CKI-alpha-like) (CK1). [Source:Uniprot/SWISSPROT;Acc:Q8N752] |
| XP_371590.2 | 0.04 | -0.54 | -0.48 | XM_371590 | PREDICTED: KIAA1571 protein [Source:RefSeq_peptide;Acc:XP_371590] |
| XP_033853.2 | -0.45 | -0.54 | -0.09 | XM_033853 | PREDICTED: zinc finger protein 630 [Source:RefSeq_peptide;Acc:XP_033853] |
| - | 1.85 | -0.54 | -0.19 | NM_014659 | 89 kDa protein [Source:IPI;Acc:IPI00479752] |
| HLA-DRB3 | 0.18 | -0.54 | 0.06 | NM_022555 | HLA class II histocompatibility antigen, DRB3-2 beta chain precursor (MHC class I antigen DRB3*2). [Source:Uniprot/SWISSPROT;Acc:P01913] |
| - | -0.45 | -0.54 | 0.00 | - | 31 kDa protein [Source:IPI;Acc:IPI00411749] |
| NP_997296.1 | -0.09 | -0.54 | 0.15 | NM_207413 | RPLK9433 [Source:RefSeq_peptide;Acc:NP_997296] |
| IFNA6 | 0.00 | -0.54 | -0.42 | NM_021002 | Interferon alpha-6 precursor (Interferon alpha-K) (LeIF K) (Interferon alpha-54). [Source:Uniprot/SWISSPROT;Acc:P05013] |
| NP_937796.1 | 0.75 | -0.53 | 1.04 | NM_198153 | triggering receptor expressed on myeloid cells-like 4 [Source:RefSeq_peptide;Acc:NP_937796] |
| PPP1R13B | -0.15 | -0.53 | -0.28 | NM_015316 | Apoptosis stimulating of p53 protein 1 (Protein phosphatase 1 regulatory subunit 13B). [Source:Uniprot/SWISSPROT;Acc:Q96KQ4] |
| FOXN1 | -0.12 | -0.53 | 0.63 | NM_003593 | Forkhead box protein N1 (Transcription factor winged-helix nude). [Source:Uniprot/SWISSPROT;Acc:O15353] |
| ATF2 | -0.20 | -0.53 | -0.17 | NM_001880 | Cyclic-AMP-dependent transcription factor ATF-2 (Activating transcription factor 2) (cAMP response element binding protein CRE- BP1) (HB16). [Source:Uniprot/SWISSPROT;Acc:P15336] |
| C2orf22 | -0.03 | -0.53 | -0.33 | NM_152391 | - |
| Q6ZT76_HUMAN | -0.08 | -0.53 | 0.16 | - | - |
| - | 0.81 | -0.53 | 0.10 | - | Non-protein coding transcript |
| - | 1.13 | -0.53 | 0.27 | - | Hypothetical protein |
| CCNB2 | -0.11 | -0.53 | -0.23 | NM_004701 | G2/mitotic-specific cyclin B2. [Source:Uniprot/SWISSPROT;Acc:O95067] |
| GHSR | 0.42 | -0.53 | 0.22 | NM_198407 | Growth hormone secretagogue receptor type 1 (GHS-R) (GH-releasing peptide receptor) (GHRP) (Ghrelin receptor). [Source:Uniprot/SWISSPROT;Acc:Q92847] |
| NP_001008783.1 | -0.20 | -0.53 | 0.43 | NM_001008783 | solute carrier family 35, member D3 [Source:RefSeq_peptide;Acc:NP_001008783] |
| - | 0.07 | -0.53 | 0.42 | - | 20 kDa protein [Source:IPI;Acc:IPI00478944] |
| NP_079328.2 | 0.38 | -0.53 | 0.52 | NM_025052 | - |
| NP_689885.3 | 1.53 | -0.53 | 0.97 | NM_152672 | organic solute transporter alpha [Source:RefSeq_peptide;Acc:NP_689885] |
| TCF21 | -0.10 | -0.53 | -0.36 | NM_198392 | Transcription factor 21 (Podocyte-expressed 1) (Pod-1) (Epicardin) (Capsulin). [Source:Uniprot/SWISSPROT;Acc:O43680] |
| WDR8 | 0.99 | -0.53 | -0.28 | NM_017818 | WD-repeat protein 8. [Source:Uniprot/SWISSPROT;Acc:Q9P2S5] |
| - | 0.12 | -0.53 | 0.48 | NM_001005499 | - |
| - | -0.44 | -0.53 | -0.22 | XM_496493 | - |
| NFIL3 | 0.71 | -0.53 | 1.88 | NM_005384 | nuclear factor, interleukin 3 regulated [Source:RefSeq_peptide;Acc:NP_005375] |
| Q9P1L5_HUMAN | 0.67 | -0.53 | 0.04 | - | - |
| NR5A2 | -0.23 | -0.53 | 0.09 | NM_205860 | Orphan nuclear receptor NR5A2 (Alpha-1-fetoprotein transcription factor) (Hepatocytic transcription factor) (B1-binding factor) (hB1F) (CYP7A promoter binding factor). [Source:Uniprot/SWISSPROT;Acc:O00482] |
| ARNT2 | 0.00 | -0.53 | -0.26 | NM_014862 | Aryl hydrocarbon receptor nuclear translocator 2 (ARNT protein 2). [Source:Uniprot/SWISSPROT;Acc:Q9HBZ2] |
| LRIG3 | 1.12 | -0.53 | 0.05 | NM_153377 | leucine-rich repeats and immunoglobulin-like domains 3 [Source:RefSeq_peptide;Acc:NP_700356] |
| HLA-DOB | 2.28 | -0.53 | -0.20 | NM_002120 | HLA class II histocompatibility antigen, DO beta chain precursor (MHC class II antigen DOB). [Source:Uniprot/SWISSPROT;Acc:P13765] |
| CNNM1 | -0.17 | -0.53 | 0.50 | NM_020348 | cyclin M1 [Source:RefSeq_peptide;Acc:NP_065081] |
| XP_291028.4 | 0.56 | -0.53 | -0.27 | XM_291028 | PREDICTED: hypothetical protein DKFZp434A128 [Source:RefSeq_peptide;Acc:XP_291028] |
| NP_872312.1 | 0.05 | -0.53 | 0.40 | NM_182506 | melanoma antigen, family B, 10 [Source:RefSeq_peptide;Acc:NP_872312] |
| - | 0.00 | -0.53 | -0.32 | - | 98 kDa protein [Source:IPI;Acc:IPI00477829] |
| KCNH8 | 0.56 | -0.53 | -0.27 | NM_144633 | Potassium voltage-gated channel subfamily H member 8 (Voltage-gated potassium channel subunit Kv12.1) (Ether-a-go-go like potassium channel 3) (ELK channel 3) (ELK3) (ELK1) (hElk1). [Source:Uniprot/SWISSPROT;Acc:Q96L42] |
| - | -0.27 | -0.53 | -0.23 | XM_496320 | - |
| - | -0.31 | -0.53 | -0.15 | - | Hypothetical protein |
| - | 0.13 | -0.53 | 0.64 | - | Homo sapiens microRNA miR-191 stem-loop |
| C13orf1 | 0.38 | -0.53 | -0.38 | NM_020456 | CLLL6 protein [Source:RefSeq_peptide;Acc:NP_065189] |
| NP_060243.1 | 0.49 | -0.53 | 1.04 | NM_017773 | - |
| TBX15 | 0.60 | -0.53 | 1.45 | NM_152380 | T-box transcription factor TBX15 (T-box protein 15). [Source:Uniprot/SWISSPROT;Acc:Q96SF7] |
| - | 0.34 | -0.53 | -0.28 | - | Non-protein coding transcript |
| KRT5 | 0.03 | -0.53 | 0.05 | NM_000424 | Keratin, type II cytoskeletal 5 (Cytokeratin 5) (K5) (CK 5) (58 kDa cytokeratin). [Source:Uniprot/SWISSPROT;Acc:P13647] |
| PHF15 | 1.09 | -0.53 | -0.32 | NM_015288 | PHD finger protein 15. [Source:Uniprot/SWISSPROT;Acc:Q9NQC1] |
| Q9NRW6_HUMAN | -0.24 | -0.53 | -0.11 | - | 17-beta-hydroxysteroid dehydrogenase type VII-like [Source:RefSeq_peptide;Acc:NP_878249] |
| COBLL1 | 0.32 | -0.53 | 0.72 | NM_014900 | COBL-like 1 [Source:RefSeq_peptide;Acc:NP_055715] |
| EPB41 | 0.15 | -0.53 | 4.07 | NM_203342,NM_004437,NM_203343 | Protein 4.1 (Band 4.1) (P4.1) (EPB4.1) (4.1R). [Source:Uniprot/SWISSPROT;Acc:P11171] |
| NP_073583.2 | 0.49 | -0.53 | -0.24 | NM_022746 | - |
| CD5 | -0.20 | -0.53 | 1.68 | NM_014207 | T-cell surface glycoprotein CD5 precursor (Lymphocyte glycoprotein T1/Leu-1) (Lymphocyte antigen CD5). [Source:Uniprot/SWISSPROT;Acc:P06127] |
| CYP2R1 | -0.19 | -0.53 | -0.18 | - | cytochrome P450, family 2, subfamily R, polypeptide 1 [Source:RefSeq_peptide;Acc:NP_078790] |
| - | 0.35 | -0.53 | -0.34 | XM_371267 | - |
| CLEC5_HUMAN | 0.04 | -0.53 | -0.38 | NM_013252 | C-type lectin superfamily member 5 (Myeloid DAP12-associating lectin) (MDL-1). [Source:Uniprot/SWISSPROT;Acc:Q9NY25] |
| XP_291726.3 | -0.12 | -0.53 | -0.47 | XM_291757,XM_291726 | PREDICTED: similar to protein of unknown function [Source:RefSeq_peptide;Acc:XP_291757] |
| PNLIPRP2 | 0.00 | -0.53 | 0.50 | NM_005396 | Pancreatic lipase related protein 2 precursor (EC 3.1.1.3). [Source:Uniprot/SWISSPROT;Acc:P54317] |
| - | -0.02 | -0.53 | -0.27 | XM_497823 | - |
| AMICA1 | -0.24 | -0.53 | 0.04 | NM_153206 | adhesion molecule, interacts with CXADR antigen 1 [Source:RefSeq_peptide;Acc:NP_694938] |
| LGALS4 | 0.06 | -0.53 | 0.10 | NM_006149 | Galectin-4 (Lactose-binding lectin 4) (L-36 lactose binding protein) (L36LBP) (Antigen NY-CO-27). [Source:Uniprot/SWISSPROT;Acc:P56470] |
| NP_940968.1 | -0.34 | -0.53 | -0.41 | NM_198566 | - |
| C1RL | 0.24 | -0.53 | 0.57 | NM_016546 | complement component 1, r subcomponent-like precursor [Source:RefSeq_peptide;Acc:NP_057630] |
| HT31_HUMAN | -0.25 | -0.53 | -0.36 | NM_015607 | Protein HT031 (pp7704). [Source:Uniprot/SWISSPROT;Acc:Q9Y3Y2] |
| - | 0.92 | -0.53 | -0.06 | XM_373076 | - |
| XM_292740.3 | 0.00 | -0.53 | 0.04 | XM_292740 | - |
| THRAP5 | -0.22 | -0.53 | 0.23 | NM_005481 | thyroid hormone receptor-associated protein 5 [Source:RefSeq_peptide;Acc:NP_005472] |
| LYSC_HUMAN | 0.11 | -0.53 | -0.37 | NM_000239 | Lysozyme C precursor (EC 3.2.1.17) (1,4-beta-N-acetylmuramidase C). [Source:Uniprot/SWISSPROT;Acc:P61626] |
| CCL7 | 0.56 | -0.53 | 0.50 | NM_006273 | Small inducible cytokine A7 precursor (CCL7) (Monocyte chemotactic protein 3) (MCP-3) (Monocyte chemoattractant protein 3) (NC28). [Source:Uniprot/SWISSPROT;Acc:P80098] |
| CHST9 | -0.03 | -0.53 | 0.01 | NM_031422 | GalNAc-4-sulfotransferase 2 [Source:RefSeq_peptide;Acc:NP_113610] |
| KIF17 | 0.24 | -0.53 | -0.32 | NM_020816 | Kinesin-like protein KIF17 (KIF3-related motor protein). [Source:Uniprot/SWISSPROT;Acc:Q9P2E2] |
| NP_659482.2 | 1.56 | -0.53 | 0.14 | NM_145045 | - |
| HPD | 0.44 | -0.53 | 0.62 | NM_002150 | 4-hydroxyphenylpyruvate dioxygenase (EC 1.13.11.27) (4HPPD) (HPD) (HPPDase). [Source:Uniprot/SWISSPROT;Acc:P32754] |
| - | 0.47 | -0.53 | 0.50 | NM_138959 | - |
| - | -0.19 | -0.53 | 0.12 | XM_211896 | - |
| AEBP1 | -0.03 | -0.52 | -0.31 | NM_001129 | adipocyte enhancer binding protein 1 precursor [Source:RefSeq_peptide;Acc:NP_001120] |
| LFNG | -0.40 | -0.52 | 0.30 | - | Beta-1,3-N-acetylglucosaminyltransferase lunatic fringe (EC 2.4.1.222) (O-fucosylpeptide 3-beta-N-acetylglucosaminyltransferase). [Source:Uniprot/SWISSPROT;Acc:Q8NES3] |
| CPNE8 | -0.08 | -0.52 | -0.50 | NM_153634 | Copine VIII. [Source:Uniprot/SWISSPROT;Acc:Q86YQ8] |
| - | 0.34 | -0.52 | -0.46 | XR_000275 | 14 kDa protein [Source:IPI;Acc:IPI00413661] |
| CD74 | -0.08 | -0.52 | 0.61 | - | HLA class II histocompatibility antigen, gamma chain (HLA-DR antigens associated invariant chain) (Ia antigen-associated invariant chain) (Ii) (p33) (CD74 antigen). [Source:Uniprot/SWISSPROT;Acc:P04233] |
| HSD3B2 | 0.73 | -0.52 | -0.12 | NM_000198 | 3 beta-hydroxysteroid dehydrogenase/delta 5-->4-isomerase type II (3Beta-HSD II) [Includes: 3-beta-hydroxy-delta(5)-steroid dehydrogenase (EC 1.1.1.145) (3-beta-hydroxy-5-ene steroid dehydrogenase) (Progesterone reductase); Steroid delta-isomerase (EC 5.3 |
| Q8N2R9_HUMAN | 0.17 | -0.52 | -0.39 | NM_032447 | Fibrillin 3 precursor. [Source:Uniprot/SWISSPROT;Acc:Q75N90] |
| DHX34 | -0.30 | -0.52 | -0.42 | NM_194428,NM_014681 | Probable ATP-dependent helicase DHX34 (DEAH-box protein 34). [Source:Uniprot/SWISSPROT;Acc:Q14147] |
| MKRN4 | 0.22 | -0.52 | -0.16 | - | Makorin 4 (Zinc finger protein 127-Xp) (ZNF127-Xp) (RING finger protein 64). [Source:Uniprot/SWISSPROT;Acc:Q13434] |
| - | 0.89 | -0.52 | 0.04 | - | - |
| - | 0.86 | -0.52 | 1.07 | - | - |
| - | -0.31 | -0.52 | -0.21 | XM_496359 | - |
| - | -0.41 | -0.52 | 0.46 | XM_498706,XM_294802 | - |
| - | -0.44 | -0.52 | -0.15 | - | Conserved hypothetical protein |
| EMR4 | 0.48 | -0.52 | 0.59 | XM_377506 | EGF-like module containing mucin-like hormone receptor-like 4 precursor (G-protein coupled receptor 127) (Fragment). [Source:Uniprot/SWISSPROT;Acc:Q86SQ3] |
| POU4F2 | -0.12 | -0.52 | 0.04 | NM_004575 | POU domain, class 4, transcription factor 2 (Brain-specific homeobox/POU domain protein 3B) (Brn-3B). [Source:Uniprot/SWISSPROT;Acc:Q12837] |
| PLXDC2 | 0.07 | -0.52 | 0.42 | NM_032812 | plexin domain containing 2 precursor [Source:RefSeq_peptide;Acc:NP_116201] |
| NP_055695.2 | -0.25 | -0.52 | 0.53 | NM_014880 | CD302 antigen [Source:RefSeq_peptide;Acc:NP_055695] |
| NUDT10 | 0.61 | -0.52 | -0.09 | NM_153183 | nudix (nucleoside diphosphate linked moiety X)-type motif 10 [Source:RefSeq_peptide;Acc:NP_694853] |
| APC10_HUMAN | 0.57 | -0.52 | 0.10 | - | Anaphase promoting complex subunit 10 (APC10) (Cyclosome subunit 10). [Source:Uniprot/SWISSPROT;Acc:Q9UM13] |
| - | 0.10 | -0.52 | -0.41 | - | 33 kDa protein [Source:IPI;Acc:IPI00261023] |
| - | 0.55 | -0.52 | 0.67 | - | 13 kDa protein [Source:IPI;Acc:IPI00477637] |
| XP_293034.1 | -0.04 | -0.52 | -0.49 | XM_293034 | PREDICTED: similar to RIKEN cDNA 2010316F05 [Source:RefSeq_peptide;Acc:XP_293034] |
| GSTA5 | 0.48 | -0.52 | 0.08 | NM_153699 | Glutathione S-transferase A5-5 (EC 2.5.1.18) (GST class-alpha). [Source:Uniprot/SWISSPROT;Acc:Q7RTV2] |
| PYCARD | 0.67 | -0.52 | 0.68 | NM_145183,NM_013258,NM_145182 | Apoptosis-associated speck-like protein containing a CARD (hASC) (PYD and CARD domain containing protein) (Target of methylation-induced silencing 1) (Caspase recruitment domain protein 5). [Source:Uniprot/SWISSPROT;Acc:Q9ULZ3] |
| - | -0.23 | -0.52 | -0.28 | XM_379036 | - |
| - | -0.28 | -0.52 | -0.43 | XM_499501 | - |
| RNF170 | 0.19 | -0.52 | 0.09 | - | ring finger protein 170 [Source:RefSeq_peptide;Acc:NP_112216] |
| BLK | 0.31 | -0.52 | -0.46 | NM_001715 | Tyrosine-protein kinase BLK (EC 2.7.1.112) (B lymphocyte kinase) (p55- BLK). [Source:Uniprot/SWISSPROT;Acc:P51451] |
| CD38 | 2.00 | -0.52 | 2.26 | NM_001775 | ADP-ribosyl cyclase 1 (EC 3.2.2.5) (Cyclic ADP-ribose hydrolase 1) (cADPr hydrolase 1) (Lymphocyte differentiation antigen CD38) (T10) (Acute lymphoblastic leukemia cells antigen CD38). [Source:Uniprot/SWISSPROT;Acc:P28907] |
| TNF | 0.01 | -0.52 | 0.39 | NM_000594 | Tumor necrosis factor precursor (TNF-alpha) (Tumor necrosis factor ligand superfamily member 2) (TNF-a) (Cachectin). [Source:Uniprot/SWISSPROT;Acc:P01375] |
| PSG9 | 0.54 | -0.52 | -0.16 | NM_002784 | Pregnancy-specific beta-1-glycoprotein 9 precursor (PSBG-9) (Pregnancy-specific glycoprotein 9) (Pregnancy-specific beta-1 glycoprotein B) (PS-beta-B) (PS34) (Pregnancy-specific glycoprotein 7) (PSG7). [Source:Uniprot/SWISSPROT;Acc:Q00887] |
| - | 0.13 | -0.52 | 0.35 | NM_213603 | - |
| - | 0.00 | -0.52 | -0.39 | XM_498213,XM_499443 | - |
| MRPS10 | -0.10 | -0.52 | -0.02 | NM_018141 | Mitochondrial 28S ribosomal protein S10 (S10mt) (MRP-S10) (MSTP040). [Source:Uniprot/SWISSPROT;Acc:P82664] |
| ZNF548 | 1.32 | -0.52 | 0.19 | NM_152909 | zinc finger protein 548 [Source:RefSeq_peptide;Acc:NP_690873] |
| THRA | -0.48 | -0.52 | -0.35 | NM_003250,NM_199334 | Thyroid hormone receptor alpha (C-erbA-alpha) (c-erbA-1) (EAR-7) (EAR7). [Source:Uniprot/SWISSPROT;Acc:P10827] |
| Q9H0V5_HUMAN | 0.00 | -0.52 | 0.52 | NM_003453,NM_197968 | - |
| PMF1 | -0.21 | -0.52 | -0.15 | NM_199173 | Osteocalcin precursor (Gamma-carboxyglutamic acid-containing protein) (Bone Gla-protein) (BGP). [Source:Uniprot/SWISSPROT;Acc:P02818] |
| XP_496688.1 | -0.46 | -0.52 | -0.50 | XM_496688 | PREDICTED: hypothetical protein BC012029 [Source:RefSeq_peptide;Acc:XP_496688] |
| CXorf27 | 0.27 | -0.52 | -0.27 | XM_048235 | PREDICTED: Huntingtin interacting protein M [Source:RefSeq_peptide;Acc:XP_048235] |
| FCER1G | -0.26 | -0.52 | -0.01 | NM_004106 | High affinity immunoglobulin epsilon receptor gamma-subunit precursor (FceRI gamma) (IgE Fc receptor gamma-subunit) (Fc-epsilon RI-gamma). [Source:Uniprot/SWISSPROT;Acc:P30273] |
| - | 0.80 | -0.52 | 1.16 | - | Hypothetical protein |
| MRPL38 | 0.36 | -0.52 | 0.39 | - | mitochondrial ribosomal protein L38 [Source:RefSeq_peptide;Acc:NP_115867] |
| O75863_HUMAN | 0.00 | -0.52 | -0.24 | - | Fos39347_1. [Source:Uniprot/SPTREMBL;Acc:O75863] |
| C6orf145 | -0.15 | -0.52 | -0.47 | NM_183373 | - |
| - | 0.31 | -0.52 | 0.33 | - | Non-protein coding transcript |
| USP6NL | 0.20 | -0.52 | 0.96 | XM_374768 | USP6 N-terminal like protein (Related to the N terminus of tre) (RN- tre). [Source:Uniprot/SWISSPROT;Acc:Q92738] |
| - | -0.10 | -0.52 | 0.24 | XM_211837 | - |
| - | -0.10 | -0.52 | -0.12 | XM_497832 | - |
| - | 0.71 | -0.52 | 0.12 | - | Hypothetical protein |
| - | -0.24 | -0.52 | -0.07 | - | Peroxisomal assembly protein PEX3 (Peroxin-3) |
| Q9H0X2_HUMAN | 0.48 | -0.52 | -0.47 | NM_206948,NM_001007470 | Transient receptor potential cation channel subfamily M member 3 (Long transient receptor potential channel 3) (LTrpC3) (Melastatin 2) (MLSN2). [Source:Uniprot/SWISSPROT;Acc:Q9HCF6] |
| - | 0.52 | -0.52 | 0.17 | - | 70 kDa protein [Source:IPI;Acc:IPI00183264] |
| MYL1 | 0.73 | -0.52 | -0.11 | - | Myosin light chain 1, skeletal muscle isoform (MLC1F) (A1 catalytic) (Alkali myosin light chain 1). [Source:Uniprot/SWISSPROT;Acc:P05976] |
| HHIP | -0.50 | -0.52 | -0.06 | NM_022475 | hedgehog-interacting protein [Source:RefSeq_peptide;Acc:NP_071920] |
| - | -0.30 | -0.52 | -0.32 | NM_001010896 | - |
| - | -0.03 | -0.52 | 1.31 | - | Hypothetical protein |
| ACOXL | -0.15 | -0.51 | 0.65 | - | acyl-Coenzyme A oxidase-like [Source:RefSeq_peptide;Acc:NP_060778] |
| TROAP | 0.23 | -0.51 | -0.18 | NM_005480 | Trophinin-associated protein (Tastin) (Trophinin-assisting protein). [Source:Uniprot/SWISSPROT;Acc:Q12815] |
| O51M1_HUMAN | -0.06 | -0.51 | 1.09 | NM_001004756 | Olfactory receptor 51M1 (HOR5'beta7). [Source:Uniprot/SWISSPROT;Acc:Q9H341] |
| - | -0.07 | -0.51 | 0.55 | NM_003868 | fibroblast growth factor 16 (FGF16), mRNA [Source:RefSeq_dna;Acc:NM_003868] |
| - | 0.89 | -0.51 | 0.83 | - | Non-protein coding transcript |
| PI3 | -0.14 | -0.51 | 0.05 | NM_002638 | Elafin precursor (Elastase-specific inhibitor) (ESI) (Skin-derived antileukoproteinase) (SKALP) (WAP four-disulfide core domain protein 14) (Protease inhibitor WAP3). [Source:Uniprot/SWISSPROT;Acc:P19957] |
| XRN1 | 0.15 | -0.51 | 0.89 | NM_019001 | 5'-3' exoribonuclease 1 [Source:RefSeq_peptide;Acc:NP_061874] |
| NP_775882.1 | 0.07 | -0.51 | 0.87 | NM_173611 | - |
| - | 0.07 | -0.51 | -0.34 | NM_001821 | - |
| - | -0.40 | -0.51 | -0.32 | - | Non-protein coding transcript |
| - | -0.30 | -0.51 | 0.24 | - | HMG-I and HMG-Y DNA-binding domain (A+T-hook) containing protein |
| N4BP2_HUMAN | -0.38 | -0.51 | -0.10 | NM_018177 | Nedd4-binding protein 2 (EC 3.-.-.-) (N4BP2) (BCL-3 binding protein). [Source:Uniprot/SWISSPROT;Acc:Q86UW6] |
| Q96NK6_HUMAN | 0.60 | -0.51 | -0.07 | - | - |
| CCAR1 | -0.07 | -0.51 | 0.15 | NM_018237 | cell-cycle and apoptosis regulatory protein 1 [Source:RefSeq_peptide;Acc:NP_060707] |
| SGKL | 1.00 | -0.51 | 0.05 | NM_013257,NM_170709 | Serine/threonine-protein kinase Sgk3 (EC 2.7.1.37) (Serum/glucocorticoid regulated kinase 3) (Serum/glucocorticoid regulated kinase-like). [Source:Uniprot/SWISSPROT;Acc:Q96BR1] |
| PI16 | -0.08 | -0.51 | -0.41 | NM_153370 | protease inhibitor 16 [Source:RefSeq_peptide;Acc:NP_699201] |
| NP_001010880.1 | -0.20 | -0.51 | 0.68 | - | - |
| MSR1 | 0.05 | -0.51 | -0.18 | NM_002445 | Macrophage scavenger receptor types I and II (Macrophage acetylated LDL receptor I and II) (CD204 antigen). [Source:Uniprot/SWISSPROT;Acc:P21757] |
| RAB37 | 0.41 | -0.51 | 0.34 | NM_175738,NM_001006637,NM_001006638 | Ras-related protein Rab-37. [Source:Uniprot/SWISSPROT;Acc:Q96AX2] |
| NDEL1 | -0.15 | -0.51 | -0.45 | NM_030808 | nudE nuclear distribution gene E homolog like 1 (A. nidulans) [Source:RefSeq_peptide;Acc:NP_110435] |
| SLC26A9 | 0.60 | -0.51 | 0.44 | NM_134325,NM_052934 | solute carrier family 26, member 9 isoform b [Source:RefSeq_peptide;Acc:NP_599152] |
| GNPDA2 | 0.10 | -0.51 | -0.06 | NM_138335 | glucosamine-6-phosphate deaminase 2 [Source:RefSeq_peptide;Acc:NP_612208] |
| NP_775941.1 | 0.32 | -0.51 | -0.31 | NM_173670 | RGM domain family, member B [Source:RefSeq_peptide;Acc:NP_775941] |
| - | 0.06 | -0.51 | -0.47 | XM_498987 | - |
| OSM | -0.06 | -0.51 | -0.44 | NM_020530 | Oncostatin M precursor (OSM). [Source:Uniprot/SWISSPROT;Acc:P13725] |
| - | 0.23 | -0.51 | 0.28 | XM_379651 | - |
| - | 0.00 | -0.51 | 0.91 | NM_000609 | - |
| - | 0.39 | -0.51 | 0.79 | XM_373600 | - |
| PTX3 | 0.42 | -0.51 | -0.47 | NM_002852 | Pentaxin-related protein PTX3 precursor (Tumor necrosis factor- inducible protein TSG-14). [Source:Uniprot/SWISSPROT;Acc:P26022] |
| ALDH1A1 | 0.10 | -0.51 | -0.33 | NM_000689 | Retinal dehydrogenase 1 (EC 1.2.1.36) (RalDH1) (RALDH 1) (Aldehyde dehydrogenase family 1 member A1) (Aldehyde dehydrogenase, cytosolic) (ALHDII) (ALDH-E1). [Source:Uniprot/SWISSPROT;Acc:P00352] |
| OR51F2 | 0.32 | -0.51 | 0.23 | NM_001004753 | Olfactory receptor 51F2. [Source:Uniprot/SWISSPROT;Acc:Q8NH61] |
| WFDC12 | 0.67 | -0.51 | 0.04 | NM_080869 | WAP four-disulfide core domain protein 12 precursor (Putative protease inhibitor WAP12) (UNQ544/PRO844). [Source:Uniprot/SWISSPROT;Acc:Q8WWY7] |
| CYFIP1 | 0.18 | -0.51 | 1.69 | NM_014608 | cytoplasmic FMR1 interacting protein 1 [Source:RefSeq_peptide;Acc:NP_055423] |
| - | 0.07 | -0.51 | -0.05 | NM_198849 | - |
| - | -0.04 | -0.51 | 0.62 | XM_497221 | - |
| - | -0.39 | -0.51 | 0.22 | - | Hypothetical protein |
| PDCD1LG2 | 0.38 | -0.51 | 0.54 | NM_025239 | programmed cell death 1 ligand 2 [Source:RefSeq_peptide;Acc:NP_079515] |
| PLSCR4 | 0.32 | -0.51 | 0.38 | NM_020353 | Phospholipid scramblase 4 (PL scramblase 4) (Ca(2+)-dependent phospholipid scramblase 4). [Source:Uniprot/SWISSPROT;Acc:Q9NRQ2] |
| - | 0.37 | -0.51 | -0.03 | XM_378316 | - |
| C14orf44 | -0.23 | -0.51 | 2.98 | NM_152445 | - |
| LYNX1 | 0.29 | -0.51 | -0.44 | NM_177457,NM_177476,NM_177477 | Ly-6/neurotoxin-like protein 1 precursor. [Source:Uniprot/SWISSPROT;Acc:Q9BZG9] |
| - | -0.20 | -0.51 | 0.18 | - | 7 kDa protein [Source:IPI;Acc:IPI00177651] |
| OTUB2_HUMAN | -0.09 | -0.51 | -0.30 | NM_023112 | Ubiquitin thiolesterase protein OTUB2 (EC 3.4.-.-) (Otubain 2) (OTU domain-containing ubiquitin aldehyde-binding protein 2) (Ubiquitin- specific processing protease OTUB2) (Deubiquitinating enzyme OTUB2). [Source:Uniprot/SWISSPROT;Acc:Q96DC9] |
| XM_377033.1 | -0.19 | -0.51 | -0.18 | - | similar to germ and embryonic stem cell enriched protein STELLA; stella-like (LOC401611), mRNA [Source:RefSeq_dna;Acc:XM_377033] |
| CPB2 | -0.35 | -0.51 | -0.46 | - | plasma carboxypeptidase B2 isoform b [Source:RefSeq_peptide;Acc:NP_057497] |
| - | 0.24 | -0.51 | 0.49 | NM_012313,NM_014513 | - |
| - | -0.16 | -0.51 | 0.10 | NM_001001661 | - |
| APOL1 | 0.64 | -0.51 | 0.08 | NM_145343 | Apolipoprotein-L1 precursor (Apolipoprotein L-I) (Apolipoprotein L) (ApoL-I) (Apo-L) (ApoL). [Source:Uniprot/SWISSPROT;Acc:O14791] |
| PGLYRP4 | 0.04 | -0.51 | 0.95 | NM_020393 | Peptidoglycan recognition protein I-beta precursor (Peptidoglycan recognition protein intermediate beta) (PGRP-I-beta) (PGLYRPIbeta) (Peptidoglycan recognition protein 4) (SBBI67). [Source:Uniprot/SWISSPROT;Acc:Q96LB8] |
| ALK | 0.14 | -0.51 | 0.41 | NM_004304 | ALK tyrosine kinase receptor precursor (EC 2.7.1.112) (Anaplastic lymphoma kinase) (CD246 antigen). [Source:Uniprot/SWISSPROT;Acc:Q9UM73] |
| Q69YU3_HUMAN | -0.04 | -0.51 | -0.33 | - | - |
| U9166_HUMAN | -0.46 | -0.51 | -0.22 | NM_001007538 | Protein UNQ9166/PRO28631 precursor. [Source:Uniprot/SWISSPROT;Acc:Q6UWI4] |
| RAPH1 | -0.09 | -0.51 | 0.00 | NM_025252,NM_213589,NM_203365 | Ras association and pleckstrin homology domains 1 isoform 1 [Source:RefSeq_peptide;Acc:NP_998754] |
| - | 0.40 | -0.51 | 1.55 | - | Hypothetical protein |
| - | -0.43 | -0.51 | 0.42 | - | Homo sapiens microRNA miR-331 stem-loop |
| KIAA0513 | 0.42 | -0.50 | 0.23 | NM_014732 | - |
| ACCN4 | -0.08 | -0.50 | 0.17 | NM_018674 | amiloride-sensitive cation channel 4 isoform 1 [Source:RefSeq_peptide;Acc:NP_061144] |
| NP_699187.1 | 0.89 | -0.50 | 0.02 | NM_153356 | TBC1 domain family, member 21 [Source:RefSeq_peptide;Acc:NP_699187] |
| - | -0.07 | -0.50 | 0.48 | - | Non-protein coding transcript |
| BRD3 | 0.46 | -0.50 | -0.42 | NM_007371 | Bromodomain-containing protein 3 (RING3-like protein). [Source:Uniprot/SWISSPROT;Acc:Q15059] |
| NP_060612.1 | -0.46 | -0.50 | -0.25 | NM_018142 | - |
| NPEPL1 | 0.69 | -0.50 | 0.67 | NM_024663 | Probable aminopeptidase NPEPL1 (EC 3.4.11.-) (Aminopeptidase-like 1). [Source:Uniprot/SWISSPROT;Acc:Q8NDH3] |
| Q8N1X1_HUMAN | -0.16 | -0.50 | 0.98 | - | - |
| - | -0.12 | -0.50 | 0.57 | XM_498210,XM_499440 | 12 kDa protein [Source:IPI;Acc:IPI00479642] |
| - | -0.47 | -0.50 | -0.30 | NM_145203 | - |
| - | 0.00 | -0.50 | 0.46 | XM_497406 | - |
| ZMYM1 | 0.66 | -0.50 | -0.26 | NM_024772 | zinc finger, MYM domain containing 1 [Source:RefSeq_peptide;Acc:NP_079048] |
| - | -0.13 | -0.50 | 0.31 | - | 35 kDa protein [Source:IPI;Acc:IPI00247806] |
| C20orf158 | 0.23 | -0.50 | 0.85 | NM_152302 | - |
| - | 0.85 | -0.50 | -0.29 | - | - |
| - | -0.30 | -0.50 | -0.01 | NM_015199 | - |
| - | 0.57 | -0.50 | 0.11 | XM_497386 | - |
| EVI5 | -0.14 | -0.50 | -0.08 | XM_496371,NM_005665 | ecotropic viral integration site 5 [Source:RefSeq_peptide;Acc:NP_005656] |
| RNF32 | 0.43 | -0.50 | 0.43 | - | ring finger protein 32 [Source:RefSeq_peptide;Acc:NP_112198] |
| SIM1 | 0.00 | -0.50 | -0.06 | NM_005068 | Single-minded homolog 1. [Source:Uniprot/SWISSPROT;Acc:P81133] |
| BCL10 | -0.06 | -0.50 | 0.64 | NM_003921 | B cell lymphoma/leukemia 10 (B-cell CLL/lymphoma 10) (Bcl-10) (CED- 3/ICH-1 prodomain homologous E10-like regulator) (CIPER) (CARD- containing molecule enhancing NFkappaB) (Cellular homolog of vCARMEN) (cCARMEN) (Mammalian CARD-containing adapter molecule |
| - | 0.67 | -0.50 | -0.29 | NM_019066 | - |
| - | 0.84 | -0.50 | 0.24 | XM_496889,XM_499312 | - |
| NP_079351.1 | -0.35 | -0.50 | -0.36 | NM_025075 | - |
| STX12 | -0.14 | -0.50 | -0.11 | NM_177424 | Syntaxin-12. [Source:Uniprot/SWISSPROT;Acc:Q86Y82] |
| IBTK | -0.14 | -0.50 | -0.43 | NM_015525 | inhibitor of Bruton's tyrosine kinase [Source:RefSeq_peptide;Acc:NP_056340] |
| Q7RTU7_HUMAN | -0.05 | -0.50 | -0.35 | - | Class II bHLH protein scleraxis. [Source:Uniprot/SPTREMBL;Acc:Q7RTU7] |

Supplementary table 2
