# Supplementary material for: Microarray Analysis on Human Neuroblastoma Cells Exposed to Aluminum, β1–42-Amyloid or the β1–42-Amyloid Aluminum Complex
Source: PLoS One. 2011 Jan 27;6(1):e15965. doi: 10.1371/journal.pone.0015965 (PMC3029275; doi:10.1371/journal.pone.0015965)
Supplement: Table S3 — List of the overexpressed genes found in the first network (see Fig. 2A ). (DOC) [file pone.0015965.s005.doc]

| ymbol | Entrez Gene Name | RefSeq | Log Ratio | Location | Family |
| --- | --- | --- | --- | --- | --- |
| ACTBL2 | actin, beta-like 2 | XM_293924 | 2.321 | unknown | other |
| AFP | alpha-fetoprotein | NM_001134 | 1.234 | Extracellular Space | transporter |
| Alcohol group acceptor phosphotransferase |  |  |  | unknown | group |
| APLP1 | amyloid beta (A4) precursor-like protein 1 | NM_005166 | 1.007 | Extracellular Space | other |
| APOM | apolipoprotein M | NM_019101 | 0.592 | Plasma Membrane | transporter |
| ARG1 | arginase, liver | NM_000045 | 0.507 | Cytoplasm | enzyme |
| ARRB1 | arrestin, beta 1 | NM_004041 | 0.847 | Cytoplasm | other |
| CCNT1 | cyclin T1 | NM_001240 | 1.09 | Nucleus | transcription regulator |
| CD5L | CD5 molecule-like | NM_005894 | 0.986 | Plasma Membrane | transmembrane receptor |
| Creb |  |  |  | unknown | group |
| EBAG9 | estrogen receptor binding site associated, antigen, 9 | NM_004215 | 0.6775 | Cytoplasm | other |
| GLP1R | glucagon-like peptide 1 receptor | NM_002062 | 0.554 | Plasma Membrane | G-protein coupled receptor |
| HAS2 | hyaluronan synthase2 | NM_005328 | 2.165 | Plasma Membrane | enzyme |
| HEXIM1 | hexamethylene bis-acetamide inducible 1 | NM_006460 | 0.571 | Nucleus | transcription regulator |
| Histone h3 |  |  |  | unknown | group |
| Histone h4 |  |  |  | unknown | group |
| ITCH | itchy E3 ubiquitin protein ligase homolog (mouse) | NM_031483 | 0.516 | Nucleus | enzyme |
| LASP1 | LIM and SH3 protein 1 | NM_006148 | 0.901 | Cytoplasm | transporter |
| LATS1 | large tumor suppressor, homolog | NM_004690 | 0.888 | Nucleus | kinase |
| LIMK1 | LIM domain kinase 1 | NM_002314 | 0.551 | Cytoplasm | kinase |
| MLANA | melan-A | NM_005511 | 0.501 | Plasma Membrane | other |
| MUC4 | mucin 4, cell surface associated | NM_018406 | 0.59 | Extracellular Space | growth factor |
| PELP1 | proline, glutamate and leucine rich protein 1 | NM_014389 | 0.8115 | Nucleus | other |
| PRKG1 | protein kinase, cGMP-dependent, type I | NM_006258 | 1.4115 | Cytoplasm | kinase |
| PRUNE2 | prune homolog 2 | XM_041018 | 2.284 | unknown | other |
| RNA polymerase II |  |  |  | Nucleus | complex |
| RRH | retinal pigment epithelium-derived rhodopsin homolog | NM_006583 | 1.236 | Plasma Membrane | G-protein coupled receptor |
| SAV1 | salvador homolog 1 | NM_021818 | 0.673 | unknown | other |
| SNTG1 | syntrophin, gamma 1 | NM_018967 | 1.044 | Nucleus | other |
| T | T, brachyury homolog (mouse) | NM_003181 | 0.628 | Nucleus | transcription regulator |
| TCOF1 | Treacher Collins-Franceschetti syndrome 1 | NM_000356 | 0.505 | Nucleus | transporter |
| TGFBR1 | transforming growth factor, beta receptor 1 | NM_004612 | 0.602 | Plasma Membrane | kinase |
| TNFSF10 | tumor necrosis factor (ligand) superfamily, member 10 | NM_003810 | 0.634 | Extracellular Space | cytokine |
| TOE1 | target of EGR1, member 1 (nuclear) | NM_025077 | 0.563 | Nucleus | other |
| TRPV6 | transient receptor potential cation channel, subfamily V, member 6 | NM_018646 | 0.588 | Plasma Membrane | ion channel |

Supplementary table 3
