# Supplementary material for: Microarray Analysis on Human Neuroblastoma Cells Exposed to Aluminum, β1–42-Amyloid or the β1–42-Amyloid Aluminum Complex
Source: PLoS One. 2011 Jan 27;6(1):e15965. doi: 10.1371/journal.pone.0015965 (PMC3029275; doi:10.1371/journal.pone.0015965)
Supplement: Table S4 — List of the overexpressed genes found in the second network (see Fig. 2B ). (DOC) [file pone.0015965.s006.doc]

| Symbol | Entrez Gene Name | RefSeq | Log Ratio | Location | Family |
| --- | --- | --- | --- | --- | --- |
| Adaptor protein 2 |  |  |  | Cytoplasm | complex |
| ALAS1 | aminolevulinate, delta-, synthase 1 | NM_000688 | 0.5125 | Cytoplasm | enzyme |
| Ap1 |  |  |  | Nucleus | complex |
| ATF7 | activating transcription factor 7 | NM_006856 | 0.5055 | Nucleus | transcription regulator |
| Clathrin |  |  |  | Cytoplasm | complex |
| CLTB | clathrin, light chain (Lcb) | NM_007097 | 0.7785 | Plasma Membrane | other |
| Collagen type I |  |  |  | unknown | complex |
| DNER | delta/notch-like EGF repeat containing | NM_139072 | 0.688 | Plasma Membrane | transmembrane receptor |
| EHMT2 | euchromatic histone-lysine N-methyltransferase 2 | NM_006709 | 0.653 | Nucleus | transcription regulator |
| ELF5 | E74-like factor 5 (ets domain transcription factor) | NM_198381 | 0.6565 | Nucleus | transcription regulator |
| EN1 | engrailed homeobox 1 | NM_001426 | 0.558 | Nucleus | transcription regulator |
| ETS |  |  |  | unknown | group |
| FOLR2 | folate receptor 2 (fetal) | NM_000803 | 2.219 | Plasma Membrane | transporter |
| GGA1 | golgi associated, gamma adaptin ear containing, ARF binding protein 1 | NM_001001560 | 1.861 | Cytoplasm | transporter |
| HIPK3 | homeodomain interacting protein kinase 3 | NM_005734 | 0.503 | Nucleus | kinase |
| HMGA2 | high mobility group AT-hook 2 | NM_003483 | 0.809 | Nucleus | other |
| HMOX1 | heme oxygenase (decycling) 1 | NM_002133 | 0.83 | Cytoplasm | enzyme |
| HNRNPL | heterogeneous nuclear ribonucleoprotein L | NM_001005335 | 0.5245 | Nucleus | other |
| IFN Beta |  |  |  | unknown | group |
| ITGA2 | integrin, alpha 2 (CD49B, alpha 2 subunit of VLA-2 receptor) | NM_002203 | 1.702 | Plasma Membrane | other |
| JUN | jun oncogene | NM_002228 | 0.906 | Nucleus | transcription regulator |
| LAIR1 | leukocyte-associated immunoglobulin-like receptor 1 | NM_021706 | 0.5375 | Plasma Membrane | transmembrane receptor |
| LMO2 | LIM domain only 2 (rhombotin-like 1) | NM_005574 | 0.692 | Nucleus | other |
| MAFG | v-maf musculoaponeurotic fibrosarcoma oncogene homolog G (avian) | NM_032711 | 1.147 | Nucleus | transcription regulator |
| NFkB (complex) |  |  |  | Nucleus | complex |
| NHLH2 | nescient helix loop helix 2 | NM_005599 | 1.174 | Nucleus | other |
| NOS1 | nitric oxide synthase 1 (neuronal) | NM_000620 | 1.0085 | Cytoplasm | enzyme |
| NOS2 | nitric oxide synthase 2, inducible | NM_000625 | 1.865 | Cytoplasm | enzyme |
| PRDM1 | PR domain containing 1, with ZNF domain | NM_001198 | 0.5 | Nucleus | transcription regulator |
| RSAD2 | radical S-adenosyl methionine domain containing 2 | NM_080657 | 0.703 | unknown | enzyme |
| SLC19A1 | solute carrier family 19 (folate transporter), member 1 | NM_194255 | 0.625 | Plasma Membrane | transporter |
| SPIB | Spi-B transcription factor (Spi-1/PU.1 related) | NM_003121 | 0.729 | Nucleus | transcription regulator |
| SYT3 | synaptotagmin III | NM_032298 | 1.27 | Cytoplasm | transporter |
| Thyroid hormone receptor |  |  |  | unknown | group |
| TPM2 | tropomyosin 2 (beta) | NM_213674 | 0.608 | Cytoplasm | other |

Supplementary table 4
