# Supplementary material for: Microarray Analysis on Human Neuroblastoma Cells Exposed to Aluminum, β1–42-Amyloid or the β1–42-Amyloid Aluminum Complex
Source: PLoS One. 2011 Jan 27;6(1):e15965. doi: 10.1371/journal.pone.0015965 (PMC3029275; doi:10.1371/journal.pone.0015965)
Supplement: Table S5 — List of the overexpressed genes found in the third network (see Fig. 2C ). (DOC) [file pone.0015965.s007.doc]

| Symbol | Entrez Gene Name | RefSeq | Log Ratio | Location | Family |
| --- | --- | --- | --- | --- | --- |
| ACTA1 | actin, alpha 1, skeletal muscle | NM_001100 | 0.542 | Cytoplasm | other |
| ACTC1 | actin, alpha, cardiac muscle 1 | NM_005159 | 0.577 | Cytoplasm | enzyme |
| CELA3B | chymotrypsin-like elastase family, member 3B | NM_007352 | 1.085 | Extracellular Space | peptidase |
| CENPO | centromere protein O | NM_024322 | 1.085 | unknown | other |
| CHRND | cholinergic receptor, nicotinic, delta | NM_000751 | 0.715 | Plasma Membrane | transmembrane receptor |
| Cyclin A |  |  |  | unknown | group |
| E2f |  |  |  | unknown | group |
| ECE1 | endothelin converting enzyme 1 | NM_001397 | 0.512 | Plasma Membrane | peptidase |
| EPC1 | enhancer of polycomb homolog 1 | NM_025209 | 0.9825 | Nucleus | transcription regulator |
| FASLG | Fas ligand (TNF superfamily, member 6) | NM_000639 | 0.591 | Extracellular Space | cytokine |
| Filamin |  |  |  | unknown | group |
| FURIN | furin (paired basic amino acid cleaving enzyme) | NM_002569 | 2.092 | Cytoplasm | peptidase |
| G-Actin |  |  |  | unknown | group |
| GNRH1 | gonadotropin-releasing hormone 1 | NM_000825 | 0.601 | Extracellular Space | other |
| HDAC9 (includes EG:9734) | histone deacetylase 9 | NM_178423 | 0.8555 | Nucleus | transcription regulator |
| Hydrolase |  |  |  | unknown | group |
| IDE | insulin-degrading enzyme | NM_004969 | 1.052 | Extracellular Space | peptidase |
| Insulin |  |  |  | unknown | group |
| MEF2 |  |  |  | unknown | group |
| MEF2C | myocyte enhancer factor2C | NM_002397 | 0.575 | Nucleus | transcription regulator |
| MYOG | myogenin | NM_002479 | 0.648 | Nucleus | transcription regulator |
| NCKIPSD | NCK interacting protein with SH3 domain | NM_016453 | 0.574 | Nucleus | other |
| NR4A1 | nuclear receptor subfamily 4, group A, member 1 | NM_173157 | 0.97 | Nucleus | ligand-dependent nuclear receptor |
| PACSIN2 | protein kinase C and casein kinase substrate in neurons 2 | NM_007229 | 0.534 | Cytoplasm | transporter |
| PAPL | iron/zinc purple acid phosphatase-like protein | NM_001004318 | 1.363 | unknown | enzyme |
| PAPPA | pregnancy-associated plasma protein A, pappalysin 1 | NM_002581 | 1.262 | Extracellular Space | peptidase |
| PCSK1 | proprotein convertase subtilisin/kexin type 1 | NM_000439 | 0.511 | Extracellular Space | peptidase |
| PDX1 (includes EG:3651) | pancreatic and duodenal homeobox 1 | NM_000209 | 1.182 | Nucleus | transcription regulator |
| peptidase |  |  |  | unknown | group |
| POU3F1 | POU class 3 homeobox 1 | NM_002699 | 0.505 | Nucleus | transcription regulator |
| Rb |  |  |  | unknown | group |
| SMYD1 | SET and MYND domain containing 1 | NM_198274 | 0.909 | Nucleus | transcription regulator |
| TBC1D10A | TBC1 domain family, member 10A | NM_031937 | 0.854 | Plasma Membrane | other |
| THTPA | thiamine triphosphatase | NM_024328 | 1.431 | Cytoplasm | phosphatase |
| WDR76 | WD repeat domain 76 | NM_024908 | 3.124 | Nucleus | other |

Supplementary table 5
