# Supplementary material for: Microarray Analysis on Human Neuroblastoma Cells Exposed to Aluminum, β1–42-Amyloid or the β1–42-Amyloid Aluminum Complex
Source: PLoS One. 2011 Jan 27;6(1):e15965. doi: 10.1371/journal.pone.0015965 (PMC3029275; doi:10.1371/journal.pone.0015965)
Supplement: Table S6 — List of the overexpressed genes found in the fourth network (see Fig. 2D ). (DOC) [file pone.0015965.s008.doc]

| Symbol | Entrez Gene Name | RefSeq | Log Ratio | Location | Family |
| --- | --- | --- | --- | --- | --- |
| Actin |  |  |  | unknown | group |
| ADRB1 | adrenergic, beta-1-, receptor | NM_000684 | 0.732 | Plasma Membrane | G-protein coupled receptor |
| AIFM3 | apoptosis-inducing factor, mitochondrion-associated, 3 | NM_144704 | 0.867 | Cytoplasm | enzyme |
| ATP2B4 | ATPase, Ca++ transporting, plasma membrane 4 | NM_001001396 | 0.5715 | Plasma Membrane | transporter |
| Calmodulin |  |  |  | unknown | group |
| CAMK2A | calcium/calmodulin-dependent protein kinase II alpha | NM_171825 | 0.5465 | Cytoplasm | kinase |
| CaMKII |  |  |  | Cytoplasm | complex |
| CASK | calcium/calmodulin-dependent serine protein kinase (MAGUK family) | NM_003688 | 0.8655 | Plasma Membrane | kinase |
| CDK5R2 | cyclin-dependent kinase 5, regulatory subunit 2 (p39) | NM_003936 | 0.768 | Nucleus | other |
| EEF2K | eukaryotic elongation factor-2 kinase | NM_013302 | 1.007 | Cytoplasm | kinase |
| EPB41L1 | erythrocyte membrane protein band 4.1-like 1 | NM_012156 | 1.064 | Plasma Membrane | other |
| Ephb |  |  |  | unknown | group |
| GPLD1 | glycosylphosphatidylinositol specific phospholipase D1 | NM_001503 | 0.7035 | Cytoplasm | enzyme |
| GRIN2C | glutamate receptor, ionotropic, N-methyl D-aspartate 2C | NM_000835 | 1.131 | Plasma Membrane | ion channel |
| HLA-DQA1 | major histocompatibility complex, class II, DQ alpha1 | NM_002122 | 0.702 | Plasma Membrane | transmembrane receptor |
| ILDR1 | immunoglobulin-like domain containing receptor1 | NM_175924 | 0.544 | Plasma Membrane | transmembrane receptor |
| KALRN | kalirin, RhoGEF kinase | NM_003947 | 0.631 | Cytoplasm | kinase |
| LAMB2 | laminin, beta 2 (laminin S) | NM_002292 | 0.7225 | Extracellular Space | enzyme |
| MYH14 | myosin, heavy chain 14, non-muscle | NM_024729 | 0.557 | Extracellular Space | other |
| MYH7B (includes EG:57644) | myosin, heavy chain 7B, cardiac muscle, beta | NM_020884 | 0.579 | unknown | other |
| MYH8 | myosin, heavy chain 8, skeletal muscle, perinatal | NM_002472 | 0.854 | Cytoplasm | other |
| Myosin |  |  |  | Cytoplasm | complex |
| NMDA Receptor |  |  |  | Plasma Membrane | complex |
| NRXN1 | neurexin 1 | NM_138735 | 1.204 | Plasma Membrane | transporter |
| Oxidoreductase |  |  |  | unknown | group |
| PLA2 |  |  |  | unknown | group |
| PLA2G2C | phospholipase A2, group IIC | XM_372769 | 0.612 | Extracellular Space | enzyme |
| PLA2G2D | phospholipase A2, group IID | NM_012400 | 1.24 | Extracellular Space | enzyme |
| PLA2G3 | phospholipase A2, group III | NM_015715 | 0.544 | Extracellular Space | enzyme |
| PLA2G4E | phospholipase A2, group IVE | XM_373627 | 0.523 | Cytoplasm | enzyme |
| Pld |  |  |  | unknown | group |
| PLD2 | phospholipase D2 | NM_002663 | 0.732 | Cytoplasm | enzyme |
| PP2A |  |  |  | Cytoplasm | complex |
| STRN | striatin, calmodulin binding protein | NM_003162 | 0.885 | Cytoplasm | other |
| STXBP1 | syntaxin binding protein 1 | NM_003165 | 1.333 | Cytoplasm | transporter |

Supplementary table 6
