# Supplementary material for: Microarray Analysis on Human Neuroblastoma Cells Exposed to Aluminum, β1–42-Amyloid or the β1–42-Amyloid Aluminum Complex
Source: PLoS One. 2011 Jan 27;6(1):e15965. doi: 10.1371/journal.pone.0015965 (PMC3029275; doi:10.1371/journal.pone.0015965)
Supplement: Table S7 — List of the downexpressed genes found in the first network (see Fig. 3A ). (DOC) [file pone.0015965.s009.doc]

| Symbol | Entrez Gene Name | RefSeq | Log Ratio |
| --- | --- | --- | --- |
| AEBP1 | AE binding protein 1 | NM_001129 | -0.524 |
| AGAP2 | ArfGAP with GTPase domain, ankyrin repeat and PH domain 2 | NM_014770 | -1.1065 |
| CD36 | CD36 molecule (thrombospondin receptor) | NM_001001548 | -0.613 |
| CITED1 | Cbp/p300-interacting transactivator, with Glu/Asp-rich carboxy-terminal domain, 1 | NM_004143 | -0.709 |
| Collagen type I |  |  |  |
| Collagen(s) |  |  |  |
| CYP4F2 | cytochrome P450, family 4, subfamily F, polypeptide 2 | NM_001082 | -0.648 |
| CYP7A1 | cytochrome P450, family 7, subfamily A, polypeptide 1 | NM_000780 | -0.98 |
| DNM3 | dynamin 3 | NM_015569 | -0.903 |
| EP300 | E1A binding protein p300 | NM_001429 | -1.018 |
| ESR1 | estrogen receptor 1 | NM_000125 | -0.5954999999999999 |
| Estrogen Receptor |  |  |  |
| GRIK1 | glutamate receptor, ionotropic, kainate 1 | NM_000830 | -0.7085 |
| Homer |  |  |  |
| HOMER2 | homer homolog 2 (Drosophila) | NM_199330 | -0.669 |
| HOMER3 | homer homolog 3 (Drosophila) | NM_004838 | -0.696 |
| HSD3B2 | hydroxy-delta-5-steroid dehydrogenase, 3 beta- and steroid delta-isomerase 2 | NM_000198 | -0.523 |
| LCOR | ligand dependent nuclear receptor corepressor | NM_032440 | -0.869 |
| MED16 | mediator complex subunit 16 | NM_005481 | -0.525 |
| MN1 | meningioma (disrupted in balanced translocation) 1 | NM_002430 | -0.558 |
| N4BP2 | NEDD4 binding protein 2 | NM_018177 | -0.512 |
| NR5A2 | nuclear receptor subfamily 5, group A, member 2 | NM_205860 | -0.530 |
| OPHN1 | oligophrenin 1 | NM_002547 | -0.721 |
| OSM | oncostatin M | NM_020530 | -0.509 |
| POU4F1 | POU class 4 homeobox 1 | NM_006237 | -1.102 |
| POU4F2 | POU class 4 homeobox 2 | NM_004575 | -0.522 |
| Rar |  |  |  |
| Rxr |  |  |  |
| SHANK3 | SH3 and multiple ankyrin repeat domains 3 | XM_037493 | -0.723 |
| SORBS3 | sorbin and SH3 domain containing 3 | NM_005775 | -0.643 |
| THRA | thyroid hormone receptor, alpha (erythroblastic leukemia viral (v-erb-a) oncogene homolog, avian) | NM_003250 | -0.519 |
| THRSP | thyroid hormone responsive (SPOT14 homolog, rat) | NM_003251 | -0.709 |
| Thyroid hormone receptor |  |  |  |
| UCP1 | uncoupling protein 1 (mitochondrial, proton carrier) | NM_021833 | -0.551 |
| ZNF384 | zinc finger protein 384 | NM_133476 | -1.031 |

Supplementary table 7
