# Supplementary material for: Microarray Analysis on Human Neuroblastoma Cells Exposed to Aluminum, β1–42-Amyloid or the β1–42-Amyloid Aluminum Complex
Source: PLoS One. 2011 Jan 27;6(1):e15965. doi: 10.1371/journal.pone.0015965 (PMC3029275; doi:10.1371/journal.pone.0015965)
Supplement: Table S8 — List of the downexpressed genes found in the second network (see Fig. 3B ). (DOC) [file pone.0015965.s010.doc]

| Symbol | Entrez Gene Name | RefSeq | Log Ratio | Location | Family |
| --- | --- | --- | --- | --- | --- |
| ACTG2 (includes EG:72) | actin, gamma 2, smooth muscle, enteric | NM_001615 | -0.673 | Cytoplasm | other |
| Actin |  |  |  | unknown | group |
| Alpha Actinin |  |  |  | unknown | group |
| CAMK4 | calcium/calmodulin-dependent protein kinase IV | NM_001744 | -0.557 | Nucleus | kinase |
| CDC14A | CDC14 cell division cycle 14 homolog A (S. cerevisiae) | NM_003672 | -0.789 | Nucleus | phosphatase |
| CDH1 | cadherin 1, type 1, E-cadherin (epithelial) | NM_004360 | -0.733 | Plasma Membrane | other |
| CHM | choroideremia (Rab escort protein 1) | NM_000390 | -0.537 | Cytoplasm | enzyme |
| CHML | choroideremia-like (Rab escort protein 2) | NM_001821 | -0.513 | Cytoplasm | enzyme |
| CRYAA | crystallin, alpha A | NM_000394 | -1.066 | Nucleus | other |
| CRYAB | crystallin, alpha B | NM_001885 | -0.989 | Nucleus | other |
| CTDP1 | CTD (carboxy-terminal domain, RNA polymerase II, polypeptide A) phosphatase, subunit 1 | NM_004715 | -0.578 | Nucleus | phosphatase |
| CUL4A | cullin 4A | NM_003589 | -0.793 | Nucleus | other |
| DCAF11 | DDB1 and CUL4 associated factor 11 | NM_025230 | -0.627 | unknown | other |
| DSG1 | desmoglein 1 | NM_001942 | -0.562 | Plasma Membrane | other |
| EPB41 | erythrocyte membrane protein band 4.1 (elliptocytosis 1, RH-linked) | NM_203342 | -0.528 | Plasma Membrane | other |
| ERMN | ermin, ERM-like protein | XM_371576 | -0.654 | Extracellular Space | other |
| F Actin |  |  |  | Cytoplasm | complex |
| MED15 | mediator complex subunit 15 | NM_001003891 | -0.98 | Nucleus | transcription regulator |
| MEN1 | multiple endocrine neoplasia I | NM_130803 | -0.8684999999999999 | Nucleus | transcription regulator |
| MLPH | melanophilin | NM_024101 | -0.552 | Cytoplasm | other |
| Myosin |  |  |  | Cytoplasm | complex |
| NOV | nephroblastoma overexpressed gene | NM_002514 | -0.936 | Extracellular Space | growth factor |
| PLEC1 | plectin 1, intermediate filament binding protein 500kDa | NM_201383 | -0.7354999999999999 | Cytoplasm | other |
| POLR2L (includes EG:5441) | polymerase (RNA) II (DNA directed) polypeptide L, 7.6kDa | NM_021128 | -1.189 | Nucleus | enzyme |
| PP2A |  |  |  | Cytoplasm | complex |
| RAB17 | RAB17, member RAS oncogene family | NM_022449 | -0.775 | Cytoplasm | enzyme |
| RAB27A | RAB27A, member RAS oncogene family | NM_004580 | -0.559 | Cytoplasm | enzyme |
| RAVER1 | ribonucleoprotein, PTB-binding 1 | NM_133452 | -0.793 | Nucleus | other |
| RNA polymerase II |  |  |  | Nucleus | complex |
| S100A8 | S100 calcium binding protein A8 | NM_002964 | -0.776 | Cytoplasm | other |
| SNAI1 | snail homolog 1 | NM_005985 | -0.661 | Nucleus | other |
| Tubulin |  |  |  | Cytoplasm | complex |
| USP6NL | USP6 N-terminal like | XM_374768 | -0.516 | Plasma Membrane | other |
| VANGL1 | vang-like 1 | NM_138959 | -0.525 | Cytoplasm | other |
| VARS | valyl-tRNA synthetase | NM_006295 | -0.6 | Cytoplasm | enzyme |

Supplementary table 8
