# Supplementary material for: Microarray Analysis on Human Neuroblastoma Cells Exposed to Aluminum, β1–42-Amyloid or the β1–42-Amyloid Aluminum Complex
Source: PLoS One. 2011 Jan 27;6(1):e15965. doi: 10.1371/journal.pone.0015965 (PMC3029275; doi:10.1371/journal.pone.0015965)
Supplement: Table S9 — List of the downexpressed genes found in the third network (see Fig. 3C ). (DOC) [file pone.0015965.s011.doc]

| Symbol | Entrez Gene Name | RefSeq | Log Ratio | Location | Family |
| --- | --- | --- | --- | --- | --- |
| 14-3-3 |  |  |  | unknown | group |
| ADIPOQ | adiponectin, C1Q and collagen domain containing | NM_004797 | -0.916 | Extracellular Space | other |
| Akt |  |  |  | unknown | group |
| Alcohol group acceptor phosphotransferase |  |  |  | unknown | group |
| BAG4 | BCL2-associated athanogene 4 | NM_004874 | -0.699 | Cytoplasm | other |
| BGLAP | bone gamma-carboxyglutamate (gla) protein | NM_199173 | -0.5185 | Extracellular Space | other |
| BLK | B lymphoid tyrosine kinase | NM_001715 | -0.52 | Cytoplasm | kinase |
| BRAF | v-raf murine sarcoma viral oncogene homolog B1 | NM_004333 | -0.669 | Cytoplasm | enzyme |
| C5ORF34 | chromosome 5 open reading frame 34 | NM_198566 | -0.526 | unknown | other |
| CCAR1 | cell division cycle and apoptosis regulator 1 | NM_018237 | -0.512 | Nucleus | other |
| Cyclin A |  |  |  | unknown | group |
| FYB | FYN binding protein (FYB-120/130) | NM_199335 | -0.601 | Nucleus | other |
| GABRG2 | gamma-aminobutyric acid (GABA) A receptor, gamma 2 | NM_000816 | -0.61 | Plasma Membrane | ion channel |
| GAD |  |  |  | unknown | group |
| Histone h3 |  |  |  | unknown | group |
| Histone h4 |  |  |  | unknown | group |
| Hsp70 |  |  |  | unknown | group |
| Hsp90 |  |  |  | unknown | group |
| ID3 | inhibitor of DNA binding 3, dominant negative helix-loop-helix protein | NM_002167 | -0.738 | Nucleus | transcription regulator |
| LAX1 | lymphocyte transmembrane adaptor 1 | NM_017773 | -0.529 | Cytoplasm | other |
| LCP2 | lymphocyte cytosolic protein 2 (SH2 domain containing leukocyte protein of 76kDa) | NM_005565 | -0.563 | Cytoplasm | other |
| MOS | v-mos Moloney murine sarcoma viral oncogene homolog | NM_005372 | -0.793 | Cytoplasm | kinase |
| MYL2 | myosin, light chain 2, regulatory, cardiac, slow | NM_000432 | -1.398 | Cytoplasm | other |
| NKX2-5 | NK2 transcription factor related, locus 5 (Drosophila) | NM_004387 | -1.8195 | Nucleus | transcription regulator |
| PECAM1 | platelet/endothelial cell adhesion molecule | NM_000442 | -1.557 | Plasma Membrane | other |
| PIK3R1 | phosphoinositide-3-kinase, regulatory subunit 1 (alpha) | NM_181524 | -0.5635 | Cytoplasm | kinase |
| PITX2 | paired-like homeodomain 2 | NM_153426 | -0.814 | Nucleus | transcription regulator |
| Pkc(s) |  |  |  | unknown | group |
| PRKCB | protein kinase C, beta | NM_212535 | -0.652 | Cytoplasm | kinase |
| PRKCE | protein kinase C, epsilon | NM_005400 | -0.663 | Cytoplasm | kinase |
| Raf |  |  |  | unknown | group |
| SHANK2 | SH3 and multiple ankyrin repeat domains 2 | XM_495888 | -0.585 | Cytoplasm | other |
| SOCS6 | suppressor of cytokine signaling 6 | NM_004232 | -0.685 | Cytoplasm | other |
| TCL1A | T-cell leukemia/lymphoma 1A | NM_021966 | -1.324 | Nucleus | other |
| TTK | TTK protein kinase | NM_003318 | -1.02 | Nucleus | kinase |

Supplementary table 9
