# Supplementary material for: Microarray Analysis on Human Neuroblastoma Cells Exposed to Aluminum, β1–42-Amyloid or the β1–42-Amyloid Aluminum Complex
Source: PLoS One. 2011 Jan 27;6(1):e15965. doi: 10.1371/journal.pone.0015965 (PMC3029275; doi:10.1371/journal.pone.0015965)
Supplement: Table S10 — List of the downexpressed genes found in the fourth network (see Fig. 3D ). (DOC) [file pone.0015965.s012.doc]

| Symbol | Entrez Gene Name | RefSeq | Log Ratio | Location | Family |
| --- | --- | --- | --- | --- | --- |
| 3',5'-cyclic-nucleotide phosphodiesterase |  |  |  | unknown | group |
| CA2 | carbonic anhydrase II | NM_000067 | -1.871 | Cytoplasm | enzyme |
| CD163 | CD163 molecule | NM_004244 | -0.655 | Plasma Membrane | transmembrane receptor |
| CD226 | CD226 molecule | NM_006566 | -0.991 | Plasma Membrane | other |
| CD3 |  |  |  | Plasma Membrane | complex |
| CD38 | CD38 molecule | NM_001775 | -0.52 | Plasma Membrane | enzyme |
| CD3E | CD3e molecule, epsilon (CD3-TCR complex) | NM_000733 | -0.636 | Plasma Membrane | transmembrane receptor |
| CD5 | CD5 molecule | NM_014207 | -0.527 | Plasma Membrane | transmembrane receptor |
| CD8B | CD8b molecule | NM_004931 | -0.6885 | Plasma Membrane | other |
| Ck2 |  |  |  | Cytoplasm | complex |
| CTSS | cathepsin S | NM_004079 | -0.596 | Cytoplasm | peptidase |
| EPS8L1 | EPS8-like 1 | NM_133180 | -0.957 | unknown | other |
| F2 | coagulation factor II (thrombin) | NM_000506 | -0.581 | Extracellular Space | peptidase |
| Fcer1 |  |  |  | Plasma Membrane | complex |
| FCER1G | Fc fragment of IgE, high affinity I, receptor for; gamma polypeptide | NM_004106 | -0.518 | Plasma Membrane | transmembrane receptor |
| G protein beta gamma |  |  |  | Cytoplasm | complex |
| GST |  |  |  | Nucleus | complex |
| GSTA5 | glutathione S-transferase alpha 5 | NM_153699 | -0.521 | Cytoplasm | enzyme |
| GSTO2 | glutathione S-transferase omega 2 | NM_183239 | -0.97 | unknown | enzyme |
| HAVCR1 | hepatitis A virus cellular receptor 1 | NM_012206 | -1.008 | Plasma Membrane | other |
| Hemoglobin |  |  |  | unknown | complex |
| HLA-DOB | major histocompatibility complex, class II, DO beta | NM_002120 | -0.53 | Plasma Membrane | transmembrane receptor |
| KCNJ3 | potassium inwardly-rectifying channel, subfamily J, member 3 | NM_002239 | -0.573 | Plasma Membrane | ion channel |
| LGALS4 | lectin, galactoside-binding, soluble, 4 | NM_006149 | -0.526 | Extracellular Space | other |
| MHC Class I (complex) |  |  |  | Plasma Membrane | complex |
| MHC Class II |  |  |  | Plasma Membrane | complex |
| MME | membrane metallo-endopeptidase | NM_007289 | -0.931 | Plasma Membrane | peptidase |
| PDE1A | phosphodiesterase 1A, calmodulin-dependent | NM_005019 | -0.538 | Cytoplasm | enzyme |
| PDE4B | phosphodiesterase 4B, cAMP-specific | NM_002600 | -1.636 | Cytoplasm | enzyme |
| PDE6A | phosphodiesterase 6A, cGMP-specific, rod, alpha | NM_000440 | -0.589 | Plasma Membrane | enzyme |
| peptidase |  |  |  | unknown | group |
| PTPN22 | protein tyrosine phosphatase, non-receptor type 22 (lymphoid) | NM_015967 | -0.815 | Cytoplasm | phosphatase |
| PTPRC | protein tyrosine phosphatase, receptor type, C | NM_080923 | -1.536 | Plasma Membrane | phosphatase |
| SLC4A1 | solute carrier family 4, anion exchanger, member 1 | NM_000342 | -0.627 | Plasma Membrane | transporter |
| TCR |  |  |  | Plasma Membrane | complex |

Supplementary table 10
